# 1,3‐Brook Rearrangement of α‐Silyl Allylic Alcohols: Copper‐Catalyzed Enantiospecific Formation of Allenes and Copper‐Mediated *ipso*‐Substitution Reactions Affording Trisubstituted Alkenes

**DOI:** 10.1002/anie.5791239

**Published:** 2026-09-10

**Authors:** Jack L. Sutro, Felix Anderl, Paul Haf‐Moths, Alois Fürstner

**Affiliations:** ^1^ Max‐Planck‐Institut für Kohlenforschung Mülheim an der Ruhr Germany; ^2^ Institut für Biologische Chemie Universität Wien Vienna Austria

**Keywords:** allenes, Brook rearrangement, hydrosilylation, organocopper chemistry, Peterson elimination, ruthenium

## Abstract

A novel and convenient set of conditions is reported for the generation of alkenylcopper reagents via 1,3‐Brook rearrangement of alkenylsilane derivatives formed by ruthenium catalyzed *trans*‐hydrosilylation of propargyl alcohol derivatives. The reactive intermediates can evolve in a stereospecific manner into allenes; this new approach is catalytic in copper and proceeds with faithful chirality transfer from the stereocenter of the substrate to the axially chiral product. The overall stereochemical course resulting from *trans*‐hydrosilylation followed by an *anti*‐elimination step is complementary to that of most protocols for allene synthesis starting from propargylic alcohols known in the literature. Alternatively, the transient organocopper species can be intercepted with a variety of electrophiles to give a wide range of substituted alkene derivatives. In this latter embodiment, the sequence of hydrosilylation/Brook rearrangement presents an opportunity for regioselective net *trans*‐hydrofunctionalization of alkynes, a field that has so far been dominated by less benign organotin chemistry.

## Graphical abstract

A regioselective ruthenium catalyzed *trans*‐hydrosilylation of propargyl alcohols followed by a 1,3‐Brook rearrangement opens access to reactive organocopper intermediates, which evolve upon warming into allenes with strict chirality transfer to the emerging axis; alternatively, these reactive species can be trapped with a large variety of electrophiles to furnish diversely substituted allylic alcohol derivatives.

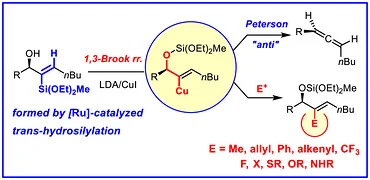

## 1 Introduction

Our group has a longstanding interest in the stereo‐ and regioselective hydrofunctionalization of alkynes [1, 2]. Ruthenium catalyzed *trans‐*hydrostannylation is currently the method of choice in the context of complex target synthesis because of the wide scope, the generally excellent chemo‐, regio‐, and stereoselectivity, and the mild and diverse downstream chemistry of the organotin compounds thus formed (Scheme 1A) [3, 4, 5, 6]. By contrast, the analogous *trans*‐hydrosilylation catalyzed by [Cp*Ru] complexes, though similarly broadly applicable [7, 8, 9, 10, 11, 12], has so far offered fewer opportunities for postfunctionalization of the resulting products; it has been largely confined to protodesilylation, oxidation of the C─Si bond, or C_sp_^2^–C_sp_^2^ cross coupling [13, 14, 15]. Many of these reactions rely on fluoridic conditions, which can be inappropriate for delicate substrates and incompatible with the protecting group strategies commonly used in advanced organic synthesis.

**SCHEME 1:**
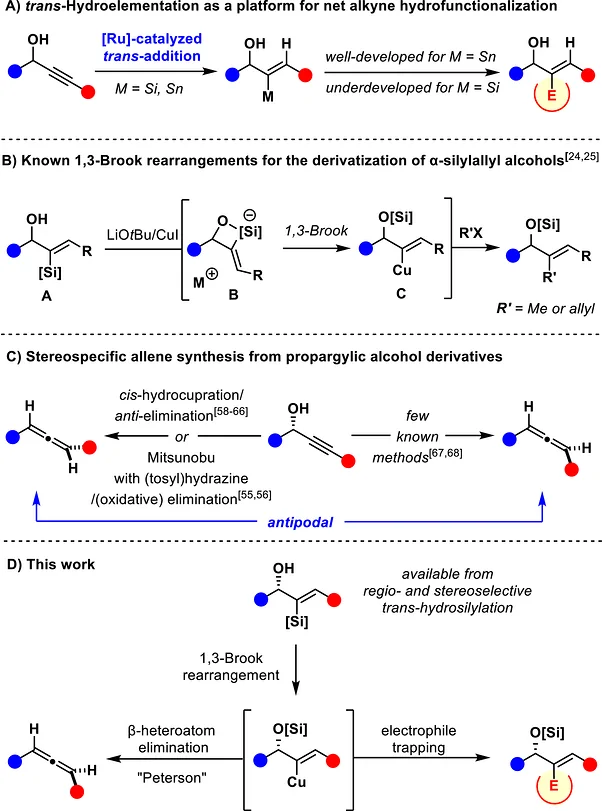
(A) The prior work on *trans‐*hydrofunctionalization by ruthenium catalyzed, hydroxy‐directed *trans*‐hydrometalation as the key step; (B) the prior work on the 1,3‐Brook rearrangement of α‐silylated allyl alcohols; (C) existing stereospecific allene syntheses; and (D) a summary of this work.

We conjectured that the Brook rearrangement might be a serious alternative, as it allows reactive organometallic species to be generated via C─Si bond activation by an internal oxygen nucleophile under comparatively mild conditions [16, 17, 18, 19, 20, 21]. Products of type **A**, which are readily available from propargylic alcohols by *trans*‐hydrosilylation catalyzed by [Cp*RuCl]_4_ [4, 5, 22, 23], are indeed amenable to a 1,3‐Brook rearrangement as had originally been shown by the Takeda group [24], followed by a more recent contribution from our laboratory (Scheme 1B) [25]. In these studies, stoichiometric amounts of CuI in combination with LiO*t*Bu were used to promote the formation of alkenylcopper species **C**, presumably via a transient four‐membered intermediate **B**. However, only the most reactive alkylating agents such as allyl halides or iodomethane were found suitable to trap the organocopper reagents thus formed. Moreover, it is essential that these electrophiles are added to the reaction without delay; if the narrow time window of just a few seconds is missed, protodesilylation, elimination, and/or additional unidentified side reactions start to set in [25]. No advanced applications of this methodology have been reported, whereas the related 1,4‐Brook rearrangements of β‐silylated allylic alcohols found more echo, even though the range of suitable electrophilic traps also remained very limited [26, 27, 28, 29, 30, 31, 32, 33, 34, 35, 36, 37].

Therefore, we decided to make a new attempt at addressing these issues and expanding the scope of the 1,3‐Brook rearrangement/functionalization sequence with the hope of developing a platform for the net *trans*‐hydrofunctionalization of propargyl alcohols that is more robust and convenient as well as (much) broader in scope with respect to the electrophilic partners. In addition, it seemed possible to deliberately engage the reactive intermediates generated in situ by the Brook rearrangement in β‐heteroatom elimination [38], which would result in allene formation via a formal Peterson reaction (Scheme 1D) [39, 40, 41, 42, 43, 44]. Since allenes are the subject of burgeoning interest as synthetic intermediates and themselves occur within a number of natural products [45, 46], the development of a novel entry route into this chemical estate starting from stable and well accessible substrates seems to be a worthwhile endeavor, especially if it complements the existing arsenal in stereochemical terms (Scheme 1C) [47, 48].

## 2 Results and Discussion

### 2.1 A New Stereospecific Allene Synthesis

Following our previous work, alkenylsilane **1** comprising a di(ethoxy)methylsilyl group was chosen as the model substrate, since this particular silyl substituent had provided a good balance of reactivity and stability [25]. Specifically, (EtO)_2_MeSiH is highly effective and selective in the ruthenium catalyzed, hydroxy‐directed *trans*‐hydrosilylation of propargyl alcohols in terms of yield and regioselectivity; moreover, the resulting products are fairly robust and easy to handle, while the electron‐withdrawing EtO− groups lower the energy of the C−Si antibonding orbitals, which benefits attack by an adjacent alkoxide as needed to trigger the envisaged 1,3‐Brook rearrangement. It is important to note, however, that other silyl groups have previously been identified that are also suitable for this purpose, including Ph_3_Si–, Me_2_(EtO)Si–, and (EtO)_3_Si– [24, 25].

We began our study by employing the known stoichiometric LiO*t*Bu/CuI system [24, 25] and simply omitted the electrophile while allowing the putative alkenylcopper intermediate **D** derived from alkenylsilane **1** to evolve at room temperature. This did indeed give rise to allene **2**, although it was formed in a ≈1:1 ratio with compound **3** as the product of protodecupration (Table 1, entry 1). This side reaction might be caused by the *tert*‐butanol formed during the initial deprotonation, which is apparently acidic enough to quench the organometallic intermediate formed. Proton back‐transfer needs to be precluded such that β‐heteroatom elimination can proceed unimpeded. To this end, mesitylcopper [49], one of the few isolable and thermally fairly robust organocopper reagents known in the literature, was tested but found suboptimal for a number of reasons [50]. Next, we turned to stronger and more hindered bases. The combination of LiHMDS/CuI (2.0 equiv. each) indeed gave allene **2** as the exclusive product. Some insight into the course of the reaction was gained by initiating the reaction at −78°C and monitoring while warming the mixture slowly to room temperature. The starting alkoxide converted to a new species at approximately −30°C, which yielded **3** quantitatively on aqueous quenching. Therefore, this intermediate was assigned as an alkenylcopper species of type **D** formed by the actual 1,3‐Brook rearrangement. This reactive intermediate appeared (meta)stable below −20°C, above which it was observed to convert into the allene product **2**.

**TABLE 1:** Optimization of allene formation by Brook rearrangement^a^.

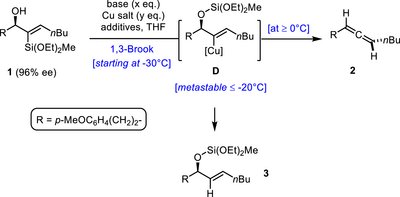		
Entry	Conditions	Result
1	LiO*t*Bu, CuI (2 eq. each)	**2** + **3** (≈1:1, 72%)
2	[Cu(NEt_2_)]_4_	**2** + **3** (≈60:40, < 10%)^b^
3	LiHMDS, CuI (2 eq. each)	**2** (90%, 94% ee)
4	LiHMDS (2 eq.), CuI (20 mol%)	**2** (≈25%)
5	LiHMDS (2 eq.), CuBr·SMe_2_ (20 mol.%)	**2** (≈25%)
6	LiHMDS (2 eq.), CuI (20 mol%), LiI (5 eq.)	**2** (88%, 85% ee)
7	LiHMDS (2 eq.), CuI (20 mol%), LiCl (5 eq.)	**2** (82%, 84% ee)
8	LiHMDS (2 eq.), CuI (20 mol%), NMI (10% *v/v*)	**2** (93%, 94% ee)
9	LiHMDS (2 eq.), CuI (20 mol%), NEt_3_ (10% *v/v*)	**2** (93%, 94% ee)
10	LiHMDS (1.2 eq.), CuI (20 mol%), NEt_3_ (10% *v/v*)	**2** + **3** (≈ 1:1; 89%)
11	LDA (1.1 eq.), CuI (20 mol%), NEt_3_ (10% *v/v*)	**2** (88%, 94% ee)

^a^ Conditions: A solution of the base (0.1 M in THF) was added to a solution of **1** (1.0 eq.) in THF at 0°C. The additive and copper salt were added subsequently, and the mixture was stirred at 0°C for 2–3 h before being quenched.

^b^ < 10% conversion after 3 h reaction time at ≤ 0°C.

Next, the preformed copper amide [Cu(NEt_2_)]_4_ (1.5 equiv. of the monomeric subunit) was tested but found to be less adequate in that no conversion was observed at −30°C independent of whether THF or THF/DMPU was used as the reaction medium. This lack of reactivity might be due, at least in part, to the poor solubility of the reagent at this low temperature. At 0°C, allene formation proceeded much more slowly than with LiHMDS/CuI, even though full conversion was eventually reached upon stirring of the mixture at ambient temperature. In this context it is important to note that [Cu(NEt_2_)]_4_ in THF/DMPU is the reagent of choice for the regio‐complementary net *trans*‐hydrofunctionalization of substrates bearing the silyl substituent distal to the ─OH group. The transient alkenylcopper reagents formed upon 1,4‐Brook rearrangement cannot undergo elimination with the formation of an allene product; rather, they are well‐behaved nucleophiles vis‐à‐vis a large range of electrophilic partners as outlined in detail in the accompanying paper [51].

On enantioenriched starting material (96% ee), the LiHMDS/CuI system gave a yield of 90% and an ee of 94% (entry 3). Although this was a viable set of conditions, we conjectured that catalytic amounts of Cu(I) should suffice to prompt the reaction. Attempts to lower the Cu(I) loading to 20 mol%, however, resulted in poor yields of allene **2**, along with some **3** and significant amounts of an intractable mixture of decomposition products after an extended reaction time; this outcome was independent of whether CuI or CuBr·SMe_2_ was used as the copper source (entries 4 and 5). Slow addition of the pre‐formed lithium alkoxide to a solution of catalytic CuX did not entail higher turnover numbers either. Based on these observations, we reasoned that the copper silanolate species primarily formed by the nominal Peterson elimination might be catalytically incompetent and its transformation back into CuI (too) slow (Scheme 2). In an attempt to promote this apparently critical step, excess LiI (or LiCl) was added to the mixture, which indeed rendered the reaction catalytic in copper; however, the stereospecificity suffered in that the ee of **2** was only 85% (84%) (entries 6 and 7). More successful was the use of aprotic‐dipolar cosolvents as an alternative way of promoting the regeneration of CuI by salt metathesis. Thus, the addition of *N*‐methylimidazole (NMI) or triethylamine (10% *v/v*) to the mixture gave satisfactory outcomes in that the central chirality of the substrate (96% ee) was faithfully relayed to the chiral axis of **2** (94% ee) (entries 8, 9) [52].

**SCHEME 2:**
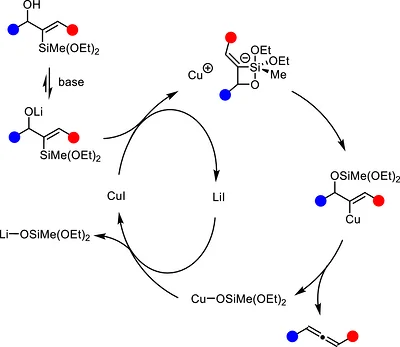
Mechanistic proposal for allene formation triggered by a 1,3‐Brook rearrangement, which is co‐catalytic in Cu(+1) and LiI.

Finally, we sought to lower the amount of LiHMDS in order to better accommodate base‐sensitive substrates. With only 1.2 equivalents, however, protodecupration with the formation of the undesired alkene **3** predominated (entry 10). This unfavorable outcome suggested that the deprotonation of **1** is incomplete due to similar pK_a_ ranges of secondary alcohols and the H‐HMDS generated in situ [53]; in the resulting equilibrium, the incipient organocopper species is evidently quenched at a competitive rate. To remedy the issue, we resorted to the more basic LDA (1.1–1.2 equiv.), which proved capable of effecting the transformation while retaining the yield and enantioselectivity when the reaction was performed at 0°C; an excellent chirality conversion relaying the stereogenic center of the substrate to the chiral axis of the allene product was observed, with an enantiospecificity of 98% (entry 11) [54]. Lowering the CuI loading to 10% was inconsequential with regard to the yield and optical purity of the product, but the reaction time approximately doubled; as CuI is cheap, we opted to explore the scope of the reaction for the sake of convenience with a 20% loading in combination with triethylamine as the additive (see below).

The stereochemical sense of the transformation was determined by subjecting the propargyl alcohol **4** (from which alkenylsilane **1** derives) to the allene synthesis developed by Myers and coworkers [55, 56]. Upon treatment with PPh_3_, DEAD and *o*‐nitrobenzenesulfonylhydrazine in THF, *ent*‐**2** was obtained in good yield (for details, see the Supporting Information) [57]. As the stereochemical course of this method is well‐established, that of our new procedure can be inferred as a corollary (Scheme 3). On the basis of the thus proven absolute configuration of **2**, one can deduce that the presumed alkenylcopper species **D** generated from **1** via 1,3‐Brook rearrangement must have undergone an *anti*‐elimination; this conclusion is consistent with earlier observations made in allene syntheses passing through organocopper reagents [58, 59, 60, 61, 62, 63, 64, 65, 66]. Re‐subjection of allene **2** to the reaction conditions led to no further erosion of enantiomeric excess, implying that the minor loss of stereochemical integrity occurs most likely as a result of slightly imperfect *anti*‐selectivity in the nominal Peterson elimination step.

**SCHEME 3:**
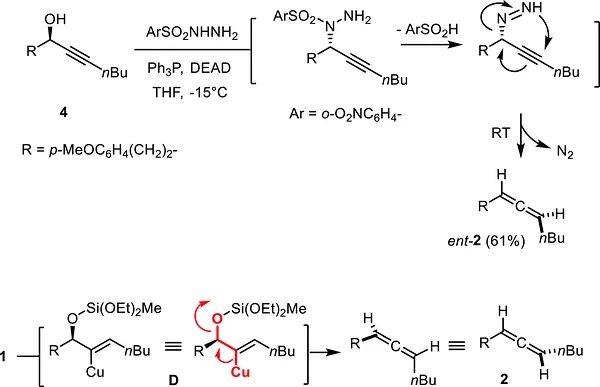
Stereochemical corollary.

The stereospecificity of the new method is deemed a major asset because it is complementary to the course of most commonly used protocols for allene synthesis starting from propargylic substrates. This includes not only the method of Myers et al. used above as the stereochemical reference point [55, 56], but also a number of well‐established procedures that rely on *cis*‐hydrocupration/*anti*‐elimination [58, 59, 60, 61, 62, 63, 64, 65, 66]. In contrast to this literature precedent, our new approach constitutes a formal *trans*‐hydrocupration/*anti*‐elimination event that is hardly known: an early example is found in a stepwise procedure starting off with a radical *trans*‐hydrostannation of a propargyl alcohol, followed by acylation of the resulting product and subsequent fluoride induced *anti*‐elimination [67]; our new method is a shorter and much more benign alternative. Yet another allene synthesis directly from a propargylic alcohol with the same overall stereospecificity is a stoichiometric method developed by the Ready group: [68] in this case, the substrate is first deprotonated with either EtMgBr or EtZnCl and the resulting alkoxide subjected to hydrozirconation with Cp_2_Zr(H)Cl; for the oxophilicity of Zr(+4), a *syn*‐elimination ensues that leads to the allene product. The net outcome of *cis*‐hydrozirconation/*syn*‐elimination is the same as that of the *trans*‐hydrosilylation/*anti*‐elimination sequence described herein. For the mild reaction conditions and the use of only cheap, benign, and non‐reducing reagents, however, the new catalytic method is deemed a genuine alternative.

The examples compiled in Scheme 4 illustrate the robustness and functional group compatibility of this new allene synthesis (for the preparation of the substrates, see the Supporting Information). Various polar substituents survived the reaction unscathed, be they on an aryl ring or an alkyl substituent (─Cl, ─CN, ─OMe, ─OTBS, ─OAc). Among them, the compatibility of the acetate in **9** and the lactone in **14** is arguably most noteworthy, as these groups are enolizable; they could themselves react with LDA or serve as internal proton sources for the transient organocopper species but did not interfere. The clean conversion of a macrocyclic propargylic alcohol to the corresponding endocyclic allene **14** is remarkable per se and has little precedent [69]. Likewise, arylallene **12** as well as the trisubstituted allene **13** were readily formed. In all but two cases, faithful chirality transfer from the stereogenic center of the optically active substrates to the chiral axis of the corresponding allene product was observed with only minimal loss in optical purity. The only exceptions were the allenes **11a**,**b**, in which partial or even complete loss of the optical purity was observed. We suppose that (partial) racemization of the extended conjugated π‐system in the presence of copper is responsible for this outcome [70, 71]. For the sake of completeness, yet another limitation of ruthenium catalyzed *trans*‐hydrosilylations—and hence also of this new allene synthesis—needs to be mentioned: the reaction usually fails with propargyl alcohols whose triple bond is flanked by a quaternary center [5].

**SCHEME 4:**
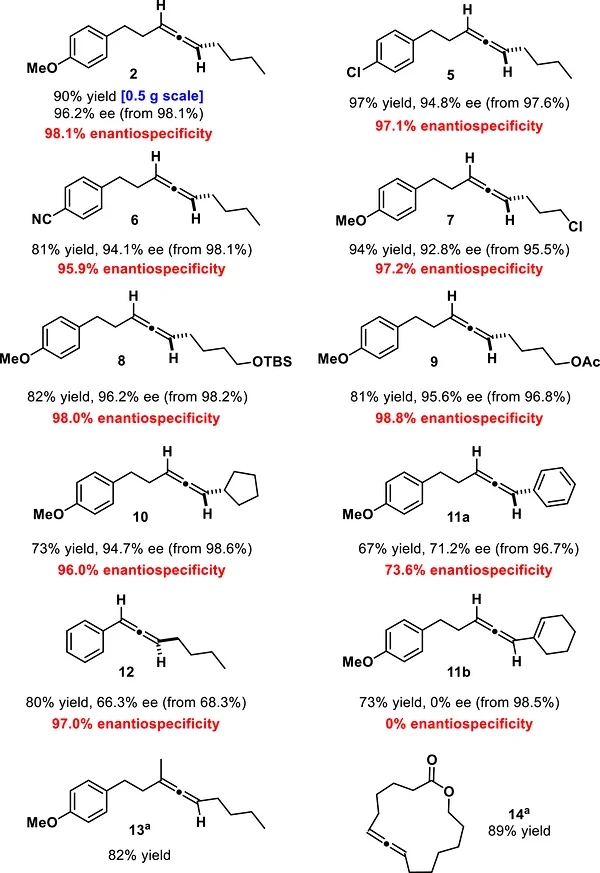
Substrate scope for the allene formation. The reactions were performed on a 0.05–0.1 mmol scale unless stated otherwise. ^a^Using racemic starting material. The enantiospecificity describing the chirality transfer is defined as the quotient of the ee of the allene and the ee of the substrate.

### 2.2 *trans*‐Hydrofunctionalization

We had observed during the initial phase of this study that the alkenylcopper intermediate **D** was formed at about −30°C and appeared to be metastable when the temperature was kept below −20°C. It struck us that trapping of this organometallic species might offer synthetic opportunities beyond allene formation. This would allow us to revisit the methodology put forth by Takeda and coworkers and later by our own group, both of which have previously pursued such an approach to generate substituted olefins but were facing the problem that the range of suitable electrophilic partners was extremely narrow and the conditions not overly practical [24, 25]. Provided that the scope of the reaction can be extended, a versatile platform for net *trans‐*hydrofunctionalization of propargylic alcohols using organosilicon chemistry might be reached, which could rival the powerful but less benign *trans*‐hydrostannylation commonly used for such purposes [72, 73, 74, 75, 76, 77, 78, 79, 80, 81, 82]. While the higher reactivity of the C−Sn bond is advantageous in purely chemical terms, it comes at the price of having to use toxic reagents; moreover, problems with protodestannylation during the actual functionalization step have occasionally been encountered. We thus endeavored to generate intermediates of type **D** in a stoichiometric fashion and assess their reactivity with a range of electrophiles (Scheme 5).

**SCHEME 5:**
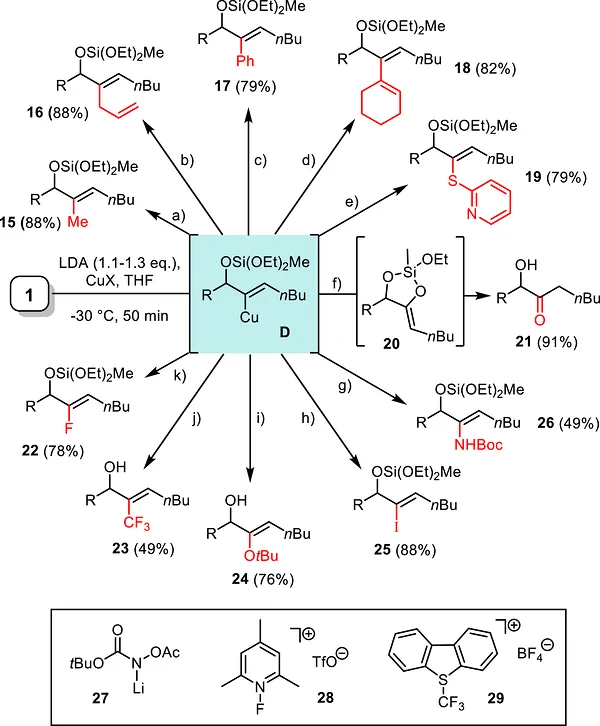
Trapping of the transient organocopper species with different electrophiles (R = *p*‐MeOC_6_H_4_(CH_2_)_2_–); conditions were generally: **1**, LDA, THF, −30°C, 5 min; then Cu salt (2.0 eq.) 50 min; then electrophile. (a) with CuI, quenched with MeI (3.0 eq.); (b) with CuI, quenched with allyl bromide (3.0 eq.); (c) with CuI, quenched with PhI (3.0 eq.) and Pd(PPh_3_)_4_ (10 mol%), warmed to rt after 1 h; (d) with CuI, quenched with cyclohexenyl triflate (3.0 eq.) and Pd(PPh_3_)_4_ (10 mol%), warmed to rt after 1 h; (e) with [Cu(MeCN)_4_]BF_4_, quenched with PySSPy (2.0 eq.); (f) with CuBr·SMe_2_, quenched with LiOO*t*Bu (3.0 eq.) at −78°C; (g) with CuI, quenched with BocN(Li)OAc (**27**, 1.8 eq.) at −78°C; (h) with CuI, quenched with I_2_ (2.0 eq.); (i) with [Cu(MeCN)_4_]BF_4_, quenched with *t*BuOOBz (2.0 eq.), then stirred with K_2_CO_3_/MeOH; j) with [Cu(MeCN)_4_]BF_4_, quenched with Umemoto's reagent (**29**, 2.0 eq.), then treated with aq. HCl (1 M)/THF at rt; (k) with [Cu(MeCN)_4_]BF_4_, quenched with fluorocollidinium triflate (**28**, 2.0 eq.).

Treatment of the organocopper species **D** formed at −30°C with iodomethane yielded the methylated product **15** in 88% yield, a result that correlates with our earlier findings [25]. Note that the resulting *E*‐configured 2‐methyl‐but‐2‐en‐1‐ol motif is very common in polyketidic natural products; this new entry is hence deemed enabling [83, 84, 85, 86, 87, 88, 89]. Likewise, the use of allyl bromide furnished the expected skipped diene **16** in an equally good yield [24]. Higher alkyl halides such as iodoethane and benzyl bromide turned out to be insufficiently electrophilic to participate in the desired C─C bond formation at low temperature; instead, mixtures of the allene **2** and protodecupration product **3** were obtained upon warming. Beyond alkylation reactions, cross‐coupling chemistry proved successful; thus, the addition of Pd(PPh_3_)_4_ and either iodobenzene or cyclohexenyl triflate gave the corresponding styrene derivative **17** and diene **18**, respectively, without incident [90].

Next, the transient organocopper species **D** was reacted with an assortment of heteroatom electrophiles. The thioether derivative **19** was obtained by reaction with dipyridyl disulfide in respectable yield, while the alkenyl iodide **25** resulted from quenching **D** with iodine. Drawing inspiration from work on the reactivity of organometallics with lithium nitrenoids [91], intermediate **D** was treated with BocN(Li)OAc (**27**) at −78°C; after warming to room temperature, enamide **26** was isolated as the major product. The analogous reaction with the lithium oxenoid *t*BuOOLi [92, 93, 94] furnished a mixture of ketone **21** and iodide **25**; to prevent the competing in situ generation of [I^+^] by the oxidant, the copper salt was changed from CuI to CuBr·SMe_2_, which resulted in the exclusive formation of acyloin **21** [95, 96, 97, 98]. In contrast to the other products described here, the silyl group was cleaved during work‐up of the crude reaction mixture. We posit that this outcome might be due to cyclization of the enolate intermediate primarily formed onto the silicon atom to generate the cyclic intermediate **20**, which is easily hydrolyzed.

The fact that the attempted reaction with the lithium oxenoid resulted in competing C─I bond formation rather than oxidative dimerization of the organocopper species [99] suggested that intermediates of this type might be amenable to stoichiometric Cu(I)/Cu(III) redox chemistry. To this end, [Cu(MeCN)_4_]BF_4_ was used as the copper source in order to preclude interference of the anion by reductive elimination. When the derived alkenylcopper species **D** was treated with *tert*‐butyl perbenzoate, the *tert*‐butyl enol ether **24** was obtained after silyl ether cleavage with K_2_CO_3_ in MeOH. Gratifyingly, the use of fluorocollidinium triflate as an electrophilic fluorinating agent gave alkenyl fluoride **22** in good yield [100, 101]. To our knowledge, this transformation (together with the complementary examples reported in the accompanying paper) constitutes the first example of a fully stereoretentive olefinic fluorodesilylation [102]; moreover, it represents a valuable alternative to the silver‐mediated fluorination of analogous alkenylstannanes, which tends to suffer from competing protodestannation [103, 104]. Finally, the reaction of **D** with Umemoto's reagent (**29**) furnished the trifluoromethylated alkene **23** in appreciable yield [105, 106, 107] which was best purified after deprotection. Except for **21**, the (EtO)_2_MeSi– ethers generated by the 1,3‐Brook rearrangement survived both aqueous work‐up and flash chromatography; if desirable, however, they can be easily cleaved using either aq. HCl (1 M) or K_2_CO_3_/MeOH as demonstrated with compounds **23** and **24**, respectively (for details, see the Supporting Information). Collectively, these experiments show that the proper choice of the copper source is critical for success.

## 3 Conclusion

In conclusion, we have developed a versatile set of conditions for the 1,3‐Brook rearrangement of α‐silylated allyl alcohols, which themselves are easy to make by ruthenium catalyzed *trans*‐hydrosilylation of propargyl alcohols. The alkenylcopper species thus formed under notably mild conditions were shown to undergo β‐heteroatom elimination to give allenes in a reaction that is catalytic in Cu(I), stereospecific, functional group tolerant, and high yielding. In stereochemical terms, the sequence is complementary to most of the preceding allene syntheses starting from propargylic alcohols. Alternatively, the alkenylcopper species can be trapped with a variety of carbon‐ and heteroatom‐based electrophiles to yield a multitude of trisubstituted alkenes in a regio‐ and stereodefined format as the result of a net *trans*‐hydrofunctionalization of propargylic alcohols. The accompanying paper in this issue reports the copper mediated functionalization of β‐silylated allylic alcohols via 1,4‐Brook rearrangement, which provides access to the regio‐complementary set of olefins carrying the newly introduced substituent distal to the propargylic alcohol [51]. Though conceptually related, the optimal reaction conditions, however, are notably different. This aspect aside, the new entry into trisubstituted alkene derivatives is arguably enabling and allows the potential of the ruthenium catalyzed *trans*‐hydrosilylation to be exploited to a previously inconceivable extent. For the mild conditions and the excellent overall selectivity profile, advanced applications of both the catalytic and the stoichiometric reaction manifolds seem promising; the first such case studies are underway in our laboratory.

Data that support the findings of this study can be found in the Supporting information section (Experimental procedures, characterization data, and copies of NMR spectra of new compounds).

## Author Contributions

**Jack L. Sutro**: conceptualization, investigation, writing – original draft, writing – review and editing, formal analysis, validation. **Felix Anderl**: conceptualization, investigation, writing – review and editing, formal analysis, validation. **Paul Haf‐Moths**: investigation, writing – review and editing, formal analysis, validation. **Alois Fürstner**: conceptualization, writing – original draft, writing – review and editing, validation, formal analysis, project administration, supervision, resources.

## Conflicts of Interest

The authors declare no conflicts of interest.

## Supporting information




**Supporting File**: anie74615‐sup‐0001‐SuppMat.pdf.

## Data Availability

The data that supports the findings of this study are available in the supplementary material of this article.
