# Supplementary material for: 1,3‐Brook Rearrangement of α‐Silyl Allylic Alcohols: Copper‐Catalyzed Enantiospecific Formation of Allenes and Copper‐Mediated ipso‐Substitution Reactions Affording Trisubstituted Alkenes
Source: Angew Chem Int Ed Engl. 2026 Sep 10;65(44):e5791239. doi: 10.1002/anie.5791239 (PMC13703936; doi:10.1002/anie.5791239)
Supplement: Supplementary file 1 [file ANIE-65-e5791239-s001.pdf]

SUPPORTING INFORMATION

1,3-Brook Rearrangement of α-Silyl Allylic Alcohols: Copper Catalyzed Enantiospecific Formation of Allenes and Copper Mediated *ipso*-Substitution Reactions Affording Trisubstituted Alkenes

Jack L. Sutro, Felix Anderl, Paul Haf-Moths, and Alois Fürstner*

Max-Planck-Institut für Kohlenforschung, 45470 Mülheim-an-der-Ruhr, Germany

Email: fuerstner@kofo.mpg.de

Table of Contents

General Information	S2
Procedures	S3
Characterization Data	S4
Weinreb Amides	S4
Acetylenic Ketones	S5
Propargylic Alcohols	S8
α-Silyl Allylic Alcohols	S29
Allenes	S34
Alkenylcopper Trapping: Functionalized Trisubstituted Allylic Alcohols	S53
NMR Spectra of New Compounds	S62
References	S123

General Information

Unless stated otherwise, all reactions were carried out in flame-dried glassware under an argon atmosphere using anhydrous solvents. The solvents were purified by distillation over the indicated drying agents and were transferred under an argon atmosphere: THF (magnesium/anthracene); diisopropylamine, dichloromethane (CaH_2); toluene (sodium tetraethylaluminate); MeOH, EtOH, *i*PrOH (Mg; stored over 3 Å MS). DMSO, DMF, NEt_3, and pyridine were dried by an adsorption solvent purification system based on molecular sieves.

Unless stated otherwise, all compounds were commercially available (Alfa Aesar, Sigma Aldrich, TCI, Strem Chemicals, ChemPUR) and used as received. 5 Å Molecular sieves were activated at 150 °C for 24 h under high vacuum (1×10^{-3} mbar), and stored and transferred under argon.

Thin layer chromatography (TLC): Macherey-Nagel precoated plates (POLYGRAM®SIL/UV254); Visualization by UV light (254 nm) or by staining with solutions of *p*-anisaldehyde or $KMnO_4$. Flash chromatography: Merck silica gel 60 (40-63 µm or 15-40 µm) with pre-distilled or HPLC grade solvents.

NMR: Spectra were recorded on Bruker AV 400, AVIII 600 or AVneo 600 spectrometers (equipped with a Bruker BBO cryoprobe) in the solvents indicated. Chemical shifts (δ) are given in ppm relative to tetramethylsilane (TMS) and coupling constants (*J*) in Hz. The (residual) solvent signals were used as references, and were themselves referenced to known values relative to TMS ($CDCl_3$: δ_C = 77.16 ppm; residual $CHCl_3$ in $CDCl_3$: δ_H = 7.26 ppm); all spectra were recorded at 25 °C. Multiplicities are reported as they appear in the spectrum, rather than as would be expected *a priori* (i.e. a resonance which would be predicted to be a double doublet but has two identical coupling constants is reported as a triplet). Peak multiplicities are indicated by the following abbreviations: s: singlet, d: doublet, t: triplet, q: quartet, p: pentet, h: hextet, hept: heptet, m: multiplet, dd: double doublet, dt: double triplet, and so on; "br" indicates a broad peak, where the width is large enough to eclipse small coupling constants which might otherwise be expected. ^{13}C NMR spectra were recorded with ^{1}H-decoupling, and the values of the chemical shifts are rounded to one decimal place. Two decimal places are given in cases where there are two or more distinct resonances which have the same value when rounded to one decimal place. Signal assignments were established using HSQC, HMBC, COSY, and NOESY.

IR: Spectra were recorded on an Alpha Platinum ATR instrument (Bruker); wavenumbers ($\tilde{\nu}$) are reported in cm^{-1}.

MS: Finnigan MAT 8200 (70 eV), ESQ3000 (Bruker), accurate mass determinations: Bruker APEX III FTMS (7 T magnet), Mat 95 (Finnigan), Q Exactive GC Orbitrap (Thermo Scientific).

Optical rotations ($[\alpha]_D^{20}$) were measured with an A-Krüss Optronic Model P8000-t polarimeter at a wavelength of 589 nm.

Procedures

General Procedure A: Weinreb Amides from Carboxylic Acids. A solution/suspension of the carboxylic acid (1.0 eq.) in dichloromethane (0.1 M in carboxylic acid) was cooled to 0 °C (bath temperature) in a flame-dried Schlenk flask. 1,1'-Carbonyl diimidazole (1.1 eq.) was added, and the resulting mixture was stirred for 30 min (***Note***: *in cases where the starting carboxylic acid was only partially soluble in dichloromethane, the reaction generally became a homogeneous solution during this period*). *N*,*O*-dimethyl hydroxylamine hydrochloride (2.2 eq.) was then added and the reaction was warmed to room temperature and stirred for a further 1 h, during the course of which a precipitate formed. The reaction mixture was filtered to remove the precipitated salts. The filtrate was washed with HCl (1 M) and brine, and then dried over anhydrous magnesium sulfate, and filtered. The filtrate was concentrated under reduced pressure, to give the crude product, which was in all cases found to be pure enough for direct use in the next step.

General Procedure B: Acetylenic Ketones from Weinreb Amides. A solution of the terminal alkyne (1.3 eq.) in THF (0.13 M in alkyne) was cooled to –78 °C (bath temperature) in a flame-dried Schlenk flask. *n*-Butyllithium (1.6 M in hexane, 1.2 eq.) was added dropwise, and the resulting solution was warmed to room temperature over a period of 30 min, after which it was cooled back to –78 °C. A solution of the Weinreb amide (1.0 eq.) in THF (1.0 M in Weinreb amide) was added dropwise, and the reaction was warmed to room temperature and stirred for 2 h. Saturated aqueous ammonium chloride was added slowly, and the reaction was diluted with ethyl acetate and water. The organic layer was separated, and the aqueous layer was extracted three times with ethyl acetate. The combined organic extracts were washed with brine, dried over anhydrous magnesium sulfate, and filtered. The filtrate was concentrated under reduced pressure, and the residue was purified *via* flash chromatography.

General Procedure C: Racemic Propargylic Alcohols from Acetylenic Ketones. A solution of the acetylenic ketone (1.0 eq.) and cerium trichloride heptahydrate (1.2 eq.) in methanol (0.1 M in ketone) was cooled to 0 °C (bath temperature). Sodium borohydride (1.2 eq.) was added in one portion, resulting in effervescence. The reaction was stirred for 5 min before being warmed to room temperature and quenched by the careful addition of saturated aqueous ammonium chloride solution. The reaction was diluted with ethyl acetate and water, the organic layer was separated, and the aqueous phase was extracted three times with ethyl acetate. The combined organic extracts were washed with brine, dried over anhydrous magnesium sulfate, and filtered. The filtrate was concentrated under reduced pressure to give the crude product, which was in all cases found to be pure enough for direct use.

General Procedure D: Asymmetric Synthesis of Propargylic Alcohols from Acetylenic Ketones. Pre-activated Ru [(*R*,*R*)-TsDPEN](mesitylene) (1 mol%)[1],[2] was added to a solution of the acetylenic ketone

(1.0 eq.) in isopropanol (0.1 M in ketone). The resulting light mauve coloured solution gradually evolved to yellow over the course of the reaction. After thin layer chromatography indicated complete consumption of the starting material, the solvent was removed under reduced pressure and the residue was purified *via* flash chromatography (0-20% EtOAc/cyclohexane).

General Procedure E: α-Silyl Allylic Alcohols from Propargylic Alcohols. $[Cp^*RuCl]_4$ (1 mol%) was added to a solution of the propargylic alcohol (1.0 eq.) in dichloromethane (0.1 M in alcohol), and the resulting deep violet solution was stirred under argon. Diethoxymethylsilane (1.1 eq.) was added dropwise, resulting in a dark green solution which was stirred for 30 min. The solvent was removed under reduced pressure and the residue was purified *via* flash chromatography (0-10% EtOAc/cyclohexane).

General Procedure F: Allenes from α-Silyl Allylic Alcohols. A solution of the allylic alcohol (1.0 eq.) in THF (0.2 M in alcohol) was cooled to 0 °C (bath temperature) in a flame-dried Schlenk flask. A solution of freshly prepared LDA (0.1 M in THF, 1.1-1.2 eq.) was added, followed by triethylamine (10% of the THF volume) and then CuI (0.2 eq.) (***Note***: *In cases where the CuI loading was too low to be measured accurately, a stock solution was prepared in 10:1 THF/triethylamine and used accordingly*). The mixture was stirred at 0 °C under argon until thin layer chromatography indicated complete consumption of the starting material. Saturated aqueous ammonium chloride solution was added, and the reaction was diluted with ethyl acetate and water. The organic layer was separated, and the aqueous phase was extracted three times with ethyl acetate. The combined organic extracts were washed with brine, dried over anhydrous magnesium sulfate, and filtered. The filtrate was concentrated under reduced pressure and the residue was purified *via* flash chromatography (0-5% *tert*-butyl methyl ether/cyclohexane).

The larger scale synthesis of compound (+)-**2** is outlined under the Section "Allenes".

Characterization Data

Weinreb Amides

Compound S1. Prepared according to the general procedure **A**; colourless oil (7.08 g, 99%). ^{1}H NMR (400 MHz, $CDCl_3$) δ 7.18 – 7.10 (m, 2H), 6.87 – 6.79 (m, 2H), 3.78 (s, 3H), 3.60 (s, 3H), 3.17 (s, 3H), 2.90 (br t, *J* = 7.8, 2H), 2.70 (br t, *J* = 7.9 Hz, 2H). ^{13}C NMR (101 MHz, $CDCl_3$) δ 173.9, 158.1, 133.5, 129.5, 114.0, 61.3, 55.4, 34.2, 32.3, 29.9. IR (film): $\tilde{\nu}$ 2937, 1659, 1612, 1512, 1462, 1443, 1416, 1384, 1300, 1244, 1178, 1111, 1034, 990, 825 cm^{-1}. HRMS (EI^+) calcd. for $C_{12}H_{17}NO_3$ $[M]^+$: 223.12029; found: 223.12009.

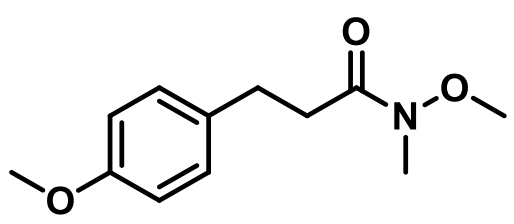

Compound S2. Prepared according to the general procedure **A**; colourless oil (536 mg, 87%). ^{1}H NMR

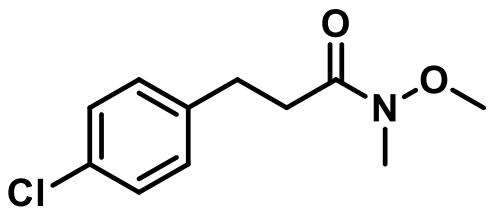

(400 MHz, $CDCl_3$) δ 7.26 – 7.21 (m, 2H), 7.18 – 7.13 (m, 2H), 3.61 (s, 3H), 3.17 (s, 3H), 2.92 (t, *J* = 7.8 Hz, 2H), 2.71 (t, *J* = 7.8 Hz, 2H). ^{13}C NMR (101 MHz, $CDCl_3$) δ 173.4, 139.9, 131.9, 130.0, 128.6, 61.3, 33.6, 32.3, 30.0. IR (film): $\tilde{\nu}$ 2937, 1657, 1492, 1441, 1410, 1384, 1338, 1177, 1091, 1015, 990, 815, 532, 496 cm^{-1}. HRMS (EI^+) calcd. for $C_{11}H_{14}NO_2Cl$ $[M]^+$: 227.07076; found: 227.07073.

Compound S3. Prepared according to the general procedure **A**; colourless oil (559 mg, 90%). ^{1}H NMR

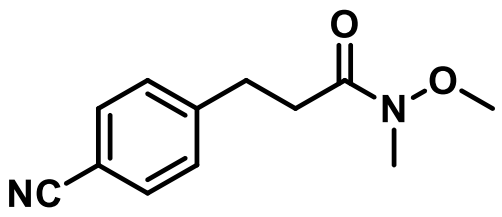

(400 MHz, $CDCl_3$) δ 7.68 – 7.49 (m, 2H), 7.40 – 7.29 (m, 2H), 3.62 (s, 3H), 3.16 (s, 3H), 3.01 (t, *J* = 7.6 Hz, 2H), 2.75 (t, *J* = 7.6 Hz, 2H). ^{13}C NMR (101 MHz, CDCl3) δ 172.9, 147.2, 132.4, 129.4, 119.1, 110.1, 61.4, 33.0, 32.3, 30.7. IR (film): $\tilde{\nu}$ 2938, 2227, 1657, 1607, 1505, 1416, 1386, 1341, 1178, 1113, 1021, 990, 827, 555, 433 cm^{-1}. HRMS (EI^+) calcd. for $C_{12}H_{14}N_2O_2$ $[M]^+$: 218.10498; found: 218.10462.

Acetylenic Ketones

Compound S4. Prepared according to the general procedure **B** from compound **S1** and 5-hexyne;

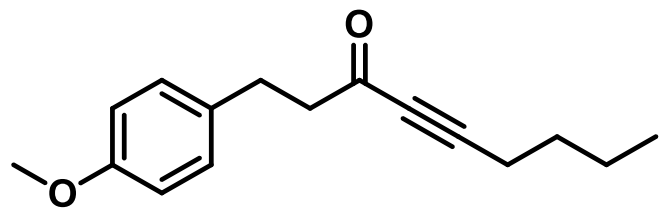

yellow oil (1.98 g, 74%). ^{1}H NMR (400 MHz, $CDCl_3$) δ 7.15 – 7.07 (m, 2H), 6.87 – 6.78 (m, 2H), 3.78 (s, 3H), 2.97 – 2.88 (m, 2H), 2.87 – 2.78 (m, 2H), 2.36 (t, *J* = 7.0 Hz, 2H), 1.58 – 1.51 (m, 2H), 1.48 – 1.37 (m, 2H), 0.93 (t, *J* = 7.3 Hz, 3H). ^{13}C NMR (101 MHz, $CDCl_3$) δ 187.5, 158.2, 132.5, 129.4, 114.0, 95.0, 81.0, 55.4, 47.4, 29.8, 29.3, 22.1, 18.8, 13.6. IR (film): $\tilde{\nu}$ 2957, 1932, 1871, 2213, 1671, 1612, 1512, 1464, 1360, 1323, 1300, 1245, 1178, 1160, 1106, 1035, 824 cm^{-1}. HRMS (EI^+) calcd. for $C_{16}H_{20}O_2$ $[M]^+$: 244.14578; found: 244.14574.

Compound S5. Prepared according to the general procedure **B** from compound **S2** and 5-hexyne;

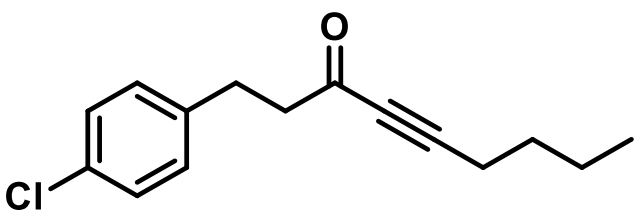

yellow oil (277 mg, 66%). ^{1}H NMR (400 MHz, $CDCl_3$) δ 7.26 – 7.22 (m, 2H), 7.16 – 7.09 (m, 2H), 2.99 – 2.89 (m, 2H), 2.89 – 2.79 (m, 2H), 2.36 (t, *J* = 7.0 Hz, 2H), 1.59 – 1.50 (m, 2H), 1.48 – 1.35 (m, 2H), 0.92 (t, *J* = 7.3 Hz, 3H). ^{13}C NMR (101 MHz, $CDCl_3$) δ 186.8, 139.0, 132.1, 129.8, 128.7, 95.3, 80.9, 46.8, 29.8, 29.4, 22.1, 18.8, 13.6. IR (film): $\tilde{\nu}$ 2958, 2932, 2871, 2213, 1671, 1492, 1465, 1407, 1357, 1325, 1288, 1245, 1205, 1160, 1092, 1015, 995, 977, 814, 771, 636, 509 cm^{-1}. HRMS (EI^+) calcd. for $C_{15}H_{17}OCl$ $[M]^+$: 248.09624; found: 248.09590.

Compound S6. Prepared according to the general procedure **B** from compound **S3** and 5-hexyne;

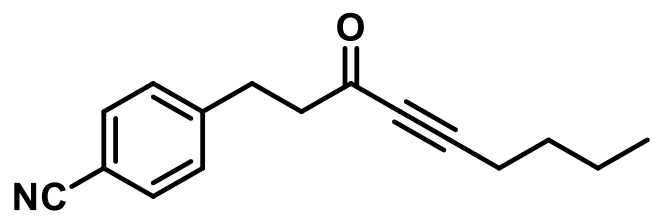

yellow oil (357 mg, 82%). ^{1}H NMR (400 MHz, $CDCl_3$) δ 7.61 – 7.52 (m, 2H), 7.35 – 7.27 (m, 2H), 3.02 (t, *J* = 7.5 Hz, 2H), 2.95 – 2.81 (m, 2H), 2.36 (t, *J*

= 7.1 Hz, 2H), 1.59 – 1.49 (m, 2H), 1.47 – 1.35 (m, 2H), 0.92 (t, *J* = 7.3 Hz, 3H). ^{13}C NMR (101 MHz, $CDCl_3$) δ 186.1, 146.2, 132.5, 129.4, 119.0, 110.4, 95.6, 80.8, 46.1, 30.0, 29.8, 22.1, 18.8, 13.6. IR (film): $\tilde{\nu}$ 2959, 2933, 2223, 2211, 1670, 1608, 1362, 1250, 1161, 826, 552 cm^{-1}. HRMS (EI$^+$) calcd. for $C_{16}H_{17}NO$ [M]$^+$: 239.13046; found: 239.13055.

Compound S7. Prepared according to the general procedure **B** from compound **S1** and 5-chloro-pent-

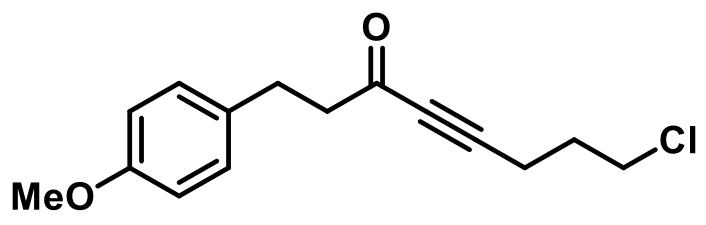

1-yne; yellow oil (437 mg, 74%). ^{1}H NMR (400 MHz, $CDCl_3$) δ 7.15 – 7.06 (m, 2H), 6.91 – 6.72 (m, 2H), 3.78 (s, 3H), 3.71 – 3.55 (m, 2H), 2.92 (ddd, *J* = 8.1, 6.8, 1.7 Hz, 2H), 2.84 (ddd, *J* = 8.7, 6.8, 1.7 Hz, 2H), 2.57 (t, *J* = 6.9 Hz, 2H), 2.02 (tt, *J* = 6.8, 6.1 Hz, 2H). ^{13}C NMR (101 MHz, $CDCl_3$) δ 187.1, 158.2, 132.4, 129.4, 114.1, 92.3, 81.5, 55.4, 47.4, 43.4, 30.5, 29.2, 16.5. IR (film): $\tilde{\nu}$ 2957, 2933, 2871, 2213, 1670, 1612, 1511, 1464, 1360, 1323, 1300, 1244, 1177, 1159, 1106, 1034, 996, 978, 823 cm^{-1}. HRMS (EI$^+$) calcd. for $C_{15}H_{17}O_2Cl$ [M]$^+$: 264.09116; found: 264.09118.

Compound S8. Prepared according to the general procedure **B** from compound **S1** and the *tert*-

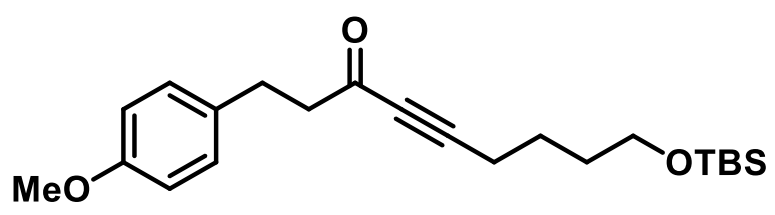

butyl(hex-5-yn-1-yloxy)dimethylsilane; yellow oil (2.82 g, 86%). ^{1}H NMR (400 MHz, $CDCl_3$) δ 7.15 – 7.06 (m, 2H), 6.92 – 6.70 (m, 2H), 3.78 (s, 3H), 3.63 (t, *J* = 5.9 Hz, 2H), 2.92 (ddd, *J* = 9.1, 7.2, 2.0 Hz, 2H), 2.87 – 2.78 (m, 2H), 2.44 – 2.36 (m, 2H), 1.79 – 1.60 (m, 4H), 0.89 (s, 9H), 0.04 (s, 6H). ^{13}C NMR (101 MHz, $CDCl_3$) δ 187.4, 158.2, 132.5, 129.4, 114.1, 94.7, 81.1, 62.5, 55.4, 47.4, 31.9, 29.3, 26.1, 24.5, 18.9, 18.5, –5.2. IR (film): $\tilde{\nu}$ 2952, 2930, 2857, 2214, 1674, 1612, 1513, 1464, 1360, 1300, 1247, 1178, 1158, 1105, 1037, 1066, 975, 835, 776 cm^{-1}. HRMS (ESI$^+$) calcd. for $C_{22}H_{34}O_3SiNa$ [M+Na]$^+$: 397.21694; found: 397.21722.

Compound S9. Compound **S8** (600 mg, 1.601 mmol) was dissolved in 1:1 acetone/water (16 mL), and

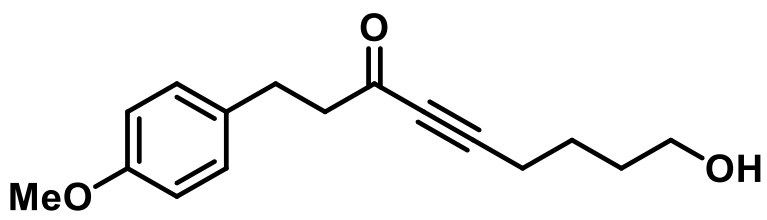

the resulting solution was cooled to 0 °C in an ice bath. *para*-Toluenesulfonic acid monohydrate (30.2 mg, 0.160 mmol) was added, and the mixture was stirred for 12 h. Saturated aqueous sodium bicarbonate solution (5 mL) was added, and the mixture was diluted with water and ethyl acetate. The organic phase was separated, and the aqueous layer was extracted three times with ethyl acetate. The combined organic extracts were washed with brine, dried over anhydrous magnesium sulfate, and filtered. The volatile organics were removed under reduced pressure to give the crude product as a pale yellow oil which was found to be pure enough for direct use in the next step (390 mg, 94%). ^{1}H NMR (400 MHz, $CDCl_3$) δ 7.15 – 7.06 (m, 2H), 6.90 – 6.75 (m, 2H), 3.78 (s, 3H), 3.68 (q, *J* = 3.5 Hz, 2H), 2.92 (ddd, *J* = 8.5, 7.0, 1.8 Hz, 2H), 2.83 (ddd, *J* = 8.5, 7.0, 1.7 Hz, 2H), 2.52 – 2.35 (m, 2H), 1.77 – 1.59 (m, 4H). ^{13}C NMR (101 MHz, CDCl3) δ 187.4, 158.2, 132.5, 129.4, 114.1, 94.3, 81.2, 62.3, 55.4, 47.4, 31.8, 29.3, 24.2, 18.9. IR (film): $\tilde{\nu}$ 3049, 2933, 2868, 2836, 2211, 1668, 1612, 1512, 1455,

1404, 1362, 1325, 1300, 1245, 1178, 1161, 1106, 1060, 1034, 825 cm^{-1}. HRMS (EI^+) calcd. for $C_{16}H_{20}O_3$ $[M]^+$: 260.14070; found: 260.14089.

Compound S10. Compound **S9** (250 mg, 0.960 mmol) was dissolved in dichloromethane (10 mL), and

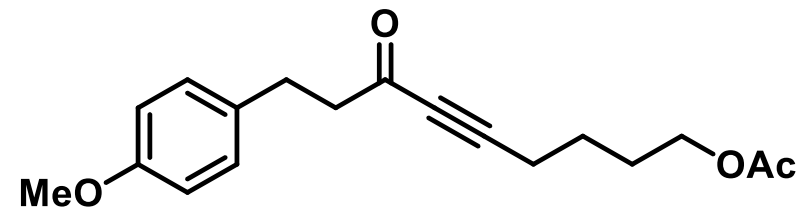

the resulting solution was cooled to 0 °C in an ice bath. Triethylamine (0.270 mL, 1.93 mmol) was added, followed by acetic anhydride (0.140 mL, 1.48 mmol). The resulting solution was warmed to room temperature and stirred for 24 h. Saturated aqueous sodium bicarbonate solution (5 mL) was added, and the mixture was diluted with water and ethyl acetate. The organic phase was separated, and the aqueous layer was extracted three times with ethyl acetate. The combined organic extracts were washed with brine, dried over anhydrous magnesium sulfate, and filtered. The volatile organics were removed under reduced pressure and the residue was purified *via* flash chromatography (eluting with 0→25% ethyl acetate/cyclohexane) to give the title compound as a pale yellow oil (256 mg, 90%). ^{1}H NMR (400 MHz, $CDCl_3$) δ 7.17 – 7.05 (m, 2H), 6.86 – 6.77 (m, 2H), 4.08 (t, *J* = 6.3 Hz, 2H), 3.78 (s, 3H), 2.92 (ddd, *J* = 8.3, 6.9, 1.7 Hz, 2H), 2.83 (ddd, *J* = 8.6, 6.9, 1.7 Hz, 2H), 2.41 (t, *J* = 6.9 Hz, 2H), 2.05 (s, 3H), 1.80 – 1.69 (m, 2H), 1.69 – 1.61 (m, 2H). ^{13}C NMR (101 MHz, $CDCl_3$) δ 187.3, 171.2, 158.2, 132.5, 129.4, 114.1, 93.7, 81.3, 63.8, 55.4, 47.4, 29.2, 27.9, 24.4, 18.8. IR (film): $\tilde{\nu}$ 2954, 2213, 1735, 1669, 1612, 1512, 1455, 1387, 1364, 1299, 1235, 1177, 1158, 1106, 1033, 824 cm^{-1}. HRMS (EI^+) calcd. for $C_{18}H_{22}O_4$ $[M]^+$: 302.15126; found: 302.15155.

Compound S11. Prepared according to the general procedure **B** from compound **S1** and cyclopentyl-

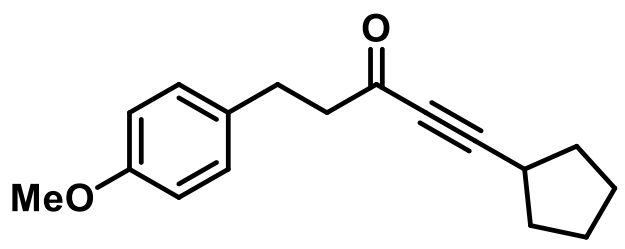

acetylene; yellow oil (530 mg, 92%). ^{1}H NMR (400 MHz, $CDCl_3$) δ 7.18 – 7.00 (m, 2H), 6.89 – 6.70 (m, 2H), 3.78 (s, 3H), 2.94 – 2.88 (m, 2H), 2.89 – 2.70 (m, 3H), 2.06 – 1.92 (m, 2H), 1.81 – 1.52 (m, 6H). ^{13}C NMR (101 MHz, $CDCl_3$) δ 187.6, 158.2, 132.6, 129.4, 114.0, 99.1, 80.5, 55.4, 47.4, 33.4, 30.2, 29.3, 25.3. IR (film): $\tilde{\nu}$ 2956, 2870, 2203, 1667, 1611, 1511, 1451, 1299, 1243, 1177, 1130, 1034, 982, 824 cm^{-1}. HRMS (EI^+) calcd. for $C_{17}H_{20}O_2$ $[M]^+$: 256.14578; found: 256.14574.

Compound S12. Prepared according to the general procedure **B** from compound **S1** and cyclohexenyl-

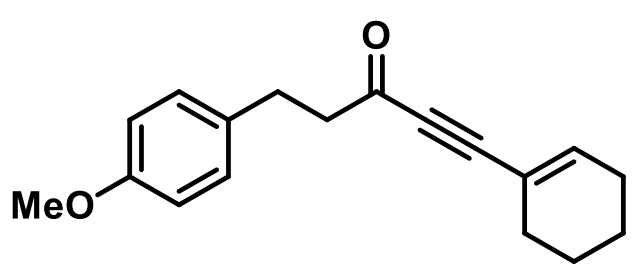

acetylene; yellow oil (366 mg, 61%). ^{1}H NMR (400 MHz, $CDCl_3$) δ 7.19 – 6.99 (m, 2H), 6.94 – 6.74 (m, 2H), 6.45 (dq, *J* = 4.1, 1.8 Hz, 1H), 3.78 (s, 3H), 2.94 (ddd, *J* = 8.2, 6.7, 1.7 Hz, 2H), 2.86 (ddd, *J* = 8.7, 6.8, 1.7 Hz, 2H), 2.21 – 2.08 (m, 4H), 1.73 – 1.59 (m, 4H). ^{13}C NMR (101 MHz, $CDCl_3$) δ 187.4, 158.2, 142.7, 132.6, 129.4, 119.1, 114.1, 93.9, 86.3, 55.4, 47.4, 29.4, 28.5, 26.3, 22.1, 21.2. IR (film): $\tilde{\nu}$ 2932, 2860, 2835, 2183, 1666, 1613, 1512, 1439, 1361, 1299, 1245, 1211, 1178, 1082, 1035, 964, 920, 824 cm^{-1}. HRMS (ESI^+) calcd. for $C_{18}H_{20}O_2$ $[M]^+$: 268.14578; found: 268.14595.

Compound S13. Prepared according to the general procedure **B** from compound **S1** and phenylacetylene; yellow oil (4.16 g, 95% yield).

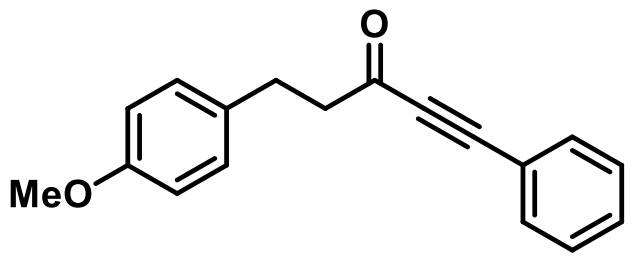

^{1}H NMR (400 MHz, $CDCl_3$) δ 7.58 – 7.55 (m, 2H), 7.46 (ddt, *J* = 8.4, 6.6, 1.4 Hz, 1H), 7.41 – 7.36 (m, 2H), 7.17 – 7.13 (m, 2H), 6.86 – 6.83 (m, 2H), 3.79 (s, 3H), 3.04 – 2.95 (m, 4H). ^{13}C NMR (101 MHz, $CDCl_3$) δ 187.2, 158.2, 133.2, 132.4, 130.9, 129.4, 128.8, 120.1, 114.1, 91.2, 87.9, 55.4, 47.4, 29.3. IR (neat): ν 2933, 2200, 1665, 1611, 1584, 1511, 1489, 1463, 1442, 1402, 1360, 1298, 1243, 1177, 1086, 1033, 997, 920, 822, 756, 688, 603, 578, 535, 514 cm^{-1}. HRMS (ESI$^+$) calcd. for $C_{18}H_{16}O_2Na$ $[M+Na]^+$: 264.11448; found: 264.11426.

Propargylic Alcohols

Compound 4. Prepared according to the general procedure **D** from compound **S4**; pale yellow oil (779 mg, 97%).

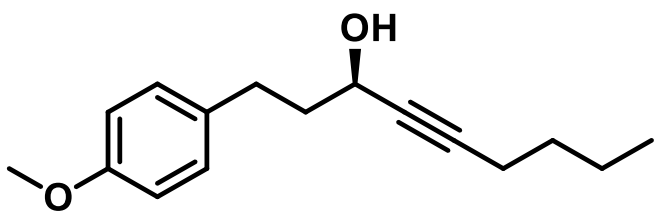

The e.e. was found to be 98.1% (see below for the HPLC data using a chiral stationary phase). $[\alpha]_D^{20}$= –38.4 (c = 0.52, $CHCl_3$). ^{1}H NMR (400 MHz, $CDCl_3$) δ 7.19 – 7.08 (m, 2H), 6.90 – 6.76 (m, 2H), 4.48 – 4.24 (m, 1H), 3.78 (s, 3H), 2.73 (t, *J* = 7.8 Hz, 2H), 2.23 (td, *J* = 7.0, 2.0 Hz, 2H), 2.04 – 1.87 (m, 2H), 1.80 (d, *J* = 5.2 Hz, 1H), 1.57 – 1.46 (m, 2H), 1.46 – 1.36 (m, 2H), 0.92 (t, *J* = 7.2 Hz, 3H). ^{13}C NMR (101 MHz, $CDCl_3$) δ 158.0, 133.7, 129.5, 114.0, 86.1, 81.1, 62.2, 55.4, 40.0, 30.9, 30.7, 22.1, 18.5, 13.7. IR (neat): $\tilde{\nu}$ 3383, 2955, 2931, 2861, 2835, 1611, 1584, 1511, 1465, 1379, 1327, 1300, 1243, 1176, 1106, 1035, 905, 822, 807, 746, 704, 566, 522 cm^{-1}. HRMS (ESI$^+$) calcd. for $C_{16}H_{22}O_2Na$ $[M+Na]^+$: 269.15120; found: 269.15149.

Compound *rac*-4. Prepared according to the general procedure **C** from compound **S4**; colourless oil (831 mg, 92%). NMR, MS, and IR data were found to be identical to those obtained for the enantioenriched compound.

SUJ-SA-330 Racemat
150 mm Chiralpak IC-3, 3 µm, 4.6 mm i.D.
n-Heptan / 2-Propanol = 90:10
1.0 ml/min / 8.1 MPa / 298 K

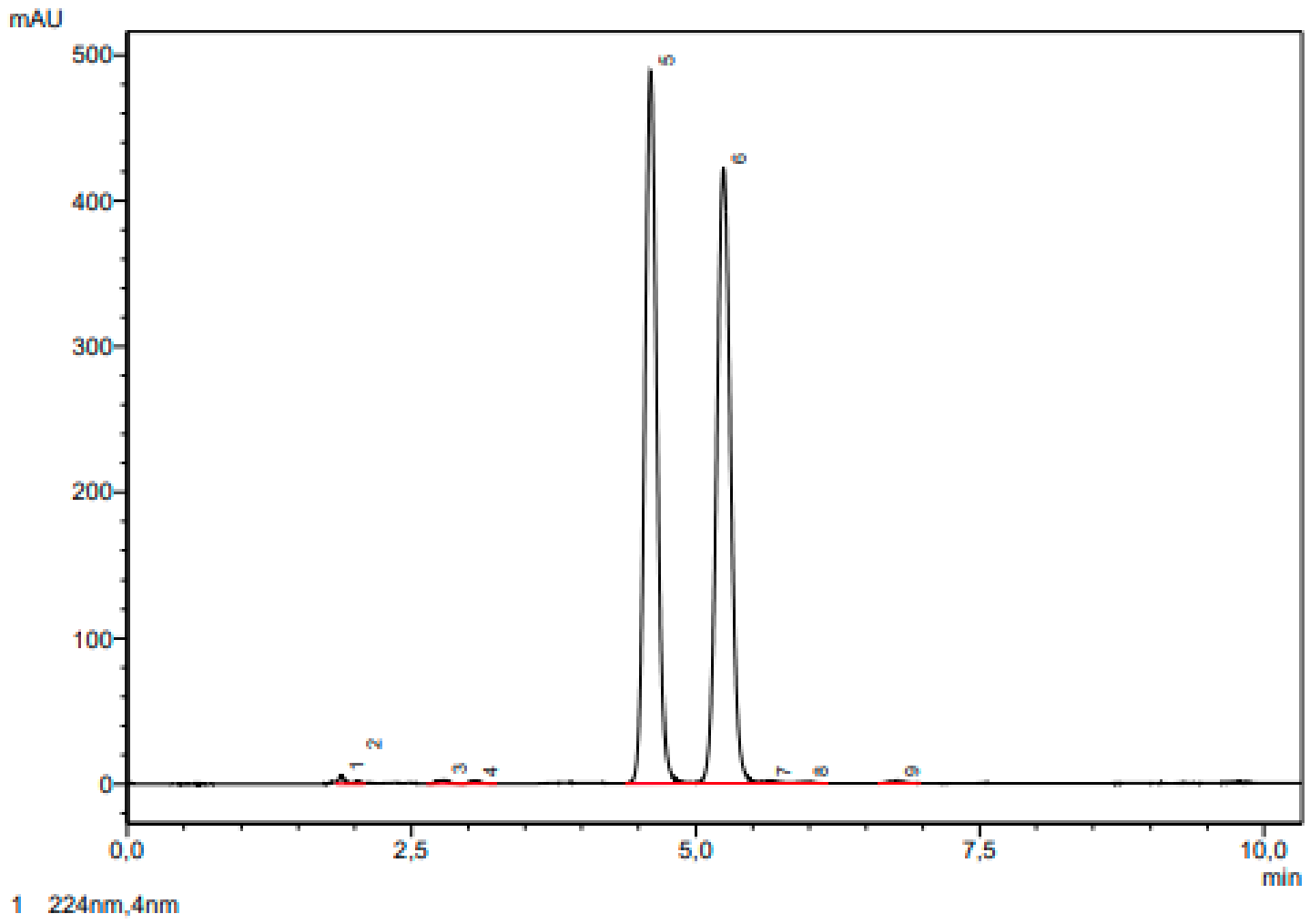

PDA Ch1 224nm

Peak #	Ret. Time	Area %	Name
1	1,88	0,28	
2	2,03	0,12	
3	2,79	0,27	
4	3,06	0,18	
5	4,61	49,07	1. Enantiomer
6	5,24	49,46	2. Enantiomer
7	5,64	0,28	
8	5,96	0,14	
9	6,76	0,21	
Total		100,00	

SUJ-SA-558-01
150 mm Chiralpak IC-3, 3 µm, 4.6 mm i.D.
n-Heptan / 2-Propanol = 90:10
1.0 ml/min / 8.1 MPa / 298 K

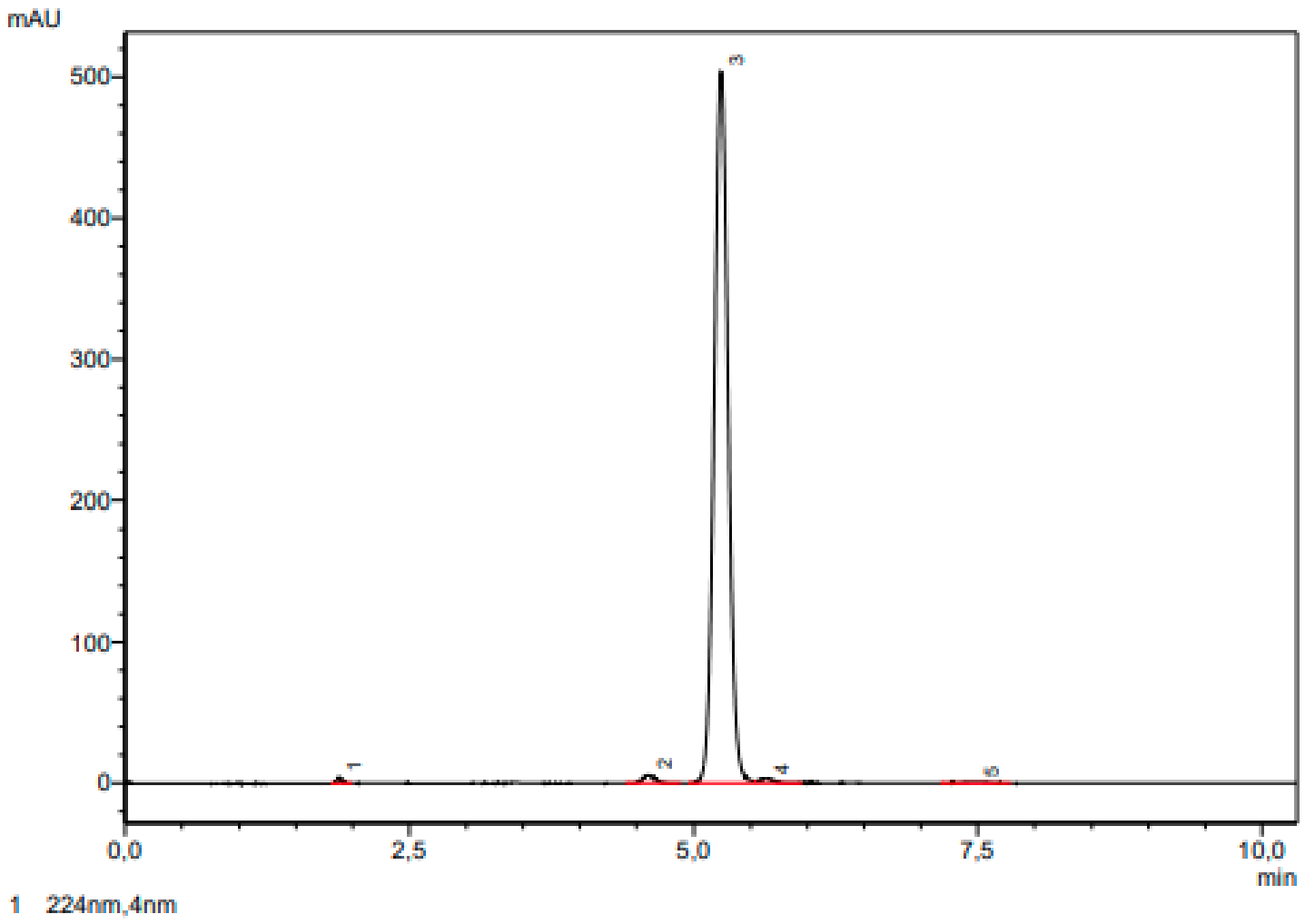

PDA Ch1 224nm

Peak #	Ret. Time	Area %	Name
1	1,87	0,27	
2	4,61	0,94	1. Enantiomer
3	5,24	97,85	2. Enantiomer 98.1 % ee
4	5,64	0,66	
5	7,48	0,28	
Total		100,00	

Compound S14. Prepared according to the general procedure **D** from compound **S5**; pale yellow oil

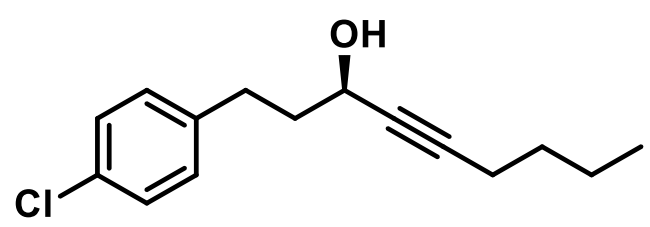

(55 mg, 27%, 55% brsm). The e.e. was found to be 97.6% (see below for the HPLC data using a chiral stationary phase). $[\alpha]_D^{20}$= −20.0 (c = 0.35, $CHCl_3$). ^{1}H NMR (400 MHz, $CDCl_3$) δ 7.26 – 7.22 (m, 2H), 7.16 – 7.11 (m, 2H), 4.34 (tt, *J* = 6.6, 2.1 Hz, 1H), 2.75 (t, *J* = 7.8 Hz, 2H), 2.22 (td, *J* = 7.0, 2.0 Hz, 2H), 2.02 – 1.86 (m, 2H), 1.73 (s, 1H), 1.61 – 1.45 (m, 2H), 1.45 – 1.34 (m, 2H), 0.92 (t, *J* = 7.2 Hz, 3H). ^{13}C NMR (101 MHz, $CDCl_3$) δ 140.1, 131.8, 130.0, 128.6, 86.3, 80.9, 62.0, 39.6, 31.0, 30.8, 22.1, 18.5, 13.7. IR (film): $\tilde{\nu}$ 3309 (br), 2956, 2931, 2862, 1491, 1455, 1431, 1407, 1379, 1093, 1058, 1032, 1015, 816 cm^{-1}. HRMS (EI$^+$) calcd. for $C_{15}H_{19}OCl$ $[M]^+$: 250.11189; found: 250.11193.

Compound *rac*-S14. Prepared according to the general procedure **C** from compound **S5**; pale yellow oil (100 mg, quant.). NMR, MS, and IR data were found to be identical to those obtained for the enantioenriched compound.

SUJ-SA-436-01 Racemat
150 mm Chiralpak IB-N-3, 3 µm, 4.6 mm i.D.
n-Heptan / 2-Propanol = 98:2
1.0 ml/min / 8.4 MPa / 298 K

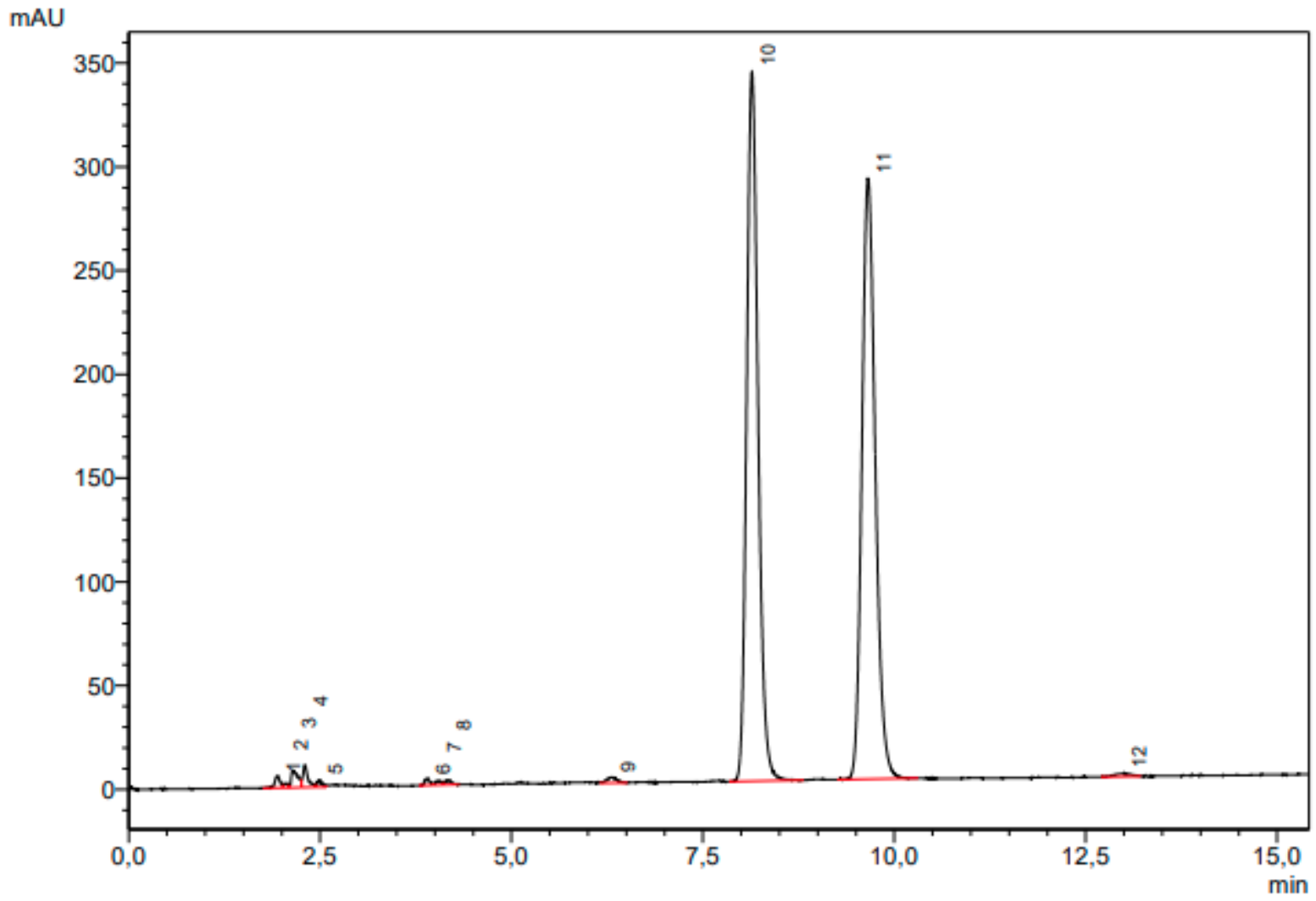

1 220nm,4nm

PDA Ch1 220nm

Peak #	Ret. Time	Area %	Name
1	1,94	0,38	
2	2,04	0,10	
3	2,14	0,72	
4	2,30	0,68	
5	2,49	0,22	
6	3,90	0,21	
7	4,04	0,16	
8	4,17	0,19	
9	6,31	0,37	
10	8,14	48,38	1. Enantiomer
11	9,66	48,27	2. Enantiomer
12	12,99	0,31	
Total		100,00	

SUJ-SA-440-01
150 mm Chiralpak IB-N-3, 3 µm, 4.6 mm i.D.
n-Heptan / 2-Propanol = 98:2
1.0 ml/min / 8.4 MPa / 298 K

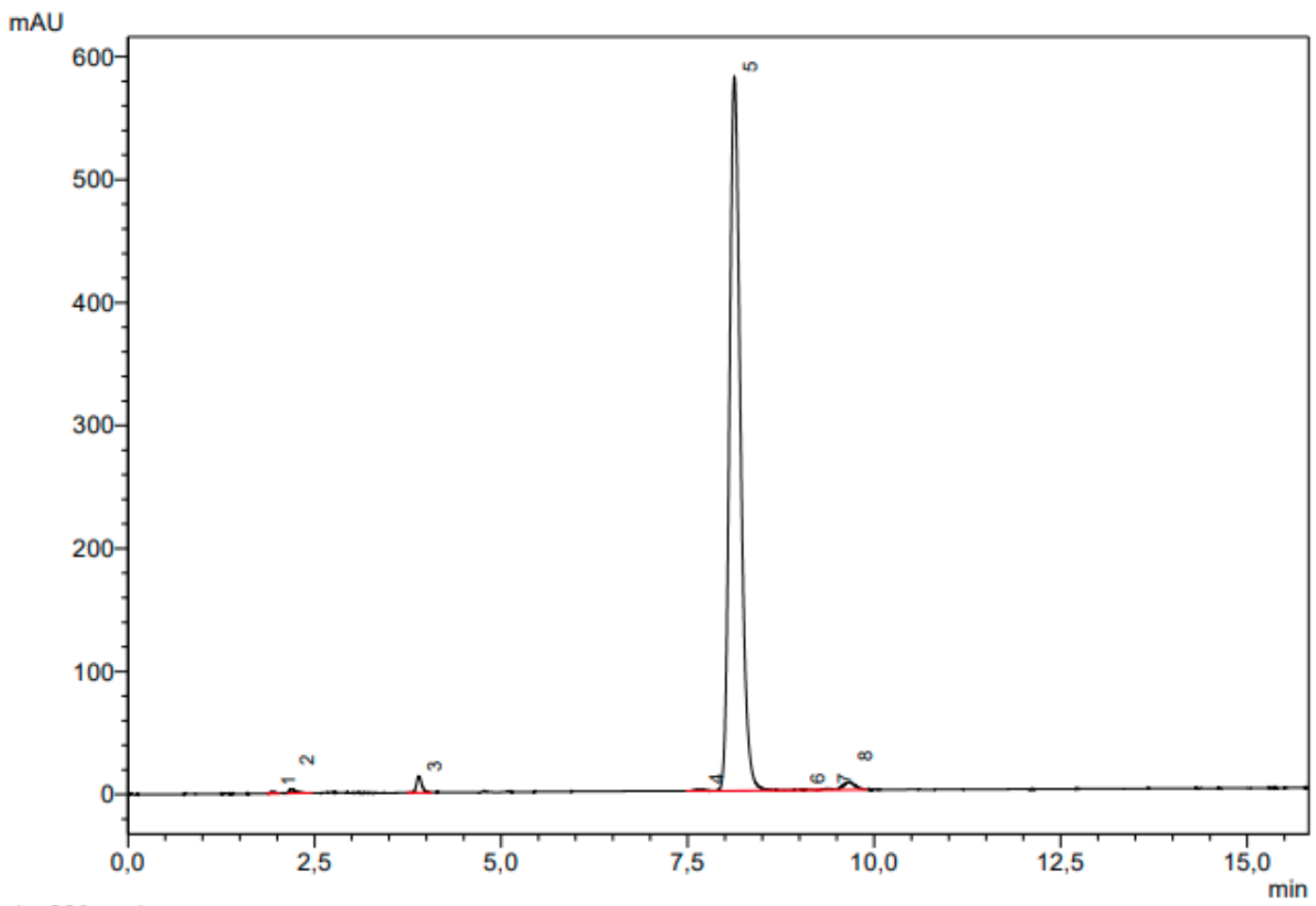

1 220nm,4nm

PDA Ch1 220nm

Peak #	Ret. Time	Area %	Name
1	1,93	0,14	
2	2,18	0,33	
3	3,90	1,10	
4	7,67	0,21	
5	8,13	96,70	1. Enantiomer 97.6 % ee
6	9,03	0,16	
7	9,39	0,18	
8	9,66	1,19	2. Enantiomer
Total		100,00	

Compound S15. Prepared according to the general procedure **D** from compound **S6**; pale yellow oil

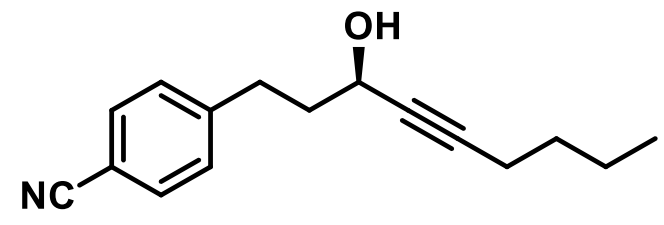

(101 mg, 50%). The e.e. was found to be 98.1% (see below for the HPLC data using a chiral stationary phase; enantioenriched sample contaminated by a solvent impurity). $[\alpha]_D^{20}$= −9.5 (c = 0.39, $CHCl_3$). ^{1}H NMR (400 MHz, $CDCl_3$) δ 7.65 – 7.53 (m, 2H), 7.36 – 7.28 (m, 2H), 4.34 (d, *J* = 6.8 Hz, 1H), 2.90 – 2.74 (m, 2H), 2.22 (td, *J* = 7.0, 2.0 Hz, 2H), 2.03 – 1.90 (m, 2H), 1.78 (d, *J* = 3.9 Hz, 1H), 1.54 – 1.44 (m, 2H), 1.44 – 1.33 (m, 2H), 0.92 (t, *J* = 7.2 Hz, 3H). ^{13}C NMR (101 MHz, $CDCl_3$) δ 147.5, 132.4, 129.5, 119.2, 110.0, 86.6, 80.7, 61.9, 39.1, 31.7, 30.8, 22.1, 18.5, 13.7. IR (film): $\tilde{\nu}$ 3410 (br) 3239 (br), 2956, 2932, 2870, 2228, 1607, 1505, 1456, 1432, 1414, 1381, 1177, 1058, 1020, 845, 825, 554 cm^{-1}. HRMS (EI$^+$) calcd. for $C_{16}H_{19}NO$ $[M]^+$: 241.14611; found: 241.14604.

Compound *rac*-S15. Prepared according to the general procedure **C** from compound **S6**; pale yellow oil (100 mg, quant.). NMR, MS, and IR data were found to be identical to those obtained for the enantioenriched compound.

SUJ-SA-437-01 Racemat
150 mm Chiralpak IB-N-3, 3 µm, 4.6 mm i.D.
n-Heptan / 2-Propanol = 98:2
1.0 ml/min / 8.4 MPa / 298 K

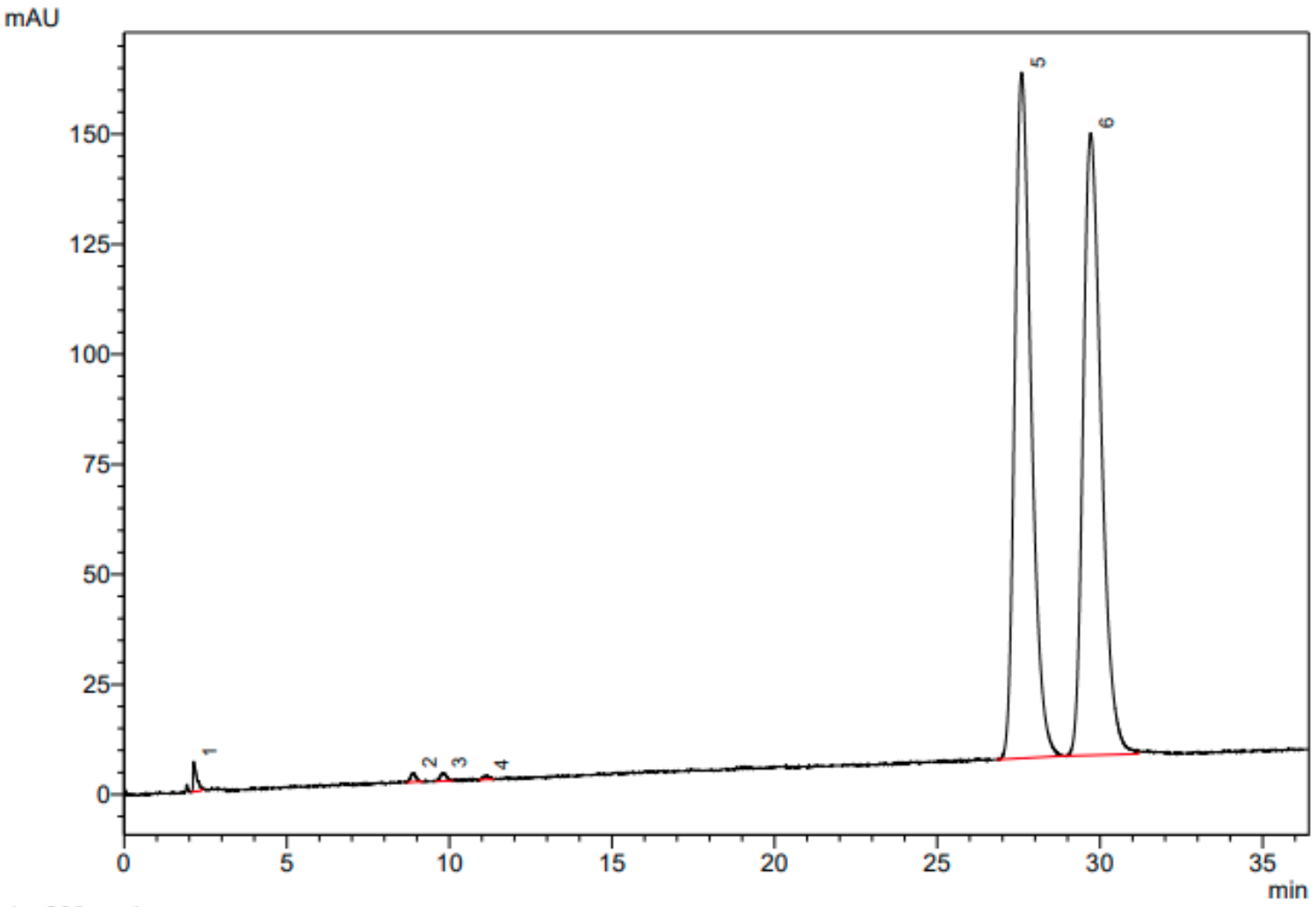

1 230nm,4nm

PDA Ch1 230nm

Peak #	Ret. Time	Area %	Name
1	2,14	0,48	
2	8,89	0,21	
3	9,81	0,22	
4	11,11	0,10	
5	27,59	49,51	1. Enantiomer
6	29,71	49,48	2. Enantiomer
Total		100,00	

SUJ-SA-441-01
150 mm Chiralpak IB-N-3, 3 µm, 4.6 mm i.D.
n-Heptan / 2-Propanol = 98:2
1.0 ml/min / 8.4 MPa / 298 K

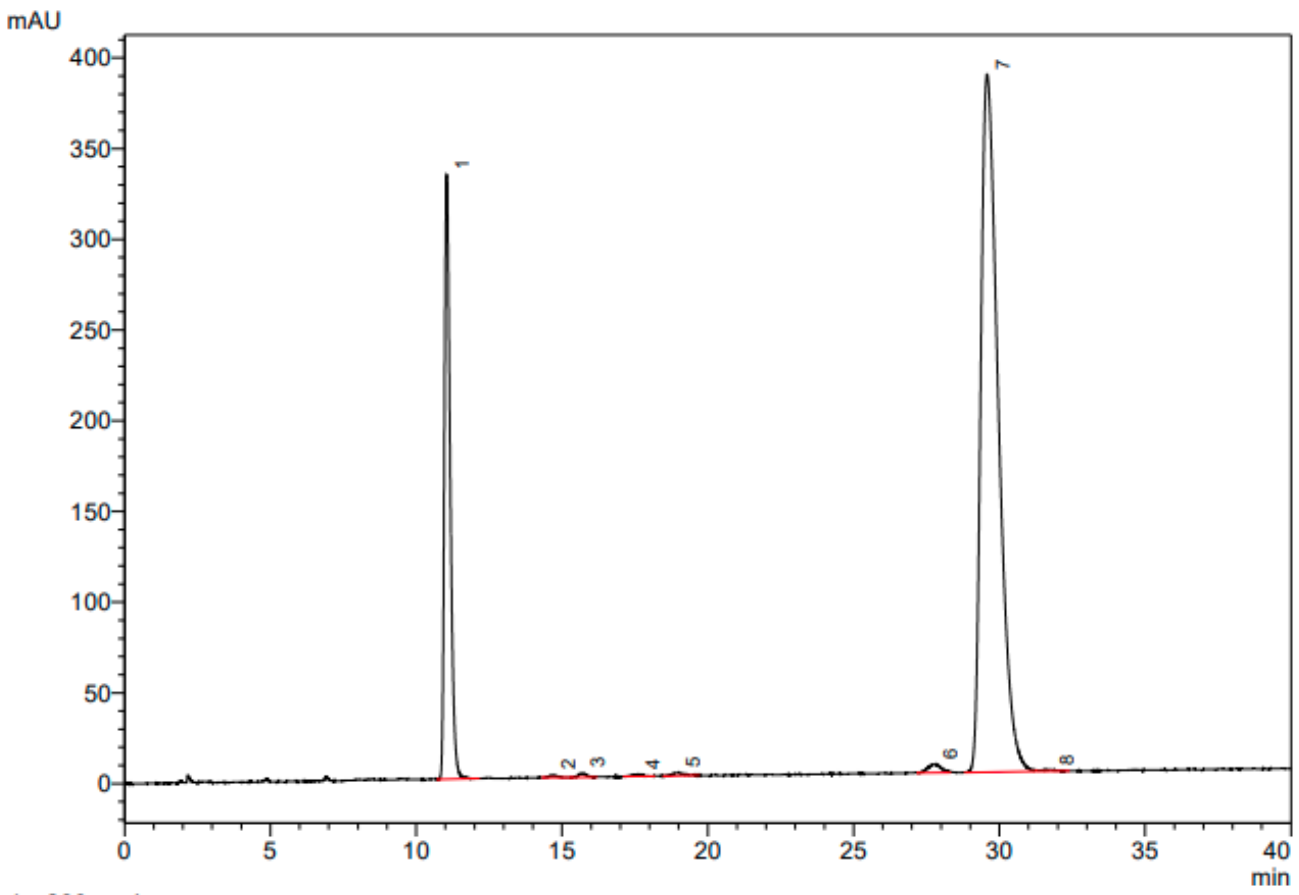

1 230nm,4nm

PDA Ch1 230nm

Peak #	Ret. Time	Area %	Name
1	11,05	22,03	
2	14,68	0,11	
3	15,69	0,19	
4	17,57	0,12	
5	18,95	0,20	
6	27,77	0,73	1. Enantiomer
7	29,58	76,47	2. Enantiomer 98.1 % ee
8	31,77	0,15	
Total		100,00	

Compound S16. Prepared according to the general procedure **D** from compound **S7**; pale yellow oil

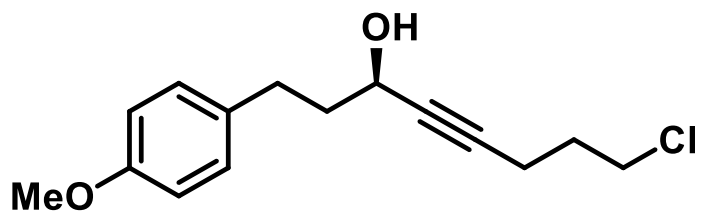

(97.0 mg, 97%). The e.e. was found to be 95.5% (see below for the HPLC data using a chiral stationary phase). $[\alpha]_D^{20}$ = –26.9 (c = 0.39, $CHCl_3$). ^1H NMR (400 MHz, $CDCl_3$) δ 7.17 – 7.08 (m, 2H), 6.88 – 6.79 (m, 2H), 4.35 (dtt, *J* = 8.5, 6.7, 2.0 Hz, 1H), 3.79 (s, 3H), 3.65 (t, *J* = 6.3 Hz, 2H), 2.72 (t, *J* = 7.8 Hz, 2H), 2.43 (td, *J* = 6.8, 2.0 Hz, 2H), 2.05 – 1.84 (m, 4H), 1.76 (d, *J* = 5.5 Hz, 1H). ^{13}C NMR (101 MHz, $CDCl_3$) δ 158.0, 133.5, 129.5, 114.0, 83.9, 82.3, 62.1, 55.4, 43.7, 39.9, 31.4, 30.7, 16.3. IR (film): $\tilde{\nu}$ 3376, 2934, 1612, 1512, 1455, 1441, 1299, 1245, 1178, 1059, 1036, 826 cm^{-1}. HRMS (EI^+) calcd. for $C_{15}H_{19}O_2Cl$ $[M]^+$: 266.10681; found: 266.10683.

Compound *rac*-S16. Prepared according to the general procedure **C** from compound **S7**; pale yellow oil (207 mg, 97%). NMR, MS, and IR data were found to be identical to those obtained for the enantioenriched compound.

SEY-SA-116-01 Racemat
150 mm Chiralpak IG-3, 3 µm, 4.6 mm i.D.
Acetonitril / Wasser = 60:40
1.0 ml/min, 12.7 MPa, 298 K

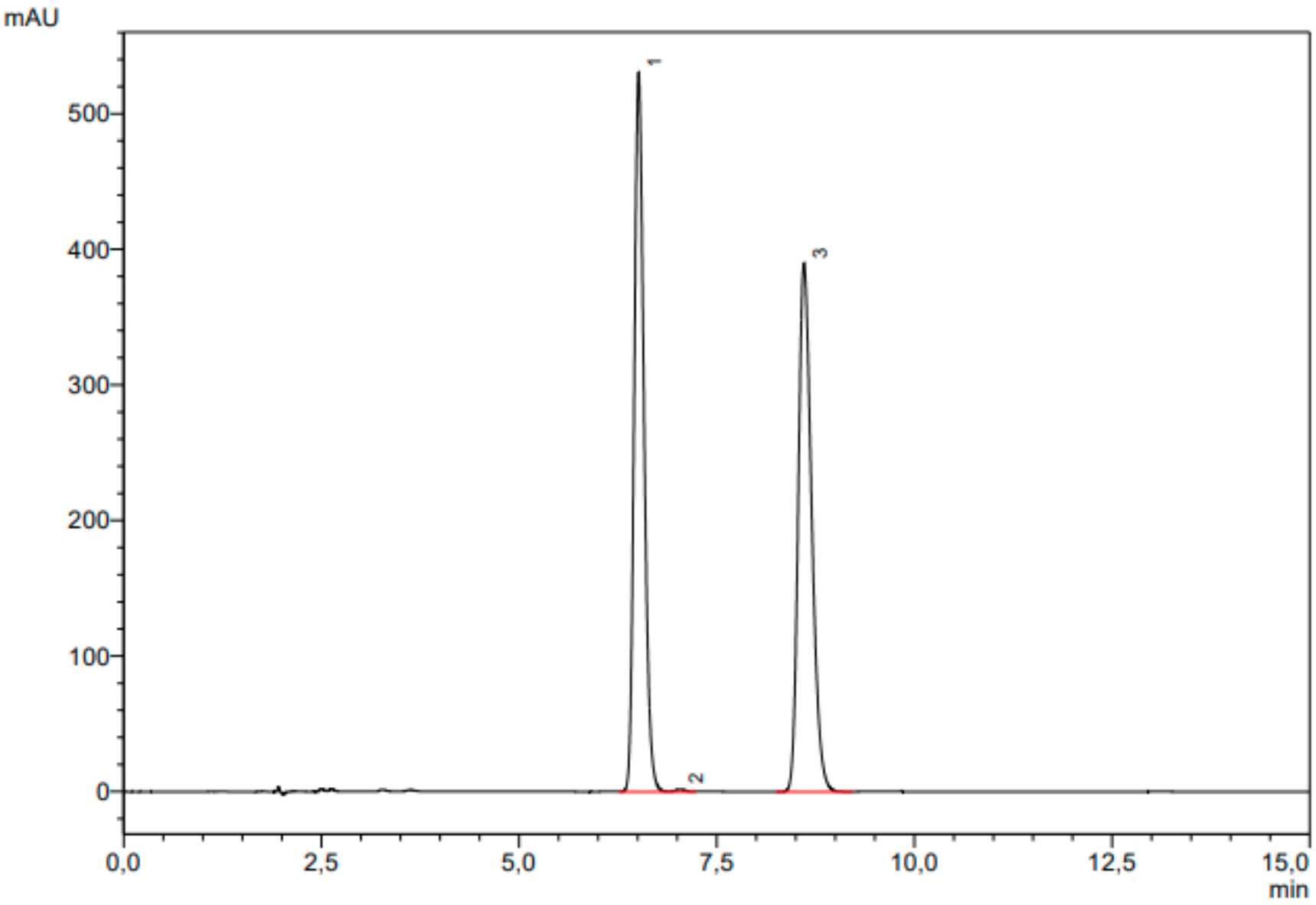

1 225nm,4nm

PDA Ch1 225nm

Peak #	Ret. Time	Area %		Name
1	6,51	49,84	1. Enantiomer	
2	7,04	0,16		
3	8,61	49,99	2. Enantiomer	
Total		100,00		

SUJ-SA-541-01
150 mm Chiralpak IG-3, 3 µm, 4.6 mm i.D.
Acetonitril / Wasser = 60:40
1.0 ml/min, 12.7 MPa, 298 K

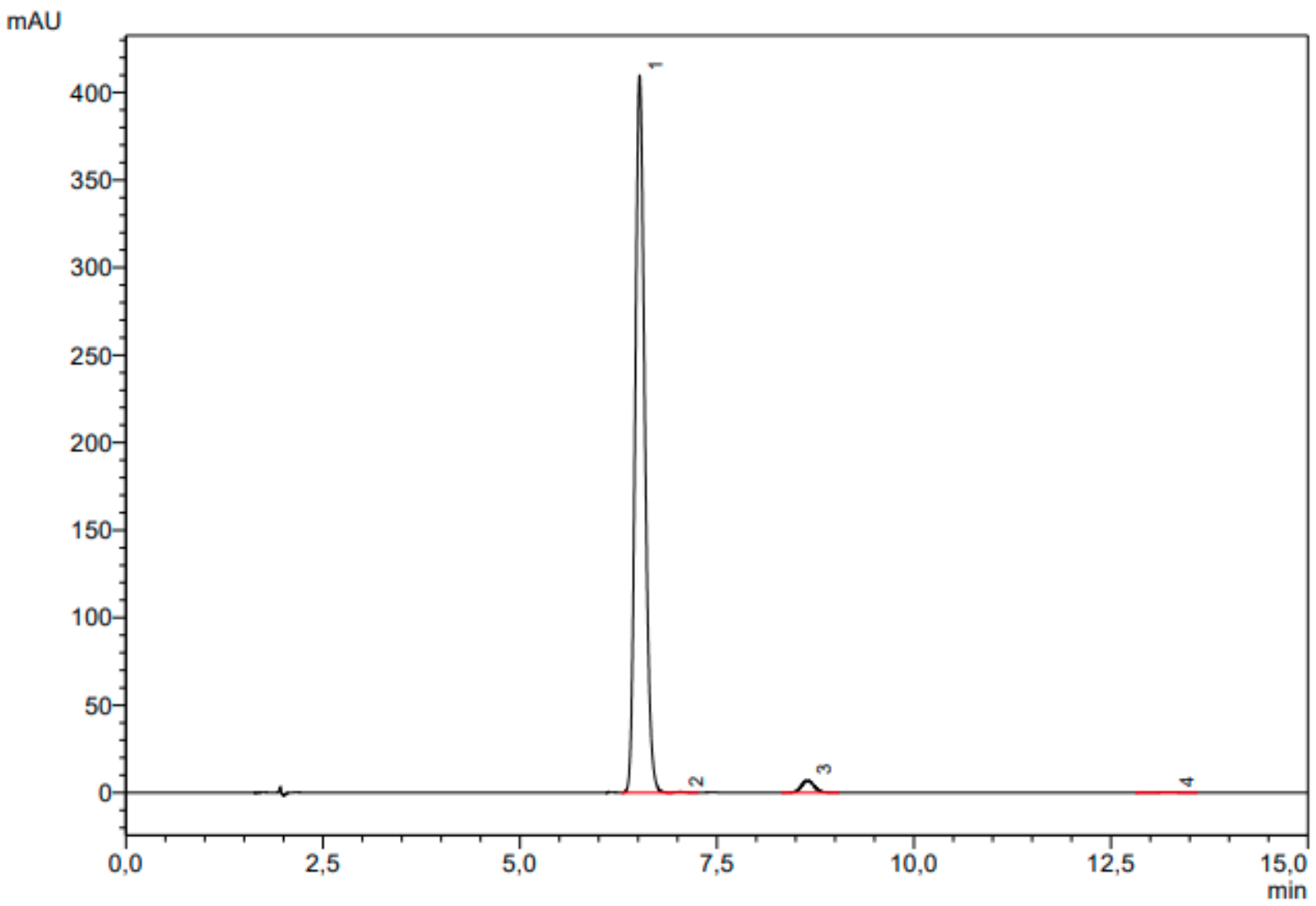

1 225nm,4nm

PDA Ch1 225nm

Peak #	Ret. Time	Area %		Name
1	6,52	97,49	1. Enantiomer 95.5 % ee	
2	7,04	0,13		
3	8,65	2,23	2. Enantiomer	
4	13,26	0,14		
Total		100,00		

Compound S17. Prepared according to the general procedure **D** from compound **S8**; pale yellow oil

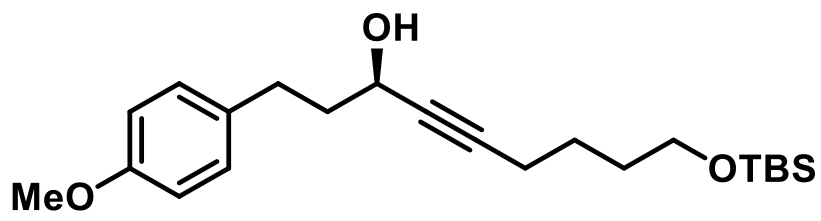

(160 mg, 80%). The e.e. was found to be 98.2% (see below for the HPLC data using a chiral stationary phase). $[\alpha]_D^{20}$ = –20.7 (c = 0.55, $CHCl_3$). ^1H NMR (400 MHz, $CDCl_3$) δ 7.20 – 7.08 (m, 2H), 6.88 – 6.76 (m, 2H), 4.45 – 4.29 (m, 1H), 3.79 (s, 3H), 3.69 – 3.55 (m, 2H), 2.72 (t, *J* = 7.8 Hz, 2H), 2.25 (td, *J* = 6.8, 1.9 Hz, 2H), 2.03 – 1.88 (m, 2H), 1.71 (d, *J* = 5.3 Hz, 1H), 1.67 – 1.54 (m, 4H), 0.89 (s, 9H), 0.05 (s, 6H). ^{13}C NMR (101 MHz, $CDCl_3$) δ 158.0, 133.6, 129.5, 114.0, 85.9, 81.4, 62.8, 62.2, 55.4, 40.0, 32.1, 30.7, 26.1, 25.3, 18.7, 18.5, –5.1. IR (film): $\tilde{\nu}$ 3377, 2950, 2930, 1857, 1612, 1512, 1463, 1388, 1361, 1300, 1247, 1178, 1105, 1039, 1007, 835, 776 cm^{-1}. HRMS (ESI^+) calcd. for $C_{22}H_{36}O_3SiNa$ $[M+Na]^+$: 399.23259; found: 399.23271.

Compound *rac*-S17. Prepared according to the general procedure **C** from compound **S8**; pale yellow oil (70 mg, quant.). NMR, MS, and IR data were found to be identical to those obtained for the enantioenriched compound.

SUJ-SA-451-01 Racemat
150 mm Chiralpak IG-3, 3 µm, 4.6 mm i.D.
CH3CN/H2O = 60:40
1.0 ml/min, 12.5 MPa, 298 K

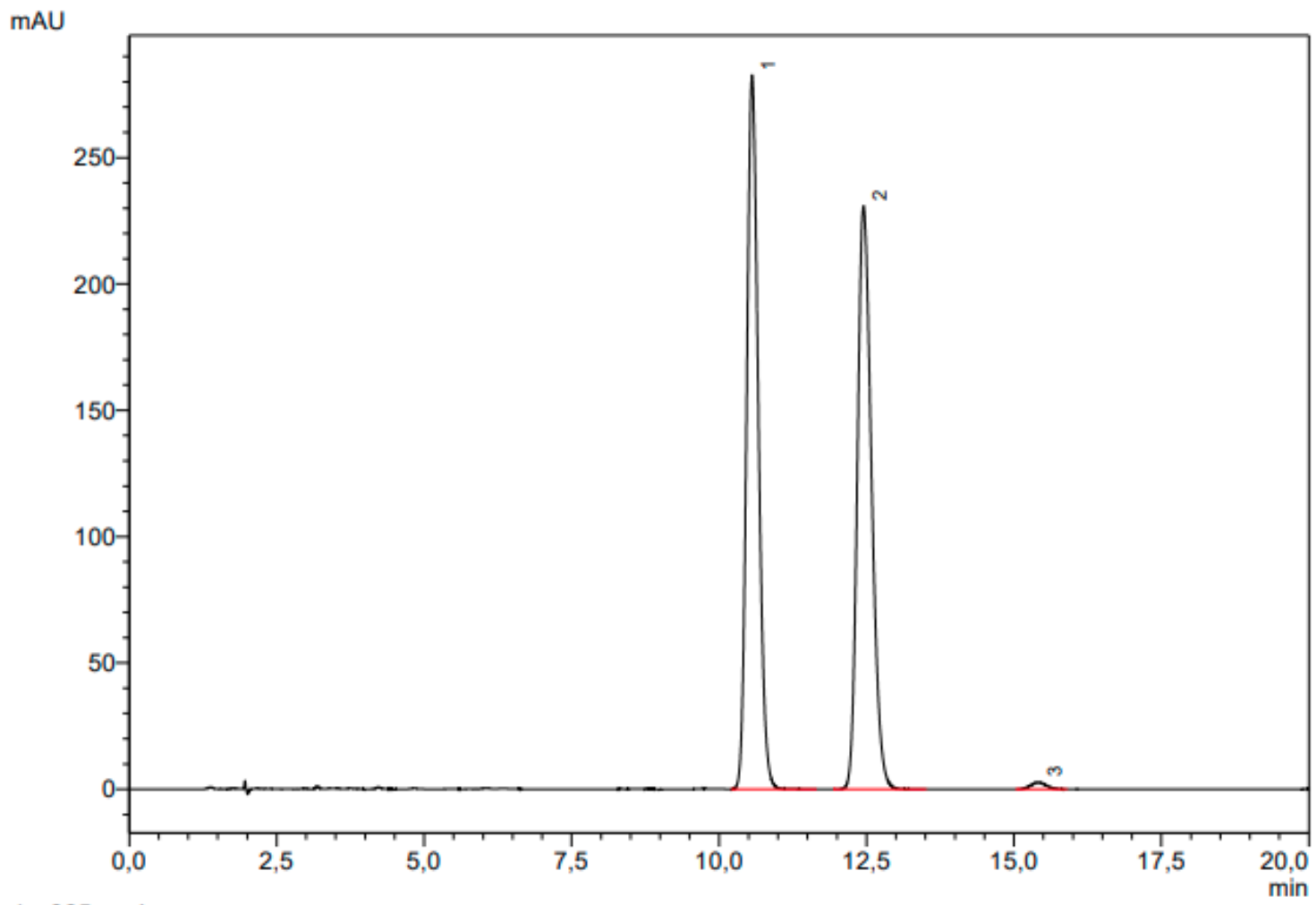

1 225nm,4nm

PDA Ch1 225nm

Peak #	Ret. Time	Area %	Name
1	10,56	49,67	1. Enantiomer
2	12,45	49,71	2. Enantiomer
3	15,42	0,62	
Total		100,00	

SUJ-SA-459-01
150 mm Chiralpak IG-3, 3 µm, 4.6 mm i.D.
CH3CN/H2O = 60:40
1.0 ml/min, 12.5 MPa, 298 K

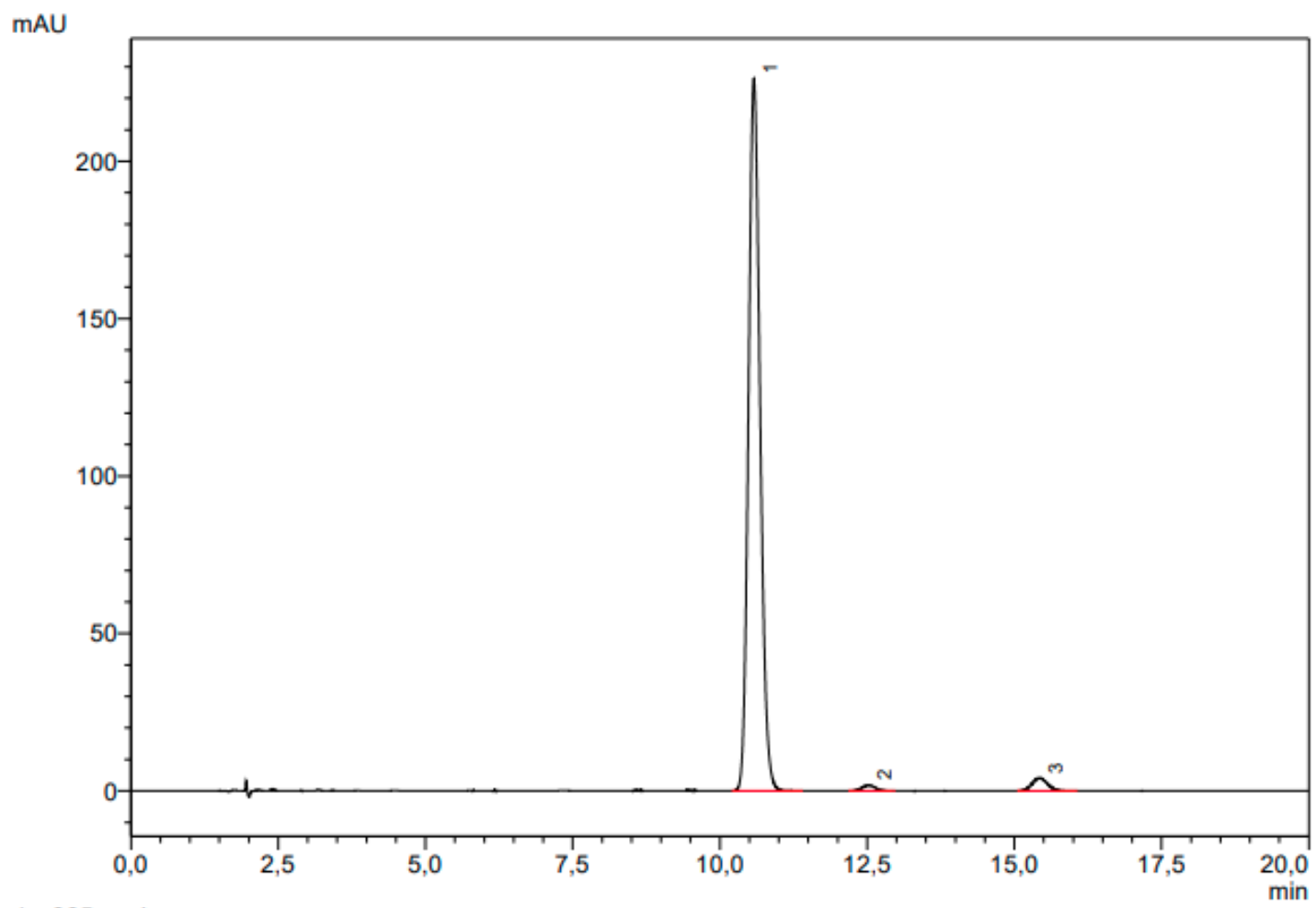

1 225nm,4nm

PDA Ch1 225nm

Peak #	Ret. Time	Area %	Name
1	10,58	96,77	1. Enantiomer 98.2 % ee
2	12,52	0,88	2. Enantiomer
3	15,43	2,35	
Total		100,00	

Compound S18. Prepared according to the general procedure **D** from compound **S10**; pale yellow oil

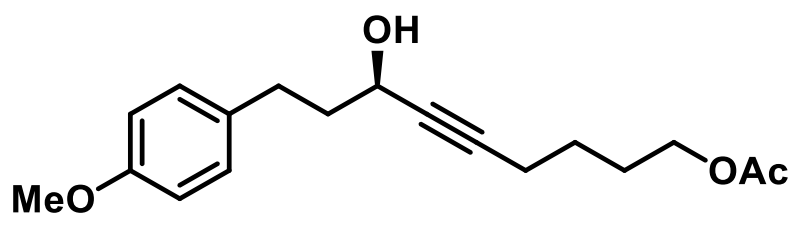

(101 mg, quant.). The e.e. was found to be 96.8% (see below for the HPLC data using a chiral stationary phase). $[\alpha]_D^{20}$= –27.1 (c = 0.25, $CHCl_3$). ^1H NMR (400 MHz, $CDCl_3$) δ 7.17 – 7.06 (m, 2H), 6.89 – 6.74 (m, 2H), 4.34 (ddq, *J* = 8.6, 4.1, 1.9 Hz, 1H), 4.10 (t, *J* = 6.5 Hz, 2H), 3.79 (s, 3H), 2.72 (t, *J* = 7.8 Hz, 2H), 2.28 (td, *J* = 7.0, 2.0 Hz, 2H), 2.05 (s, 3H), 2.02 – 1.89 (m, 2H), 1.82 (d, *J* = 5.4 Hz, 1H), 1.81 – 1.70 (m, 2H), 1.66 – 1.53 (m, 2H). ^{13}C NMR (101 MHz, $CDCl_3$) δ 171.4, 158.0, 133.6, 129.5, 114.0, 85.2, 81.8, 64.1, 62.1, 55.4, 40.0, 30.7, 27.9, 25.2, 21.1, 18.5. IR (film): $\tilde{\nu}$ 3445, 2950, 2864, 1737, 1612, 1512, 1455, 1390, 1366, 1299, 1245, 1178, 1038, 830 cm^{-1}. HRMS (ESI^+) calcd. for $C_{18}H_{24}O_4Na$ $[M+Na]^+$: 327.15668; found: 327.15643.

Compound *rac*-S18. Prepared according to the general procedure **C** from compound **S10**; pale yellow oil (50 mg, 99%). NMR, MS, and IR data were found to be identical to those obtained for the enantioenriched compound.

SUJ-SA-518-01 Racemat
150 mm Chiralpak IG-3, 3 µm, 4.6 mm i.D.
CH3CN / H2O = 60:40
1.0 ml/min, 12.5 MPa, 298 K

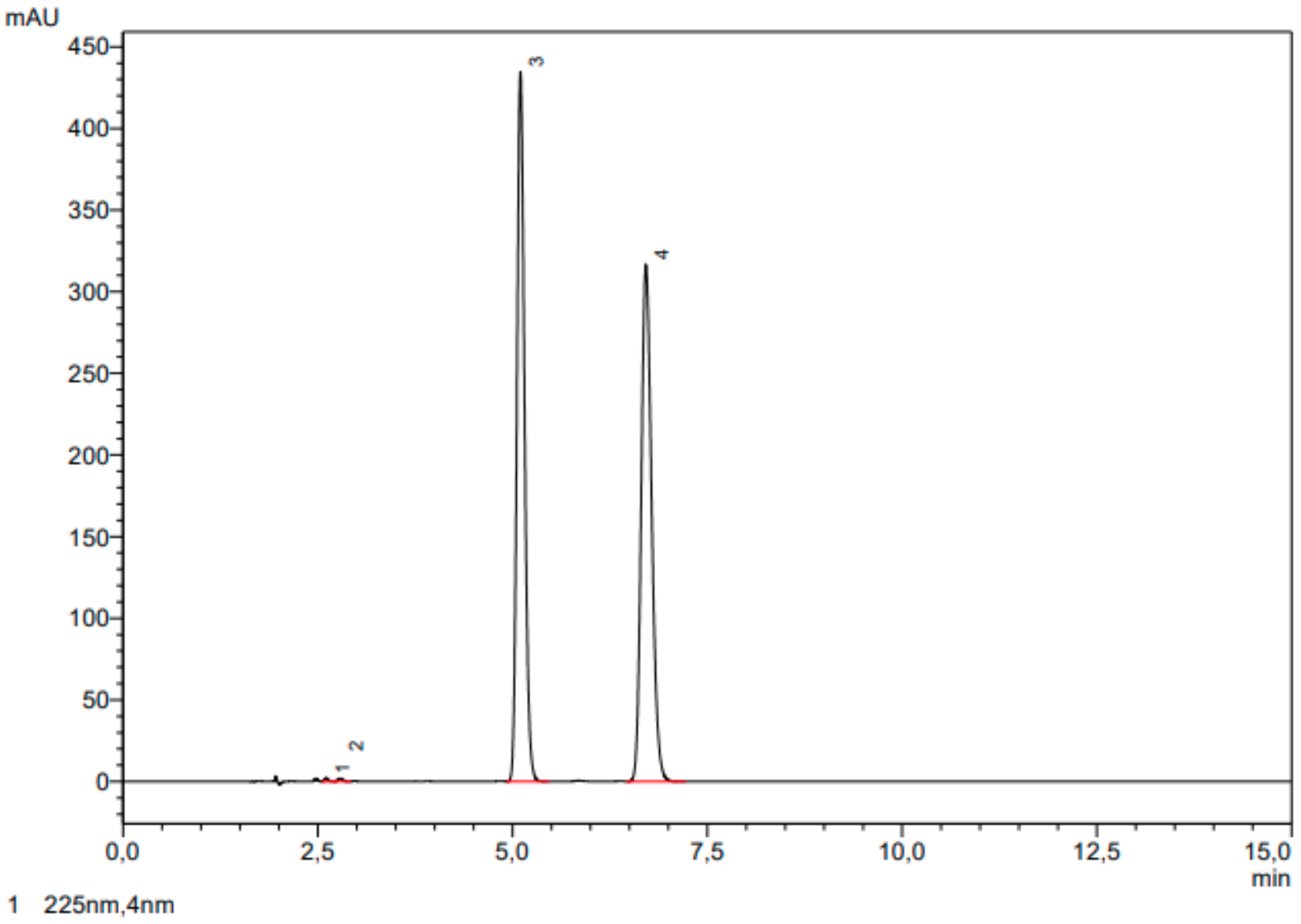

1 225nm,4nm

PDA Ch1 225nm

Peak #	Ret. Time	Area %	Name
1	2,61	0,11	
2	2,79	0,14	
3	5,10	49,77	1. Enantiomer
4	6,71	49,98	2. Enantiomer
Total		100,00	

SUJ-SA-521-01
150 mm Chiralpak IG-3, 3 µm, 4.6 mm i.D.
CH3CN / H2O = 60:40
1.0 ml/min, 12.5 MPa, 298 K

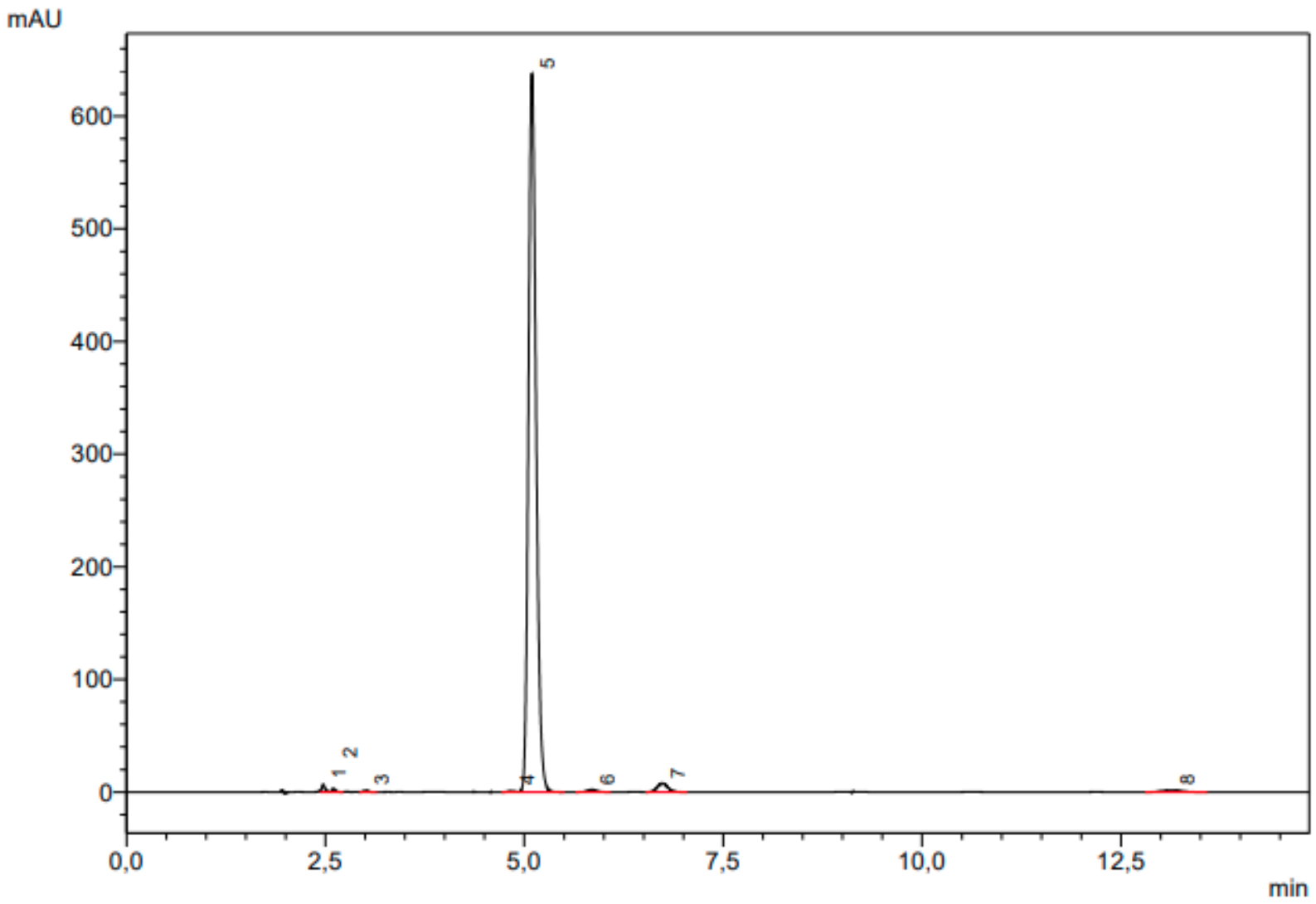

1 225nm,4nm

PDA Ch1 225nm

Peak #	Ret. Time	Area %	Name
1	2,47	0,44	
2	2,60	0,19	
3	3,01	0,12	
4	4,84	0,13	
5	5,10	96,48	1. Enantiomer 96.8 % ee
6	5,85	0,36	
7	6,73	1,58	2. Enantiomer
8	13,14	0,70	
Total		100,00	

Compound S19. Prepared according to the general procedure **D** from compound **S11**; pale yellow oil

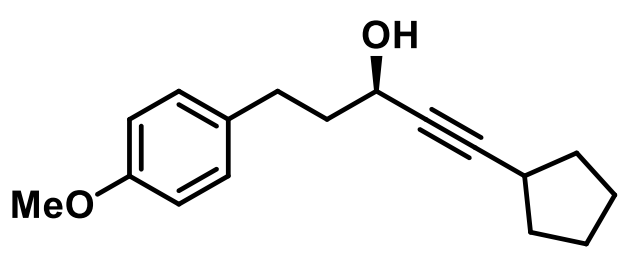

(195 mg, 94%). The e.e. was found to be 98.6% (see below for the HPLC data using a chiral stationary phase). $[\alpha]_D^{20}$= −27.7 (c = 0.25, $CHCl_3$). ^{1}H NMR (400 MHz, $CDCl_3$) δ 7.18 – 7.07 (m, 2H), 6.97 – 6.76 (m, 2H), 4.40 – 4.27 (m, 1H), 3.79 (s, 3H), 2.72 (t, *J* = 7.8 Hz, 2H), 2.68 – 2.57 (m, 1H), 2.03 – 1.84 (m, 4H), 1.77 – 1.67 (m, 2H), 1.66 – 1.46 (m, 4H). ^{13}C NMR (101 MHz, $CDCl_3$) δ 158.0, 133.7, 129.5, 114.0, 90.4, 80.6, 62.2, 55.4, 40.1, 34.01, 33.99, 30.7, 30.2, 25.1. IR (film): $\tilde{\nu}$ 3390, 2950, 2868, 2835, 1612, 1511, 1453, 1337, 1300, 1244, 1177, 1037, 829 cm^{-1}. HRMS (EI$^+$) calcd. for $C_{17}H_{22}O_2$ $[M]^+$: 258.16143; found: 258.16136.

Compound *rac*-S19. Prepared according to the general procedure **C** from compound **S11**; pale yellow oil (99.8 mg, 99%). NMR, MS, and IR data were found to be identical to those obtained for the enantioenriched compound.

SUJ-SA-504-01 Racemat
150 mm Chiralpak IG-3, 3 µm, 4.6 mm i.D.
CH3CN / H2O = 60:40
1.0 ml/min, 12.5 MPa, 298 K

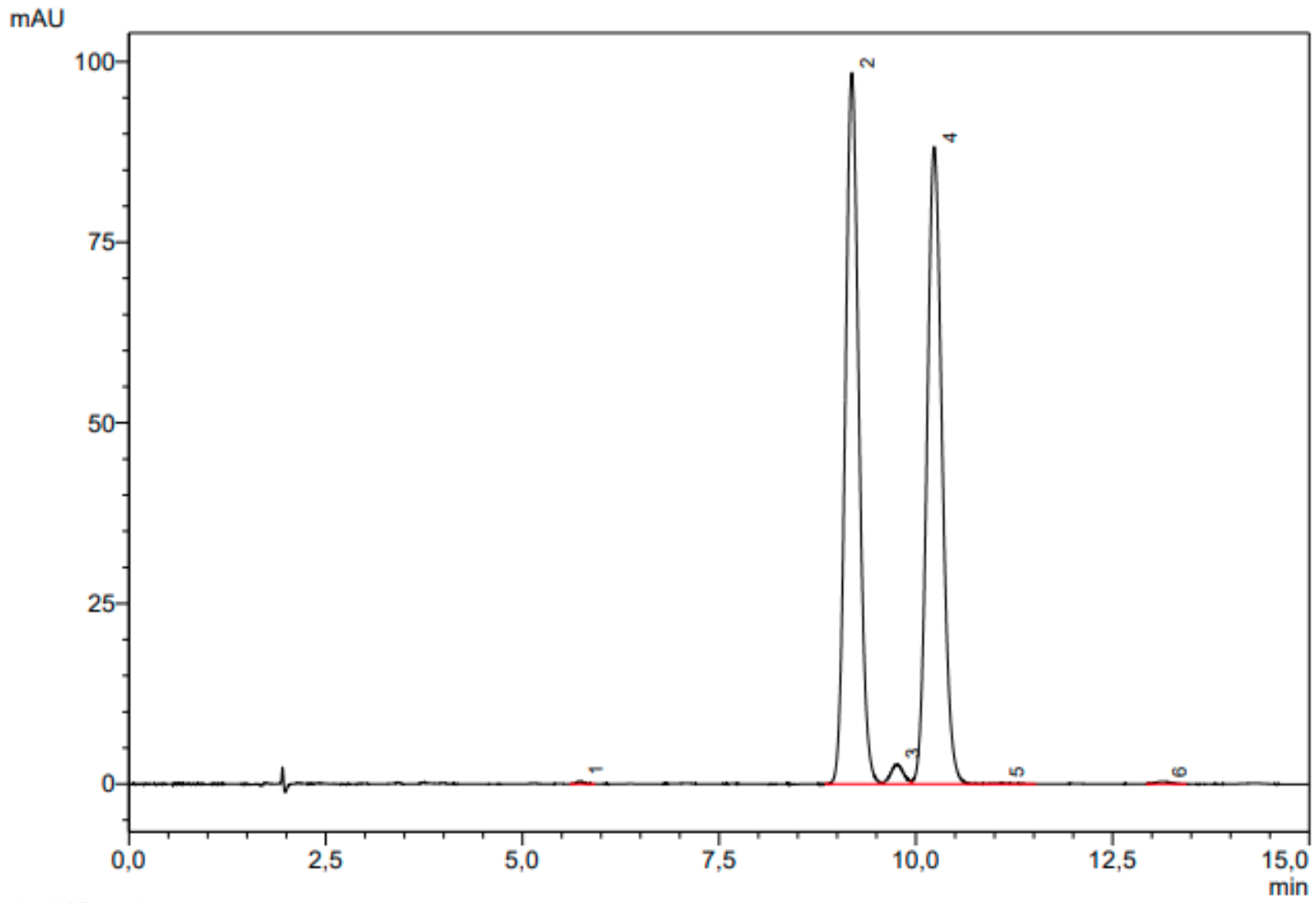

1 225nm,4nm

PDA Ch1 225nm

Peak #	Ret. Time	Area %	Name
1	5,73	0,10	
2	9,19	49,21	1. Enantiomer
3	9,76	1,30	
4	10,24	49,10	2. Enantiomer
5	11,09	0,10	
6	13,16	0,19	
Total		100,00	

SUJ-SA-522-01
150 mm Chiralpak IG-3, 3 µm, 4.6 mm i.D.
CH3CN / H2O = 60:40
1.0 ml/min, 12.5 MPa, 298 K

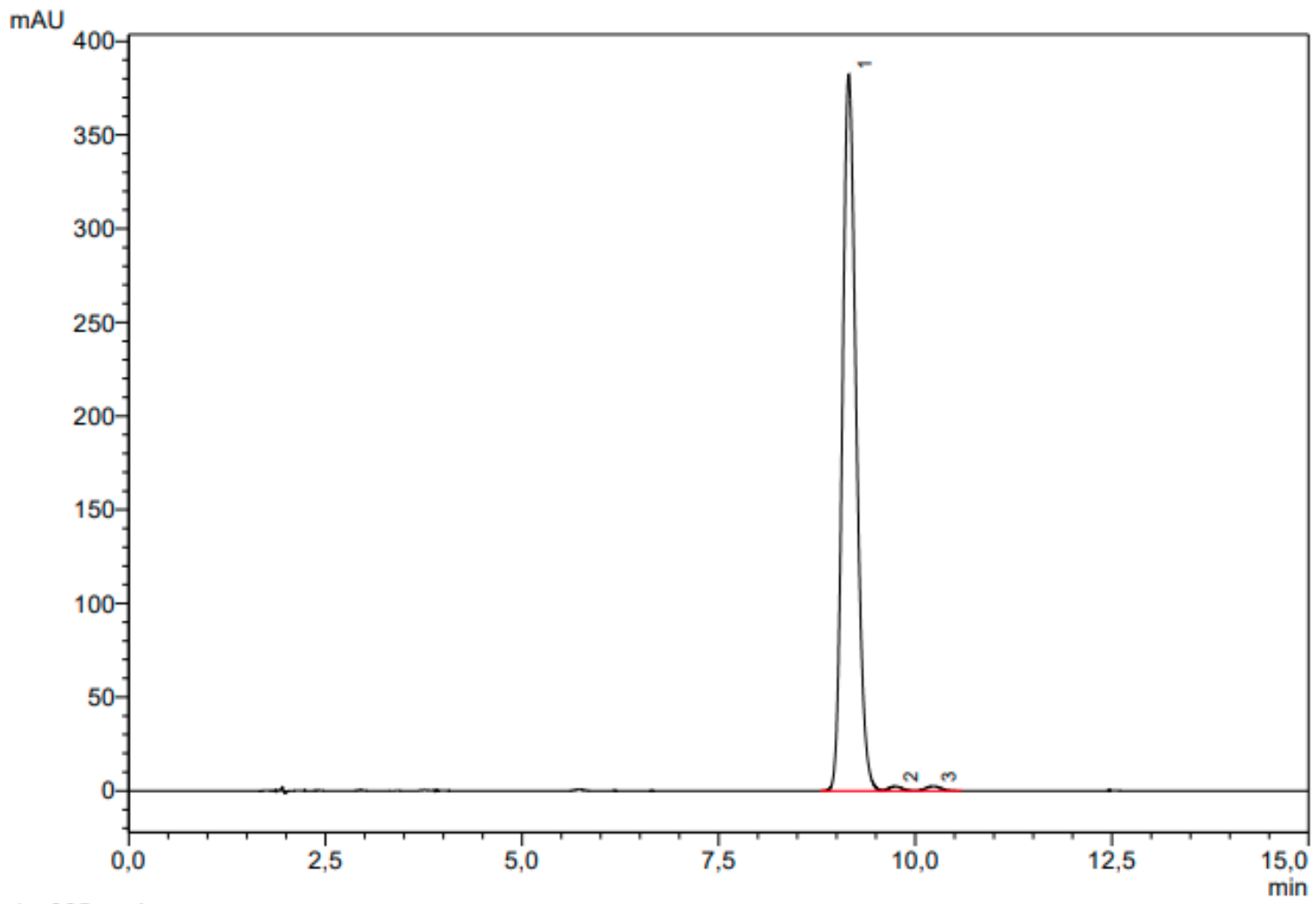

1 225nm,4nm

PDA Ch1 225nm

Peak #	Ret. Time	Area %	Name
1	9,16	98,72	1. Enantiomer 98.6 % ee
2	9,75	0,59	
3	10,23	0,69	2. Enantiomer
Total		100,00	

Compound S20. Prepared according to the general procedure **D** from compound **S12**; pale yellow oil

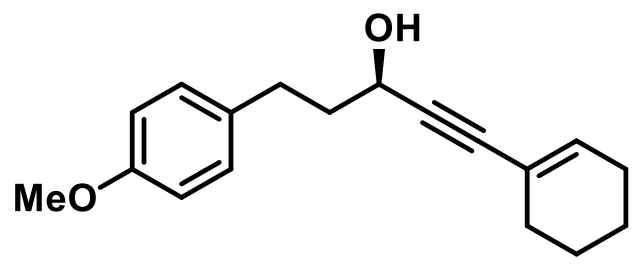

(97.9 mg, 97%). The e.e. was found to be 98.5% (see below for the HPLC data using a chiral stationary phase). $[\alpha]_D^{20}$= −32.4 (c = 0.21, $CHCl_3$). ^{1}H NMR (400 MHz, $CDCl_3$) δ 7.19 – 7.05 (m, 2H), 6.95 – 6.67 (m, 2H), 6.27 – 5.99 (m, 1H), 4.47 (t, *J* = 6.5 Hz, 1H), 3.79 (s, 3H), 2.74 (t, *J* = 7.8 Hz, 2H), 2.22 – 2.05 (m, 4H), 2.06 – 1.92 (m, 2H), 1.70 – 1.41 (m, 4H). ^{13}C NMR (101 MHz, $CDCl_3$) δ 158.0, 135.5, 133.6, 129.5, 120.2, 114.0, 87.28, 87.27, 62.4, 55.4, 39.8, 30.7, 29.3, 25.8, 22.4, 21.6. IR (film): $\tilde{\nu}$ 3401, 2931, 2859, 2835, 1612, 1512, 1440, 1338, 1300, 1245, 1177, 1109, 1036, 919, 830 cm^{-1}. HRMS (EI$^+$) calcd. for $C_{18}H_{22}O_2$ [M]$^+$: 270.16143; found: 270.16113.

Compound *rac*-S20. Prepared according to the general procedure **C** from compound **S12**; pale yellow oil (179 mg, quant.). NMR, MS, and IR data were found to be identical to those obtained for the enantioenriched compound.

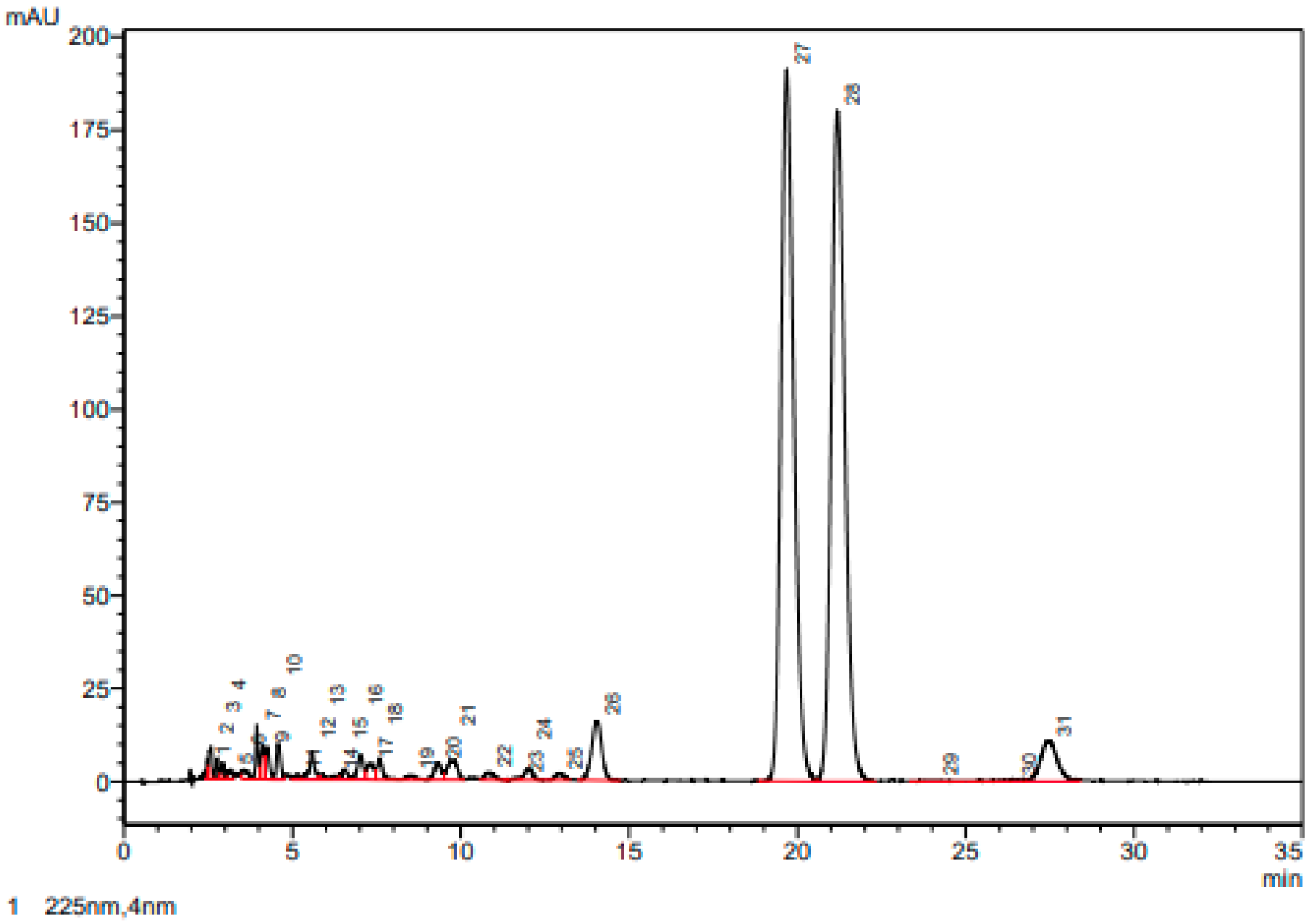

Peak #	Ret. Time	Area %	Name
20	9,32	0,54	
21	9,77	0,86	
22	10,84	0,32	
23	11,77	0,07	
24	12,02	0,41	
25	12,95	0,27	
26	14,05	2,77	
27	19,69	42,16	1. Enantiomer
28	21,19	42,07	2. Enantiomer
29	24,07	0,12	
30	26,43	0,24	
31	27,47	3,12	
Total		100,00	

SUJ-SA-529-01
150 mm Chiralpak IG-3, 3 µm, 4.6 mm i.D.
Acetonitril / Wasser = 55:45
1.0 ml/min, 13.5 MPa, 298 K

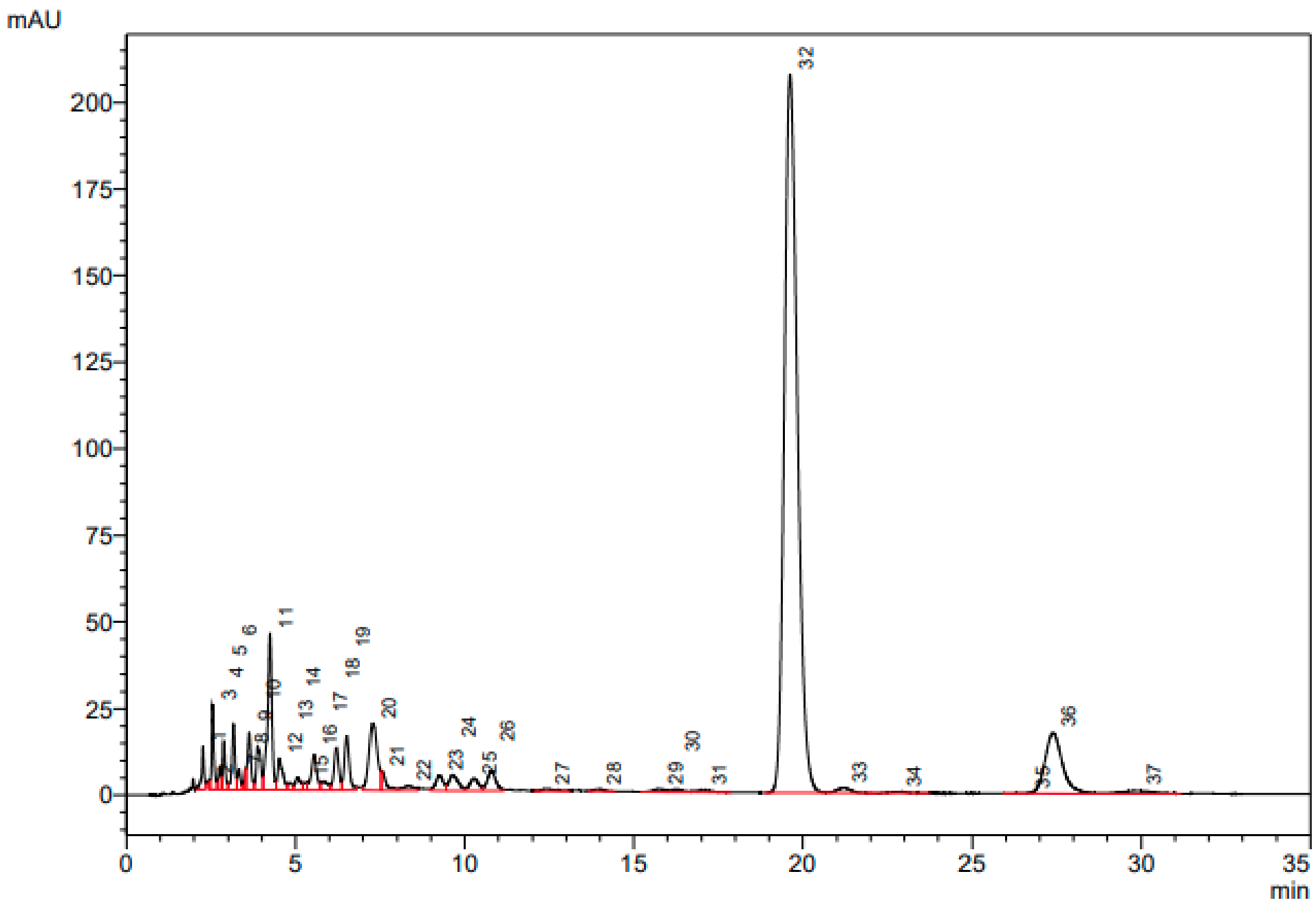

1 225nm,4nm

Peak #	Ret. Time	Area %	Name
20	7,29	3,56	
21	7,53	0,64	
22	8,33	0,36	
23	9,26	0,78	
24	9,65	0,98	
25	10,28	0,73	
26	10,80	1,14	
27	12,41	0,22	
28	13,96	0,16	
29	15,77	0,22	
30	16,25	0,22	
31	17,05	0,22	
32	19,63	62,74	1. Enantiomer 98.5 % ee
33	21,21	0,48	2. Enantiomer
34	22,80	0,17	
35	26,62	0,04	
36	27,40	7,13	
37	29,91	0,60	
Total		100,00	

Compound S21. Prepared according to the general procedure **D** from compound **S13**; pale yellow oil

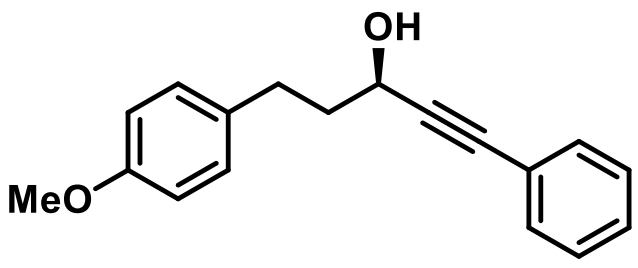

(498 mg, 1.87 mmol, 99% yield). The e.e. was found to be 97.2% (see below for the HPLC data using a chiral stationary phase). $[\alpha]_D^{20}$= −81.1 (c = 1.00, $CHCl_3$). ^{1}H NMR (400 MHz, $CDCl_3$) δ 7.46 – 7.43 (m, 2H), 7.33 – 7.31 (m, 3H), 7.18 – 7.16 (m, 2H), 6.86 – 6.84 (m, 2H), 4.59 (td, *J* = 6.9, 3.1 Hz, 1H), 3.80 (s, 3H), 2.82 (t, *J* = 7.8 Hz, 2H), 2.10 (tdd, *J* = 7.6, 6.4, 4.1 Hz, 2H), 1.95 (br s, 1H). ^{13}C NMR (101 MHz, $CDCl_3$) δ 158.1, 133.4, 131.8, 129.6, 128.6, 128.4, 122.7, 114.0, 90.0, 85.4, 62.4, 55.4, 39.7, 30.7. IR (neat): ν 3378, 2931, 1161, 1510, 1489, 1463, 1454, 1442, 1420, 1333, 1299, 1242, 1177, 1035, 1011, 917, 282, 755, 690, 569, 540, 523 cm^{-1}. HRMS (ESI$^+$) calcd. for $C_{18}H_{18}O_2Na$ [M+Na]$^+$: 266.13013; found: 266.12989.

Compound *rac*-S21. Prepared according to the general procedure **C** from compound **S13**; pale yellow oil (150 mg, 90%). NMR, MS, and IR data were found to be identical to those obtained for the enantioenriched compound.

MOP-MA-476-01 Racemat
150 mm Chiralpak IG-3, 3 µm, 4.6 mm i.D.
CH3CN / H2O = 60:40
1.0 ml/min / 242 bar / 298 K

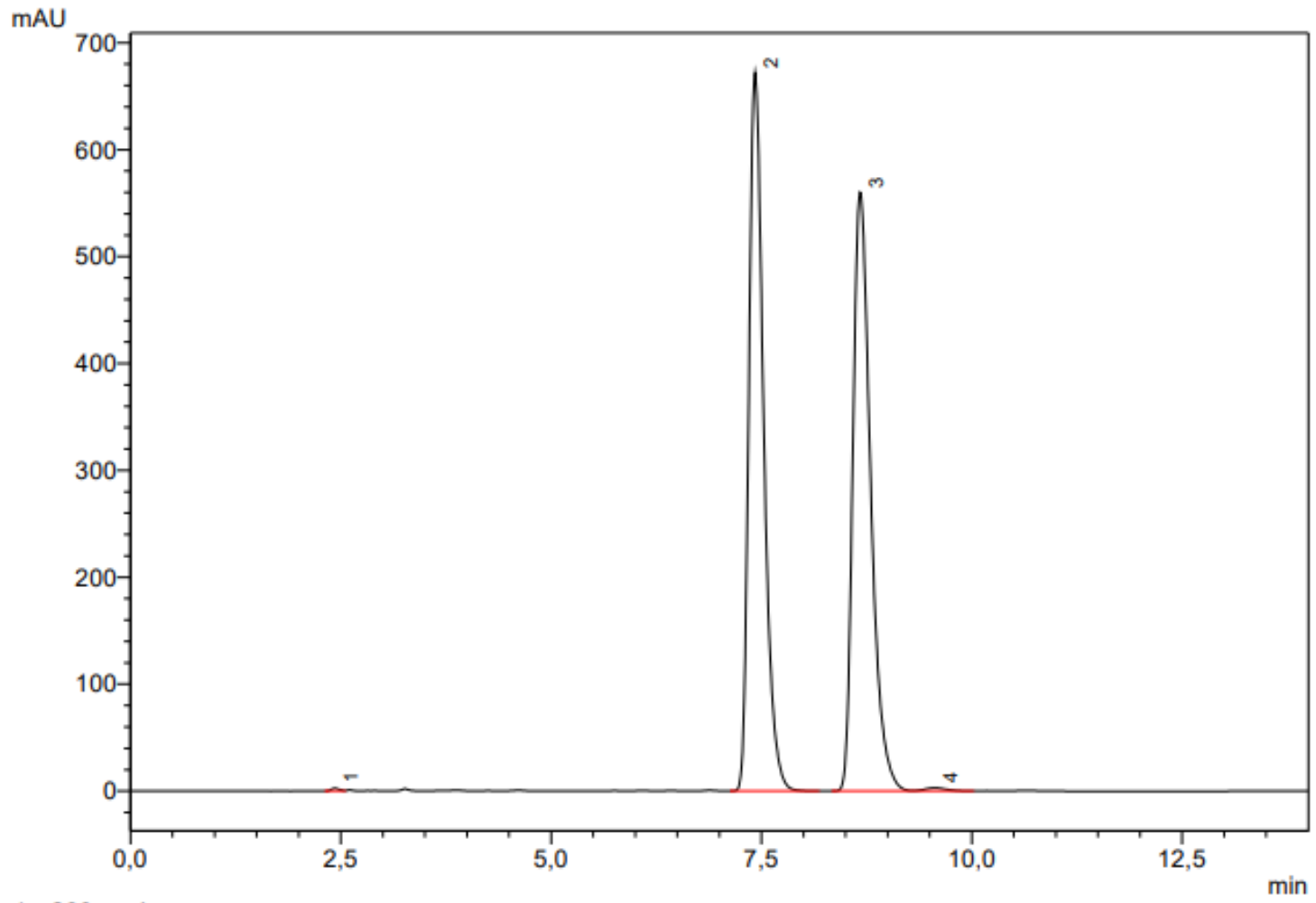

1 230nm,4nm

PDA Ch1 230nm

Peak #	Ret. Time	Area %	Name
1	2,43	0,11	
2	7,43	49,71	1. Enantiomer
3	8,68	49,85	2. Enantiomer
4	9,56	0,32	
Total		100,00	

MOP-MA-477-01
150 mm Chiralpak IG-3, 3 µm, 4.6 mm i.D.
CH3CN / H2O = 60:40
1.0 ml/min / 242 bar / 298 K

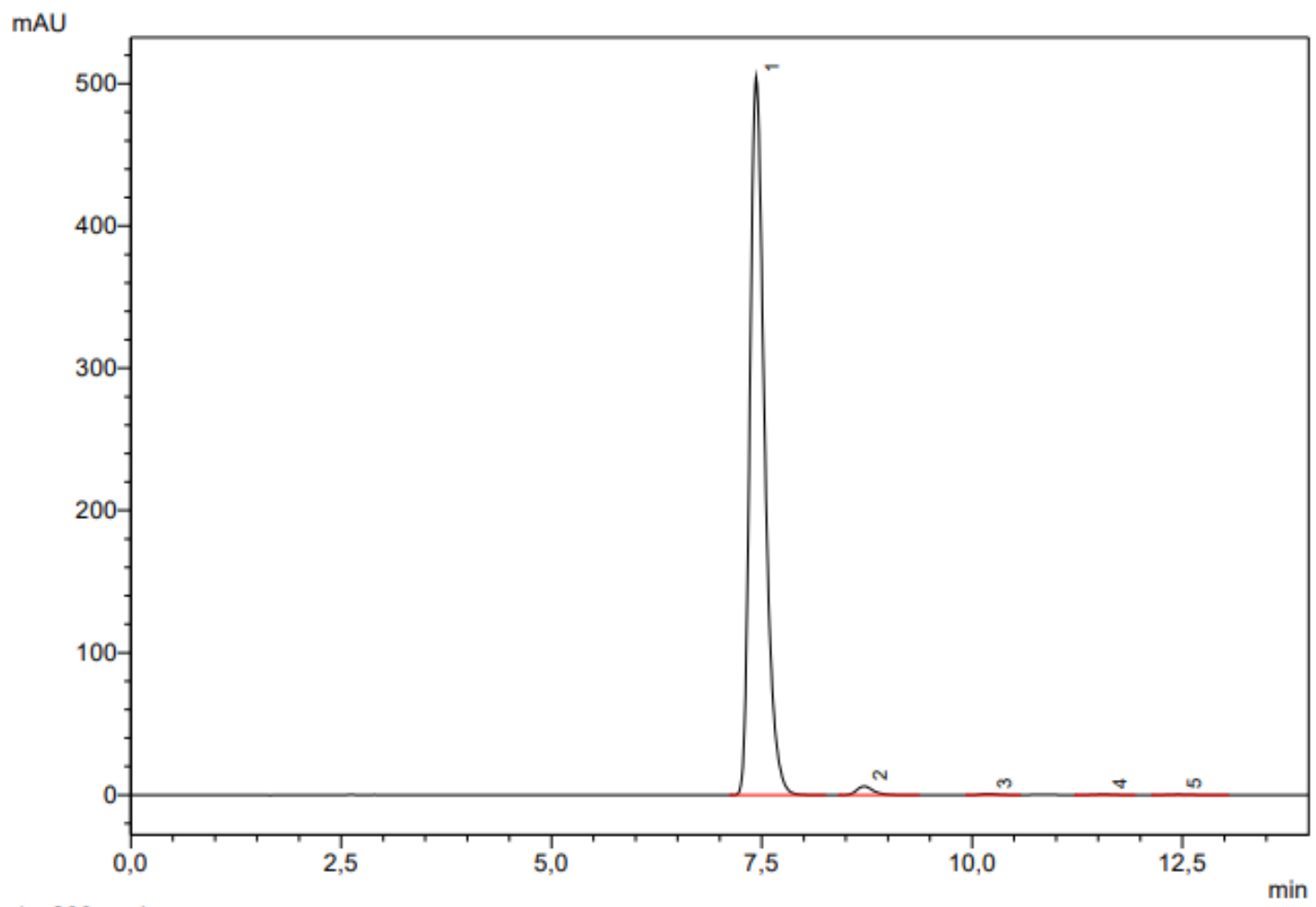

1 230nm,4nm

PDA Ch1 230nm

Peak #	Ret. Time	Area %	Name
1	7,43	98,20	1. Enantiomer 97.2 % ee
2	8,72	1,37	2. Enantiomer
3	10,20	0,19	
4	11,57	0,12	
5	12,45	0,12	
Total		100,00	

Compound S22. Prepared according to the literature;[3],[4] pale yellow oil (561 mg, 35%, 79% brsm).

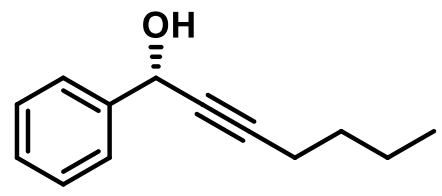

The e.e. was found to be 68.3% (see below for the HPLC data using a chiral stationary phase). $[\alpha]_D^{20}$= –13.5 (c = 1.01, $CHCl_3$). ^1H NMR (400 MHz, $CDCl_3$) δ 7.57 – 7.52 (m, 2H), 7.43 – 7.35 (m, 2H), 7.35 – 7.29 (m, 1H), 5.46 (dt, *J* = 6.3, 2.1 Hz, 1H), 2.28 (td, *J* = 7.1, 2.0 Hz, 2H), 2.07 (d, *J* = 6.2 Hz, 1H), 1.61 – 1.48 (m, 2H), 1.46 – 1.37 (m, 2H), 0.92 (t, *J* = 7.3 Hz, 3H). ^{13}C NMR (101 MHz, $CDCl_3$) δ 141.4, 128.7, 128.4, 126.8, 87.9, 80.0, 65.0, 30.8, 22.1, 18.7, 13.7. IR (film): $\tilde{\nu}$ 3367, 2957, 2931, 1871, 1493, 1453, 1430, 1379, 1327, 1276, 1250, 1192, 1134, 1105, 1075, 999, 917, 825, 696, 634 cm^{-1}. HRMS (EI^+) calcd. for $C_{13}H_{16}O$ $[M]^+$: 188.119565; found: 188.119640.

NMR, MS, and IR data for *rac*-**S22**[5] were found to be identical to those obtained for the enantioenriched compound.

SFS-SD-973-20-Racemat
150 mm Chiralpak IG-3, 3 µm, 4.6 mm i.D.
n-Heptan / 2-Propanol = 95:5
1.0 ml/min / 8.5 MPa / 298 K

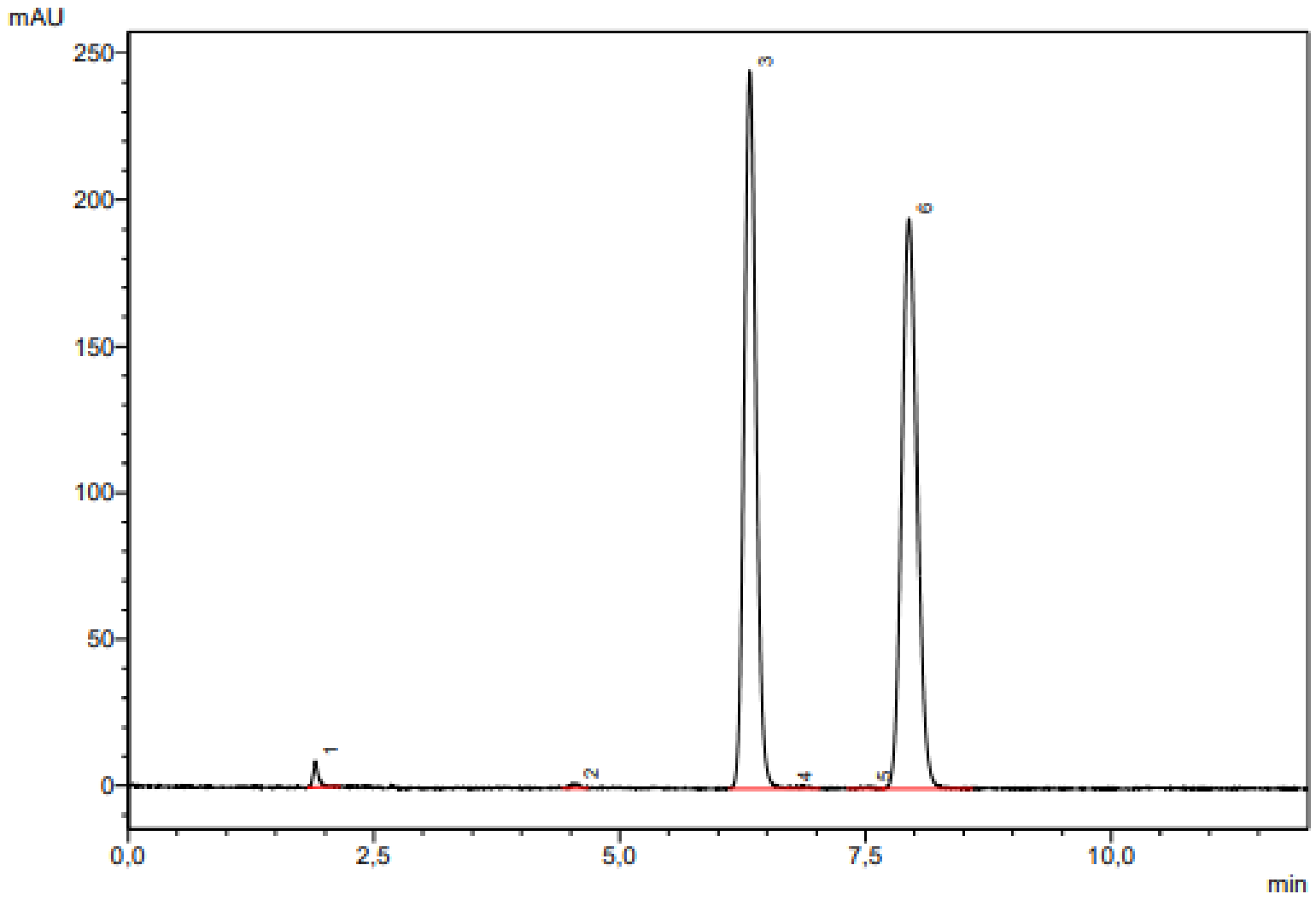

1 210nm,4nm

PDA Ch1 210nm

Peak #	Ret. Time	Area %	Name
1	1,90	0,77	
2	4,55	0,27	
3	6,33	49,24	1. Enantiomer
4	6,72	0,22	
5	7,53	0,14	
6	7,95	49,37	2. Enantiomer
Total		100,00	

SFS-SD-973-11
150 mm Chiralpak IG-3, 3 µm, 4.6 mm i.D.
n-Heptan / 2-Propanol = 95:5
1.0 ml/min / 8.5 MPa / 298 K

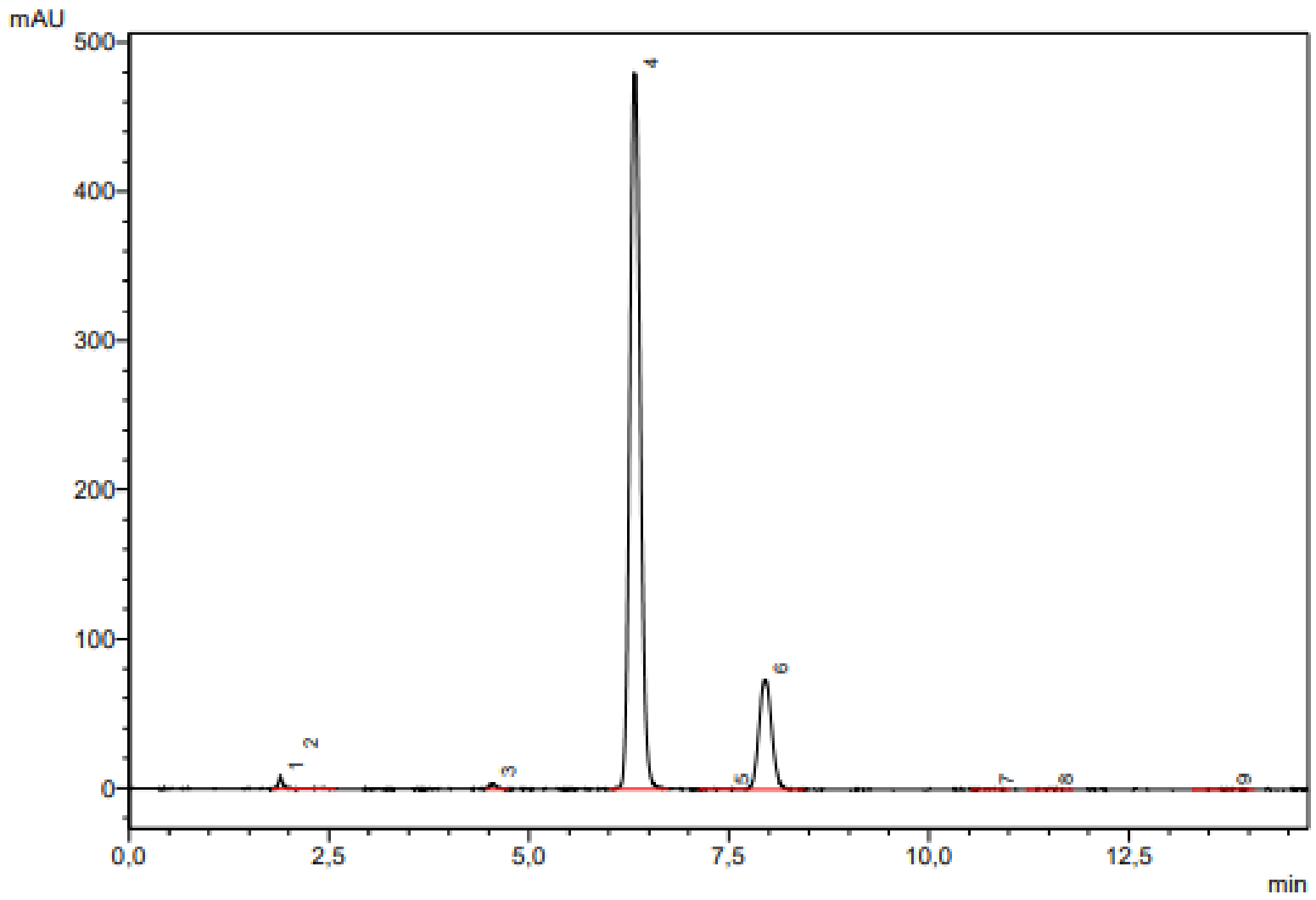

1 210nm,4nm

PDA Ch1 210nm

Peak #	Ret. Time	Area %	Name
1	1,89	0,65	
2	2,07	0,15	
3	4,55	0,48	
4	6,32	82,62	1. Enantiomer 68.3 % ee
5	7,47	0,10	
6	7,95	15,57	2. Enantiomer
7	10,77	0,10	
8	11,51	0,17	
9	13,75	0,16	
Total		100,00	

Compound S23. A solution of Weinreb amide **S1** (238 mg, 1.06 mmol) in THF (10 mL) was cooled to

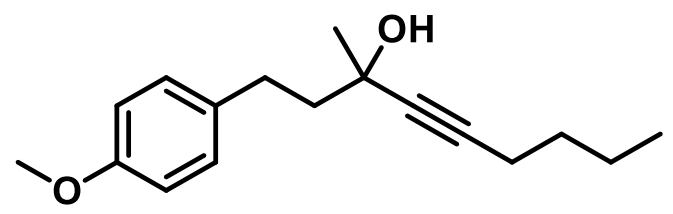

–78 °C (bath temperature). Methylmagnesium bromide (3 M in diethyl ether, 0.71 mL, 2.13 mmol) was added, and the reaction was warmed to room temperature over 1 h. Saturated aqueous ammonium chloride solution was added slowly, and the reaction was diluted with ethyl acetate and water. The organic phase was separated, and the aqueous layer was extracted three times with ethyl acetate. The combined organic extracts were washed with brine, dried over anhydrous magnesium sulfate, and filtered.

A solution of the 1-hexyne (0.518 mL, 1.38 mmol) in THF (10 mL) was cooled to –78 °C (bath temperature) in a flame-dried Schlenk flask. *n*-Butyllithium (1.6 M in hexane, 0.785 mL, 1.27 mmol.) was added dropwise, and the resulting solution was warmed to room temperature over a period of 30 minutes, after which it was cooled back to –78 °C. A solution of the crude residue from the previous step in THF (10 mL) was added dropwise, and the reaction was warmed to room temperature and stirred for 2 h. Saturated aqueous ammonium chloride solution was added slowly, and the reaction was diluted with ethyl acetate and water. The organic layer was separated, and the aqueous phase was extracted three times with ethyl acetate. The combined organic extracts were washed with brine, dried over anhydrous magnesium sulfate, and filtered. The filtrate was concentrated under reduced pressure, and the residue was purified *via* flash chromatography (0-10% ethyl acetate/cyclohexane) to give the title compound as a colorless oil (186 mg, 67%). ^{1}H NMR (400 MHz, $CDCl_3$) δ 7.20 – 7.06 (m, 2H), 6.89 – 6.73 (m, 2H), 3.79 (s, 3H), 2.90 – 2.66 (m, 2H), 2.23 (t, *J* = 7.0 Hz, 2H), 2.03 – 1.82 (m, 2H), 1.58 – 1.37 (m, 7H), 0.93 (t, *J* = 7.2 Hz, 3H). ^{13}C NMR (101 MHz, $CDCl_3$) δ 157.9, 134.3, 129.4, 114.0, 84.5, 83.8, 68.4, 55.4, 46.1, 31.0, 30.6, 30.5, 22.1, 18.5, 13.8. IR (film): $\tilde{\nu}$ 3401, 1955, 2932, 2862, 2836, 1612, 1584, 1512, 1464, 1368, 1326, 1300, 1245, 1177, 1109, 1083, 1036, 938, 888 cm^{-1}. HRMS (EI$^+$) calcd. for $C_{17}H_{24}O_2$ $[M]^+$: 260.17708; found: 260.17714.

α-Silyl Allylic Alcohols

Note: *For the following compounds, the reader will note that the ethoxy groups bound to the silicon atom are diastereotopic, and so a difference in the NMR resonance frequencies of the carbon nuclei is expected. Indeed, it was often found that the carbon nuclei of the terminal methyl groups exhibited detectably different NMR shifts, whereas only one (rather broad) peak was observed for the two diastereotopic methylene groups. The occasional coincidence of these resonances accounts for any apparent discrepancy in the total number of carbon shifts reported.*

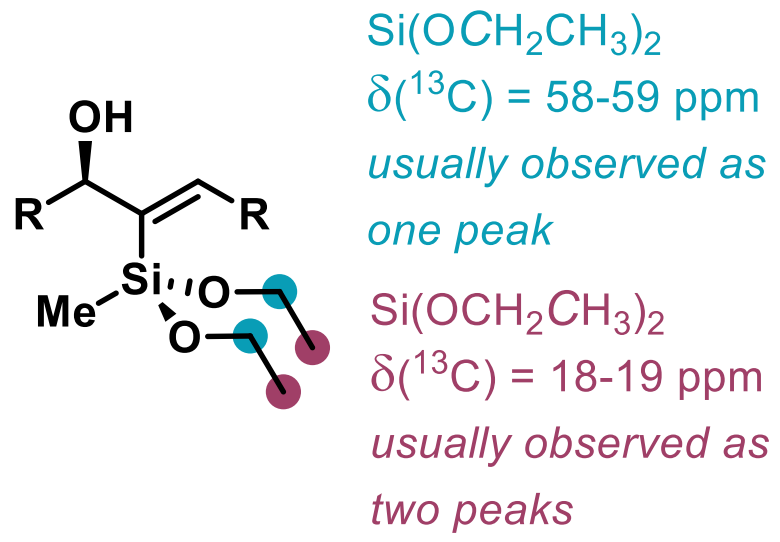

Compound 1. Prepared according to the general procedure **E** from compound **4**; yellow oil (543 mg, 58%). $[\alpha]_D^{20}$= 4.7 (c = 0.83, $CHCl_3$). ^{1}H NMR (400 MHz, $CDCl_3$) δ 7.15 – 7.06 (m, 2H), 6.87 – 6.79 (m, 2H), 6.11 (t, *J* = 7.6 Hz, 1H), 3.93 (t, *J* = 7.0 Hz, 1H), 3.89 – 3.67 (m, 7H), 3.47 (s, 1H), 2.66 (ddd, *J* = 13.3, 10.3, 5.6 Hz, 1H), 2.55 (ddd, *J* = 13.7, 10.1, 6.1 Hz, 1H), 2.39 – 2.10 (m, 2H), 2.07 – 1.89 (m, 1H), 1.78 (dddd, *J* = 16.4, 12.2, 6.1, 3.1 Hz, 1H), 1.48 – 1.29 (m, 4H), 1.24 (tdd, *J* = 6.9, 5.0, 1.2 Hz, 6H), 0.91 (tt, *J* = 7.2, 1.2 Hz, 3H), 0.28 (d, *J* = 1.2 Hz, 3H). ^{13}C NMR (101 MHz, $CDCl_3$) δ 157.8, 146.9, 138.3, 134.6, 129.4, 113.9, 79.7, 58.4, 55.4, 40.8, 31.99, 31.95, 31.4, 22.6, 18.33, 18.28, 14.2, −1.9. IR (film): $\tilde{\nu}$ 3502, 2956, 2925, 2873, 2835, 1612, 1584, 1511, 1455, 1412, 1390, 1364, 1299, 1244, 1175, 1165, 1070, 1101, 1038, 946, 873, 819, 802, 760, 730, 702, 690, 638, 566, 546, 513, 444, 429, 213 cm^{-1}. HRMS (ESI$^+$) calcd. for $C_{21}H_{36}O_4SiNa$ $[M+Na]^+$: 403.22751; found: 403.22766.

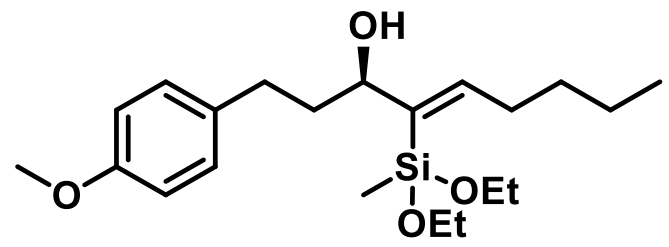

Compound S24. Prepared according to the general procedure **E** from compound **S14**; yellow oil (59 mg, 77%). $[\alpha]_D^{20}$= 2.7 (c = 0.41, $CHCl_3$). ^{1}H NMR (400 MHz, $CDCl_3$) δ 7.26 – 7.22 (m, 2H), 7.16 – 7.06 (m, 2H), 6.10 (td, *J* = 7.6, 0.9 Hz, 1H), 3.98 – 3.86 (m, 1H), 3.79 (qd, *J* = 7.0, 3.0 Hz, 4H), 3.46 (d, *J* = 10.2 Hz, 1H), 2.69 (ddd, *J* = 13.8, 10.1, 5.6 Hz, 1H), 2.58 (ddd, *J* = 13.9, 9.9, 6.3 Hz, 1H), 2.35 – 2.10 (m, 2H), 1.96 (dddd, *J* = 13.6, 9.9, 8.0, 5.6 Hz, 1H), 1.76 (ddt, *J* = 13.4, 10.1, 6.2 Hz, 1H), 1.45 – 1.29 (m, 4H), 1.27 – 1.12 (m, 6H), 1.00 – 0.83 (m, 3H), 0.27 (s, 3H). ^{13}C NMR (101 MHz, $CDCl_3$) δ 147.0, 141.0, 138.3, 131.5, 130.0, 128.5, 79.4, 58.4, 40.5, 32.2, 32.0, 31.4, 22.6, 18.34, 18.28, 14.2, −1.9. IR (film): $\tilde{\nu}$ 3495, 2959, 2925, 2874, 1614, 1492, 1456, 1407, 1390, 1294, 1258, 1164, 1100, 1073, 1015, 948, 818, 805, 726, 508 cm^{-1}. HRMS (ESI$^+$) calcd. for $C_{20}H_{33}O_3ClSiNa$ $[M+Na]^+$: 407.17797; found: 407.17754.

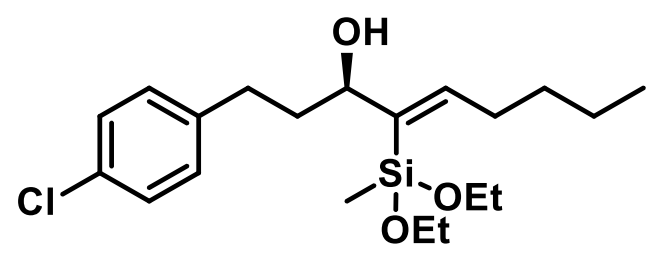

Compound S25. Prepared according to the general procedure **E** from compound **S15**; yellow oil (85

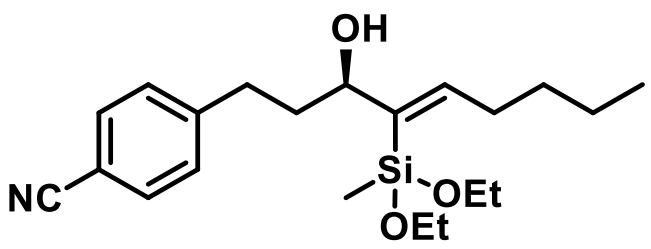

mg, 78%). $[\alpha]_D^{20}$= 7.5 (c = 0.34, $CHCl_3$). ^1H NMR (400 MHz, $CDCl_3$) δ 7.60 – 7.52 (m, 2H), 7.31 – 7.27 (m, 2H), 6.10 (td, *J* = 7.6, 0.9 Hz, 1H), 3.98 – 3.85 (m, 1H), 3.86 – 3.72 (m, 4H), 3.47 (d, *J* = 10.1 Hz, 1H), 2.80 (ddd, *J* = 13.8, 10.0, 5.5 Hz, 1H), 2.69 (ddd, *J* = 13.9, 9.8, 6.5 Hz, 1H), 2.20 (ttd, *J* = 14.3, 7.7, 4.0 Hz, 2H), 1.98 (dddd, *J* = 13.8, 9.9, 8.4, 5.6 Hz, 1H), 1.78 (dddd, *J* = 13.5, 10.0, 6.6, 5.6 Hz, 1H), 1.43 – 1.29 (m, 4H), 1.23 (td, *J* = 7.0, 4.7 Hz, 6H), 0.99 – 0.85 (m, 3H), 0.27 (s, 3H). ^{13}C NMR (101 MHz, $CDCl_3$) δ 148.4, 147.1, 138.2, 132.3, 129.4, 119.3, 109.7, 79.2, 58.5, 58.4, 40.0, 33.0, 31.9, 31.4, 22.6, 18.34, 18.28, 14.2, –1.9. IR (film): $\tilde{\nu}$ 3489, 2970, 2926, 2874, 2228, 1608, 1442, 1413, 1390, 1258, 1165, 1102, 1075, 950, 821, 802, 763 cm^{-1}. HRMS (ESI$^+$) calcd. for $C_{21}H_{33}NO_3SiNa$ $[M+Na]^+$: 398.21219; found: 398.21177.

Compound S26. Prepared according to the general procedure **E** from compound **S16**; yellow oil (49.6

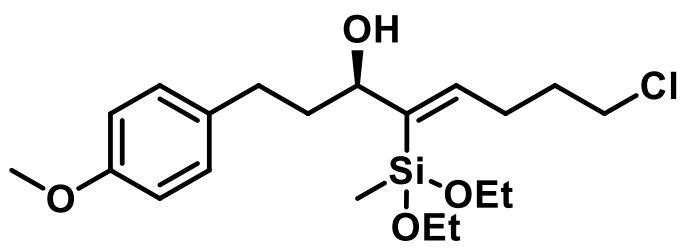

mg, 62%). $[\alpha]_D^{20}$= –0.5 (c = 1.07, $CHCl_3$). ^1H NMR (400 MHz, $CDCl_3$) δ 7.14 – 7.04 (m, 2H), 6.89 – 6.73 (m, 2H), 6.07 (td, *J* = 7.5, 0.9 Hz, 1H), 4.05 – 3.89 (m, 1H), 3.88 – 3.71 (m, 7H), 3.54 (t, *J* = 6.6 Hz, 2H), 3.39 (d, *J* = 10.0 Hz, 1H), 2.66 (ddd, *J* = 13.8, 10.2, 5.5 Hz, 1H), 2.55 (ddd, *J* = 13.9, 10.0, 6.2 Hz, 1H), 2.47 – 2.30 (m, 2H), 2.03 – 1.70 (m, 4H), 1.24 (td, *J* = 7.0, 5.0 Hz, 6H), 0.29 (s, 3H). ^{13}C NMR (101 MHz, $CDCl_3$) δ 157.8, 144.2, 140.4, 134.5, 129.5, 113.9, 79.4, 58.51, 58.49, 55.4, 44.5, 40.7, 32.7, 31.9, 28.9, 18.34, 18.30, –2.0. IR (film): $\tilde{\nu}$ 3493, 2972, 1927, 1612, 1512, 1442, 1390, 1366, 1299, 1246, 1177, 1165, 1102, 1074, 1038, 951, 821, 763 cm^{-1}. HRMS (ESI$^+$) calcd. for $C_{20}H_{33}O_4ClSiNa$ $[M+Na]^+$: 423.17288; found: 423.17318.

Compound S27. Prepared according to the general procedure **E** from compound **S17**; yellow oil (46

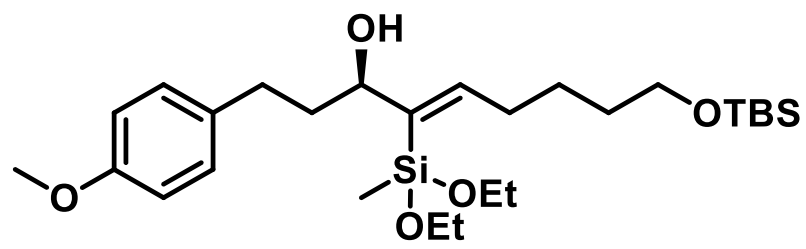

mg, 67%). $[\alpha]_D^{20}$= 10.1 (c = 0.48, $CHCl_3$). ^1H NMR (400 MHz, $CDCl_3$) δ 7.18 – 7.00 (m, 2H), 6.96 – 6.68 (m, 2H), 6.10 (td, *J* = 7.6, 0.9 Hz, 1H), 3.93 (tdd, *J* = 8.2, 6.8, 3.4 Hz, 1H), 3.88 – 3.72 (m, 7H), 3.60 (t, *J* = 6.3 Hz, 2H), 3.46 (d, *J* = 10.2 Hz, 1H), 2.66 (ddd, *J* = 13.8, 10.3, 5.6 Hz, 1H), 2.54 (ddd, *J* = 13.9, 10.1, 6.2 Hz, 1H), 2.23 (qd, *J* = 7.3, 5.0 Hz, 2H), 2.04 – 1.89 (m, 1H), 1.77 (ddt, *J* = 13.4, 10.3, 6.2 Hz, 1H), 1.56 – 1.49 (m, 2H), 1.48 – 1.37 (m, 2H), 1.24 (td, *J* = 7.0, 5.3 Hz, 6H), 0.88 (s, 9H), 0.27 (s, 3H), 0.04 (s, 6H). ^{13}C NMR (101 MHz, $CDCl_3$) δ 157.8, 146.6, 138.6, 134.6, 129.5, 113.9, 79.6, 63.1, 58.4, 55.4, 40.8, 32.8, 31.9, 31.5, 26.2, 26.1, 18.5, 18.35, 18.29, –1.9, –5.1. IR (film): $\tilde{\nu}$ 3504, 2953, 2929, 2858, 1613, 1512, 1463, 1442, 1390, 1361, 1247, 1176, 1165, 1101, 1076, 1041, 1006, 951, 836, 775 cm^{-1}. HRMS (ESI$^+$) calcd. for $C_{27}H_{50}O_5Si_2Na$ $[M+Na]^+$: 533.30890; found: 533.30937.

Compound S28. Prepared according to the general procedure **E** from compound **S18**; yellow oil (40.1

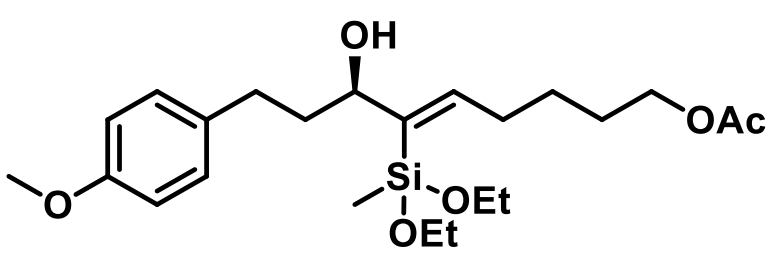

mg, 62%). $[\alpha]_D^{20}$= 3.7 (c = 0.46, $CHCl_3$). ^1H NMR (400 MHz, $CDCl_3$) δ 7.15 – 7.04 (m, 2H), 6.89 – 6.76 (m, 2H), 6.09 (td, *J* = 7.5, 0.9 Hz, 1H), 4.06 (t, *J* = 6.6 Hz, 2H), 4.00 – 3.88 (m, 1H), 3.88 – 3.70 (m, 7H), 3.42

(d, J = 10.1 Hz, 1H), 2.66 (ddd, J = 13.9, 10.3, 5.5 Hz, 1H), 2.54 (ddd, J = 13.9, 10.1, 6.2 Hz, 1H), 2.25 (ddt, J = 14.4, 12.7, 6.9 Hz, 2H), 2.04 (s, 3H), 2.01 – 1.89 (m, 1H), 1.77 (ddt, J = 13.4, 10.3, 6.1 Hz, 1H), 1.71 – 1.61 (m, 2H), 1.46 (dtd, J = 12.2, 5.3, 2.5 Hz, 2H), 1.24 (td, J = 7.0, 5.3 Hz, 6H), 0.27 (s, 3H). ^{13}C NMR (101 MHz, $CDCl_3$) δ 171.3, 157.8, 145.8, 139.3, 134.6, 129.5, 113.9, 79.5, 64.4, 58.4, 55.4, 40.8, 31.9, 31.2, 28.5, 26.2, 21.1, 18.34, 18.29, –1.9. IR (film): $\tilde{\nu}$ 3498, 2971, 2932, 1739, 1612, 1512, 1442, 1390, 1366, 1299, 1245, 1176, 1165, 1102, 1075, 1039, 952, 820, 763 cm^{-1}. HRMS (ESI$^+$) calcd. for $C_{23}H_{38}O_6SiNa$ $[M+Na]^+$: 461.23299; found: 461.23337.

Compound S29. Prepared according to the general procedure **E** from compound **S19**; yellow oil (151 mg, 66%). $[\alpha]_D^{20}$= 3.7 (c = 0.46, $CHCl_3$). ^{1}H NMR (400 MHz, $CDCl_3$) δ 7.17 – 7.03 (m, 2H), 6.94 – 6.74 (m, 2H), 5.96 (dd, J = 10.6, 0.8 Hz, 1H), 3.92 (dddd, J = 10.2, 7.4, 6.3, 0.9 Hz, 1H), 3.87 – 3.72 (m, 7H), 3.49 (d, J = 10.3 Hz, 1H), 2.77 (dddd, J = 16.0, 10.7, 8.6, 7.4 Hz, 1H), 2.65 (ddd, J = 13.8, 10.3, 5.7 Hz, 1H), 2.54 (ddd, J = 13.9, 10.1, 6.1 Hz, 1H), 1.97 (dddd, J = 13.4, 10.1, 7.7, 5.8 Hz, 1H), 1.89 – 1.71 (m, 3H), 1.71 – 1.62 (m, 2H), 1.61 – 1.50 (m, 2H), 1.30 – 1.14 (m, 8H), 0.28 (s, 3H). ^{13}C NMR (101 MHz, $CDCl_3$) δ 157.8, 151.8, 136.1, 134.6, 129.5, 113.9, 79.5, 58.5, 58.4, 55.4, 42.3, 40.8, 34.0, 33.6, 32.0, 25.8, 25.7, 18.35, 18.29, –1.5. IR (film): $\tilde{\nu}$ 3400, 2952, 2868, 1612, 1512, 1246, 1176, 1165, 1102, 1074, 1039, 947, 820, 803, 763 cm^{-1}. HRMS (ESI$^+$) calcd. for $C_{22}H_{36}O_4SiNa$ $[M+Na]^+$: 415.22751; found: 415.22781.

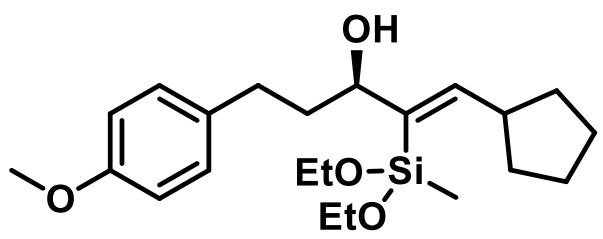

Compound S30. Prepared according to the general procedure **E** from compound **S20**; yellow oil (58.0 mg, 65%). $[\alpha]_D^{20}$= –53.8 (c = 0.70, $CHCl_3$). ^{1}H NMR (400 MHz, $CDCl_3$) δ 7.18 – 7.06 (m, 2H), 6.88 – 6.74 (m, 2H), 6.56 – 6.45 (m, 1H), 5.85 – 5.69 (m, 1H), 4.04 – 3.94 (m, 1H), 3.87 (d, J = 10.1 Hz, 1H), 3.81 – 3.75 (m, 7H), 2.67 (ddd, J = 13.8, 10.2, 5.7 Hz, 1H), 2.57 (ddd, J = 13.9, 10.0, 6.2 Hz, 1H), 2.12 – 2.04 (m, 2H), 2.04 – 1.90 (m, 3H), 1.91 – 1.74 (m, 1H), 1.67 – 1.52 (m, 4H), 1.30 – 1.18 (m, 6H), 0.25 (s, 3H). ^{13}C NMR (101 MHz, $CDCl_3$) δ 157.8, 148.6, 138.3, 136.9, 134.7, 129.5, 127.0, 113.9, 80.0, 58.5, 58.4, 55.4, 40.7, 32.0, 28.3, 25.6, 22.7, 22.0, 18.4, 18.3, –2.2. IR (film): $\tilde{\nu}$ 3401, 2971, 2927, 2835, 1774, 1724, 1612, 1512, 1442, 1390, 1299, 1245, 1166, 1102, 1069, 1036, 992, 931, 799, 761 cm^{-1}. HRMS (ESI$^+$) calcd. for $C_{23}H_{36}O_4SiNa$ $[M+Na]^+$: 427.22751; found: 427.22794.

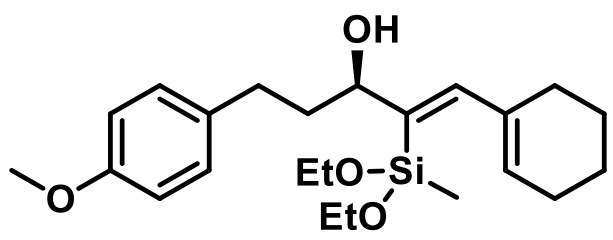

Compound S31. Prepared according to the general procedure **E** from compound **S21**; pale orange oil

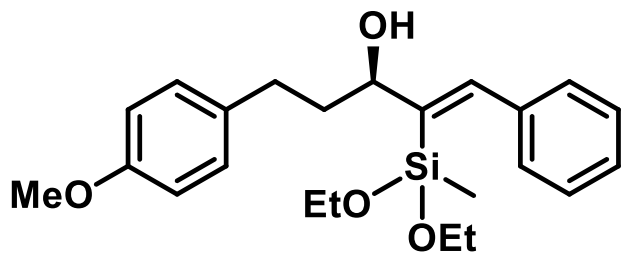

(219 mg, 49% yield, 96.7% ee). $[\alpha]_D^{20}$= –91.9 (c = 1.27, $CHCl_3$). ^1H NMR (400 MHz, $CDCl_3$) δ 7.39 – 7.37 (m, 2H), 7.33 – 7.28 (m, 3H), 7.25 (s, 1H), 7.16 – 7.14 (m, 2H), 6.85 – 6.83 (m, 2H), 4.17 (dd, *J* = 15.9, 8.0 Hz, 1H), 3.82 – 3.78 (m, 6H), 3.70 (dq, *J* = 10.2, 7.0 Hz, 1H), 3.61 (dq, *J* = 10.3, 7.0 Hz, 1H), 2.76 (ddd, *J* = 13.9, 10.0, 5.7 Hz, 1H), 2.66 (ddd, *J* = 13.9, 9.8,6.3 Hz, 1H), 2.09 (dddd, *J* = 13.6, 9.9, 8.0, 5.8 Hz, 1H), 1.95 (ddt, *J* = 13.5, 10.1, 6.2 Hz, 1H), 1.24 (t, *J* = 7.0 Hz, 3H), 1.11 (t, *J* = 7.0 Hz, 3H),0.05 (s, 3H). ^{13}C NMR (101 MHz, $CDCl_3$) δ 157.9, 145.4, 142.0, 138.4, 134.5, 129.5, 128.7, 128.1, 127.9, 113.9, 80.3, 58.6, 58.4, 55.4, 40.6, 32.0, 18.3, 18.1, -2.4. IR (neat): ν 3477, 2972, 2924, 2835, 1611, 1594, 1511, 1493, 1443, 1419, 1390, 1364, 1299, 1244, 1176, 1164, 1101, 1069, 1037, 945, 793, 754, 697, 573, 546, 512 cm^{-1}. HRMS (ESI^+) calcd. for $C_{23}H_{32}O_4SiNa$ $[M+Na]^+$: 423.19621; found: 423.19622.

MOP-MA-479-01 Racemat
150 mm Chiralpak IG-3, 3 µm, 4.6 mm i.D.
CH3CN / H2O = 55:45
1.0 ml/min / 262 bar / 298 K

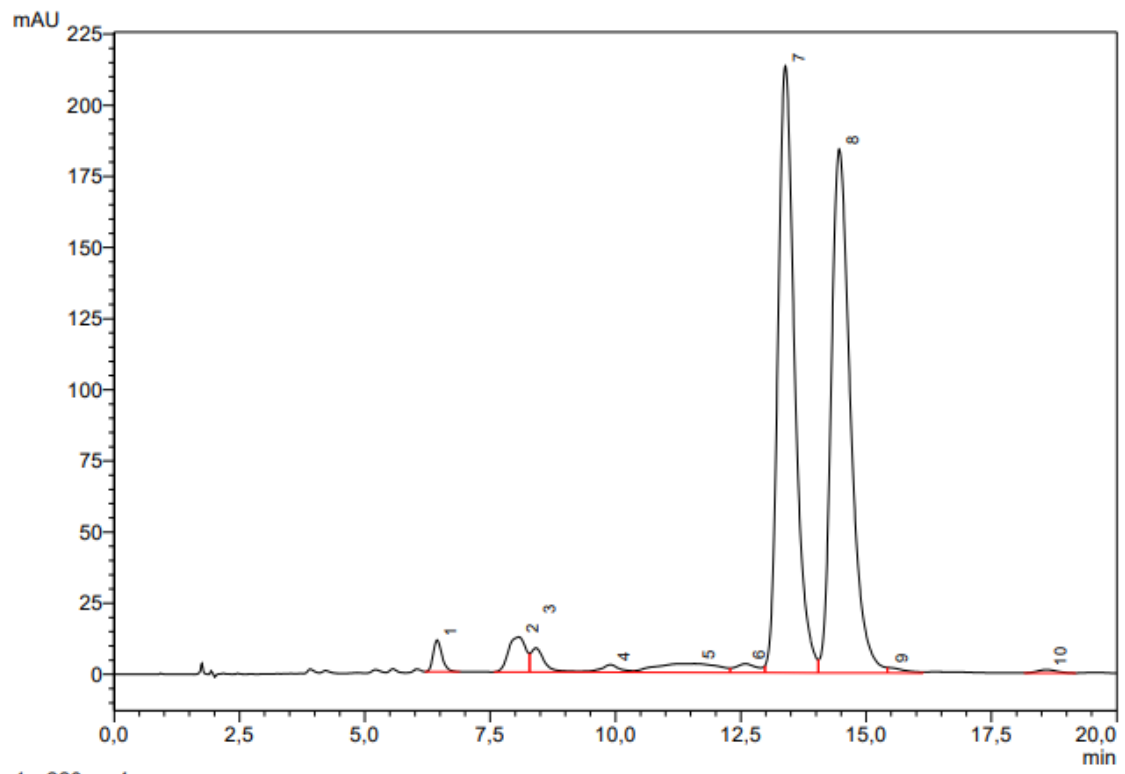

1 220nm,4nm

PDA Ch1 220nm

Peak #	Ret. Time	Area %	Name
1	6,44	1,25	
2	8,07	2,72	
3	8,42	1,43	
4	9,90	0,65	
5	11,59	2,44	
6	12,59	0,87	
7	13,39	45,21	1. Enantiomer
8	14,47	44,76	2. Enantiomer
9	15,44	0,39	
10	18,61	0,29	
Total		100,00	

MOP-MA-480-01
150 mm Chiralpak IG-3, 3 µm, 4.6 mm i.D.
CH3CN / H2O = 55:45
1.0 ml/min / 262 bar / 298 K

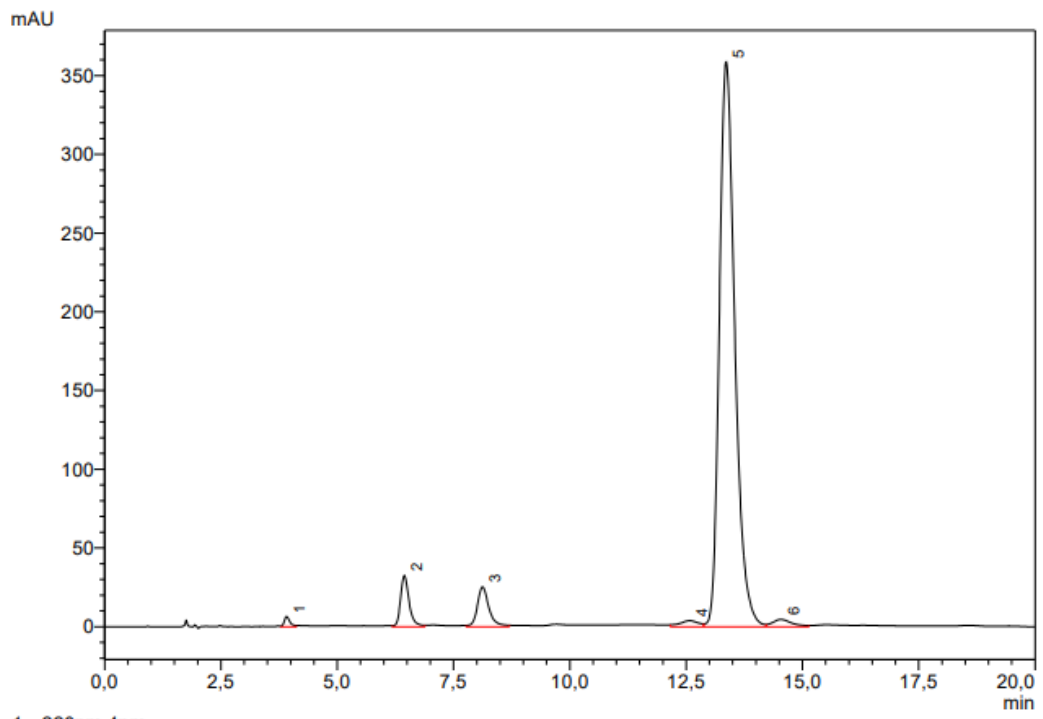

1 220nm,4nm

PDA Ch1 220nm

Peak #	Ret. Time	Area %	Name
1	3,91	0,59	
2	6,44	4,44	
3	8,12	4,67	
4	12,57	1,16	
5	13,36	87,68	1. Enantiomer 96.7 % ee
6	14,54	1,47	2. Enantiomer
Total		100,00	

Compound S32. Prepared according to the general procedure **E** from compound **S22**; yellow oil (65.2

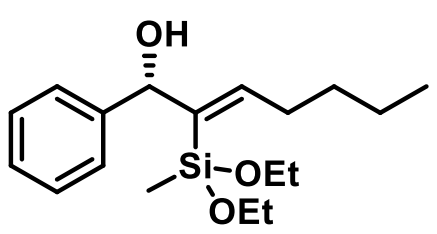

mg, 76%). $[\alpha]_D^{20}$= –39.7 (c = 0.94, $CHCl_3$). ^1H NMR (400 MHz, $CDCl_3$) δ 7.41 – 7.27 (m, 4H), 7.22 – 7.15 (m, 1H), 6.28 (td, *J* = 7.6, 0.9 Hz, 1H), 5.14 (d, *J* = 10.0 Hz, 1H), 4.16 (d, *J* = 10.0 Hz, 1H), 3.86 – 3.63 (m, 2H), 3.63 – 3.25 (m, 2H), 2.29 (qd, *J* = 7.4, 1.2 Hz, 2H), 1.52 – 1.28 (m, 4H), 1.21 (t, *J* = 7.0 Hz, 3H), 1.07 (t, *J* = 7.0 Hz, 3H), 0.93 (t, *J* = 7.1 Hz, 3H), 0.01 (s, 3H). ^{13}C NMR (101 MHz, CDCl3) δ 148.0, 144.6, 138.1, 127.9, 126.6, 126.1, 80.8, 58.4, 32.0, 31.5, 22.7, 18.2, 18.1, 14.2, –2.3. IR (film): $\tilde{\nu}$ 3478, 2969, 2925, 2875, 1613, 1492, 1448, 1390, 1258, 1164, 1101, 1073, 1013, 949, 823, 802, 766, 700 cm^{-1}. HRMS (ESI^+) calcd. for $C_{18}H_{30}O_3SiNa$ $[M+Na]^+$: 345.18564; found: 345.18549.

Compound S33. Prepared according to the general procedure **E** from compound **S23**; yellow oil (55.1 mg, 72%). ^{1}H NMR (400 MHz, $CDCl_3$) δ 7.14 – 7.03 (m, 2H), 6.88 – 6.77 (m, 2H), 6.15 (t, *J* = 7.6 Hz, 1H), 4.39 (s, 1H), 3.95 – 3.68 (m, 7H), 2.65 – 2.45 (m, 2H), 2.35 – 2.16 (m, 2H), 1.96 (ddd, *J* = 13.5, 12.0, 5.2 Hz, 1H), 1.83 (ddd, *J* = 13.5, 12.2, 5.4 Hz, 1H), 1.45 – 1.30 (m, 7H), 1.24 (t, *J* = 7.0 Hz, 6H), 0.99 – 0.87 (m, 3H), 0.30 (s, 3H). ^{13}C NMR (101 MHz, $CDCl_3$) δ 157.7, 143.9, 142.0, 135.3, 129.3, 113.9, 76.3, 58.4, 55.4, 45.2, 32.3, 31.3, 30.3, 27.5, 22.7, 18.3, 18.2, 14.2, –1.6. IR (film): $\tilde{\nu}$ 3364 (br), 2952, 2928, 2857, 1739, 1514, 1462, 1441, 1362, 1326, 1250, 1081, 837, 777 cm^{-1}. HRMS (ESI$^+$) calcd. for $C_{22}H_{38}O_4SiNa$ [M+Na]$^+$: 417.24316; found: 417.24353.

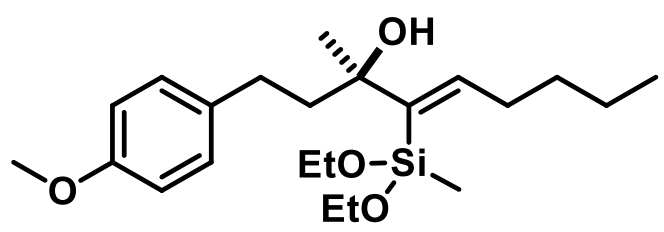

Compound S34. Prepared according to the general procedure **E** from the corresponding macrocyclic propargyl alcohol; yellow oil (52.0 mg, 74%). ^{1}H NMR (400 MHz, $CDCl_3$) δ 6.06 – 5.93 (m, 1H), 4.17 – 3.94 (m, 2H), 3.91 – 3.66 (m, 5H), 2.42 – 2.21 (m, 4H), 1.80 – 1.67 (m, 2H), 1.65 – 1.52 (m, 4H), 1.50 – 1.18 (m, 12H), 1.17 – 1.03 (m, 2H), 0.27 (s, 3H). ^{13}C NMR (101 MHz, $CDCl_3$) δ 174.7, 148.8, 138.1, 81.4, 64.0, 58.4, 38.3, 35.2, 31.1, 29.1, 27.62, 27.58, 26.7, 26.5, 26.1, 18.3, 18.2, –2.0. IR (film): $\tilde{\nu}$ 3506, 2970, 2928, 2859, 1731, 1613, 1461, 1440, 1389, 1365, 1339, 1257, 1226, 1167, 1151, 1101, 1074, 948, 819, 792, 761 cm^{-1}. HRMS (ESI$^+$) calcd. for $C_{19}H_{36}O_5SiNa$ [M+Na]$^+$: 395.22242; found: 395.22222.

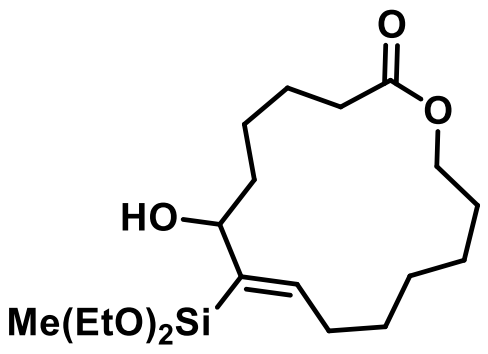

Allenes

Larger Scale Synthesis of Compound (+)-2. A solution of allylic alcohol **1** (500 mg, 1.31 mmol; 98.1% ee) in THF (13 mL) was cooled to 0 °C (bath temperature) in a flame-dried Schlenk flask. A solution of LDA (freshly prepared from diisopropylamine (0.29 mL, 2.03 mmol) and *n*BuLi (1.6 M in hexane, 1.25 mL, 2.0 mmol) in THF (18.5 mL)) was added, followed by triethylamine (1.3 mL) and then CuI (50 mg, 0.26 mmol). The mixture was stirred at 0 °C under argon for 2 h before saturated aqueous ammonium chloride solution (20 mL) was added, and the reaction was diluted with ethyl acetate and water. The organic layer was separated, and the aqueous phase was extracted with ethyl acetate (3 x 20 mL). The combined organic extracts were washed with brine, dried over anhydrous magnesium sulfate, and filtered. The filtrate was concentrated under reduced pressure and the residue was purified *via* flash chromatography (0-5% *tert*-butyl methyl ether/cyclohexane)to give the title compound as a yellow oil (271 mg, 90%). The e.e. was found to be 96.2% (see below for the HPLC data using a chiral stationary phase). $[\alpha]_D^{20}$= 40.8 (c = 0.75, $CHCl_3$). ^1H NMR (400 MHz, $CDCl_3$) δ 7.21 – 7.04 (m, 2H), 6.91 – 6.69 (m, 2H), 5.16 – 5.03 (m, 2H), 3.79 (s, 3H), 2.66 (t, *J* = 7.8 Hz, 2H), 2.32 – 2.19 (m, 2H), 2.08 – 1.86 (m, 2H), 1.47 – 1.27 (m, 4H), 0.90 (t, *J* = 6.9 Hz, 3H). ^{13}C NMR (101 MHz, $CDCl_3$) δ 204.1, 157.9, 134.2, 129.5, 113.8, 91.5, 90.4, 55.4, 34.7, 31.5, 31.1, 28.7, 22.3, 14.1. IR (film): $\tilde{\nu}$ 2955, 2927, 2855, 1962, 1612, 1584, 1512, 1455, 1300, 1244, 1176, 1106, 1039, 821 cm^{-1}. HRMS (EI^+) calcd. for $C_{16}H_{22}O$ $[M]^+$: 230.16652; found: 230.16648.

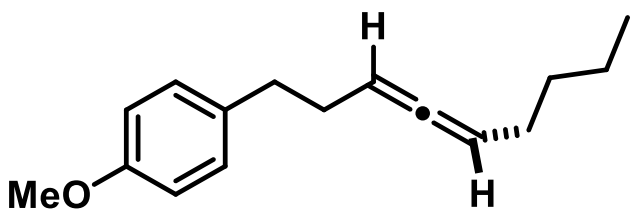

The enantiomer, *i. e*. compound (–)-**2**, was prepared using the method reported by Myers and coworker.[6] This sample exhibited an optical rotation of $[\alpha]_D^{20}$= –49.8 (c = 0.26, $CHCl_3$), and the e.e. was found to be 97.8% (see below for the HPLC data using a chiral stationary phase). All other data were identical. The two samples were confirmed to be of opposite absolute configuration by a comparison of the retention times. Further, mixture of the two samples gave an HPLC trace wherein both enantiomers were clearly visible and of approximately equal intensity.

SUJ-SA-332 Racemat
150 mm Chiralpak IB-N-3, 3 µm, 4.6 mm i.D.
n-Heptan / 2-Propanol = 99.96:0.04 (v/v)
1.0 ml/min / 8.7 MPa / 293 K

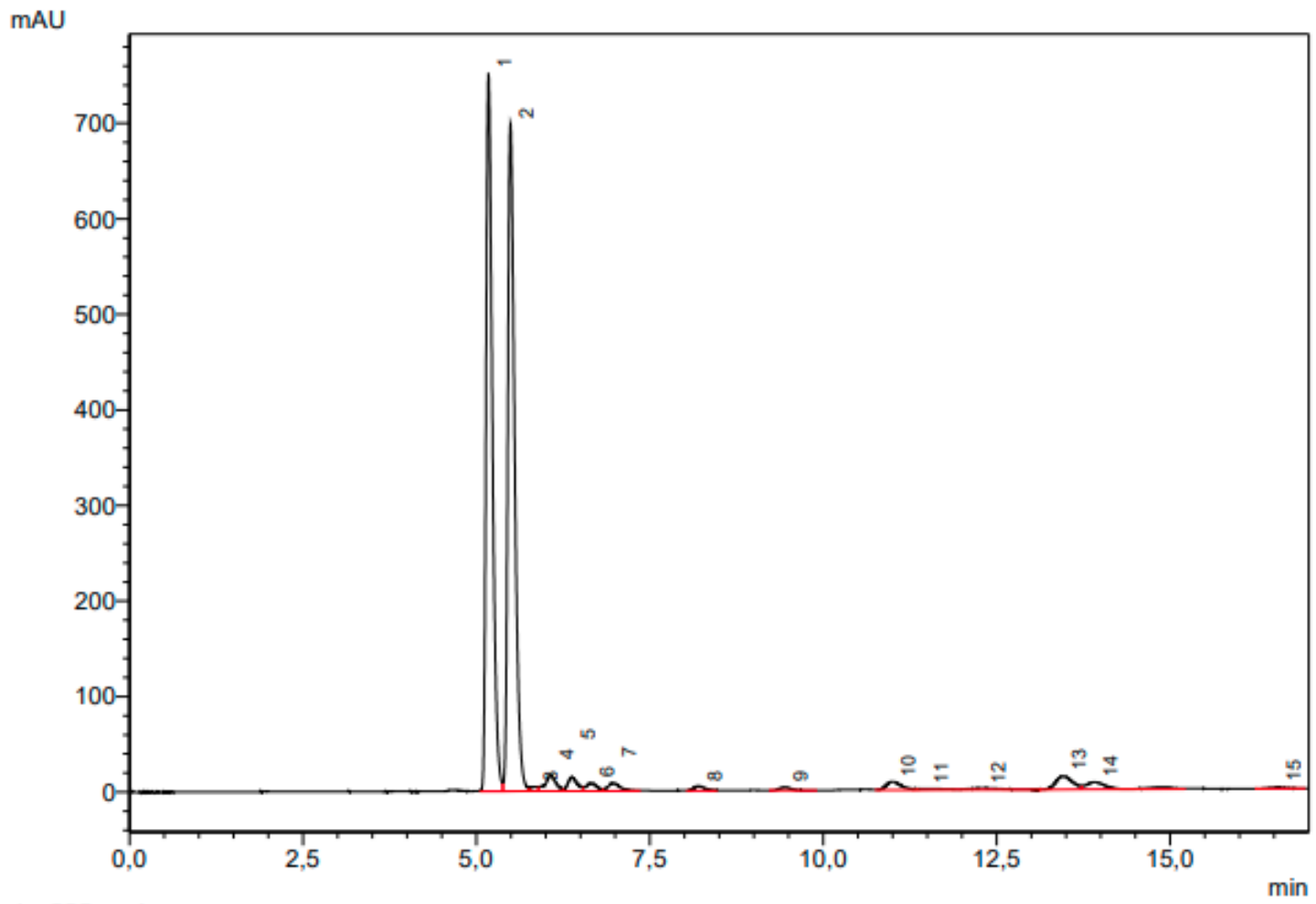

1 225nm,4nm

PDA Ch1 225nm

Peak #	Ret. Time	Area %	Name
1	5,18	43,61	1. Enantiomer
2	5,49	44,21	2. Enantiomer
3	5,84	0,30	
4	6,07	1,63	
5	6,38	1,27	
6	6,66	0,82	
7	6,97	0,96	
8	8,21	0,50	
9	9,44	0,34	
10	11,00	1,27	
11	11,47	0,33	
12	12,30	0,51	
13	13,46	2,16	
14	13,91	1,81	
15	16,56	0,26	
Total		100,00	

SUJ-SA-562-01
150 mm Chiralpak IB-N-3, 3 µm, 4.6 mm i.D.
n-Heptan / 2-Propanol = 99.96:0.04 (v/v)
1.0 ml/min / 8.7 MPa / 293 K

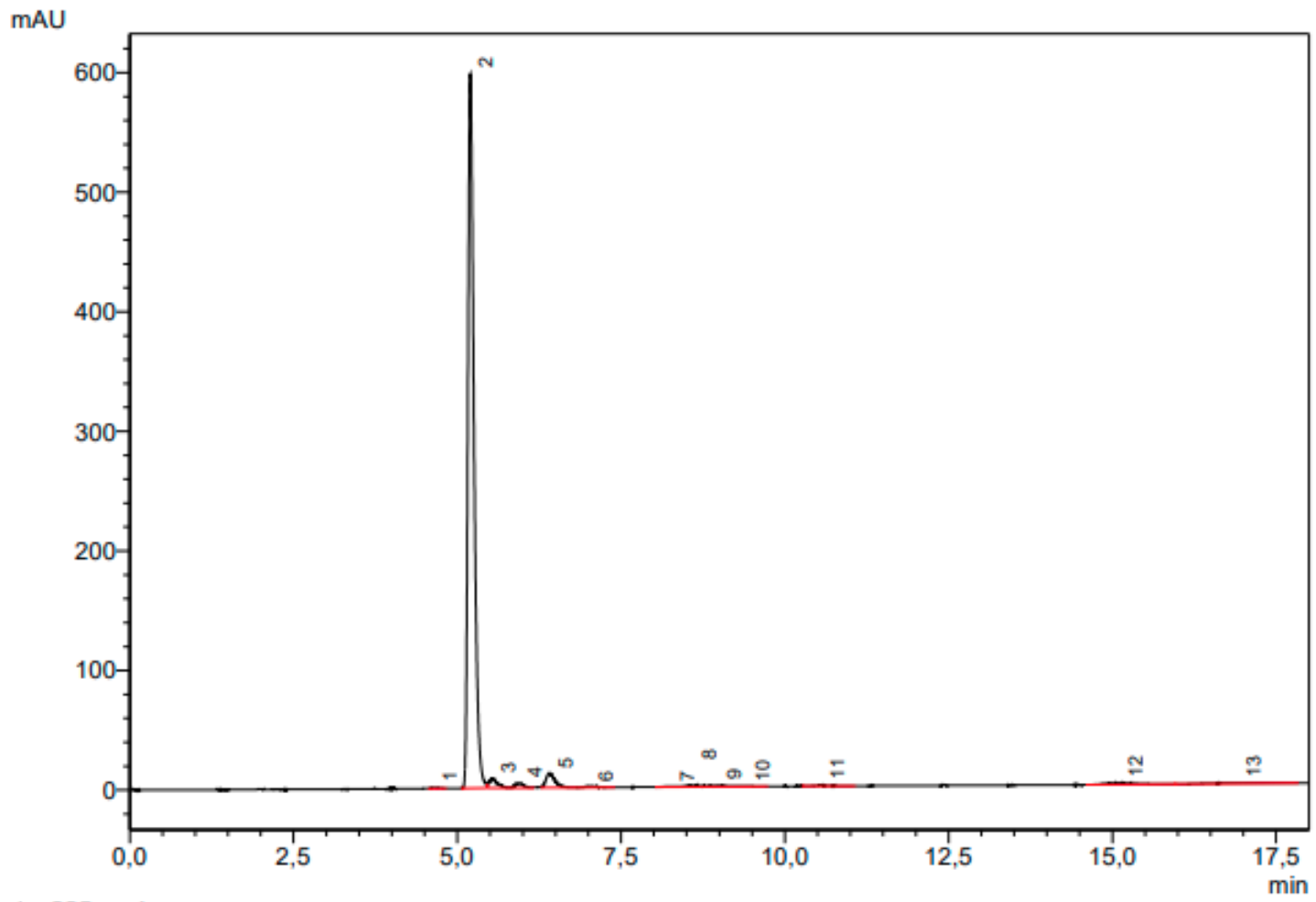

1 225nm,4nm

PDA Ch1 225nm

Peak #	Ret. Time	Area %	Name
1	4,64	0,16	
2	5,20	90,69	1. Enantiomer 96.2 % ee
3	5,54	1,74	2. Enantiomer
4	5,94	0,92	
5	6,41	2,85	
6	7,02	0,39	
7	8,26	0,19	
8	8,60	0,53	
9	8,98	0,39	
10	9,42	0,16	
11	10,57	0,20	
12	15,11	1,15	
13	16,91	0,62	
Total		100,00	

Data for compound (–)-**2** prepared using the method of Myers and coworker:[6]

SUJ-SA-566-01
150 mm Chiralpak IB-N-3, 3 µm, 4.6 mm i.D.
n-Heptan / 2-Propanol = 99.96:0.04 (v/v)
1.0 ml/min / 8.7 MPa / 293 K

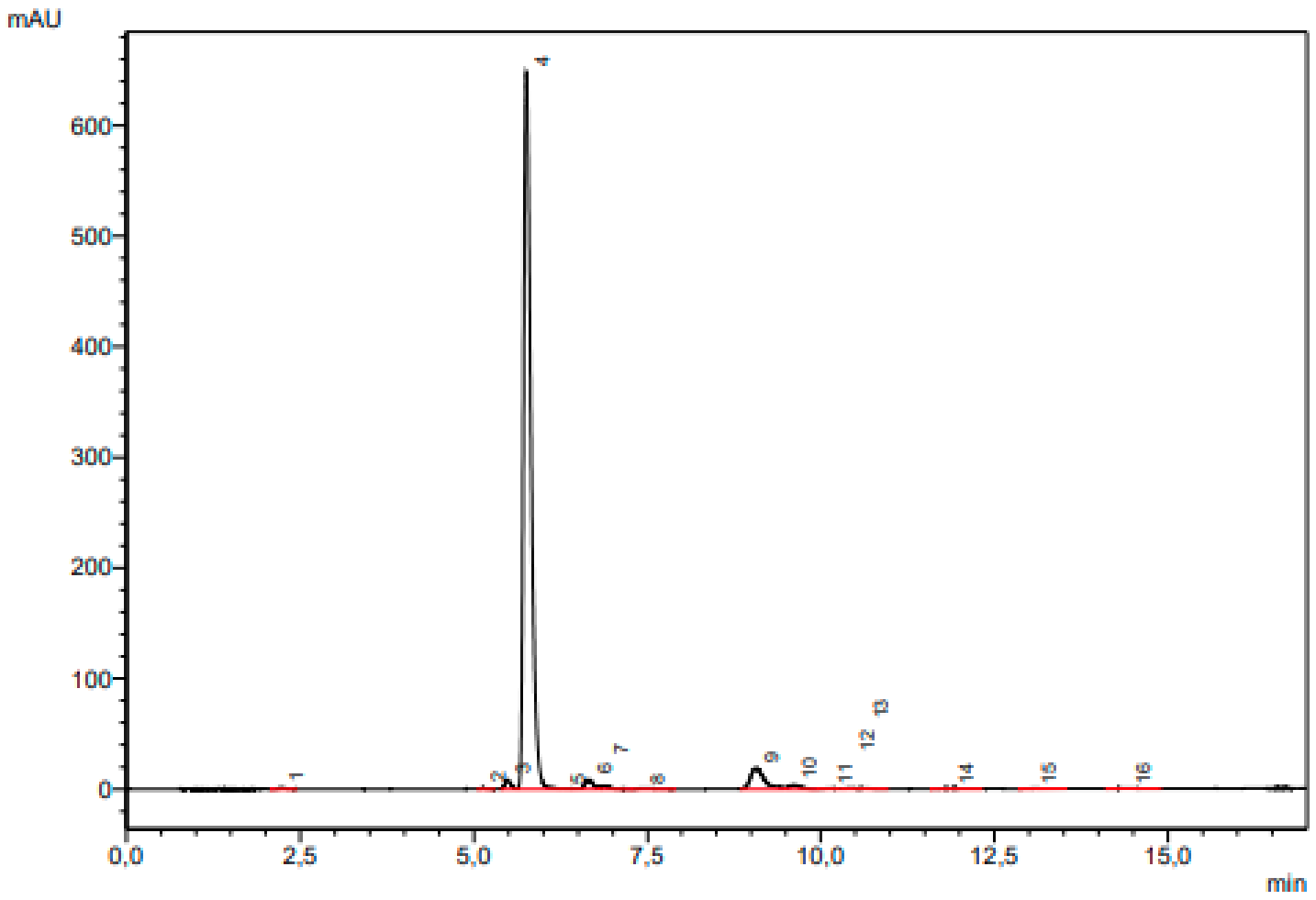

1 225nm,4nm

PDA Ch1 225nm

Peak #	Ret. Time	Area %	Name
1	2,22	0,17	
2	5,12	0,11	
3	5,48	1,01	1. Enantiomer
4	5,75	89,49	2. Enantiomer 97.8 % ee
5	6,28	0,15	
6	6,65	1,31	
7	6,89	0,51	
8	7,42	0,30	
9	9,07	5,05	
10	9,62	0,88	
11	10,13	0,15	
12	10,45	0,19	
13	10,64	0,16	
14	11,87	0,27	
15	13,06	0,12	
16	14,42	0,13	
Total		100,00	

SUJ-SA-562-01 u. SUJ-SA-566-01
150 mm Chiralpak IB-N-3, 3 µm, 4.6 mm i.D.
n-Heptan / 2-Propanol = 99.96:0.04 (v/v)
1.0 ml/min / 8.7 MPa / 293 K

Trace from mixture of the samples:

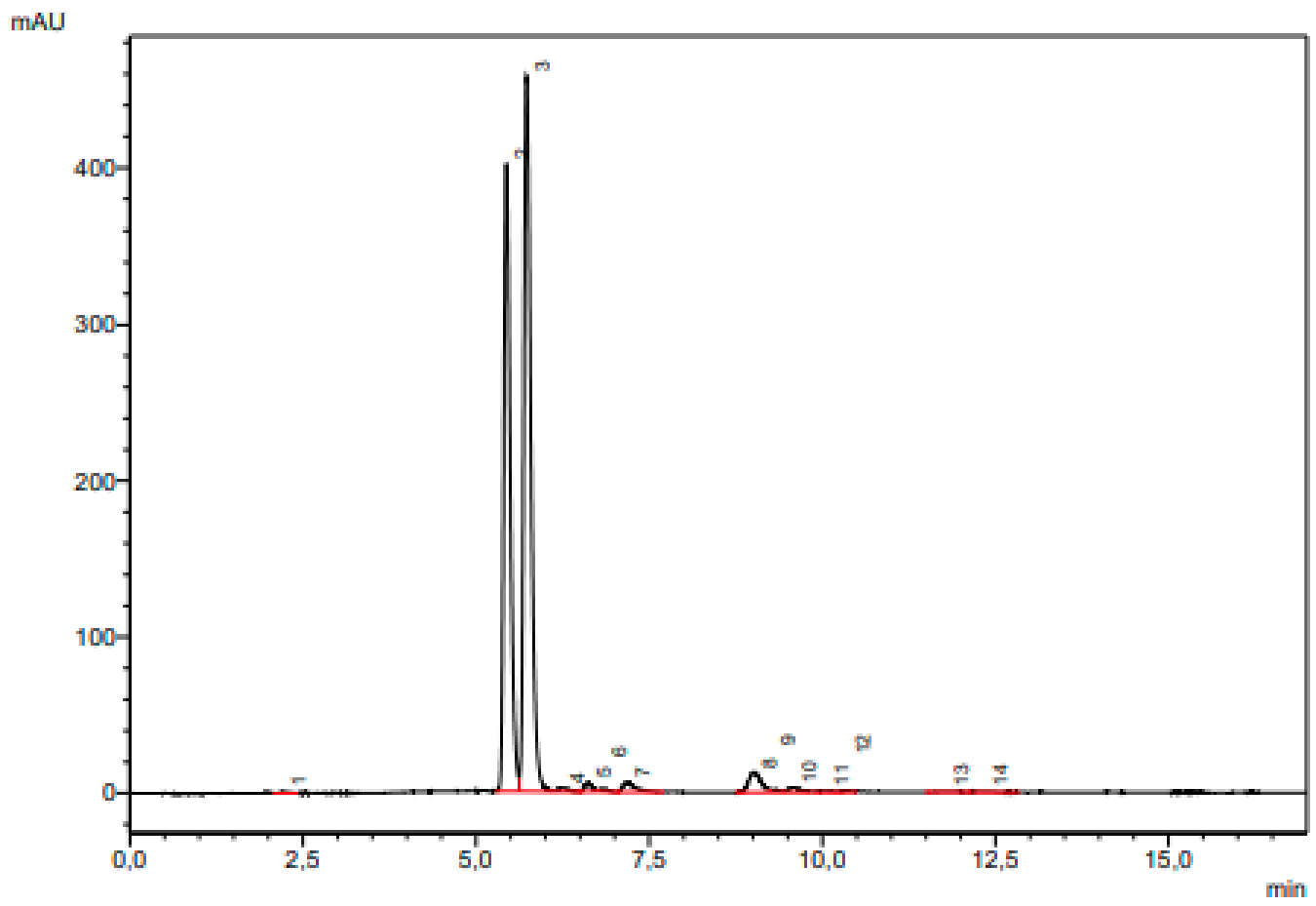

1 225nm,4nm

PDA Ch1 225nm

Peak #	Ret. Time	Area %	Name
1	2,22	0,15	
2	5,44	41,27	1. Enantiomer
3	5,73	51,89	2. Enantiomer
4	6,24	0,41	
5	6,62	0,72	
6	6,84	0,26	
7	7,18	1,23	
8	9,01	2,55	
9	9,28	0,24	
10	9,59	0,63	
11	10,06	0,21	
12	10,37	0,19	
13	11,78	0,12	
14	12,34	0,14	
Total		100,00	

Compound 5. Prepared according to the general procedure **F** from compound **S24**; colourless oil (11.8 mg, 97%). The e.e. was found to be 94.8% (see below for the HPLC data using a chiral stationary phase). $[\alpha]_D^{20}$= 2.4 (c = 0.25, $CHCl_3$). ^{1}H NMR (400 MHz, $CDCl_3$) δ 7.25 – 7.21 (m, 2H), 7.15 – 7.08 (m, 2H), 5.19 – 4.95 (m, 2H), 2.68 (t, *J* = 7.4 Hz, 2H), 2.39 – 2.19 (m, 2H), 2.00 – 1.85 (m, 2H), 1.37 – 1.27 (m, 4H), 0.96 – 0.78 (m, 3H). ^{13}C NMR (101 MHz, $CDCl_3$) δ 204.2, 140.5, 131.6, 130.0, 128.5, 91.8, 90.0, 34.9, 31.4, 30.7, 28.7, 22.3, 14.1. IR (film): $\tilde{\nu}$ 2957, 2927, 2857, 1962, 1492, 1454, 1407, 1378, 1092, 1016, 870, 832, 808 cm^{-1}. HRMS (EI^+) calcd. for $C_{15}H_{19}Cl$ $[M]^+$: 234.11698; found: 234.11697.

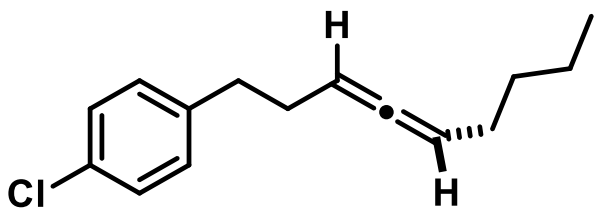

SUJ-SA-442-01 Racemat
150 mm Chiralpak IG-3, 3 µm, 4.6 mm i.D.
CH3CN / H2O = 60:40
1.0 ml/min, 114 bar, 298 K

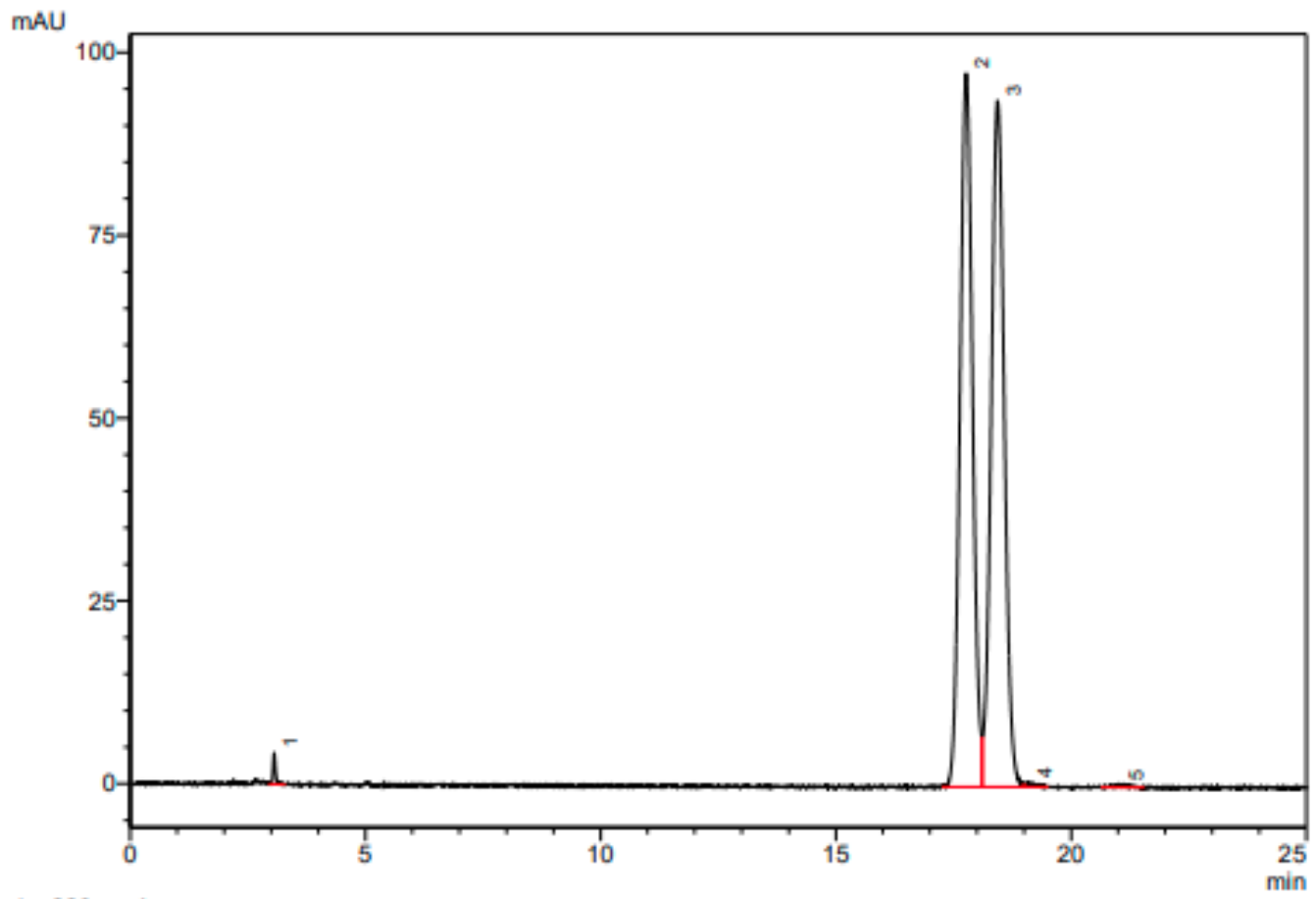

1 220nm,4nm

PDA Ch1 220nm

Peak #	Ret. Time	Area %	Name
1	3,06	0,41	
2	17,77	49,39	1. Enantiomer
3	18,44	49,86	2. Enantiomer
4	19,11	0,20	
5	21,05	0,14	
Total		100,00	

SUJ-SA-446-01
150 mm Chiralpak IG-3, 3 µm, 4.6 mm i.D.
CH3CN / H2O = 60:40
1.0 ml/min, 114 bar, 298 K

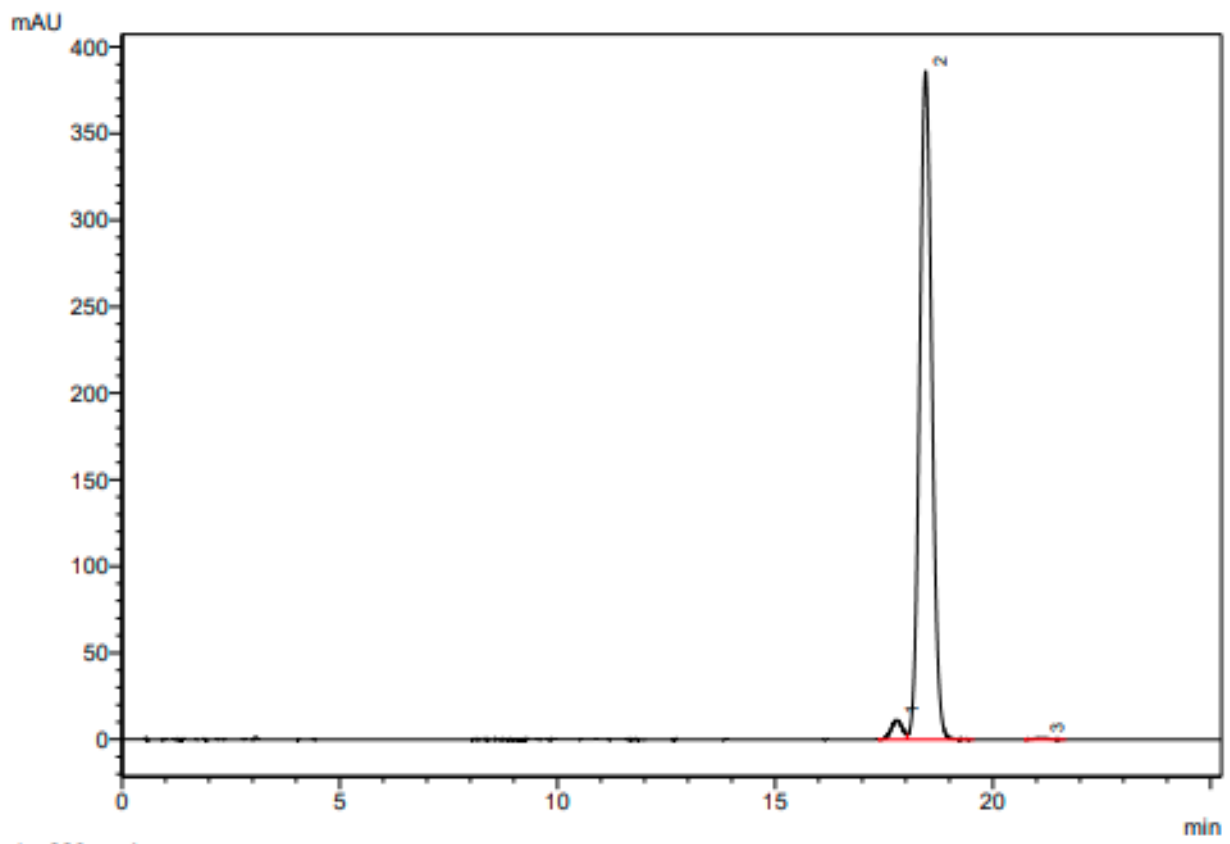

1 220nm,4nm

PDA Ch1 220nm

Peak #	Ret. Time	Area %	Name
1	17,79	2,61	1. Enantiomer
2	18,45	97,15	2. Enantiomer 94.8 % ee
3	21,14	0,25	
Total		100,00	

Compound 6. Prepared according to the general procedure **F** from compound **S25**; colourless oil (9.7 mg, 81%). The e.e. was found to be 94.1% (see below for the HPLC data using a chiral stationary phase). $[\alpha]_D^{20}$= 0.4 (c = 0.24, $CHCl_3$). ^1H NMR (600 MHz, $CDCl_3$) δ 7.64 – 7.54 (m, 2H), 7.31 – 7.27 (m, 2H), 5.08 (tt, J = 5.3, 4.3 Hz, 2H), 2.78 (td, J = 7.6, 1.1 Hz, 2H), 2.40 – 2.23 (m, 2H), 1.96 – 1.86 (m, 2H), 1.40 – 1.26 (m, 4H), 0.94 – 0.80 (m, 3H). ^{13}C NMR (151 MHz, $CDCl_3$) δ 204.3, 147.8, 132.2, 129.5, 119.3, 109.8, 92.2, 89.6, 35.5, 31.4, 30.1, 28.6, 22.3, 14.0. IR (film): $\tilde{\nu}$ 2957, 2928, 2856, 2228, 1963, 1608, 1505, 1455, 1440, 1415, 1289, 1177, 1106, 1021, 871, 841, 822, 777 cm^{-1}. HRMS (EI^+) calcd. for $C_{16}H_{19}N$ $[M]^+$: 225.15120; found: 225.15106.

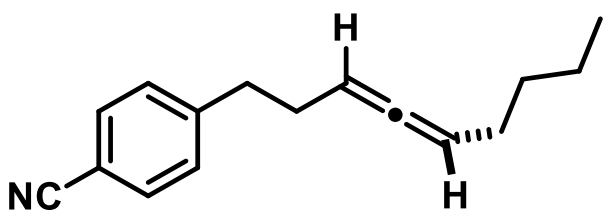

SUJ-SA-443-01 Racemat
150 mm Chiralpak IG-3, 3 µm, 4.6 mm i.D.
CH3OH/H2O = 85:15
0.5 ml/min, 9.0 MPa, 293 K

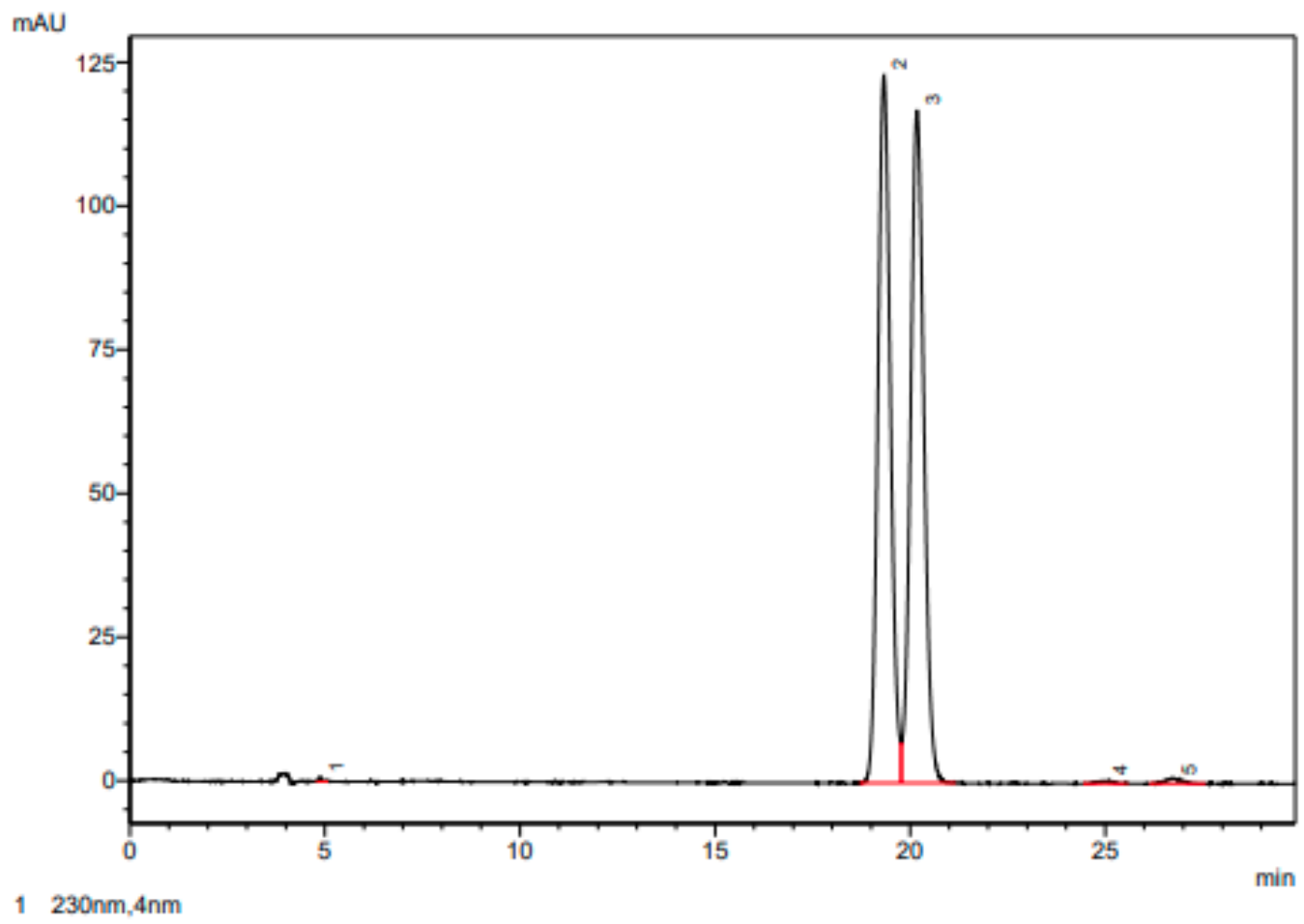

PDA Ch1 230nm

Peak #	Ret. Time	Area %	Name
1	4,89	0,07	
2	19,33	49,37	1. Enantiomer
3	20,18	49,86	2. Enantiomer
4	24,99	0,22	
5	26,76	0,48	
Total		100,00	

SUJ-SA-447-01
150 mm Chiralpak IG-3, 3 µm, 4.6 mm i.D.
CH3OH/H2O = 85:15
0.5 ml/min, 9.0 MPa, 293 K

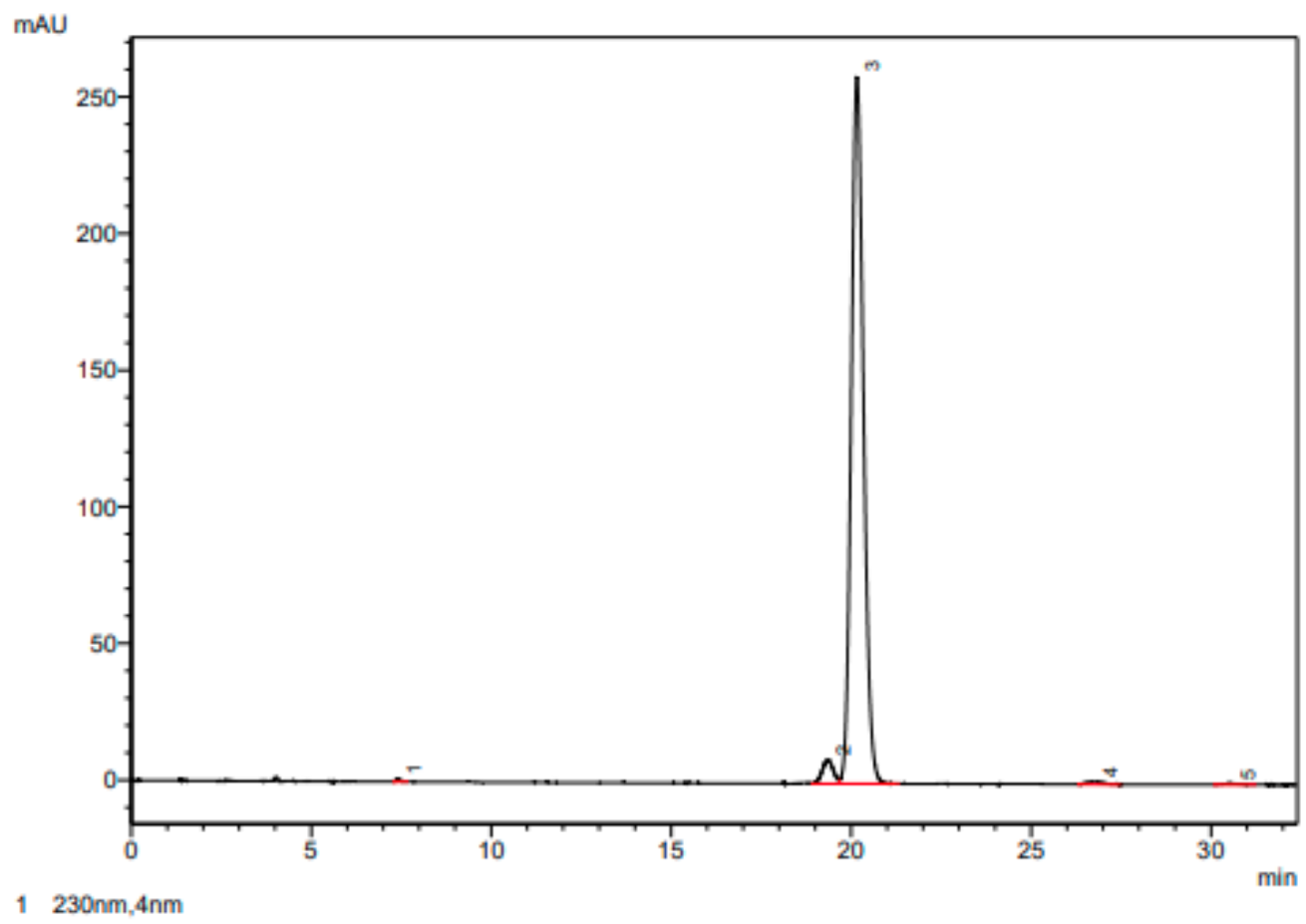

PDA Ch1 230nm

Peak #	Ret. Time	Area %	Name
1	7,42	0,14	
2	19,35	2,94	1. Enantiomer
3	20,16	96,31	2. Enantiomer 94.1 % ee
4	26,77	0,46	
5	30,60	0,15	
Total		100,00	

Compound 7. Prepared according to the general procedure **F** from compound **S26**; colourless oil (11.8 mg, 94%, contaminated by ≈5% protodecupration product as observed by NMR). The e.e. was found to be 92.8% (see below for the HPLC data using a chiral stationary phase). $[\alpha]_D^{20}$= 36.6 (c = 0.32, $CHCl_3$). ^1H NMR (400 MHz, $CDCl_3$) δ 7.15 – 7.03 (m, 2H), 6.87 – 6.70 (m, 2H), 5.15 (ddq, *J* = 9.6, 6.5, 3.1 Hz, 1H), 5.08 (qt, *J* = 6.3, 3.0 Hz, 1H), 3.79 (s, 3H), 3.54 (t, *J* = 6.6 Hz, 2H), 2.77 – 2.61 (m, 2H), 2.38 – 2.23 (m, 2H), 2.16 – 2.02 (m, 2H), 1.81 (dtd, *J* = 8.2, 6.6, 1.3 Hz, 2H). ^{13}C NMR (101 MHz, $CDCl_3$) δ 204.3, 157.9, 134.0, 129.5, 113.8, 91.3, 89.9, 55.4, 44.5, 34.7, 31.8, 31.0, 26.0. IR (film): $\tilde{\nu}$ 2928, 2853, 1962, 1612, 1583, 1511, 1441, 1300, 1245, 1177, 1106, 1076, 1037, 956, 873, 822, 764 cm^{-1}. HRMS (EI^+) calcd. for $C_{15}H_{19}OCl$ $[M]^+$: 250.11189; found: 250.11187.

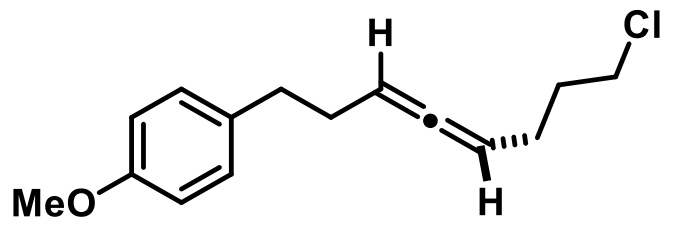

SUJ-SA-539-01 Racemat
150 mm Chiralpak IB-N-3, 3 µm, 4.6 mm i.D.
n-Heptan / 2-Propanol = 99.95:0.05 (v/v)
1.0 ml/min / 9.8 MPa / 293 K

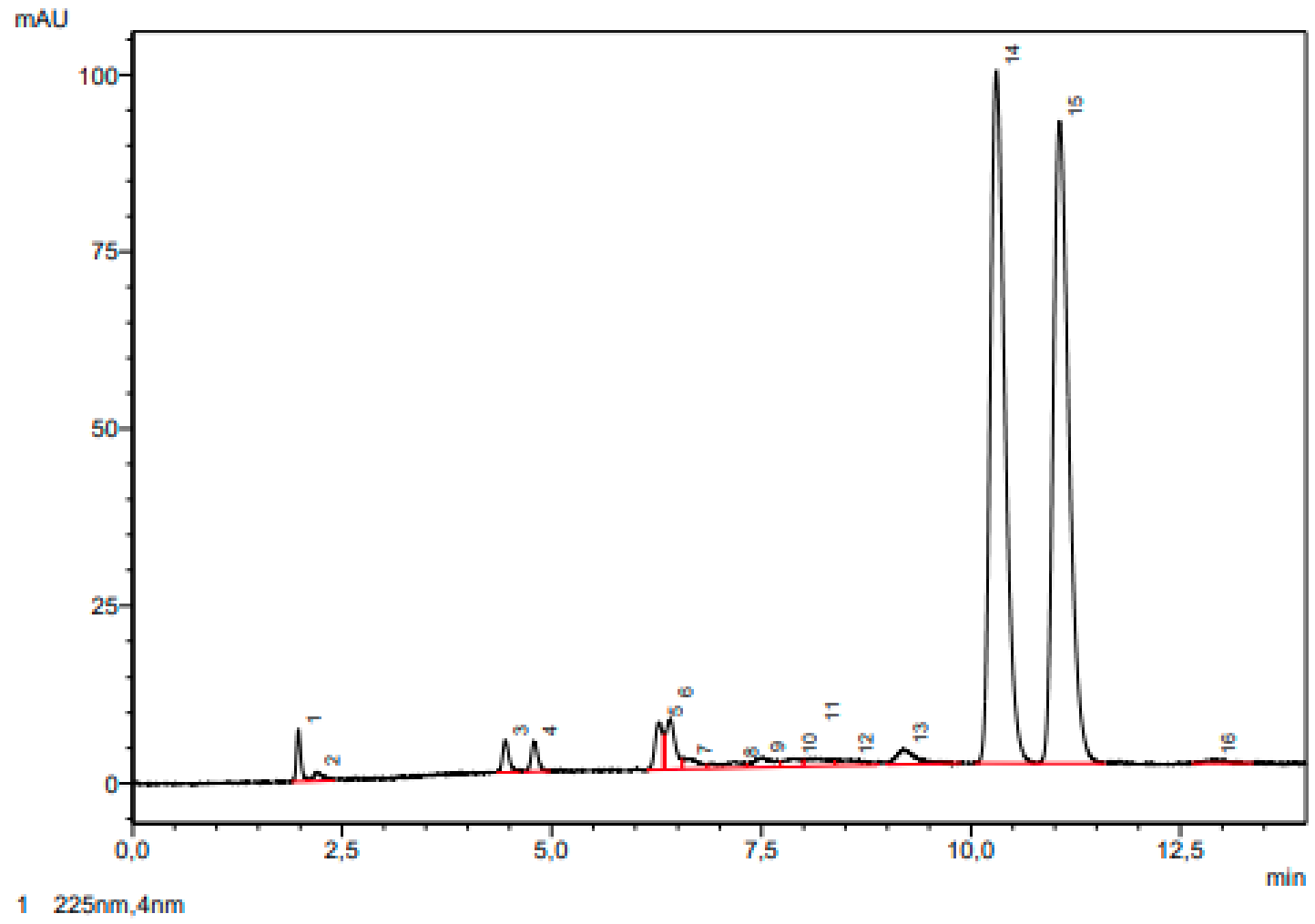

PDA Ch1 225nm

Peak #	Ret. Time	Area %	Name
1	1,98	1,01	
2	2,20	0,41	
3	4,45	0,89	
4	4,80	0,88	
5	6,28	1,56	
6	6,40	2,01	
7	6,63	0,72	
8	7,19	0,68	
9	7,50	0,84	
10	7,88	0,63	
11	8,15	0,78	
12	8,55	0,69	
13	9,20	1,31	
14	10,30	43,59	1. Enantiomer
15	11,06	43,70	2. Enantiomer
16	12,89	0,31	
Total		100,00	

SUJ-SA-545-01
150 mm Chiralpak IB-N-3, 3 µm, 4.6 mm i.D.
n-Heptan / 2-Propanol = 99.95:0.05 (v/v)
1.0 ml/min / 9.8 MPa / 293 K

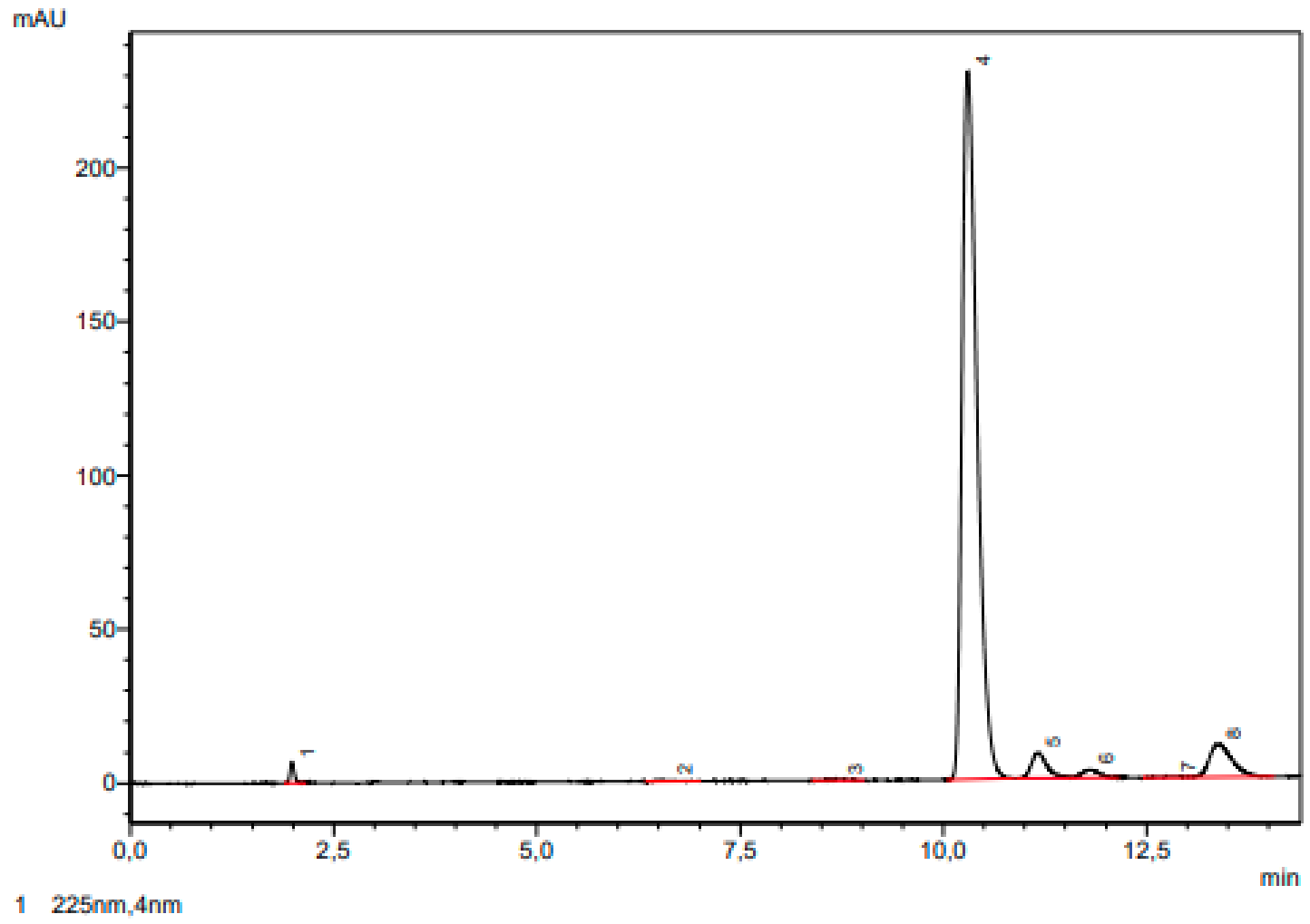

PDA Ch1 225nm

Peak #	Ret. Time	Area %	Name
1	1,98	0,75	
2	6,62	0,13	
3	8,73	0,19	
4	10,30	87,95	1. Enantiomer 92.8 % ee
5	11,17	3,30	2. Enantiomer
6	11,82	1,12	
7	12,82	0,21	
8	13,38	6,35	
Total		100,00	

Compound 8. Prepared according to the general procedure **F** from compound **S27**; colourless oil (14.4

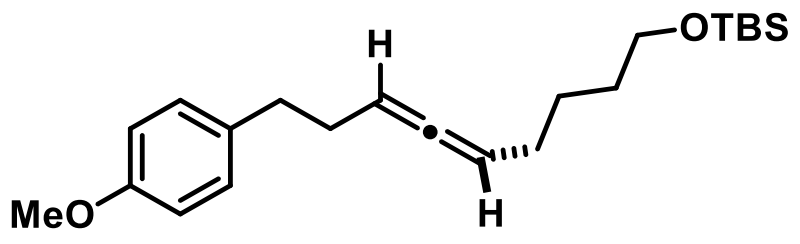

mg, 82%). The e.e. was found to be 96.2% (see below for the HPLC data using a chiral stationary phase). $[\alpha]_D^{20}$= –18.0 (c = 0.25, $CHCl_3$). ^1H NMR (400 MHz, $CDCl_3$) δ 7.16 – 7.06 (m, 2H), 6.91 – 6.77 (m, 2H), 5.21 – 4.98 (m, 2H), 3.79 (s, 3H), 3.60 (t, *J* = 6.5 Hz, 2H), 2.75 – 2.57 (m, 2H), 2.35 – 2.17 (m, 2H), 1.97 (tdd, *J* = 9.7, 5.0, 2.0 Hz, 2H), 1.56 – 1.49 (m, 1H), 1.47 – 1.37 (m, 2H), 0.89 (s, 10H), 0.05 (s, 6H). ^{13}C NMR (101 MHz, $CDCl_3$) δ 204.1, 157.9, 134.2, 129.5, 113.8, 91.4, 90.5, 63.2, 55.4, 34.7, 32.5, 31.1, 28.8, 26.1, 25.6, 18.5, –5.1. IR (film): $\tilde{\nu}$ 2928, 2855, 1612, 1512, 1463, 1441, 1300, 1246, 1177, 1300, 1246, 1177, 1101, 1039, 1007, 834, 774 cm^{-1}. HRMS (ESI^+) calcd. for $C_{22}H_{37}O_2Si$ $[M+H]^+$: 361.25573; found: 361.25573.

SUJ-SA-457-01 Racemat
150 mm Chiralpak IB-N-3, 3 µm, 4.6 mm i.D.
n-Heptan / 2-Propanol = 99.96 : 0.04 (v/v)
1.0 ml/min / 9.7 MPa / 298 K

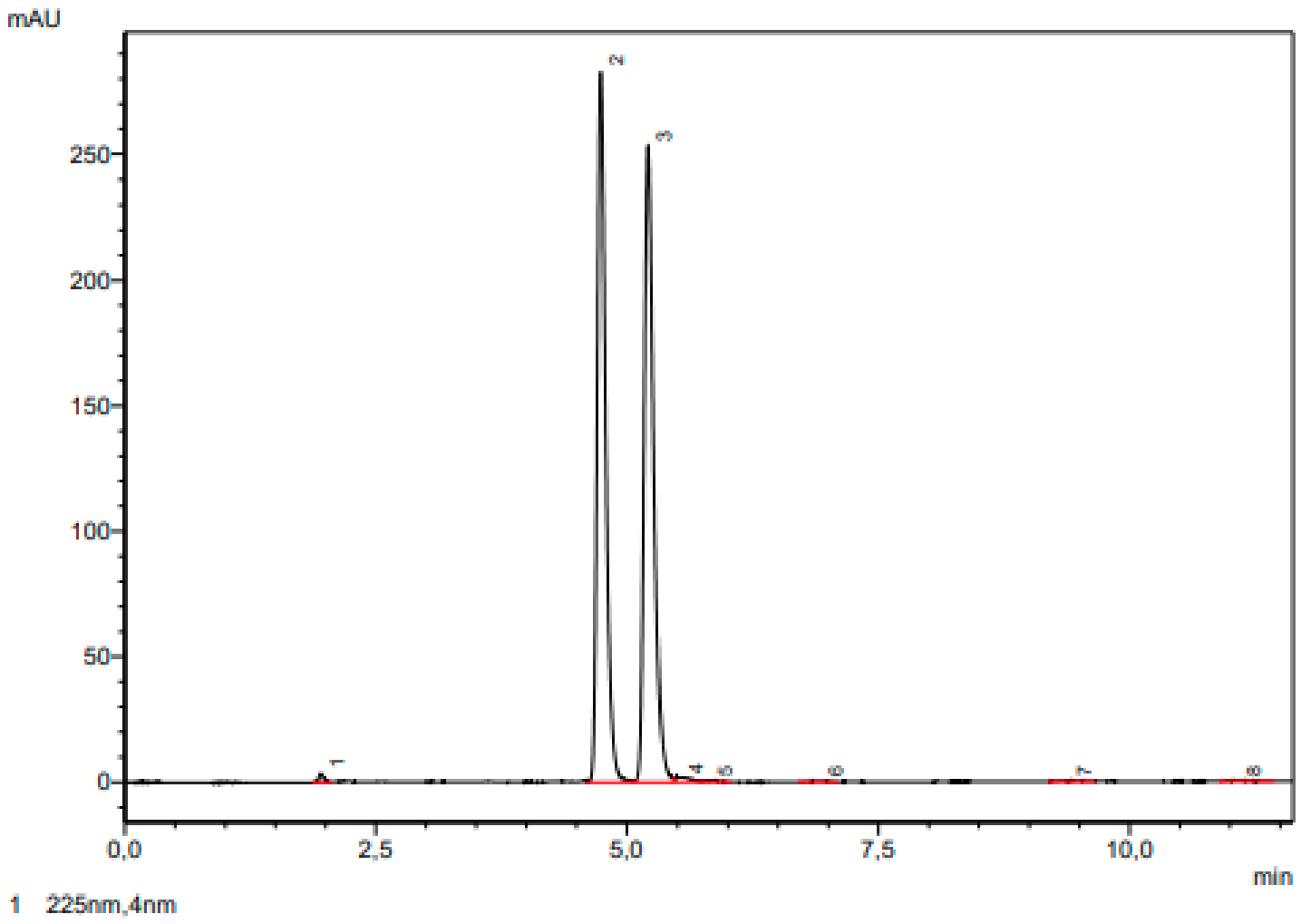

PDA Ch1 225nm

Peak #	Ret. Time	Area %	Name
1	1,95	0,37	
2	4,74	49,14	1. Enantiomer
3	5,21	49,30	2. Enantiomer
4	5,52	0,65	
5	5,82	0,16	
6	6,91	0,11	
7	9,39	0,17	
8	11,08	0,11	
Total		100,00	

SUJ-SA-465-01
150 mm Chiralpak IB-N-3, 3 µm, 4.6 mm i.D.
n-Heptan / 2-Propanol = 99.96 : 0.04 (v/v)
1.0 ml/min / 9.7 MPa / 298 K

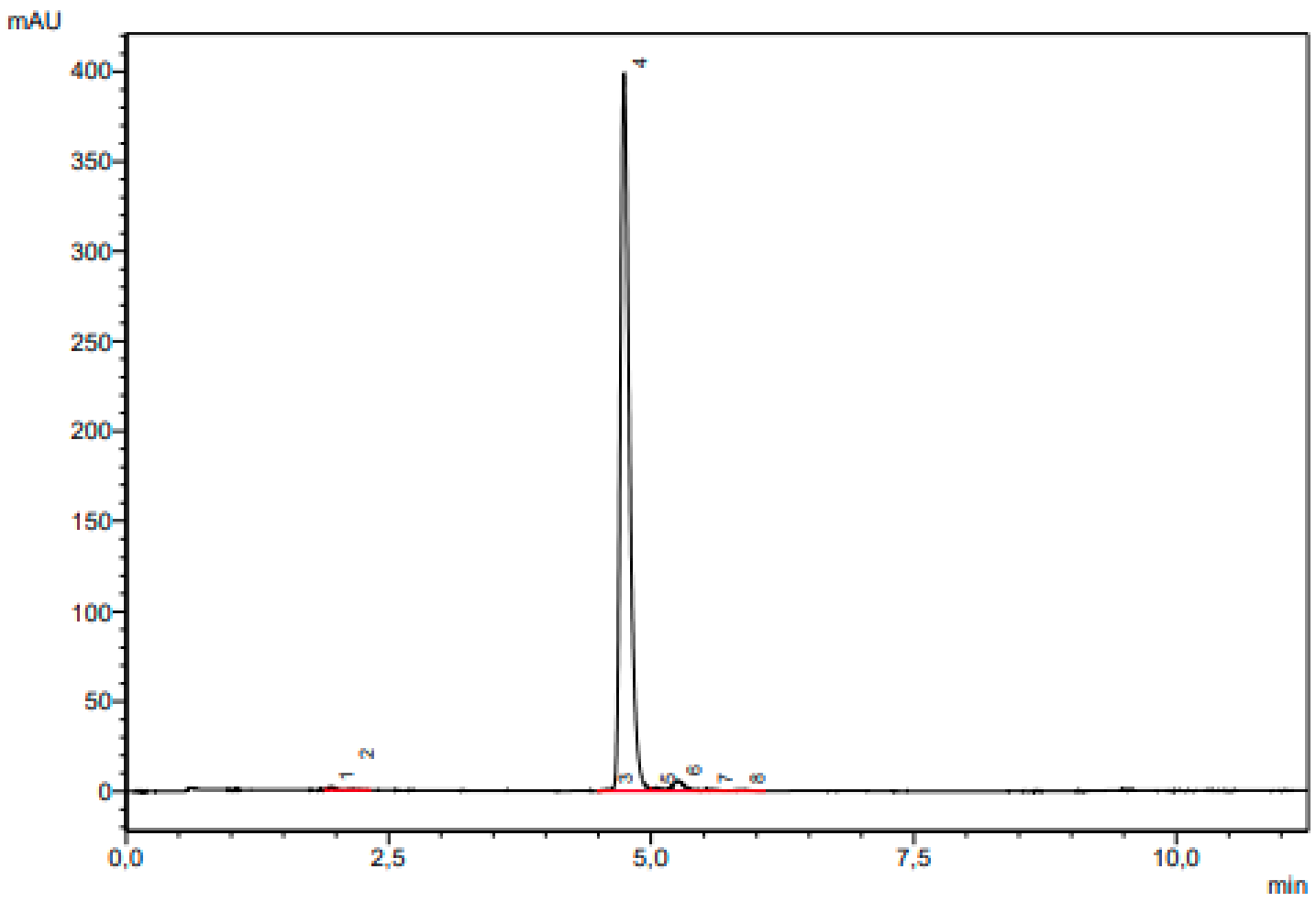

PDA Ch1 225nm

Peak #	Ret. Time	Area %	Name
1	1,95	0,26	
2	2,15	0,13	
3	4,60	0,16	
4	4,74	96,54	1. Enantiomer 96.2 % ee
5	5,00	0,45	
6	5,25	1,85	2. Enantiomer
7	5,55	0,35	
8	5,86	0,26	
Total		100,00	

Compound 9. Prepared according to the general procedure **F** from compound **S28**; colourless oil (10.6 mg, 81%). The e.e. was found to be 95.6% (see below for the HPLC data using a chiral stationary phase). $[\alpha]_D^{20}$= 20.2 (c = 0.57, $CHCl_3$). ^1H NMR (400 MHz, $CDCl_3$) δ 7.17 – 7.04 (m, 2H), 6.90 – 6.72 (m, 2H), 5.15 – 5.05 (m, 2H), 4.05 (t, J = 6.7 Hz, 2H), 3.79 (s, 3H), 2.65 (t, J = 7.7 Hz, 2H), 2.39 – 2.21 (m, 2H), 2.04 (s, 3H), 2.02 – 1.93 (m, 2H), 1.73 – 1.59 (m, 2H), 1.52 – 1.35 (m, 2H). ^{13}C NMR (101 MHz, $CDCl_3$) δ 204.2, 171.4, 157.9, 134.1, 129.5, 113.8, 91.0, 90.8, 64.6, 55.4, 34.7, 31.1, 28.6, 28.2, 25.5, 21.2. IR (film): $\tilde{\nu}$ 2926, 2855, 1737, 1611, 1513, 1458, 1366, 1300, 1245, 1177, 1037, 831 cm^{-1}. HRMS (EI^+) calcd. for $C_{18}H_{24}O_3$ $[M]^+$: 288.17200; found: 288.17223.

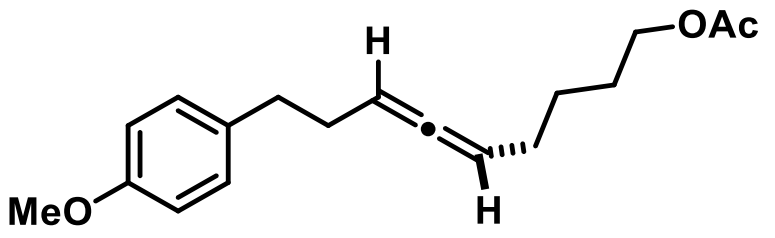

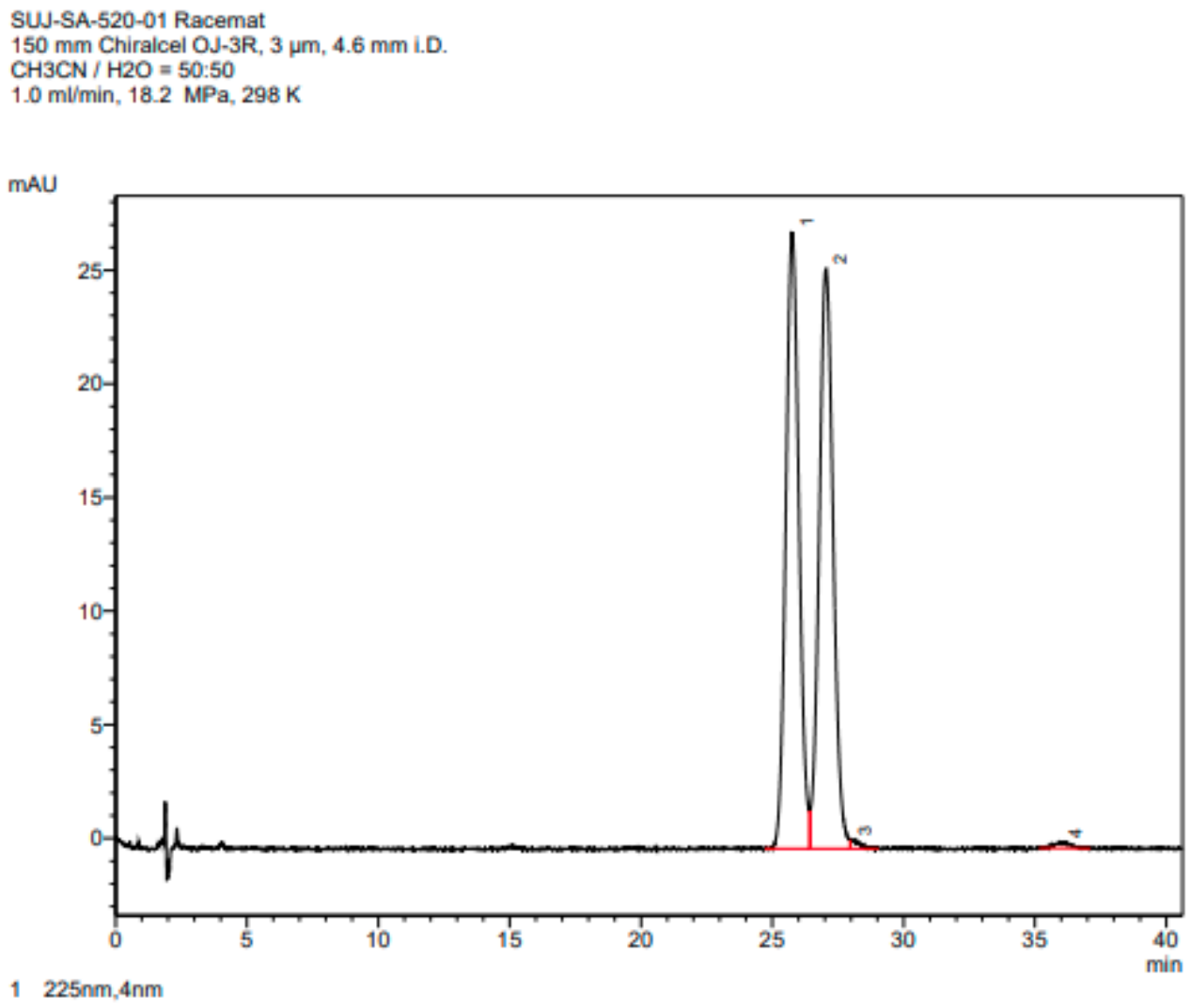

PDA Ch1 225nm

Peak #	Ret. Time	Area %	Name
1	25,76	49,23	1. Enantiomer
2	27,05	49,68	2. Enantiomer
3	27,98	0,48	
4	35,98	0,60	
Total		100,00	

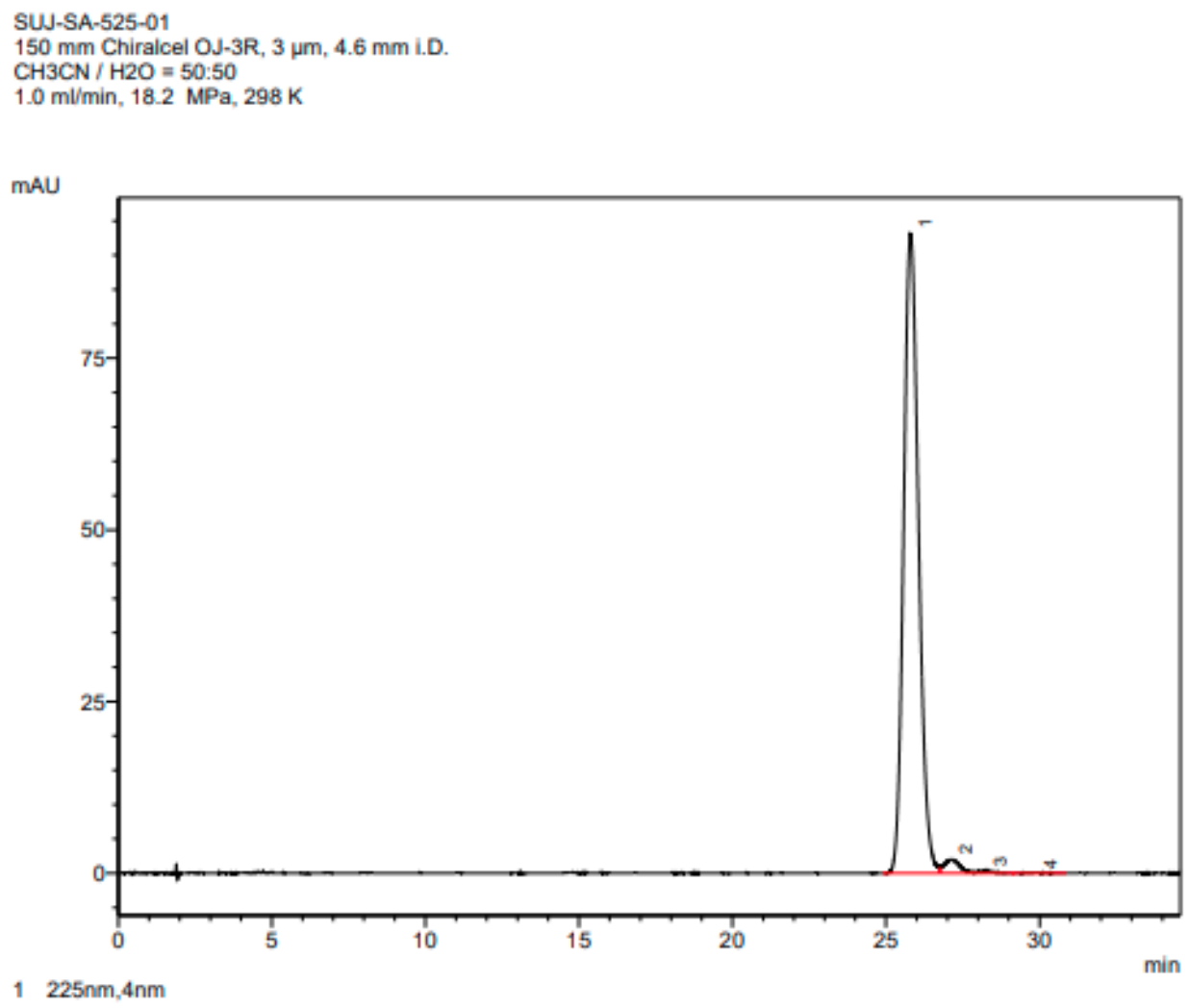

PDA Ch1 225nm

Peak #	Ret. Time	Area %	Name
1	25,80	97,33	1. Enantiomer 95.6 % ee
2	27,13	2,18	2. Enantiomer
3	28,25	0,38	
4	29,91	0,11	
Total		100,00	

Compound 10. Prepared according to the general procedure **F** from compound **S29**; colourless oil (12.1 mg, 73%). The e.e. was found to be 94.7% (see below for the HPLC data using a chiral stationary phase). $[\alpha]_D^{20}$= 61.8 (c = 0.40, $CHCl_3$). ^1H NMR (400 MHz, $CDCl_3$) δ 7.18 – 7.06 (m, 2H), 6.92 – 6.73 (m, 2H), 5.32 – 4.98 (m, 2H), 3.79 (s, 3H), 2.65 (t, *J* = 7.7 Hz, 2H), 2.47 – 2.34 (m, 1H), 2.33 – 2.20 (m, 2H), 1.81 – 1.68 (m, 2H), 1.67 – 1.59 (m, 2H), 1.59 – 1.48 (m, 2H), 1.38 – 1.29 (m, 2H). ^{13}C NMR (101 MHz, $CDCl_3$) δ 202.9, 157.9, 134.2, 129.5, 113.8, 96.6, 91.2, 55.4, 39.4, 34.8, 32.9, 32.8, 31.3, 25.0. IR (film): $\tilde{\nu}$ 2952, 2864, 1612, 1512, 1453, 1300, 1246, 1177, 1106, 1079, 1039 821 cm^{-1}. HRMS (EI^+) calcd. for $C_{17}H_{22}O$ $[M]^+$: 242.16652; found: 242.16646.

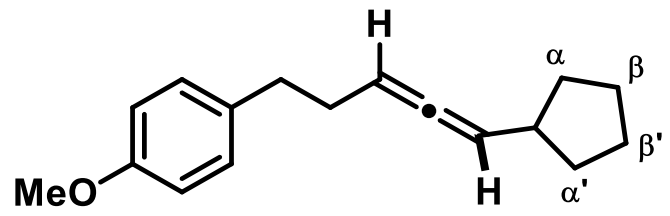

Note*: The carbon nuclei in the cyclopentyl ring of this compound are diastereotopic. This is reflected in the appearance of separate* ^{13}C *NMR resonances for* C_α *and* $C_{\alpha'}$*; however the resonances for* C_β *and* $C_{\beta'}$ *coincided; this was confirmed by analysis of the* ^1H-^{13}C *HSQC spectrum.*

SUJ-SA-507-01 Racemat
250 mm Chiralpak IB, 4.6 mm i.D.
n-Heptan / 2-Propanol = 99.95:0.05 (v/v)
1.0 ml/min / 6.8 MPa / 288 K

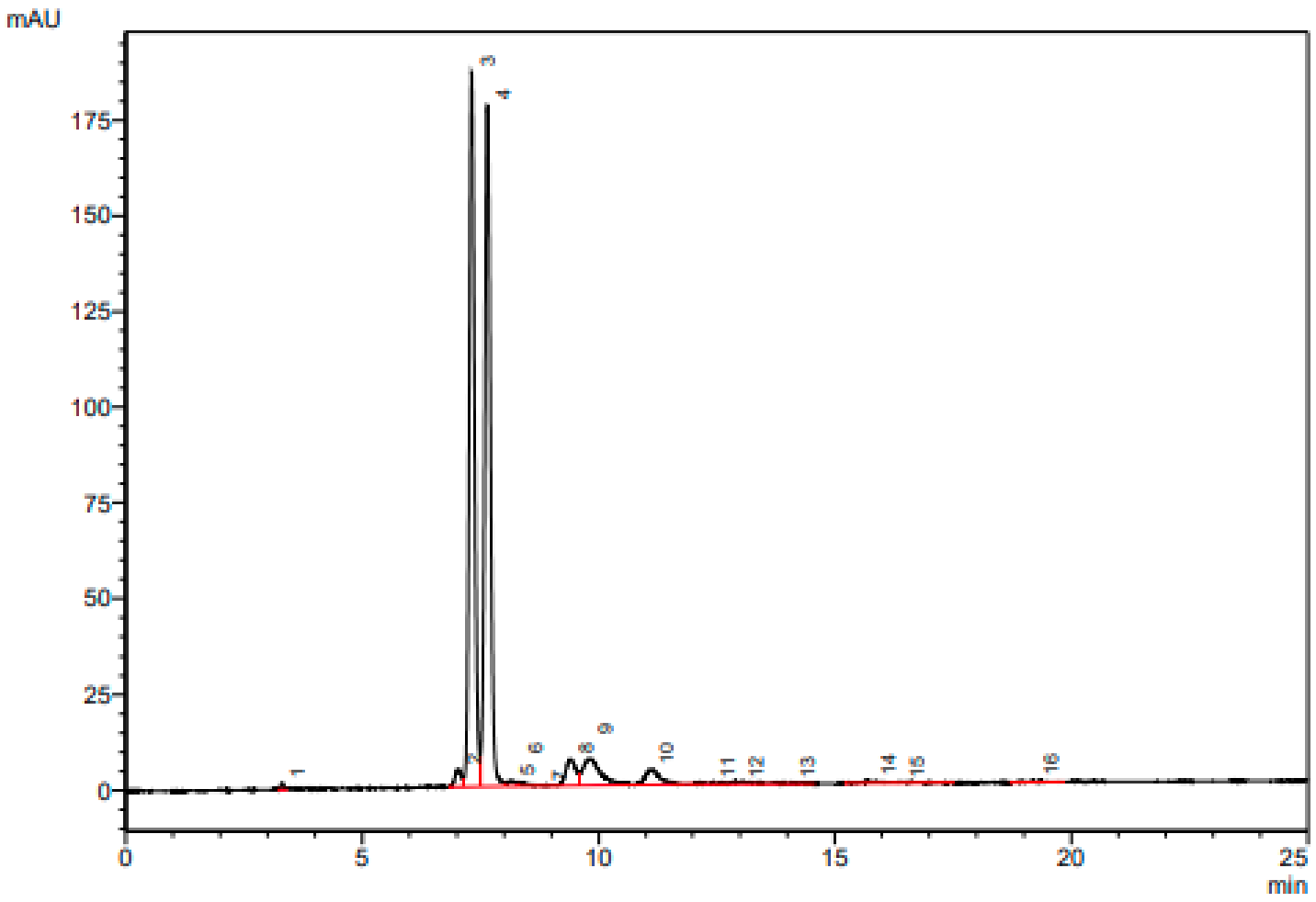

1 225nm,4nm

PDA Ch1 225nm

Peak #	Ret. Time	Area %	Name
1	3,30	0,23	
2	7,03	1,25	
3	7,31	42,08	1. Enantiomer
4	7,65	42,72	2. Enantiomer
5	8,14	0,50	
6	8,33	0,34	
7	8,83	0,14	
8	9,40	2,99	
9	9,80	5,28	
10	11,11	2,47	
11	12,40	0,26	
12	13,00	0,70	
13	14,10	0,11	
14	15,81	0,46	
15	16,41	0,30	
16	19,24	0,15	
Total		100,00	

SUJ-SA-526-01
250 mm Chiralpak IB, 4.6 mm i.D.
n-Heptan / 2-Propanol = 99.95:0.05 (v/v)
1.0 ml/min / 6.8 MPa / 288 K

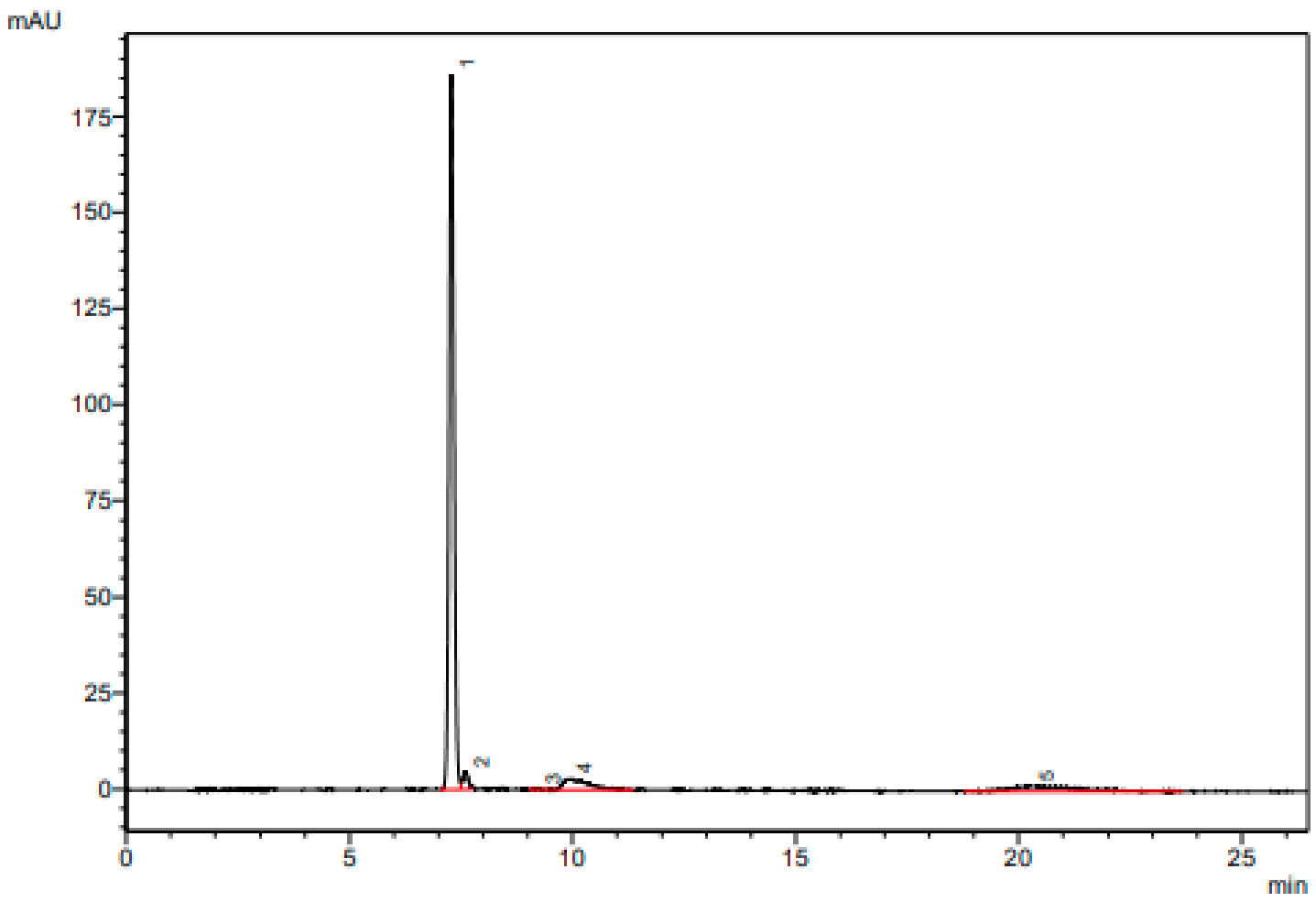

1 225nm,4nm

PDA Ch1 225nm

Peak #	Ret. Time	Area %	Name
1	7,29	82,40	1. Enantiomer 94.7 % ee
2	7,60	2,25	2. Enantiomer
3	9,21	0,12	
4	9,92	6,63	
5	20,28	8,59	
Total		100,00	

Compound 12. Prepared according to the general procedure **F** from compound **S32**; colourless oil (43.0

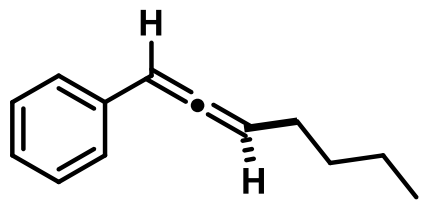

mg, 80%). The e.e. was found to be 66.3% (see below for the HPLC data using a chiral stationary phase). $[\alpha]_D^{20}$= –17.0 (c = 0.60, $CHCl_3$). ^1H NMR (400 MHz, $CDCl_3$) δ 7.34 – 7.27 (m, 4H), 7.22 – 7.14 (m, 1H), 6.12 (dt, *J* = 6.2, 3.0 Hz, 1H), 5.57 (q, *J* = 6.7 Hz, 1H), 2.19 – 2.08 (m, 2H), 1.53 – 1.34 (m, 4H), 0.91 (t, *J* = 7.2 Hz, 3H). ^{13}C NMR (101 MHz, $CDCl_3$) δ 204.3, 157.9, 134.0, 129.5, 113.8, 91.3, 89.9, 55.4, 44.5, 34.7, 31.8, 31.0, 26.0. ^{13}C NMR (101 MHz, $CDCl_3$) δ 205.3, 135.3, 128.7, 126.73, 126.70, 95.2, 94.7, 31.5, 28.6, 22.4, 14.0. IR (film): $\tilde{\nu}$ 2957, 2925, 2857, 1949, 1727, 1674, 1598, 1495, 1458, 1378, 1263, 1166, 1101, 1078, 1027, 965, 910, 874, 823, 793, 770, 691, 652, 628, 555, 496, 460, 417 cm^{-1}. HRMS (ESI^+) calcd. for $C_{13}H_{17}$ $[M+H]^+$: 173.1324; found: 173.1324.

SFS-SD-985-01-Racemat
150 mm Chiralpak IB-N-3, 3 µm, 4.6 mm i.D.
n-Heptan / 2-Propanol = 99.96:0.04 (v/v)
1.0 ml/min / 8.2 MPa / 293 K

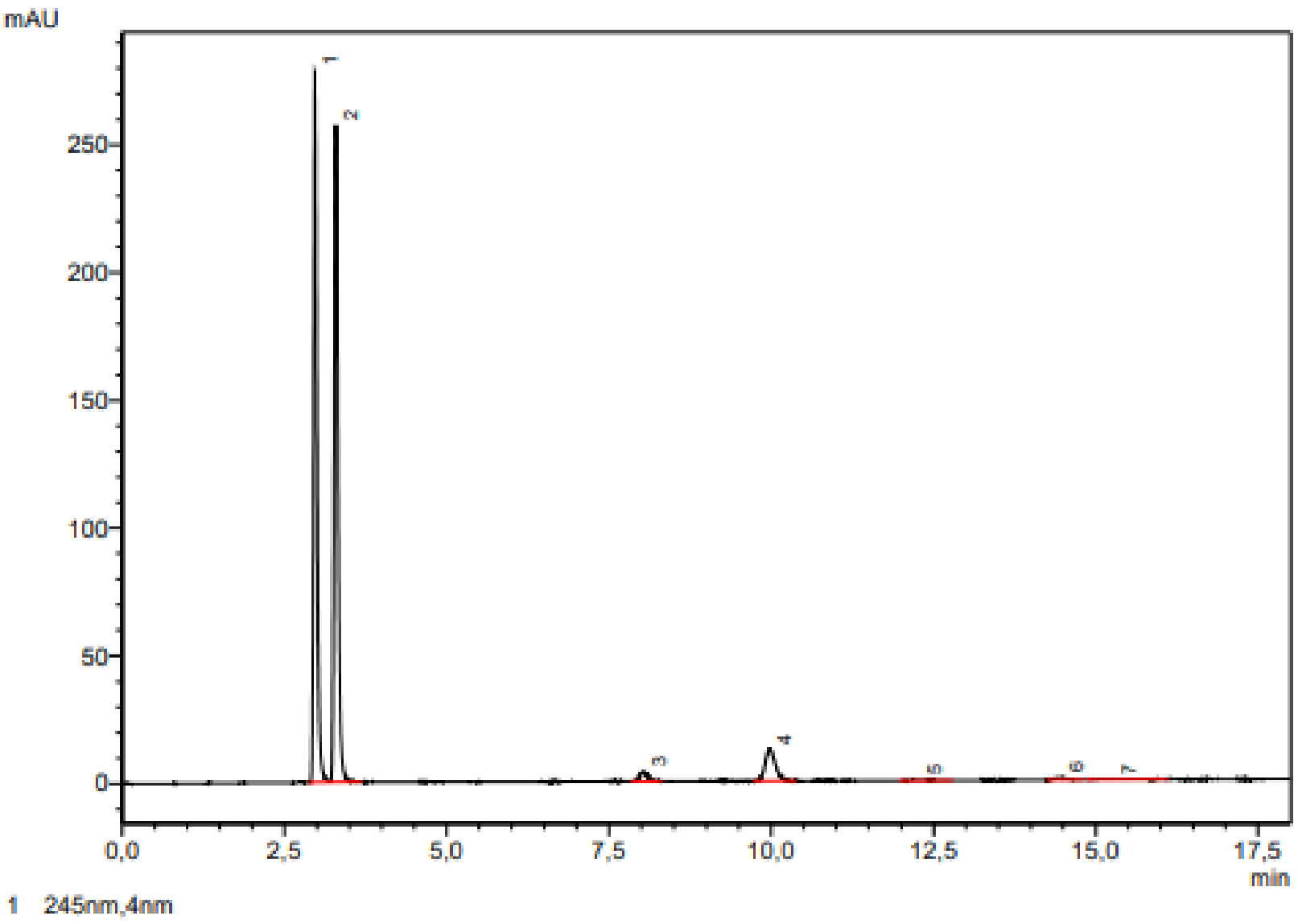

PDA Ch1 245nm

Peak #	Ret. Time	Area %	Name
1	2,97	45,56	1. Enantiomer
2	3,29	45,80	2. Enantiomer
3	8,03	1,47	
4	9,97	6,14	
5	12,27	0,19	
6	14,46	0,43	
7	15,27	0,40	
Total		100,00	

SFS-SD-983-01
150 mm Chiralpak IB-N-3, 3 µm, 4.6 mm i.D.
n-Heptan / 2-Propanol = 99.96:0.04 (v/v)
1.0 ml/min / 8.2 MPa / 293 K

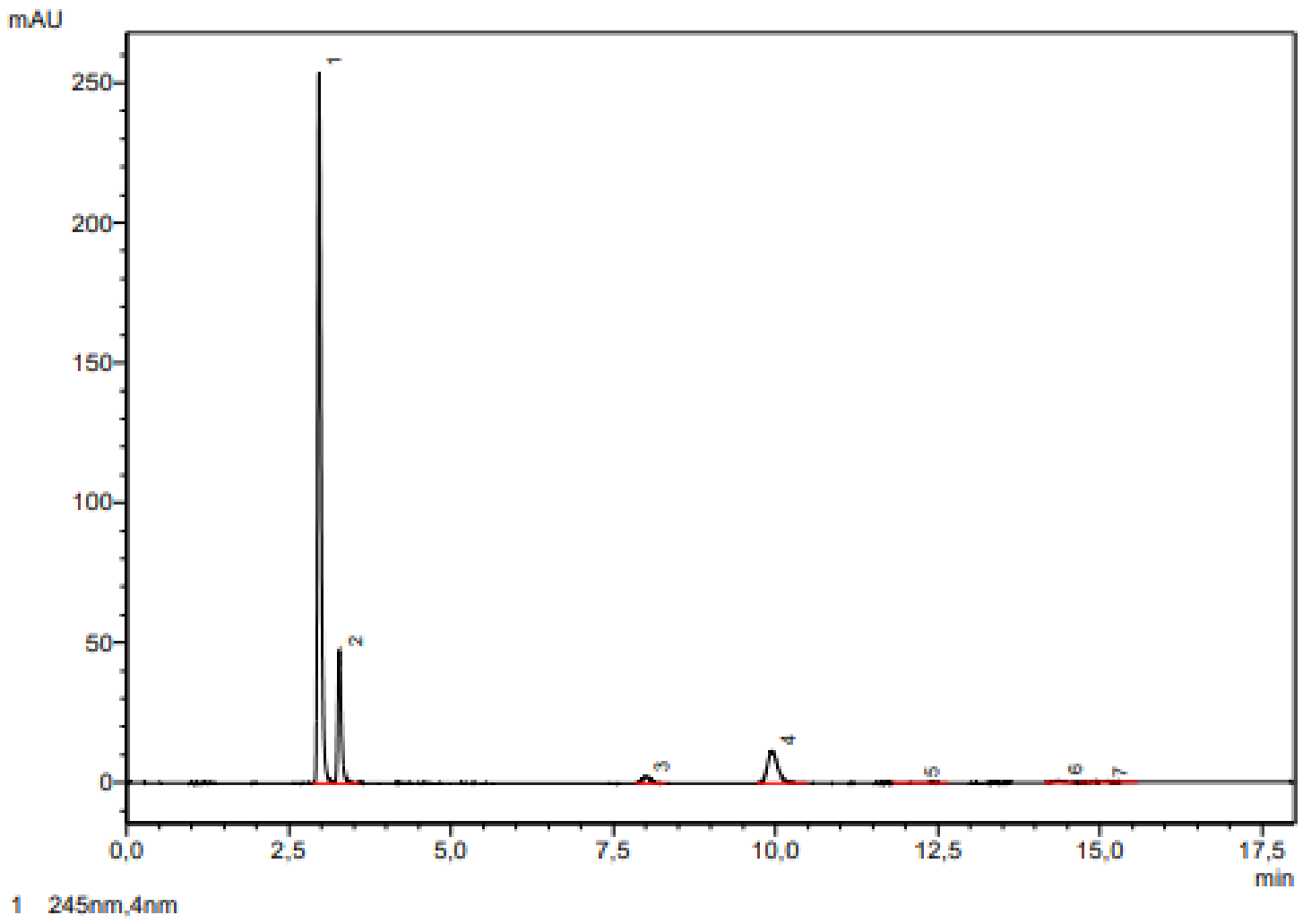

PDA Ch1 245nm

Peak #	Ret. Time	Area %	Name
1	2,97	71,99	1. Enantiomer 66.3 % ee
2	3,28	14,61	2. Enantiomer
3	8,01	1,85	
4	9,95	10,25	
5	12,16	0,22	
6	14,37	0,69	
7	15,06	0,39	
Total		100,00	

Compound 11a. Prepared by an adaptation of the general procedure **F**: Freshly prepared LDA (0.1 M in THF, 899 µL, 89.9 mmol) was added dropwise to a solution of allylic alcohol **S31** (30.0 mg, 74.9 µmol) in THF (0.375 mL) at −20 °C. A solution of CuI (0.1 M in THF/NEt_3 10:1 *v/v*, 150 µL, 20 mol%) and NEt_3 (127 µL) were added. The mixture was gradually warmed to room temperature while stirring was continued for 24 h, when TLC induced complete consumption of the starting material. Sat. aq. NH_4Cl was added, and the mixture was diluted with ethyl acetate and water. The organic layer was separated, the aqueous layer was extracted three times with ethyl acetate, and the combined organic extracts were washed with brine, dried over anhydrous magnesium sulfate, and filtered. The filtrate was concentrated under reduced pressure and the residue purified by flash column chromatography (0-5% *tert*-butyl methyl ether/hexanes) to give the title compound as a colorless oil (12.0 mg, 64% yield). The e.e. was found to be 71.2% (see below for the HPLC data using a chiral stationary phase). $[\alpha]_D^{20}$= 115.3 (c = 1.20, $CHCl_3$). ^1H NMR (400 MHz, $CDCl_3$) δ 7.28 – 7.24 (m, 2H), 7.19 – 7.11 (m, 5H), 6.85 – 6.82 (m, 2H), 6.12 (dt, J = 6.3, 3.0 Hz, 1H), 5.57 (q, J = 6.6 Hz, 1H), 3.80 (s, 3H), 2.82 – 2.70 (m, 2H), 2.51 – 2.35 (m, 2H). ^{13}C NMR (101 MHz, $CDCl_3$) δ 205.4, 158.0, 135.0, 133.8, 129.6, 128.6, 126.81, 126.78, 113.9, 95.0, 94.5, 55.4, 34.6, 30.9. IR (neat): ν 2922, 2852, 1948, 1612, 1511, 1496, 1460, 1300, 1245, 1177, 1106, 1071, 1038, 911, 874, 821, 808, 777, 724, 691, 628, 540, 517 cm^{-1}. HRMS (ESI^+) calcd. for $C_{18}H_{18}ONa$ $[M+Na]^+$: 273.12499; found: 273.12482.

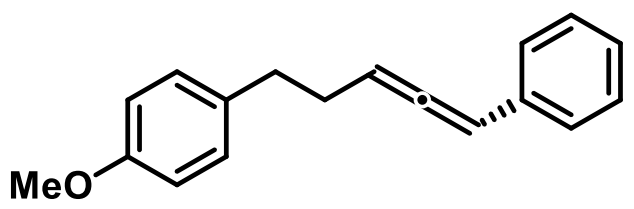

The data are consistent with the literature.[7]

MOP-MA-491-01 Racemat
150 mm Chiralcel OJ-3R, 3 µm, 4.6 mm i.D.
CH3OH
1.0 ml/min / 114 bar / 298 K

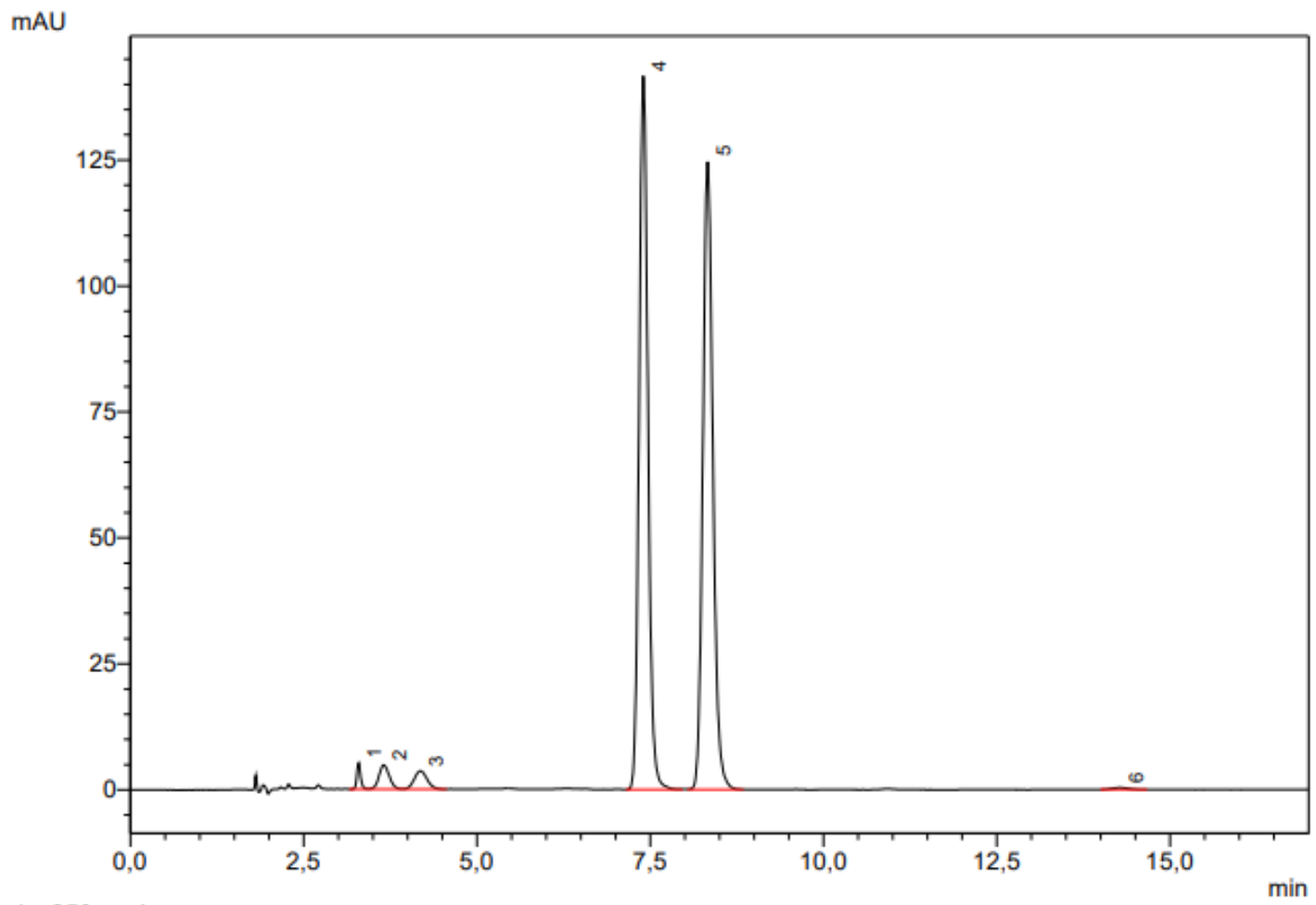

1 250nm,4nm

PDA Ch1 250nm

Peak #	Ret. Time	Area %	Name
1	3,29	0,75	
2	3,66	1,86	
3	4,19	1,76	
4	7,40	47,69	1. Enantiomer
5	8,33	47,72	2. Enantiomer
6	14,28	0,22	
Total		100,00	

MOP-MA-492-01
150 mm Chiralcel OJ-3R, 3 µm, 4.6 mm i.D.
CH3OH
1.0 ml/min / 114 bar / 298 K

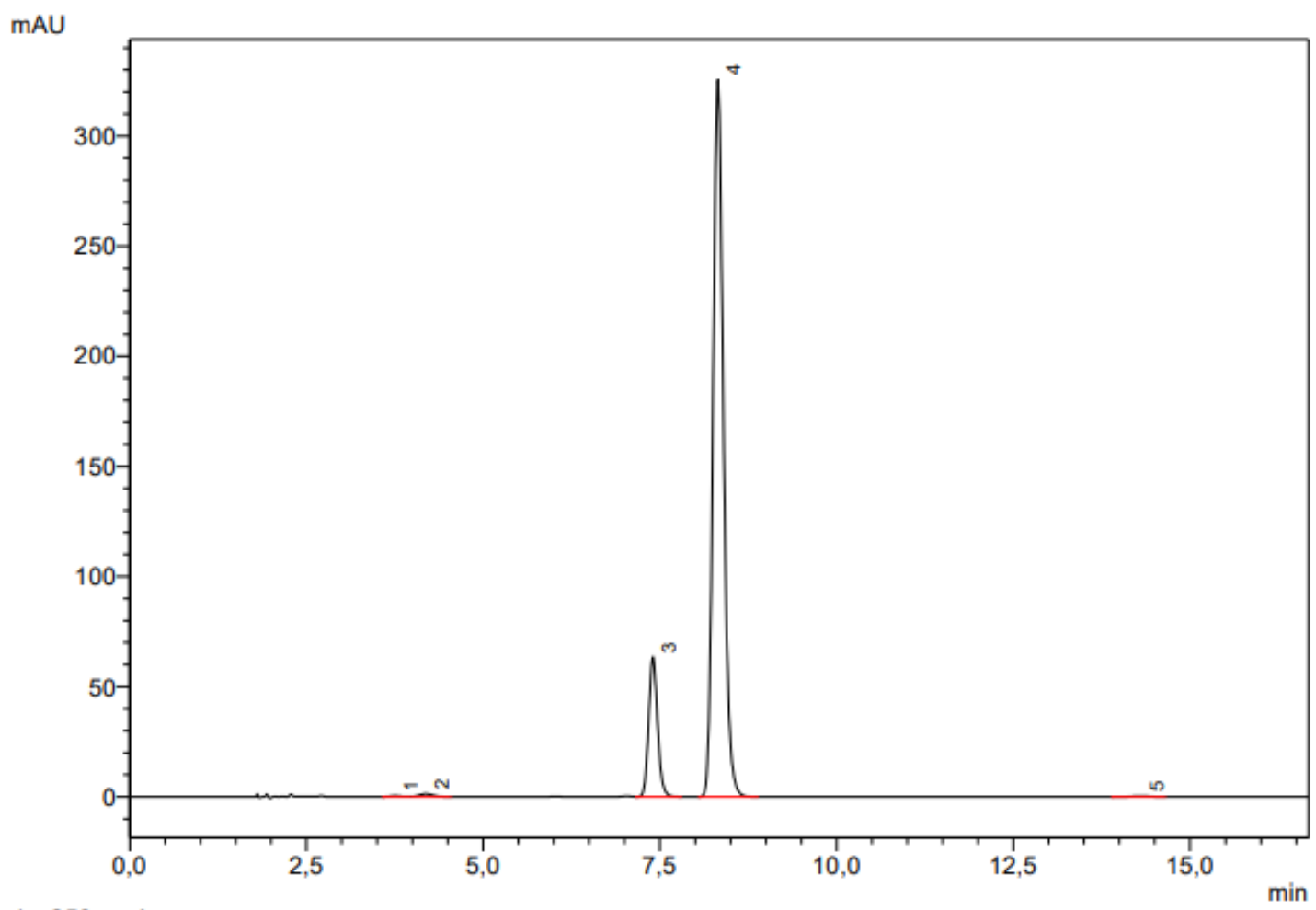

1 250nm,4nm

PDA Ch1 250nm

Peak #	Ret. Time	Area %	Name
1	3,75	0,12	
2	4,19	0,43	
3	7,40	14,27	1. Enantiomer
4	8,32	85,02	2. Enantiomer 71.2 % ee
5	14,30	0,17	
Total		100,00	

Compound 11b. Prepared according to the general procedure **F** from compound **S30**; colourless oil

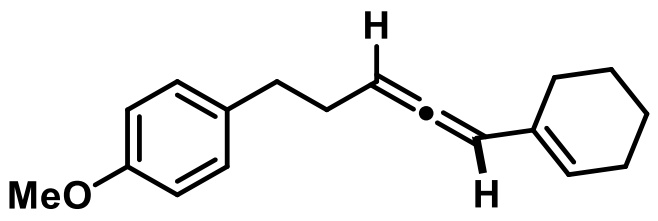

(10.1 mg, 73%). ^{1}H NMR (400 MHz, $CDCl_3$) δ 7.17 – 7.04 (m, 2H), 6.89 – 6.76 (m, 2H), 5.81 (dt, J = 6.1, 3.0 Hz, 1H), 5.67 – 5.58 (m, 1H), 5.44 – 5.31 (m, 1H), 3.79 (s, 3H), 2.80 – 2.56 (m, 2H), 2.40 – 2.21 (m, 2H), 2.15 – 2.03 (m, 2H), 2.03 – 1.89 (m, 1H), 1.88 – 1.76 (m, 1H), 1.67 – 1.57 (m, 4H). ^{13}C NMR (101 MHz, $CDCl_3$) δ 204.5, 157.9, 134.0, 132.6, 129.6, 125.7, 113.8, 98.2, 93.6, 55.4, 34.7, 31.4, 26.0, 25.7, 22.7, 22.6. IR (film): $\tilde{\nu}$ 2924, 2855, 2833, 1612, 1511, 1439 ,1300, 1245, 1177, 1105, 1039, 880, 821, 804 cm^{-1}. HRMS (EI$^+$) calcd. for $C_{18}H_{22}O$ [M]$^+$: 254.16651; found: 254.16630.

Although the chosen substrate was optically pure (98.5% ee), the reaction furnished racemic product as confirmed by the HPLC data using a chiral stationary phase, see below:

SUJ-SA-560-01 Racemat
150 mm Chiralpak IB-N-3, 3 µm, 4.6 mm i.D.
n-Heptan / 2-Propanol = 99.95:0.05 (v/v)
1.0 ml/min / 9.7 MPa / 293 K

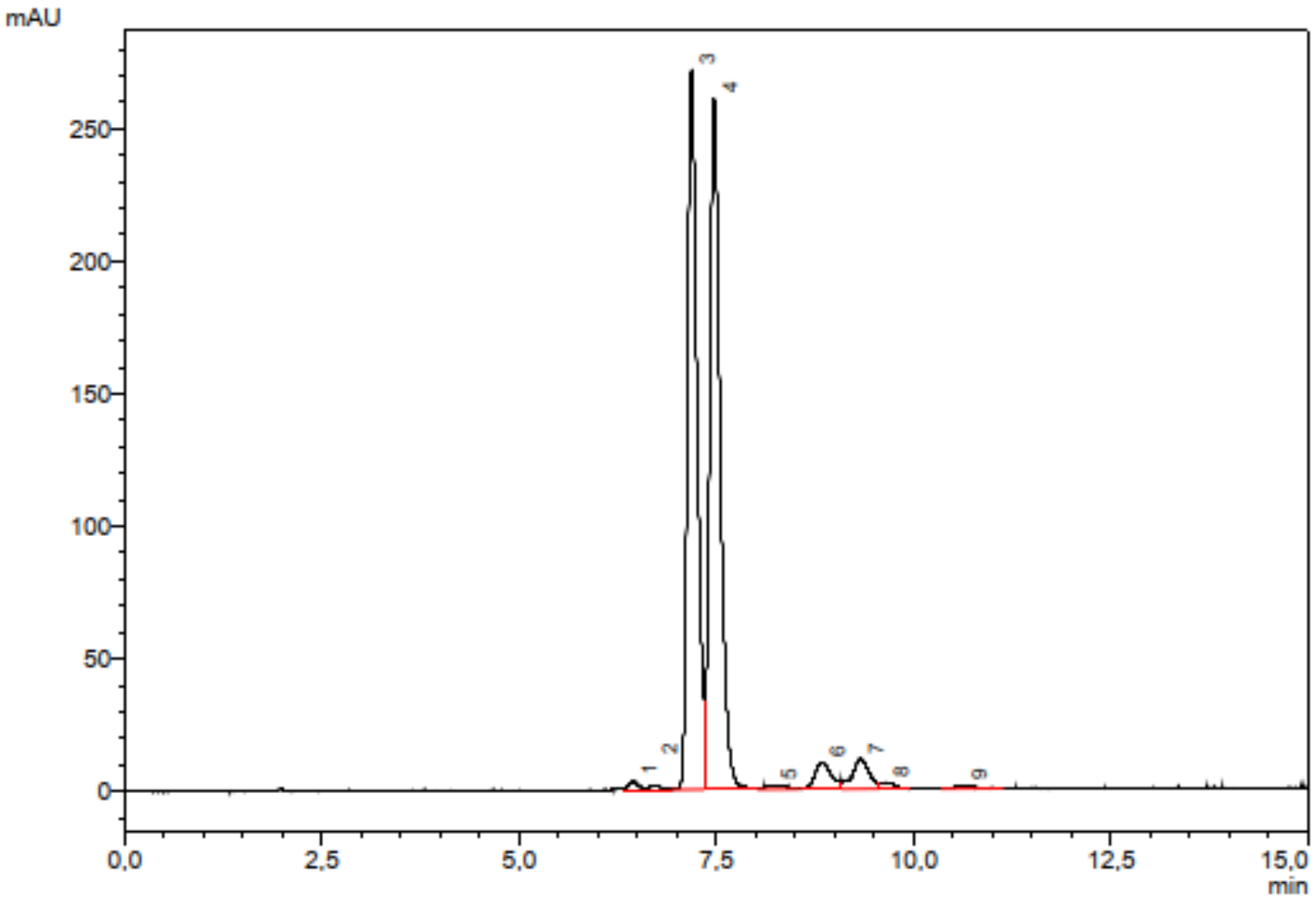

1 225nm,4nm

PDA Ch1 225nm

Peak #	Ret. Time	Area %	Name
1	6,45	0,52	
2	6,71	0,31	
3	7,18	44,47	1. Enantiomer
4	7,47	47,03	2. Enantiomer
5	8,22	0,50	
6	8,83	2,81	
7	9,32	3,34	
8	9,64	0,58	
9	10,63	0,44	
Total		100,00	

SUJ-SA-561-01
150 mm Chiralpak IB-N-3, 3 µm, 4.6 mm i.D.
n-Heptan / 2-Propanol = 99.95:0.05 (v/v)
1.0 ml/min / 9.7 MPa / 293 K

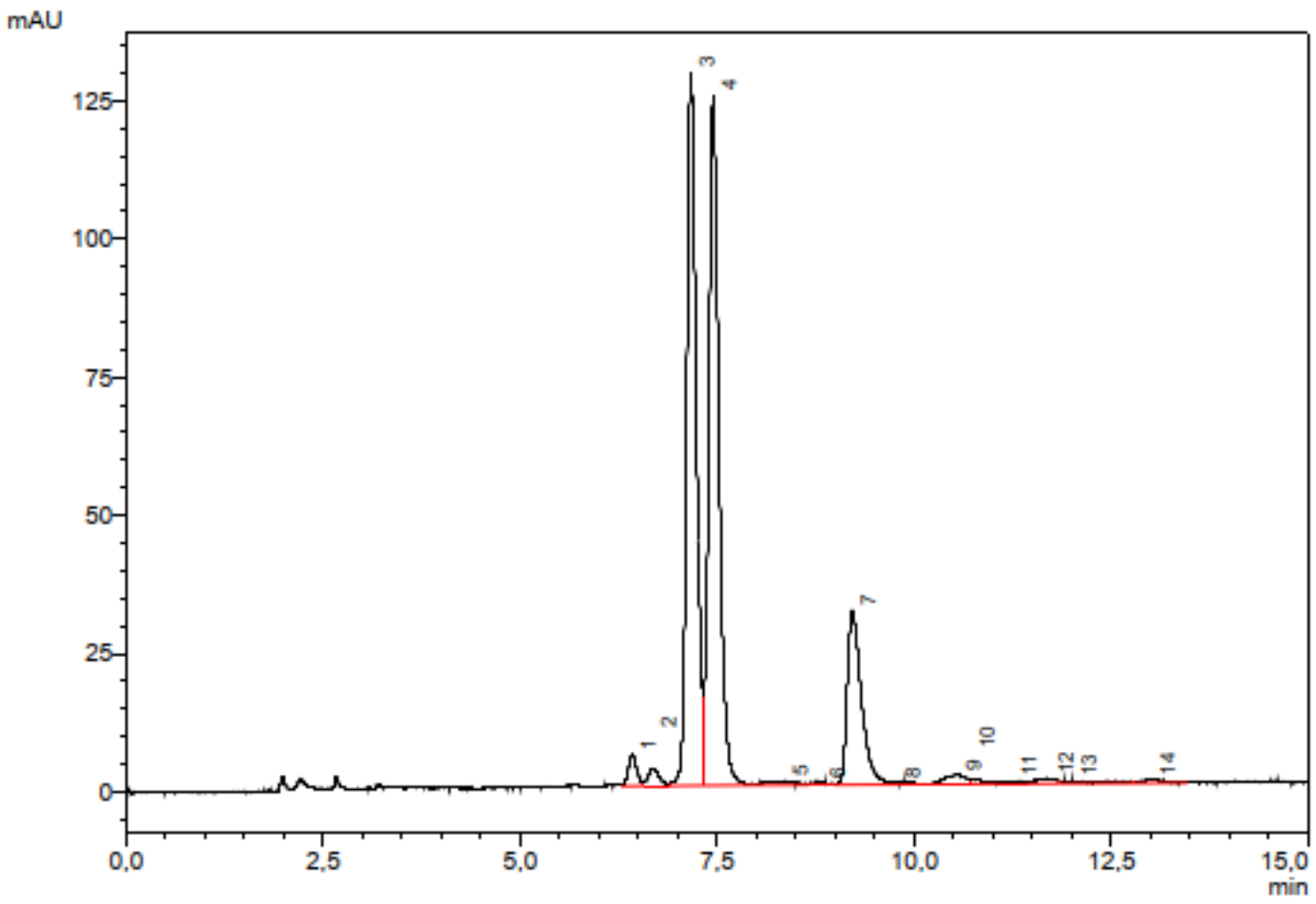

1 225nm,4nm

PDA Ch1 225nm

Peak #	Ret. Time	Area %	Name
1	6,42	1,49	
2	6,68	1,07	
3	7,17	38,75	1. Enantiomer
4	7,45	40,26	2. Enantiomer
5	8,35	0,69	
6	8,83	0,19	
7	9,21	13,95	
8	9,78	0,20	
9	10,55	1,14	
10	10,72	0,36	
11	11,25	0,30	
12	11,72	0,58	
13	12,01	0,61	
14	13,01	0,41	
Total		100,00	

Compound 13. Prepared according to the general procedure **F** from compound **S33**; colourless oil (5.1 mg, 82%). ^{1}H NMR (400 MHz, $CDCl_3$) δ 7.15 – 7.01 (m, 2H), 6.95 – 6.71 (m, 2H), 5.01 (tp, *J* = 8.8, 2.9 Hz, 1H), 3.78 (s, 3H), 2.70 – 2.62 (m, 2H), 2.42 – 2.13 (m, 2H), 1.99 – 1.83 (m, 2H), 1.69 (d, *J* = 2.9 Hz, 3H), 1.41 – 1.29 (m, 4H), 0.96 – 0.83 (m, 3H). ^{13}C NMR (101 MHz, $CDCl_3$) δ 201.4, 157.8, 134.7, 129.4, 113.8, 98.9, 91.0, 55.4, 36.2, 33.3, 31.6, 29.2, 22.3, 19.6, 14.1. IR (film): $\tilde{\nu}$ 2957, 2926, 2872, 2856, 1612, 1512, 1464, 1441, 1300, 1246, 1176, 1103, 1080, 1040, 960, 821, 771 cm^{-1}. HRMS (EI$^+$) calcd. for $C_{17}H_{24}O$ [M]$^+$: 244.18217; found: 244.18214.

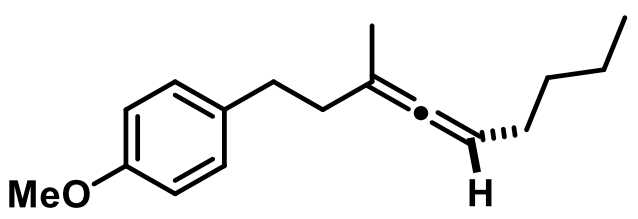

Compound 14. Prepared according to the general procedure **F** from compound **S34**; colourless oil (9.5 mg, 89%). ^{1}H NMR (400 MHz, $CDCl_3$) δ 5.20 – 5.09 (m, 1H), 5.06 – 4.90 (m, 1H), 4.16 (ddd, *J* = 11.1, 7.6, 2.7 Hz, 1H), 4.07 (ddd, *J* = 11.0, 7.6, 2.8 Hz, 1H), 2.43 – 2.25 (m, 2H), 2.17 – 2.05 (m, 1H), 2.06 – 1.89 (m, 3H), 1.81 – 1.59 (m, 4H), 1.55 – 1.27 (m, 8H). ^{13}C NMR (101 MHz, $CDCl_3$) δ 204.5, 174.4, 91.5, 90.7, 64.4, 34.9, 28.6, 28.30, 28.29, 28.24, 28.19, 27.7, 26.1, 25.6. IR (film): $\tilde{\nu}$ 2927, 2855, 1960, 1731, 1458, 1339, 1260, 1230, 1178, 1136, 1059, 1002, 876 cm^{-1}. HRMS (ESI$^+$) calcd. for $C_{14}H_{22}O_2Na$ [M+Na]$^+$: 245.15120; found: 245.15104.

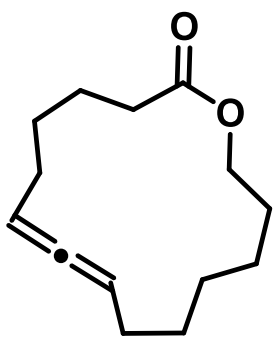

Alkenylcopper Trapping: Functionalized Trisubstituted Allylic Alcohols

Compound 3. Compound **1** (19.8 mg, 0.0520 mmol) was dissolved in THF (1.0 mL) and the resulting solution was cooled to −30 °C (bath temperature). A solution of freshly prepared LDA (0.1 M in THF, 0.6 mL, 0.060 mmol) was added in a single portion, and the solution was stirred for 5 min. Copper(I) iodide (20.3 mg, 0.107 mmol) was added and stirring was continued whilst maintaining a reaction temperature of between −30 °C and −25 °C. Once full consumption of the starting material was detected by thin-layer chromatography, the reaction was quenched by the addition of saturated aqueous ammonium chloride solution, and was diluted with water and ethyl acetate. The organic phase was separated and the aqueous phase was extracted three times with ethyl acetate. The combined organic extracts were washed with brine, dried over anhydrous magnesium sulfate, and filtered. The volatile organics were removed under reduced pressure to give the title compound as a pale yellow oil (19.7 mg, 99%). ^{1}H NMR (400 MHz, $CDCl_3$) δ 7.15 – 6.98 (m, 2H), 6.86 – 6.62 (m, 2H), 5.69 – 5.53 (m, 1H), 5.45 (ddt, *J* = 15.3, 7.2, 1.3 Hz, 1H), 4.26 (q, *J* = 6.6 Hz, 1H), 3.94 – 3.63 (m, 7H), 2.76 – 2.47 (m, 2H), 2.03 (q, *J* = 6.7 Hz, 2H), 1.93 – 1.81 (m, 1H), 1.81 – 1.69 (m, 1H), 1.38 – 1.29 (m, 4H), 1.21 (t, *J* = 7.0 Hz, 6H), 0.90 (t, *J* = 4.7 Hz, 3H), 0.11 (s, 3H). ^{13}C NMR (101 MHz, $CDCl_3$) δ 157.8, 134.5, 132.5, 131.8, 129.4, 113.9, 73.4, 58.39, 58.38, 55.4, 40.1, 32.0, 31.5, 30.9, 22.4, 18.4, 14.1, −6.0. IR (film): $\tilde{\nu}$ 2957, 2926, 2873, 1613, 1512, 1465, 1442, 1299, 1246, 1175, 1102, 1076, 1040, 966, 822 cm^{-1}. HRMS (ESI$^+$) calcd. for $C_{21}H_{36}O_4SiNa$ $[M+Na]^+$: 403.22751; found: 403.22790.

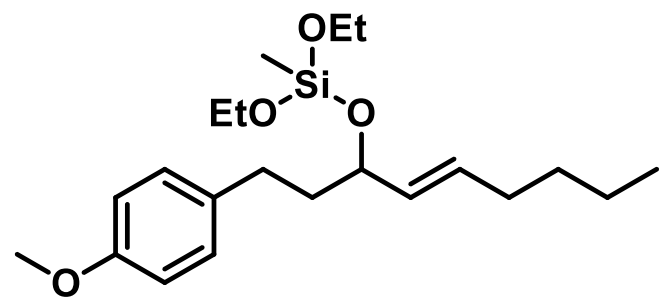

Compound 15. Compound **1** (35.0 mg, 0.0920 mmol) was dissolved in THF (0.9 mL) and the resulting solution was cooled to −30 °C (bath temperature). A solution of freshly prepared LDA (0.1 M in THF, 1.00 mL, 0.100 mmol) was added in a single portion, and the resulting solution was stirred for 5 min. Copper(I) iodide (21.0 mg, 0.110 mmol) was then added and stirring continued whilst maintaining a reaction temperature of between −30 °C and −25 °C. Once full consumption of the starting material was detected by thin-layer chromatography (approximately 50 min), iodomethane (0.020 mL, 0.32 mmol) was added in a single portion. The reaction was stirred for a 1 h before being warmed to room temperature. Excess iodomethane was quenched by the addition of saturated aqueous ammonium chloride solution and saturated aqueous sodium bicarbonate solution, followed by 15 min of rapid stirring. The mixture was diluted with water and ethyl acetate, the organic phase was separated, and the aqueous layer was extracted three times with ethyl acetate. The combined organic extracts were washed with brine, dried over anhydrous magnesium sulfate, and filtered. The volatile organics were removed under reduced pressure and the residue was purified *via* flash chromatography (eluting with 0→5% ethyl acetate/cyclohexane) to give the title compound as a

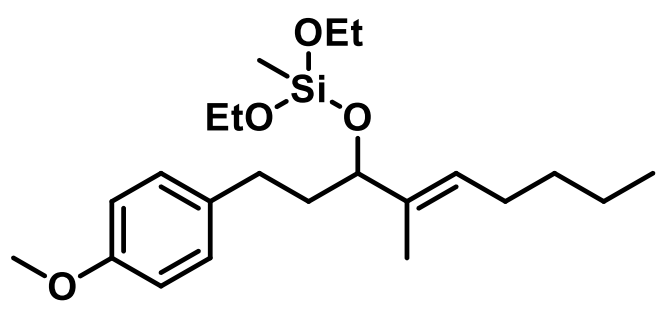

colourless oil (32.1 mg, 88%). ^{1}H NMR (400 MHz, $CDCl_3$) δ 7.17 – 7.03 (m, 2H), 6.96 – 6.72 (m, 2H), 5.43 – 5.26 (m, 1H), 4.19 (t, *J* = 6.6 Hz, 1H), 3.90 – 3.66 (m, 7H), 2.51 (dddd, *J* = 35.8, 13.9, 10.2, 6.0 Hz, 2H), 2.08 – 1.95 (m, 2H), 1.88 (dddd, *J* = 13.2, 10.2, 7.1, 6.1 Hz, 1H), 1.77 (ddt, *J* = 13.5, 10.2, 6.0 Hz, 1H), 1.60 (dt, *J* = 1.5, 0.8 Hz, 3H), 1.40 – 1.28 (m, 4H), 1.20 (t, *J* = 7.0 Hz, 6H), 0.97 – 0.83 (m, 3H), 0.08 (s, 3H). ^{13}C NMR (101 MHz, $CDCl_3$) δ 157.8, 136.2, 134.6, 129.4, 127.1, 113.8, 78.2, 58.4, 58.3, 37.9, 31.9, 31.3, 27.3, 22.5, 18.39, 18.37, 14.1, 11.1, –6.3. IR (film): $\tilde{\nu}$ 2957, 2927, 1612, 1512, 1464, 1442, 1299, 1261, 1245, 1175, 1102, 1073, 1038, 986, 956, 888, 823, 790 cm^{-1}. HRMS (ESI^+) calcd. for $C_{22}H_{38}O_4SiNa$ $[M+Na]^+$: 417.24316; found: 417.24338.

Compound 16. Compound **1** (33.0 mg, 0.0867 mmol) was dissolved in THF (0.9 mL) and the resulting solution was cooled to –30 °C (bath temperature). A solution of freshly prepared LDA (0.1 M in THF, 1.00 mL, 0.100 mmol) was added in a single portion, and the resulting solution was stirred for 5 min. Copper(I) iodide (19.9 mg, 0.105 mmol) was added and stirring continued whilst maintaining a reaction temperature of between –30 °C and –25 °C. Once full consumption of the starting material was detected by thin-layer chromatography (approximately 50 min), allyl bromide (0.030 mL, 0.35 mmol) was added in a single portion. The reaction was stirred for 1 h, before being warmed to room temperature. Excess allyl bromide was quenched by the addition of saturated aqueous ammonium chloride and saturated aqueous sodium bicarbonate solutions, followed by 15 min of rapid stirring. The mixture was diluted with water and ethyl acetate, the organic phase was separated, and the aqueous layer was extracted three times with ethyl acetate. The combined organic extracts were washed with brine, dried over anhydrous magnesium sulfate, and filtered. The volatile organics were removed under reduced pressure and the residue was purified *via* flash chromatography (eluting with 0→5% ethyl acetate/cyclohexane) to give the title compound as a colourless oil (32.1 mg, 88%). ^{1}H NMR (400 MHz, $CDCl_3$) δ 7.14 – 7.03 (m, 2H), 6.87 – 6.79 (m, 2H), 5.77 (dddd, *J* = 17.0, 10.0, 6.8, 6.1 Hz, 1H), 5.56 – 5.42 (m, 1H), 5.07 – 4.99 (m, 1H), 4.96 (dq, *J* = 10.0, 1.6 Hz, 1H), 4.24 (t, *J* = 6.3 Hz, 1H), 3.96 – 3.67 (m, 7H), 2.93 – 2.75 (m, 2H), 2.63 – 2.44 (m, 2H), 2.14 – 1.97 (m, 2H), 1.93 – 1.74 (m, 2H), 1.42 – 1.29 (m, 4H), 1.27 – 1.13 (m, 6H), 0.96 – 0.81 (m, 3H), 0.09 (s, 3H). ^{13}C NMR (101 MHz, $CDCl_3$) δ 157.8, 137.7, 137.1, 134.6, 129.4, 128.7, 115.0, 113.8, 77.1, 58.40, 58.37, 55.4, 38.4, 31.9, 31.5, 31.2, 27.4, 22.6, 18.4, 14.1, –6.2. IR (film): $\tilde{\nu}$ 2958, 2926, 1612, 1512, 1464, 1442, 1299, 1262, 1246, 1175, 1102, 1077, 990, 958, 910, 824, 792 cm^{-1}. HRMS (ESI^+) calcd. for $C_{24}H_{40}O_4SiNa$ $[M+Na]^+$: 443.25881; found: 443.25904.

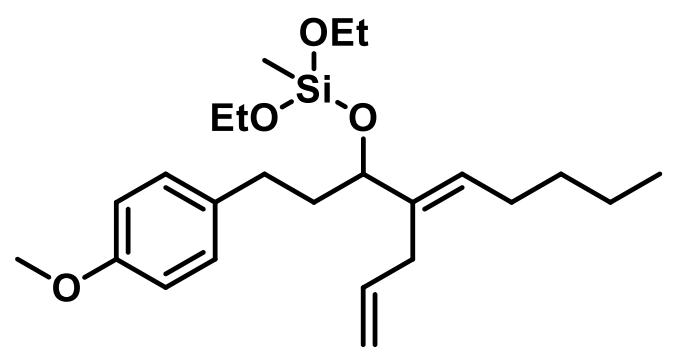

Compound 17. Compound **1** (34.9 mg, 0.0917 mmol) was dissolved in THF (0.9 mL) and the resulting solution was cooled to –30 °C (bath temperature). A solution of freshly prepared LDA (0.1 M in THF, 1.00 mL, 0.100 mmol) was added in a single portion, and the mixture was stirred for 5 min. Copper(I) iodide (21.0 mg, 0.110 mmol) was added, and stirring continued whilst maintaining a reaction temperature of between –30 °C and –25 °C. Once full consumption of the starting material was detected by thin-layer chromatography (approximately 50 min), iodobenzene (0.030 mL, 0.27 mmol) was added, followed by tetrakis(triphenylphosphine)palladium(0) (10.0 mg, 0.00870 mmol). The mixture was stirred for 1 h before being warmed to room temperature over 30 min. The reaction was quenched by the addition of saturated aqueous ammonium chloride solution and the mixture was diluted with water and ethyl acetate. The organic layer was separated, and the aqueous phase was extracted three times with ethyl acetate. The combined organic extracts were washed with brine, dried over anhydrous magnesium sulfate, and filtered. The volatile organics were removed under reduced pressure and the residue was purified *via* flash chromatography (eluting with 0→5% ethyl acetate/cyclohexane) to give the title compound as a colourless oil (33.2 mg, 79%). ^{1}H NMR (400 MHz, $CDCl_3$) δ 7.35 – 7.29 (m, 2H), 7.28 – 7.24 (m, 1H), 7.22 – 7.13 (m, 2H), 7.05 – 6.97 (m, 2H), 6.85 – 6.65 (m, 2H), 5.74 (td, *J* = 7.4, 1.1 Hz, 1H), 4.56 (t, *J* = 6.2 Hz, 1H), 3.91 – 3.80 (m, 4H), 3.76 (s, 3H), 2.71 – 2.57 (m, 1H), 2.52 (ddd, *J* = 13.9, 10.3, 5.9 Hz, 1H), 1.93 (q, *J* = 7.3 Hz, 2H), 1.77 – 1.61 (m, 2H), 1.41 – 1.10 (m, 10H), 0.82 (t, *J* = 7.1 Hz, 3H), 0.17 (s, 3H). ^{13}C NMR (101 MHz, $CDCl_3$) δ 157.8, 142.3, 138.9, 134.6, 129.5, 129.4, 129.1, 128.1, 126.8, 113.8, 76.6, 58.51, 58.49, 55.4, 38.3, 32.2, 30.9, 28.3, 22.4, 18.5, 14.1, –6.2. IR (film): $\tilde{\nu}$ 2957, 2926, 1612, 1512, 1440, 1262, 1246, 1175, 1075, 1040, 985, 957, 823, 790, 768, 703 cm^{-1}. HRMS (ESI$^+$) calcd. for $C_{27}H_{40}O_4SiNa$ [M+Na]$^+$: 479.25881; found: 479.25917.

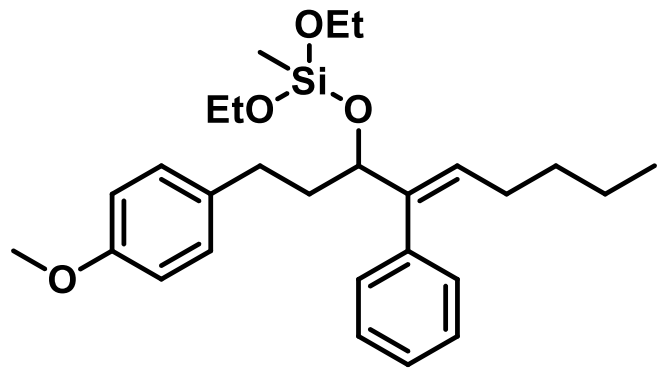

Compound 18. Compound **1** (35.2 mg, 0.0925 mmol) was dissolved in THF (0.9 mL) and the resulting solution was cooled to –30 °C (bath temperature). A solution of freshly prepared LDA (0.1 M in THF, 1.00 mL, 0.100 mmol) was added in a single portion, and the mixture was stirred for 5 min. Copper(I) iodide (21.0 mg, 0.110 mmol) was added and stirring continued whilst maintaining a reaction temperature of between –30 °C and –25 °C. Once full consumption of the starting material was detected by thin-layer chromatography (approximately 50 min), cyclohexenyl triflate (63.0 mg, 0.274 mmol) was added followed by tetrakis(triphenylphosphine)palladium(0) (10.0 mg, 0.00870 mmol). The mixture was stirred for 1 h before being warmed to room temperature over 30 min. The reaction was quenched by the addition of saturated aqueous ammonium chloride solution and the mixture was diluted with water and ethyl acetate. The organic phase was separated, and the aqueous layer was extracted three times with ethyl acetate. The combined organic extracts were washed with brine, dried over anhydrous magnesium sulfate, and filtered. The volatile organics were removed

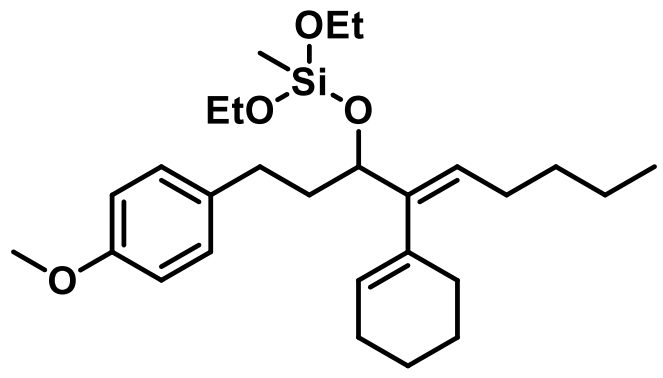

under reduced pressure and the residue was purified *via* flash chromatography (eluting with 0→5% ethyl acetate/cyclohexane) to give the title compound as a colourless oil (34.9 mg, 82%). ^{1}H NMR (400 MHz, $CDCl_3$) δ 7.14 – 7.02 (m, 2H), 6.93 – 6.73 (m, 2H), 5.43 (td, *J* = 7.4, 1.1 Hz, 1H), 5.39 (td, *J* = 3.4, 1.5 Hz, 1H), 4.32 (ddd, *J* = 6.4, 5.2, 1.1 Hz, 1H), 3.89 – 3.71 (m, 7H), 2.57 (dddd, *J* = 34.8, 13.8, 10.8, 6.1 Hz, 2H), 2.17 – 1.95 (m, 5H), 1.91 – 1.68 (m, 2H), 1.67 – 1.57 (m, 4H), 1.41 – 1.25 (m, 5H), 1.25 – 1.15 (m, 6H), 0.99 – 0.82 (m, 3H), 0.12 (s, 3H). ^{13}C NMR (101 MHz, $CDCl_3$) δ 157.7, 144.6, 135.5, 135.0, 129.4, 126.5, 125.9, 113.8, 75.3, 58.44, 58.40, 55.4, 38.4, 32.4, 30.9, 29.7, 28.2, 25.5, 23.2, 22.5, 22.4, 18.4, 14.1, –6.2. IR (film): $\tilde{\nu}$ 2925, 1612, 1512, 1261, 1246, 1175, 1076, 1041, 986, 958, 823, 805 cm^{-1}. HRMS (ESI$^+$) calcd. for $C_{27}H_{44}O_4SiNa$ $[M+Na]^+$: 483.29011; found: 483.29026.

Compound 19. Compound **1** (33.0 mg, 0.0867 mmol) was dissolved in THF (1.0 mL) and the resulting solution was cooled to –30 °C (bath temperature). A solution of freshly prepared LDA (0.1 M in THF, 0.95 mL, 0.095 mmol) was added in a single portion, and the mixture was stirred for five minutes. Tetrakis(acetonitrile) copper(I) tetrafluoroborate (55.2 mg, 0.176 mmol) was added, and stirring was continued whilst maintaining a reaction temperature of between –30 °C and –25 °C. Once full consumption of the starting material was detected by thin-layer chromatography (approximately 50 min), dipyridyl disulfide (40.3 mg, 0.183 mmol) was added in a single portion. The mixture was stirred for 2 h before being warmed to room temperature over a further 2 h. The reaction was quenched by the addition of saturated aqueous ammonium chloride solution and the mixture was diluted with water and ethyl acetate. The organic phase was separated, and the aqueous layer was extracted three times with ethyl acetate. The combined organic extracts were washed with brine, dried over anhydrous magnesium sulfate, and filtered. The volatile organics were removed under reduced pressure and the residue was purified *via* flash chromatography (eluting with 0→35% ethyl acetate/cyclohexane) to give the title compound as a colourless oil (33.5 mg, 79%). ^{1}H NMR (400 MHz, $CDCl_3$) δ 8.38 (ddd, *J* = 4.9, 2.0, 0.9 Hz, 1H), 7.46 (ddd, *J* = 8.1, 7.4, 1.9 Hz, 1H), 7.18 (dt, *J* = 8.1, 1.0 Hz, 1H), 7.11 – 7.04 (m, 2H), 6.97 (ddd, *J* = 7.4, 4.9, 1.1 Hz, 1H), 6.86 – 6.75 (m, 2H), 6.51 (td, *J* = 7.3, 1.1 Hz, 1H), 4.50 (ddd, *J* = 6.9, 4.3, 1.1 Hz, 1H), 3.92 – 3.66 (m, 7H), 2.71 – 2.48 (m, 2H), 2.42 – 2.24 (m, 2H), 2.17 – 2.01 (m, 1H), 1.93 (dddd, *J* = 13.7, 10.6, 6.9, 5.6 Hz, 1H), 1.44 – 1.26 (m, 4H), 1.21 (td, *J* = 7.0, 1.3 Hz, 6H), 0.86 (t, *J* = 7.2 Hz, 3H), 0.14 (s, 3H). ^{13}C NMR (101 MHz, $CDCl_3$) δ 160.3, 157.8, 149.7, 141.0, 136.5, 134.4, 132.5, 129.4, 120.9, 119.6, 113.8, 75.6, 58.5, 55.4, 38.5, 31.3, 30.7, 29.6, 22.5, 18.4, 14.1, –6.3. IR (film): $\tilde{\nu}$ 2956, 2928, 2856, 1612, 1580, 1558, 1512, 1454, 1417, 1300, 1270, 1246, 1177, 1123, 1072, 1038, 822, 763 cm^{-1}. HRMS (ESI$^+$) calcd. for $C_{21}H_{27}O_2NSNa$ $[M+Na]^+$ (desilylated product): 380.16547; found: 380.16525.

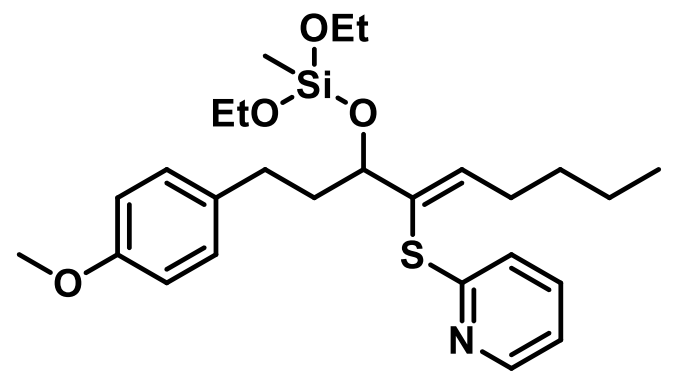

Compound 21. Compound **1** (40.0 mg, 0.105 mmol) was dissolved in THF (1.0 mL) and the resulting

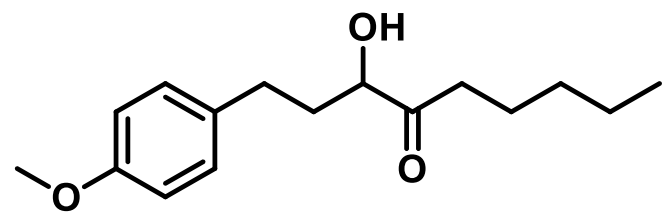

solution was cooled to –30 °C (bath temperature). A solution of freshly prepared LDA (0.1 M in THF, 0.95 mL, 0.095 mmol) was added in a single portion and the solution was stirred for 5 min. Copper(I) bromide dimethyl sulfide complex (43.1 mg, 0.210 mmol) was added, and stirring was continued whilst maintaining a reaction temperature of between –30 °C and –25 °C. Once full consumption of the starting material was detected by thin-layer chromatography (approximately 50 min), the mixture was cooled to –78 °C.

In a separate flask, a solution of *tert*-butyl hydroperoxide (5.5 M in decane, 0.060 mL, 0.33 mmol) was cooled to –78 °C (bath temperature). *n*BuLi (1.6 M in hexane, 0.20 mL, 0.32 mmol) was added dropwise, and the solution was stirred for 30 min. The resulting lithium *tert*-butyl peroxide solution was added to the organocopper solution (both at –78 °C) *via* rapid cannula transfer. The mixture was stirred for 1 h before being warmed to room temperature over a further 2 h. The reaction was quenched by the addition of saturated aqueous ammonium chloride solution and the mixture was diluted with water and ethyl acetate. The organic phase was separated, and the aqueous layer was extracted three times with ethyl acetate. The combined organic extracts were washed with brine, dried over anhydrous magnesium sulfate, and filtered. The volatile organics were removed under reduced pressure and the residue was purified *via* flash chromatography (eluting with 0→40% ethyl acetate/cyclohexane) to give the title compound as a colourless oil (25.3 mg, 91%). ^{1}H NMR (400 MHz, $CDCl_3$) δ 7.20 – 7.04 (m, 2H), 6.89 – 6.76 (m, 2H), 4.13 (dt, *J* = 8.1, 3.8 Hz, 1H), 3.79 (s, 3H), 3.57 (d, *J* = 4.8 Hz, 1H), 2.80 – 2.59 (m, 2H), 2.40 (q, *J* = 7.3 Hz, 2H), 2.09 (dddd, *J* = 13.8, 9.5, 7.3, 3.4 Hz, 1H), 1.76 (dddd, *J* = 13.8, 9.1, 8.4, 5.3 Hz, 1H), 1.59 (p, *J* = 7.5 Hz, 2H), 1.37 – 1.16 (m, 4H), 0.88 (t, *J* = 7.0 Hz, 3H). ^{13}C NMR (101 MHz, $CDCl_3$) δ 212.5, 158.1, 133.3, 129.7, 114.1, 75.7, 55.4, 37.9, 36.0, 31.5, 30.4, 23.4, 22.5, 14.0. IR (film): $\tilde{\nu}$ 3471, 2955, 2930, 2858, 1710, 1611, 1584, 1512, 1464, 1407, 1363, 1300, 1246, 1117, 1035, 939, 834, 780 cm^{-1}. HRMS (ESI^{+}) calcd. for $C_{16}H_{24}O_3Na$ $[M+Na]^{+}$: 287.16176; found: 287.16159.

Compound 22. Compound **1** (33.4 mg, 0.0878 mmol) was dissolved in THF (1.0 mL) and the resulting

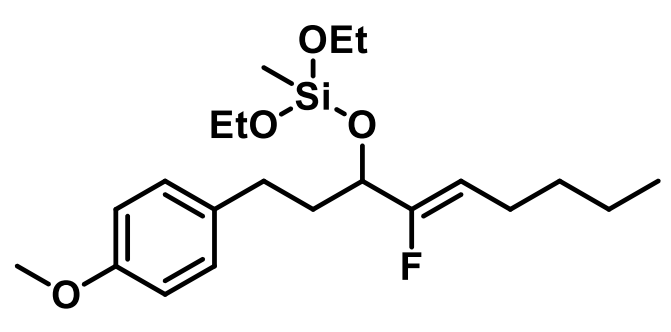

solution was cooled to –30 °C (bath temperature). A solution of freshly prepared LDA (0.1 M in THF, 1.00 mL, 0.100 mmol) was added in a single portion, and the solution was stirred for 5 min. Tetrakis(acetonitrile) copper(I) tetrafluoroborate (54.0 mg, 0.207 mmol) was added and stirring was continued whilst maintaining a reaction temperature of between –30 °C and –25 °C. Once full consumption of the starting material was detected by thin-layer chromatography (approximately 50 min), *N*-fluorocollidinium triflate (**28**) (54.0 mg, 0.187 mmol)[8] was added in a single portion. The mixture was stirred for 1 h before being warmed to room temperature over 30 min. The reaction was quenched by

the addition of saturated aqueous ammonium chloride solution and the mixture was diluted with water and ethyl acetate. The organic phase was separated, and the aqueous layer was extracted three times with ethyl acetate. The combined organic extracts were washed with brine, dried over anhydrous magnesium sulfate, and filtered. The volatile organics were removed under reduced pressure and the residue was purified *via* flash chromatography (eluting with 0→10% ethyl acetate/cyclohexane) to give the title compound as a colourless oil (27.4 mg, 78%). ^{1}H NMR (400 MHz, $CDCl_3$) δ 7.23 – 7.04 (m, 2H), 6.96 – 6.71 (m, 2H), 4.80 (dt, *J* = 37.4, 7.6 Hz, 1H), 4.29 (dt, *J* = 16.9, 6.4 Hz, 1H), 3.99 – 3.69 (m, 7H), 2.71 – 2.52 (m, 2H), 2.15 – 2.04 (m, 2H), 1.96 (td, *J* = 7.9, 6.4 Hz, 2H), 1.41 – 1.29 (m, 4H), 1.21 (td, *J* = 7.0, 1.3 Hz, 6H), 0.98 – 0.80 (m, 3H), 0.14 (s, 3H). ^{13}C NMR (101 MHz, $CDCl_3$) δ 158.8 (d, *J* = 257.2 Hz), 157.9, 134.0, 129.5, 113.9, 107.0 (d, *J* = 13.8 Hz), 70.7 (d, *J* = 31.2 Hz), 58.5, 58.5, 55.4, 36.5, 31.6 (d, *J* = 1.7 Hz), 30.6, 23.1 (d, *J* = 4.6 Hz), 22.4, 18.4, 14.0, –6.4. ^{19}F NMR (282 MHz, $CDCl_3$) δ –126.1. IR (film): $\tilde{\nu}$ 2958, 2927, 1612, 1513, 1263, 1247, 1176, 1102, 1080, 961, 827, 792 cm^{-1}. HRMS (EI$^+$) calcd. for $C_{21}H_{35}O_4FSi$ [M]$^+$: 398.22832; found: 398.22888.

Compound 23. Compound **1** (22.0 mg, 0.0578 mmol) was dissolved in THF (1.0 mL) and the resulting

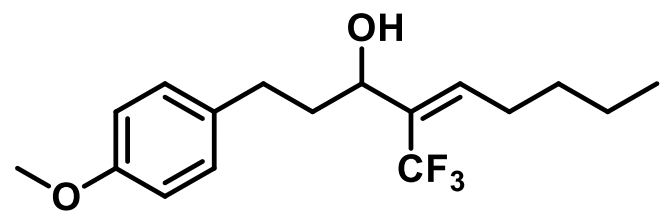

solution was cooled to –30 °C (bath temperature). A solution of freshly prepared LDA (0.1 M in THF, 0.70 mL, 0.070 mmol) was added in a single portion and the resulting solution was stirred for 5 min. Tetrakis(acetonitrile) copper(I) tetrafluoroborate (36.5 mg, 0.116 mmol) was added, and stirring continued whilst maintaining a reaction temperature of between –30 °C and –25 °C. Once full consumption of the starting material was detected by thin-layer chromatography (approximately 50 min), Umemoto's reagent (**29**) (53.0 mg, 0.132 mmol) was added in a single portion. The mixture was stirred for 1 h before being warmed to room temperature over 30 min. The reaction was quenched by the addition of saturated aqueous ammonium chloride solution and the mixture was diluted with water and ethyl acetate. The organic phase was separated, and the aqueous layer was extracted three times with ethyl acetate. The combined organic extracts were washed with brine, dried over anhydrous magnesium sulfate, and filtered. The volatile organics were removed under reduced pressure and the crude residue was redissolved in THF (1.0 mL).

Aqueous HCl (1.0 M, 0.5 mL, 0.5 mmol) was added, and the mixture was stirred for 5 min at room temperature. The reaction was quenched by the careful addition of saturated aqueous sodium bicarbonate solution and the mixture was diluted with water and ethyl acetate. The organic phase was separated, and the aqueous layer was extracted three times with ethyl acetate. The combined organic extracts were washed with brine, dried over anhydrous magnesium sulfate, and filtered. The volatile organics were removed under reduced pressure and the residue was purified *via* flash chromatography (eluting with 0→25% ethyl acetate/cyclohexane) to give the title compound as a colourless oil (8.9 mg,

49%). ^{1}H NMR (600 MHz, $CDCl_3$) δ 7.16 – 7.05 (m, 2H), 6.87 – 6.79 (m, 2H), 6.09 (ddt, *J* = 7.8, 7.0, 0.9 Hz, 1H), 4.29 (dd, *J* = 8.4, 4.2 Hz, 1H), 3.79 (s, 3H), 2.73 (ddd, *J* = 13.9, 9.8, 5.5 Hz, 1H), 2.63 (ddd, *J* = 13.9, 9.6, 6.6 Hz, 1H), 2.40 – 2.23 (m, 2H), 2.01 – 1.91 (m, 1H), 1.91 – 1.79 (m, 1H), 1.68 (s, 1H), 1.47 – 1.38 (m, 2H), 1.37 – 1.29 (m, 2H), 0.91 (t, *J* = 7.3 Hz, 3H). ^{13}C NMR (151 MHz, $CDCl_3$) δ 158.0, 139.0 (q, *J* = 3.6 Hz), 133.5, 132.4 (q, *J* = 26.7 Hz), 129.5, 124.5 (q, *J* = 276.9 Hz), 114.0, 70.6 (q, *J* = 2.4 Hz), 55.4, 38.5, 31.4, 31.2, 28.0 (q, *J* = 1.3 Hz), 22.4, 13.9. ^{19}F NMR (565 MHz, $CDCl_3$) δ –58.4. IR (film): $\tilde{\nu}$ 3456, 2958, 2930, 2862, 1612, 1512, 1466, 1380, 1334, 1300, 1247, 1208, 1160, 1118, 1037, 824 cm^{-1}. HRMS (EI$^+$) calcd. for $C_{17}H_{23}O_2F_3$ [M]$^+$: 316.16447; found: 316.16465.

Compound 24. Compound **1** (34.0 mg, 0.0893 mmol) was dissolved in THF (1.0 mL) and the resulting

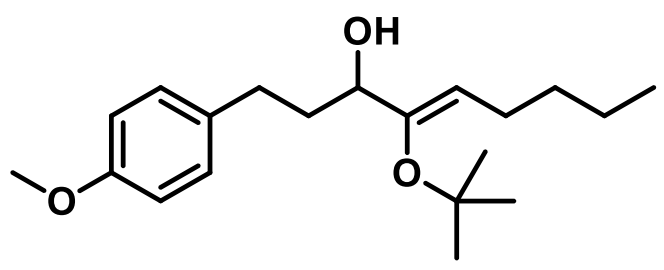

solution was cooled to –30 °C (bath temperature). A solution of freshly prepared LDA (0.1 M in THF, 1.00 mL, 0.100 mmol) was added in a single portion, and the solution was stirred for 5 min. Tetrakis(acetonitrile) copper(I) tetrafluoroborate (56.1 mg, 0.179 mmol) was added and stirring was continued whilst maintaining a reaction temperature of between –30 °C and –25 °C. Once full consumption of the starting material was detected by thin-layer chromatography (approximately 50 min), *tert*-butyl perbenzoate (0.034 mL, 0.182 mmol) was added in a single portion. The mixture was stirred for 1 h before being warmed to room temperature over 30 min. The reaction was quenched by the addition of saturated aqueous ammonium chloride solution and the mixture was diluted with water and ethyl acetate. The organic phase was separated, and the aqueous layer was extracted three times with ethyl acetate. The combined organic extracts were washed with brine, dried over anhydrous magnesium sulfate, and filtered.

The volatile organics were removed under reduced pressure, the crude residue was redissolved in 1:4 THF/MeOH (1 mL), and potassium carbonate (29 mg, 0.209 mmol) was added. The mixture was stirred for 5 min before the reaction was quenched by the careful addition of saturated aqueous ammonium chloride solution. The mixture was diluted with water and ethyl acetate, the organic phase was separated, and the aqueous layer was extracted three times with ethyl acetate. The combined organic extracts were washed with brine, dried over anhydrous magnesium sulfate, and filtered. The volatile organics were removed under reduced pressure and the residue was purified *via* flash chromatography (eluting with 0→35% ethyl acetate/cyclohexane) to give the title compound as a colourless oil (21.7 mg, 76%). ^{1}H NMR (400 MHz, $CDCl_3$) δ 7.18 – 7.09 (m, 2H), 6.87 – 6.77 (m, 2H), 5.16 (td, *J* = 7.1, 1.0 Hz, 1H), 4.04 (d, *J* = 8.2 Hz, 1H), 3.79 (s, 3H), 2.76 (ddd, *J* = 14.5, 9.6, 5.3 Hz, 1H), 2.63 (ddd, *J* = 13.8, 9.4, 7.0 Hz, 1H), 2.14 – 1.96 (m, 3H), 1.84 – 1.71 (m, 2H), 1.38 – 1.29 (m, 4H), 1.27 (s, 9H), 0.93 – 0.84 (m, 3H). ^{13}C NMR (101 MHz, $CDCl_3$) δ 157.9, 153.5, 134.3, 129.6, 116.2, 113.9, 78.2, 72.2, 55.4, 37.3, 31.9, 31.2, 29.7, 25.9, 22.7, 14.1. IR (film): $\tilde{\nu}$ 3472, 2954, 2926, 2856, 1710, 1612, 1512, 1464, 1402, 1376,

1300, 1246, 1178, 1094, 1037, 825 cm^{-1}. HRMS (EI$^+$) calcd. for $C_{16}H_{24}O_3$ [M]$^+$ (*hydrolysis product*): 264.17200; found: 264.17208

Compound 25. Compound **1** (19.7 mg, 0.0518 mmol) was dissolved in THF (1.0 mL) and the resulting solution was cooled to –30 °C (bath temperature). A solution of freshly prepared LDA (0.1 M in THF, 0.6 mL, 0.060 mmol) was added in a single portion and the solution was stirred for 5 min. Copper(I) iodide (20.3 mg, 0.107 mmol) was added and stirring was continued whilst maintaining a reaction temperature of between –30 °C and –25 °C. Once full consumption of the starting material was detected by thin-layer chromatography (approximately 50 min), a solution of iodine (26.0 mg, 0.104 mmol) in THF (1.0 mL) was added in a single portion. The mixture was stirred for a further 5 min before being warmed to room temperature over 30 min. The reaction was quenched by the addition of saturated aqueous ammonium chloride solution and saturated aqueous sodium thiosulfate solution. The mixture was diluted with water and ethyl acetate, the organic phase was separated, and the aqueous layer was extracted three times with ethyl acetate. The combined organic extracts were washed with brine, dried over anhydrous magnesium sulfate, and filtered. The volatile organics were removed under reduced pressure and the residue was purified *via* flash chromatography (eluting with 0→10% ethyl acetate/cyclohexane) to give the title compound as a colourless oil (23.1 mg, 88%). ^{1}H NMR (400 MHz, $CDCl_3$) δ 7.16 – 7.03 (m, 2H), 6.88 – 6.75 (m, 2H), 4.04 – 3.89 (m, 1H), 3.88 – 3.66 (m, 7H), 2.58 – 2.45 (m, 2H), 2.19 (q, *J* = 7.0 Hz, 2H), 1.99 – 1.85 (m, 2H), 1.48 – 1.30 (m, 4H), 1.21 (td, *J* = 7.0, 3.1 Hz, 6H), 0.92 (t, *J* = 7.1 Hz, 3H), 0.12 (s, 3H). ^{13}C NMR (101 MHz, $CDCl_3$) δ 157.9, 136.5, 133.9, 129.4, 114.7, 113.9, 77.8, 58.62, 58.56, 55.4, 39.3, 35.4, 30.51, 30.45, 22.4, 18.4, 14.1, –6.0. IR (film): $\tilde{\nu}$ 2956, 2927, 2858, 1612, 1512, 1464, 1300, 1246, 1176, 1077, 1039, 961, 827, 792 cm^{-1}. HRMS (ESI$^+$) calcd. for $C_{21}H_{35}O_4SiINa$ [M+Na]$^+$: 529.12416; found: 529.12415.

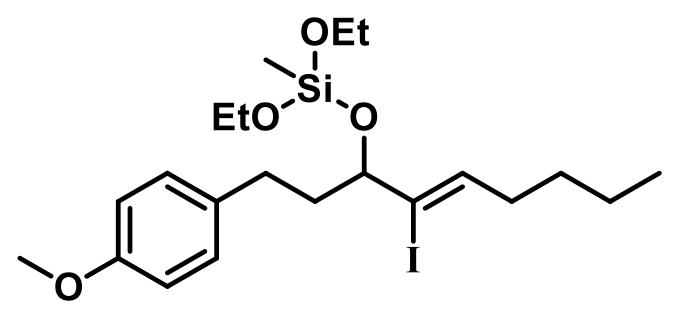

Compound 26. Boc-NH-OAc (31.9 mg, 0.183 mmol) was dissolved in THF (1.0 mL) and the resulting solution was cooled to –78 °C (bath temperature). *n*-Butyllithium (1.6 M in hexanes, 0.11 mL, 0.174 mmol) was added dropwise and the resulting solution was stirred at –78 °C for 1 h to yield a solution of the lithium nitrenoid reagent Boc-NLi-OAc (**27**), which was kept at –78 °C.

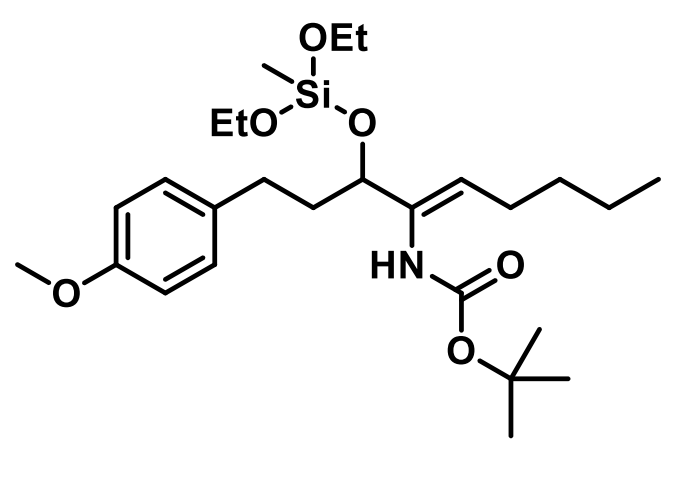

In a separate flask, compound **1** (39.0 mg, 0.103 mmol) was dissolved in THF (1.0 mL) and the resulting solution was cooled to –30 °C (bath temperature). A solution of freshly prepared LDA (0.1 M in THF, 1.10 mL, 0.110 mmol) was added in a single portion, and the solution was stirred for 5 min. Copper(I) iodide (21.1 mg, 0.110 mmol) was then added and stirring continued whilst maintaining a reaction temperature of between –30 °C and –25 °C. Once full consumption of the starting material was detected by thin-layer chromatography (approximately 50 min), the mixture was cooled to –78 °C

(bath temperature). The solution of Boc-NLi-OAc was added *via* rapid cannula transfer and stirring continued for 1 h at −78 °C before the mixture was warmed to room temperature. The reaction was quenched by the addition of saturated aqueous ammonium chloride solution. The mixture was diluted with water and ethyl acetate, the organic phase was separated, and the aqueous layer was extracted three times with ethyl acetate. The combined organic extracts were washed with brine, dried over anhydrous magnesium sulfate, and filtered. The volatile organics were removed under reduced pressure and the residue was purified *via* flash chromatography (eluting with 0→25% ethyl acetate/cyclohexane) to give the title compound as a colourless oil (24.9 mg, 49%). ^{1}H NMR (400 MHz, $CDCl_3$) δ 7.13 – 7.03 (m, 2H), 6.86 – 6.71 (m, 2H), 5.85 (s, 1H), 5.24 (t, *J* = 7.0 Hz, 1H), 4.27 (t, *J* = 6.7 Hz, 1H), 3.88 – 3.66 (m, 7H), 2.68 – 2.43 (m, 2H), 2.20 – 1.95 (m, 2H), 1.93 – 1.75 (m, 2H), 1.46 (s, 9H), 1.38 – 1.27 (m, 4H), 1.26 – 1.12 (m, 6H), 1.00 – 0.80 (m, 3H), 0.11 (s, 3H). ^{13}C NMR (101 MHz, $CDCl_3$) δ 157.8, 153.7, 134.2, 133.0, 129.5, 125.1, 113.8, 79.6, 75.5, 58.5, 55.4, 37.3, 31.2, 30.8, 28.4, 27.3, 22.6, 18.3, 14.1, −6.5. IR (film): $\tilde{\nu}$ 2970, 1929, 1729, 1512, 1482, 1468, 1366, 1244, 1162, 1074, 1040, 957, 823, 790 cm^{-1}. HRMS (ESI^+) calcd. for $C_{26}H_{45}O_6NSiNa$ $[M+Na]^+$: 518.29084; found: 518.29084.

NMR Spectra of New Compounds

Compound S1

1H, CDCl3, 400 MHz

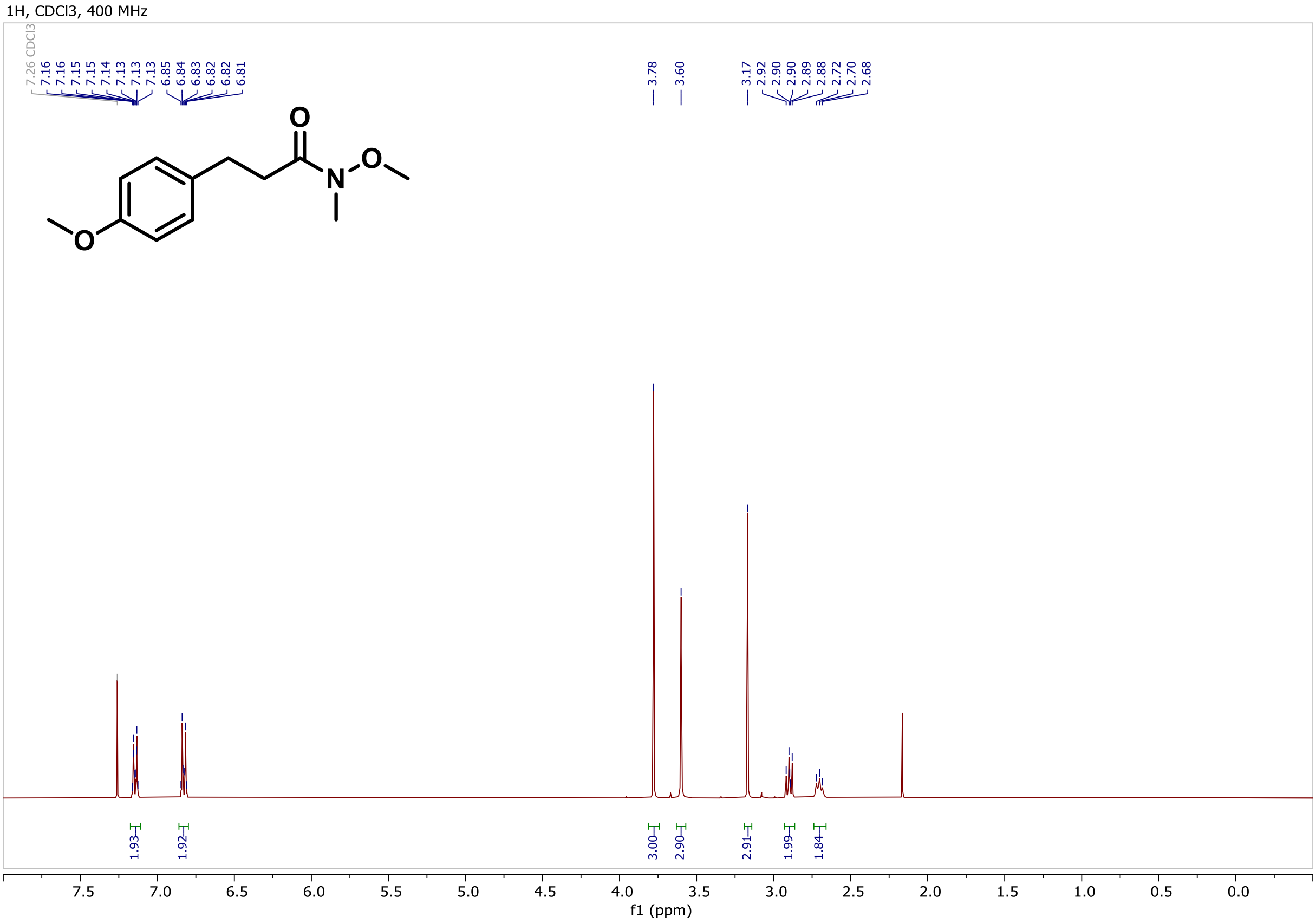

13C, CDCl3, 101 MHz

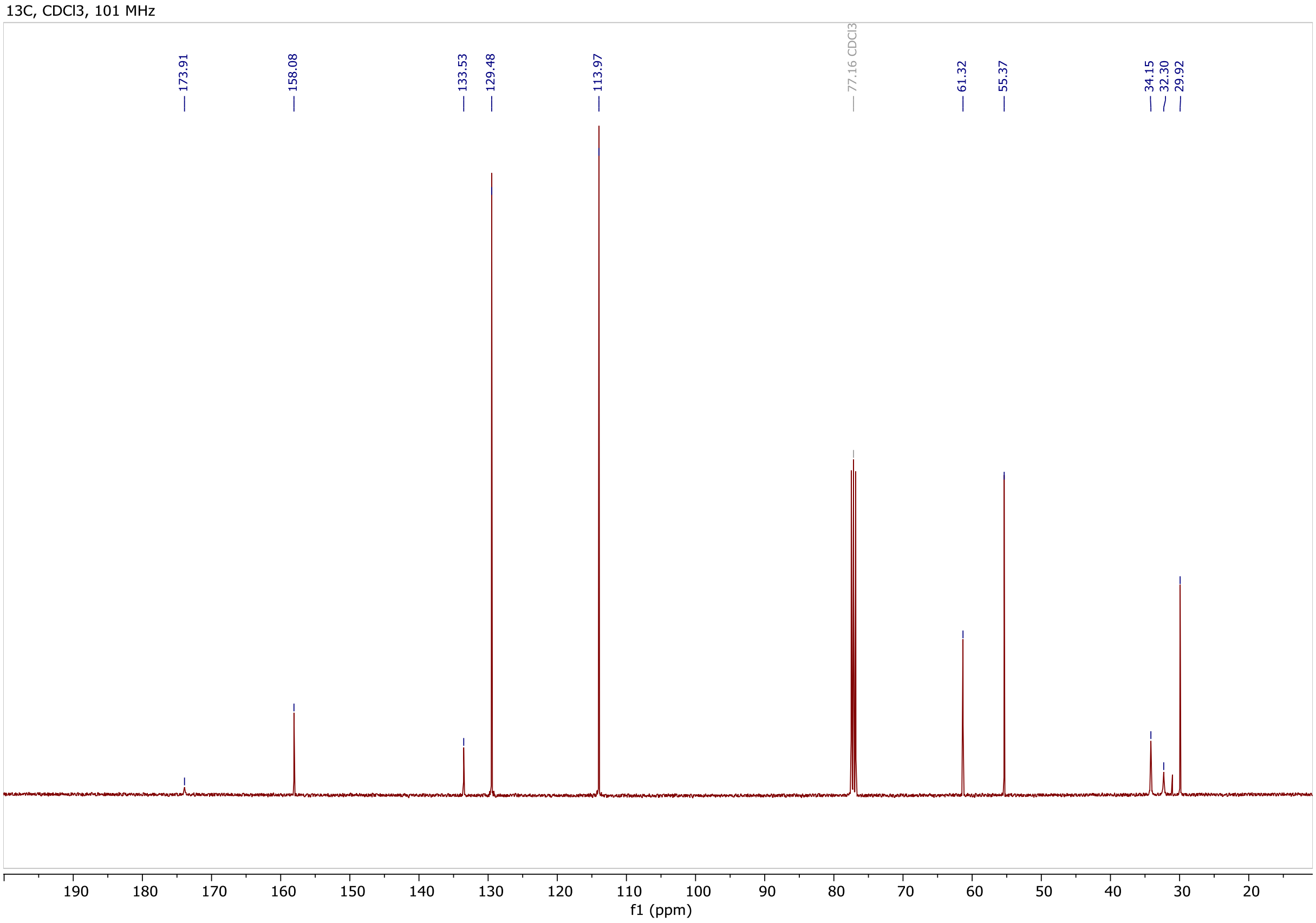

Compound S2

1H, CDCl3, 400 MHz

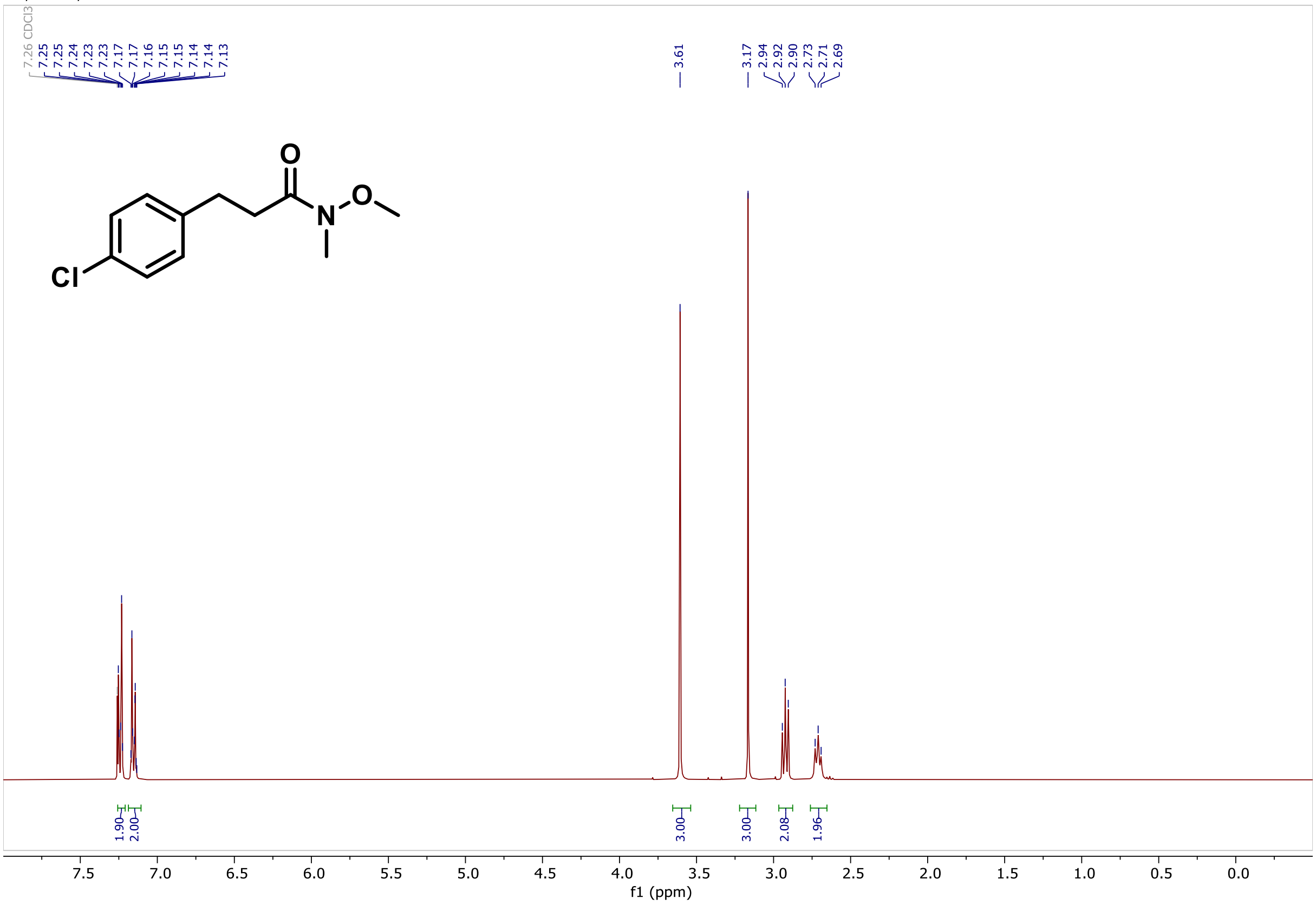

13C, CDCl3, 101 MHz

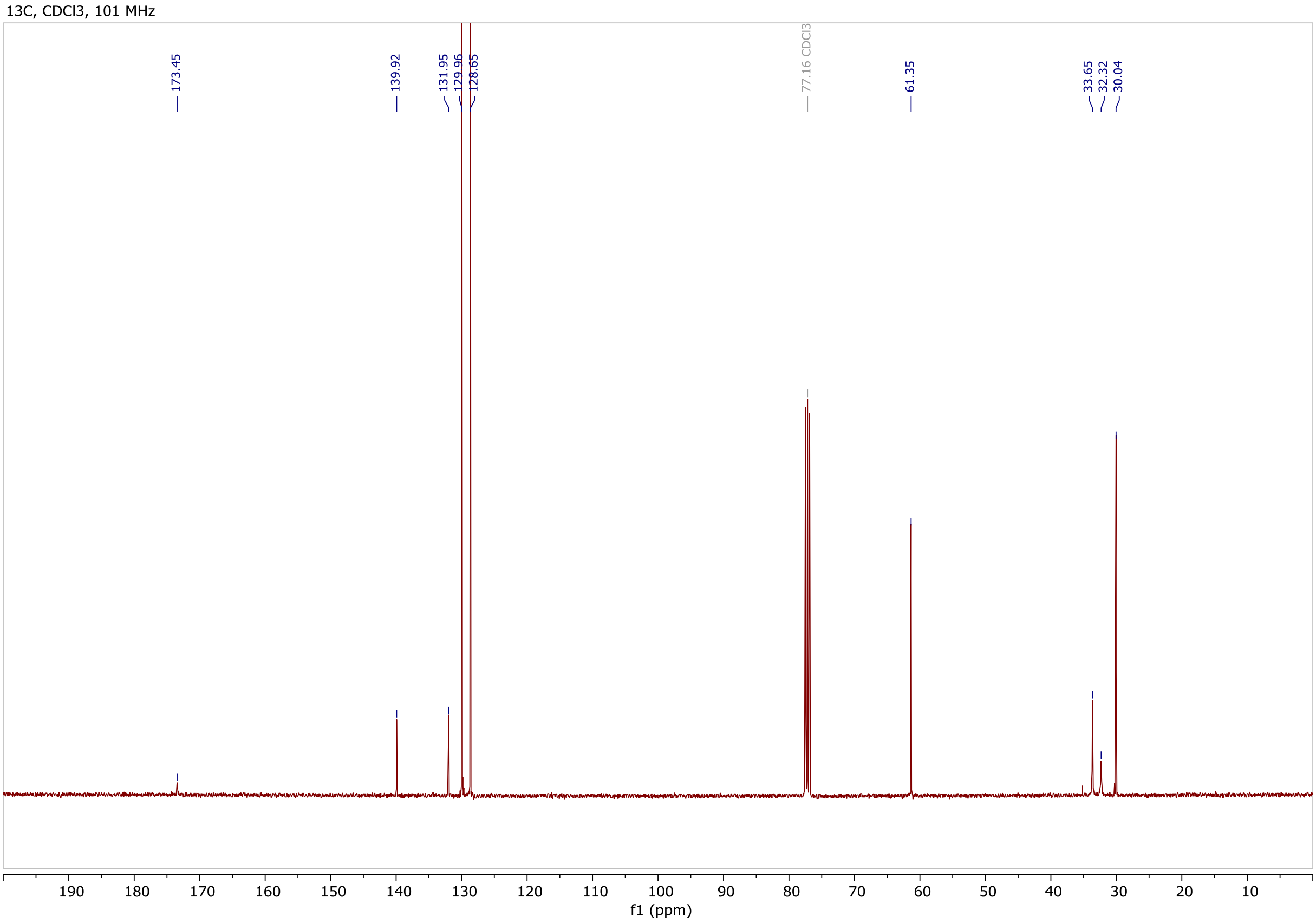

Compound S3

1H, CDCl3, 400 MHz

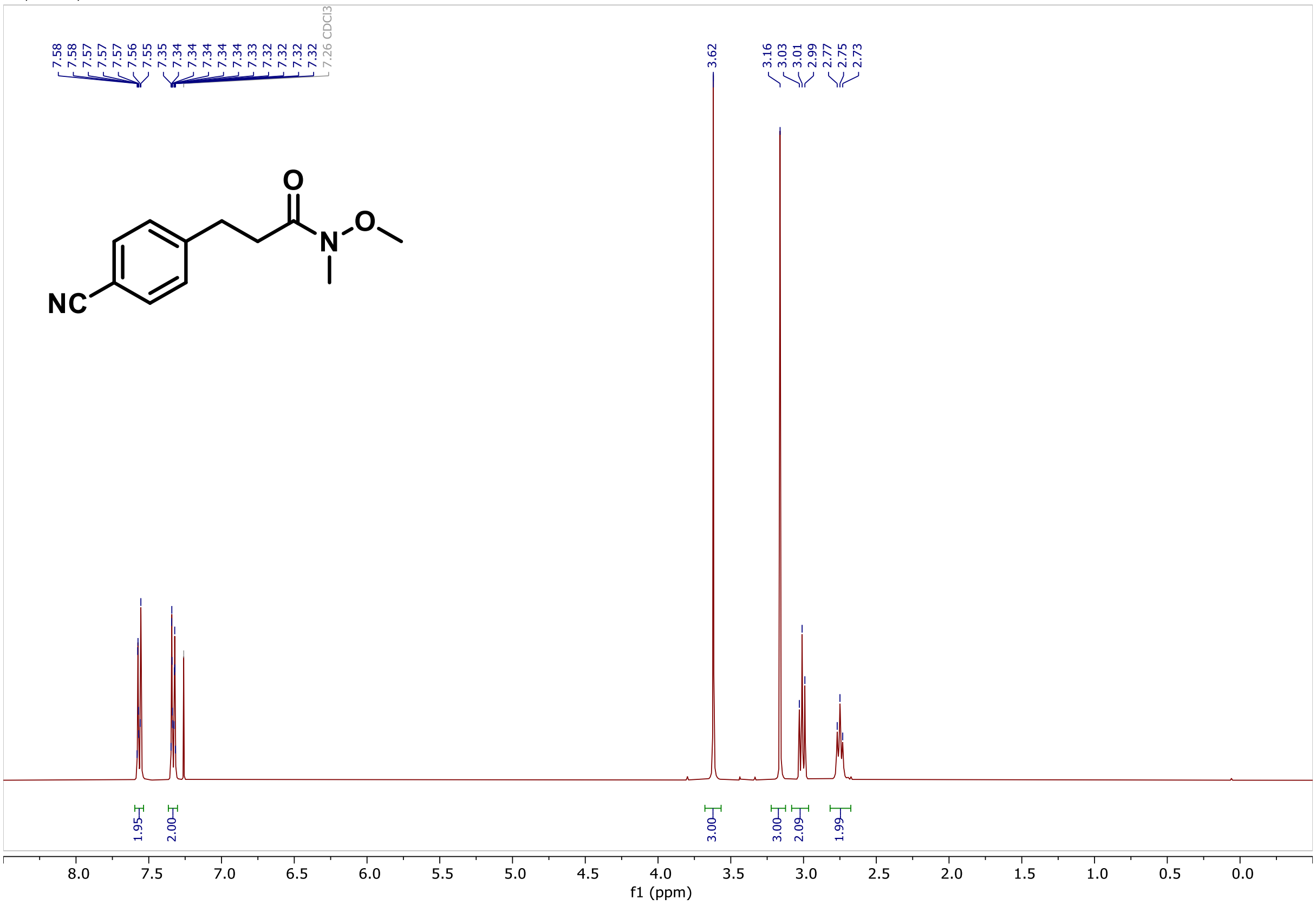

13C, CDCl3, 101 MHz

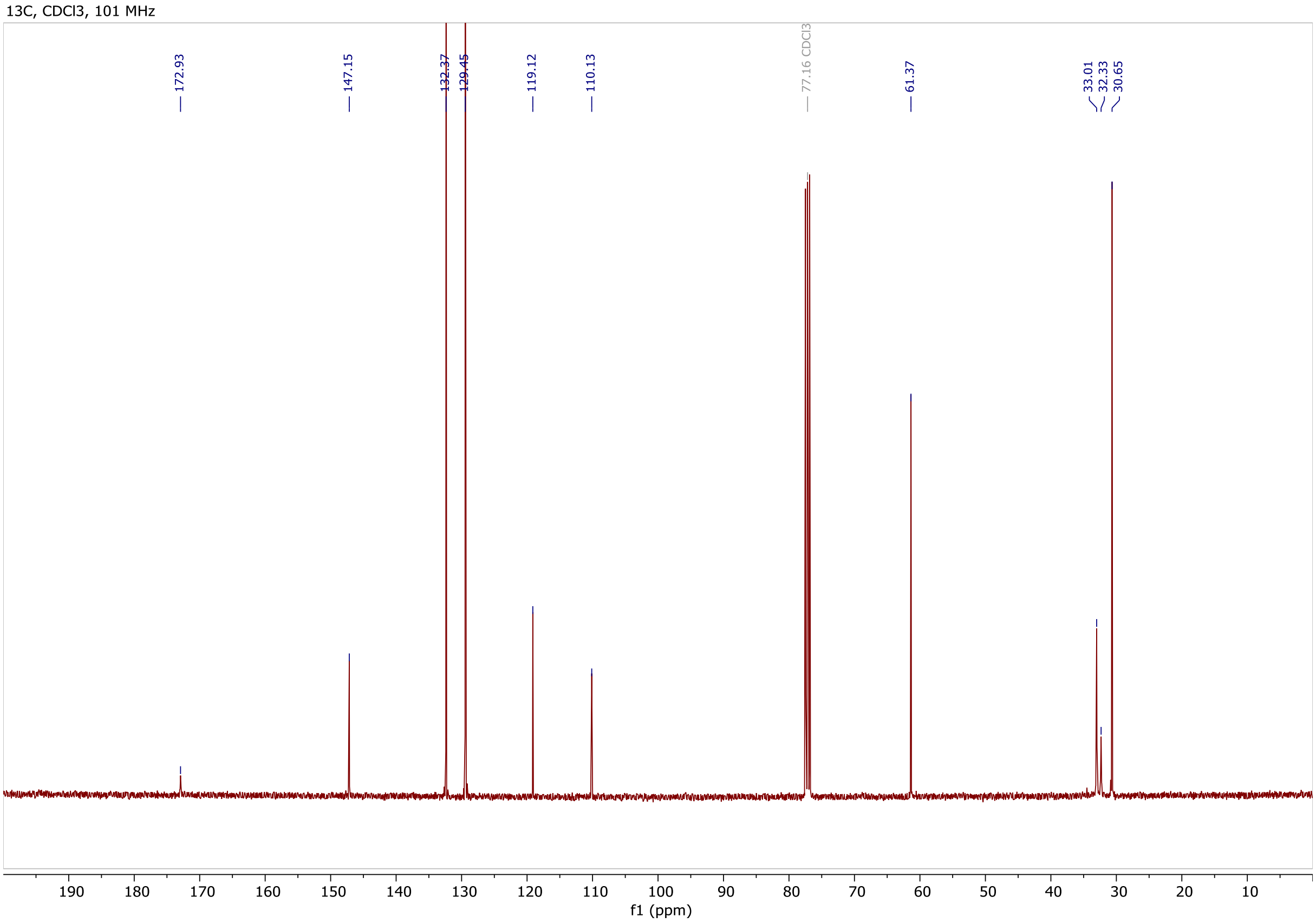

Compound S4

1H, CDCl3, 400 MHz

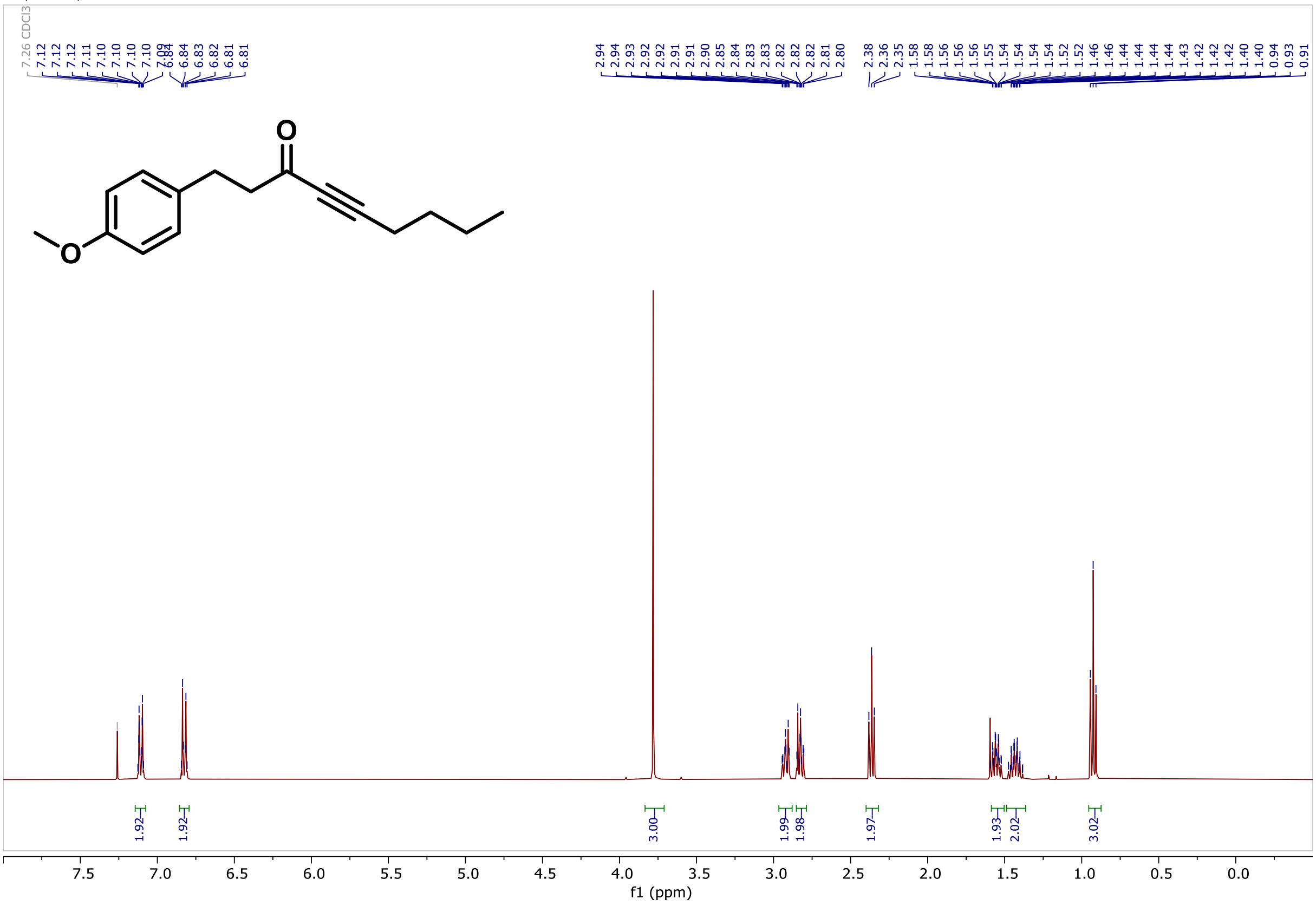

13C, CDCl3, 101 MHz

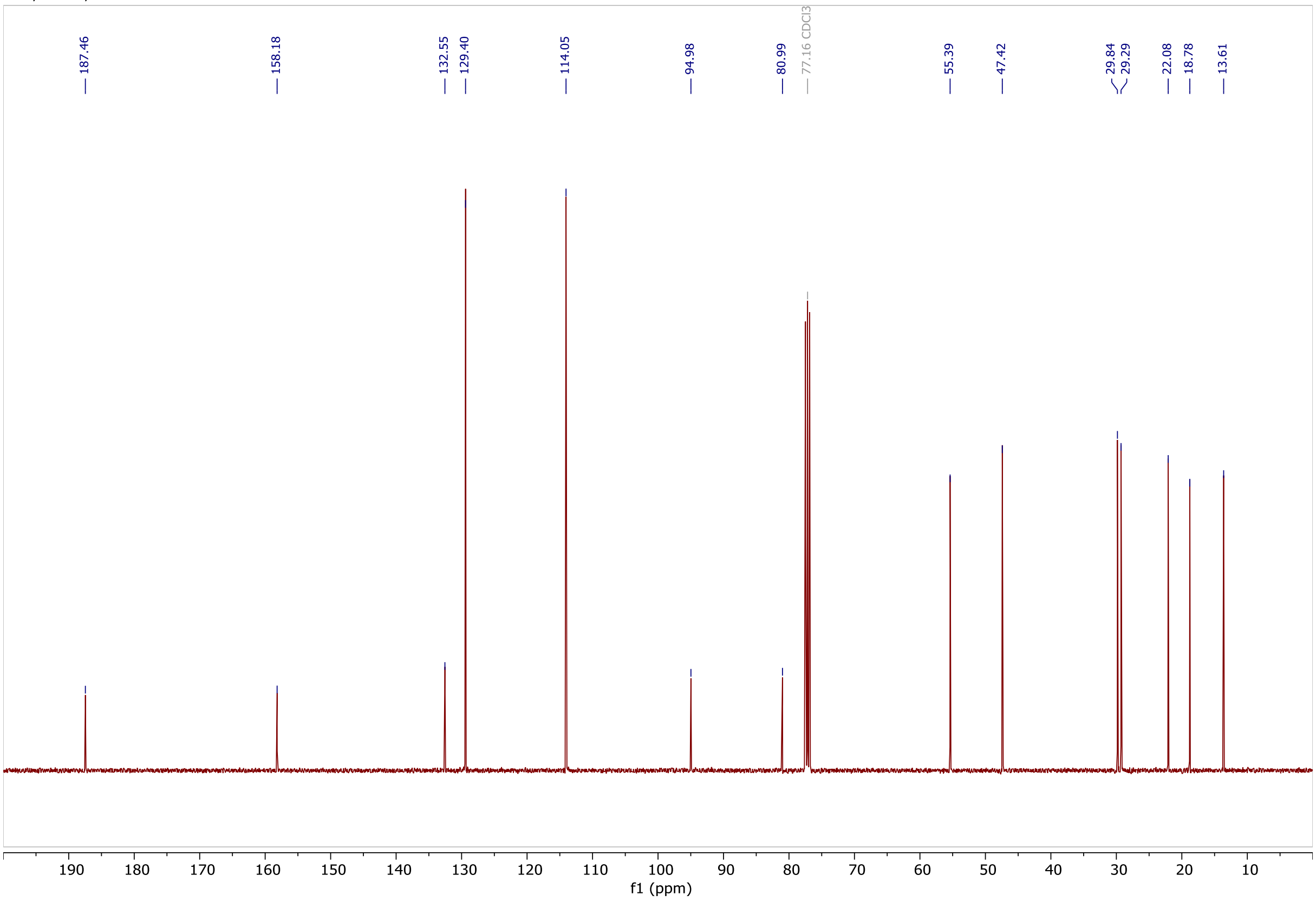

Compound S5

1H, CDCl3, 400 MHz

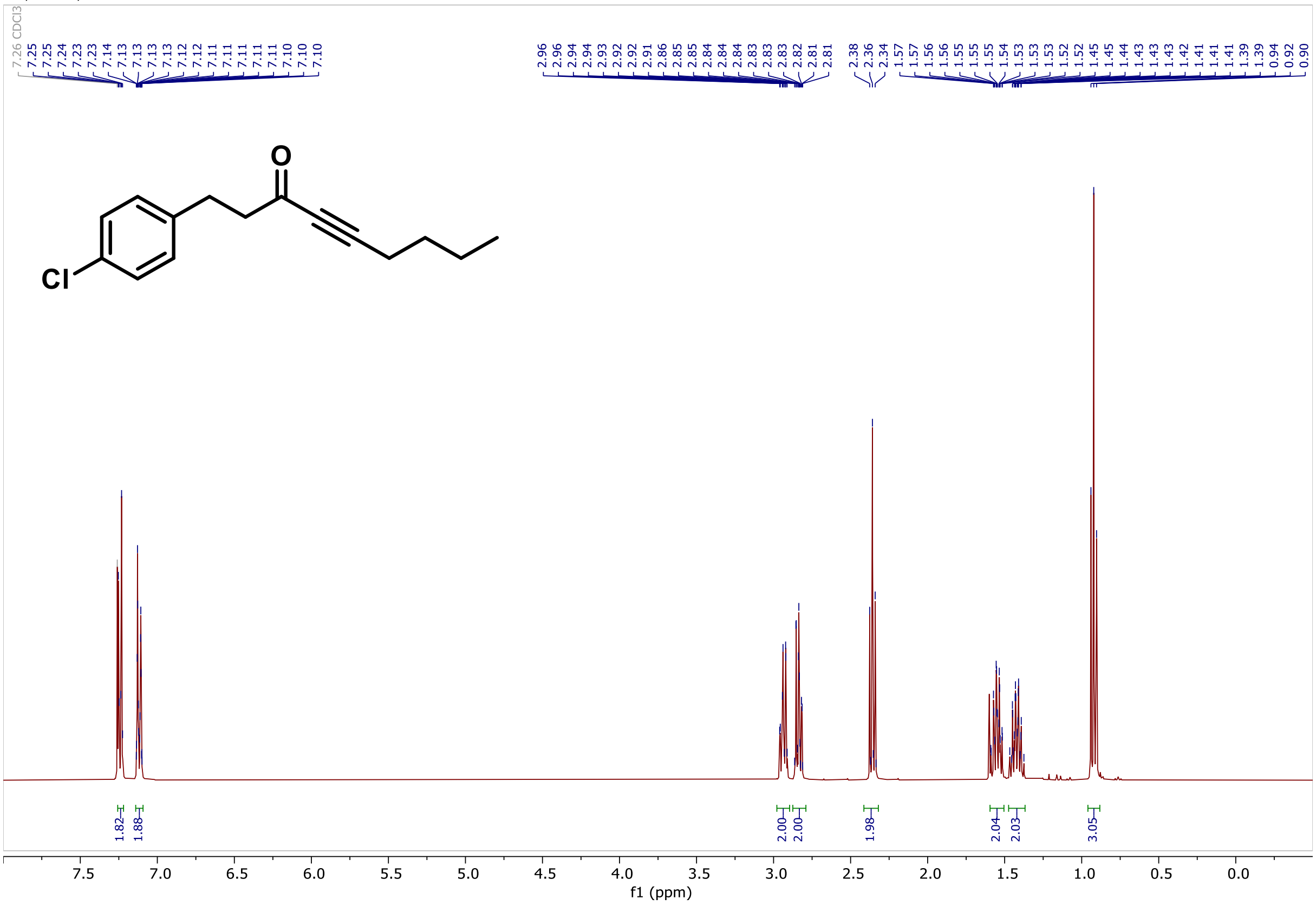

13C, CDCl3, 101 MHz

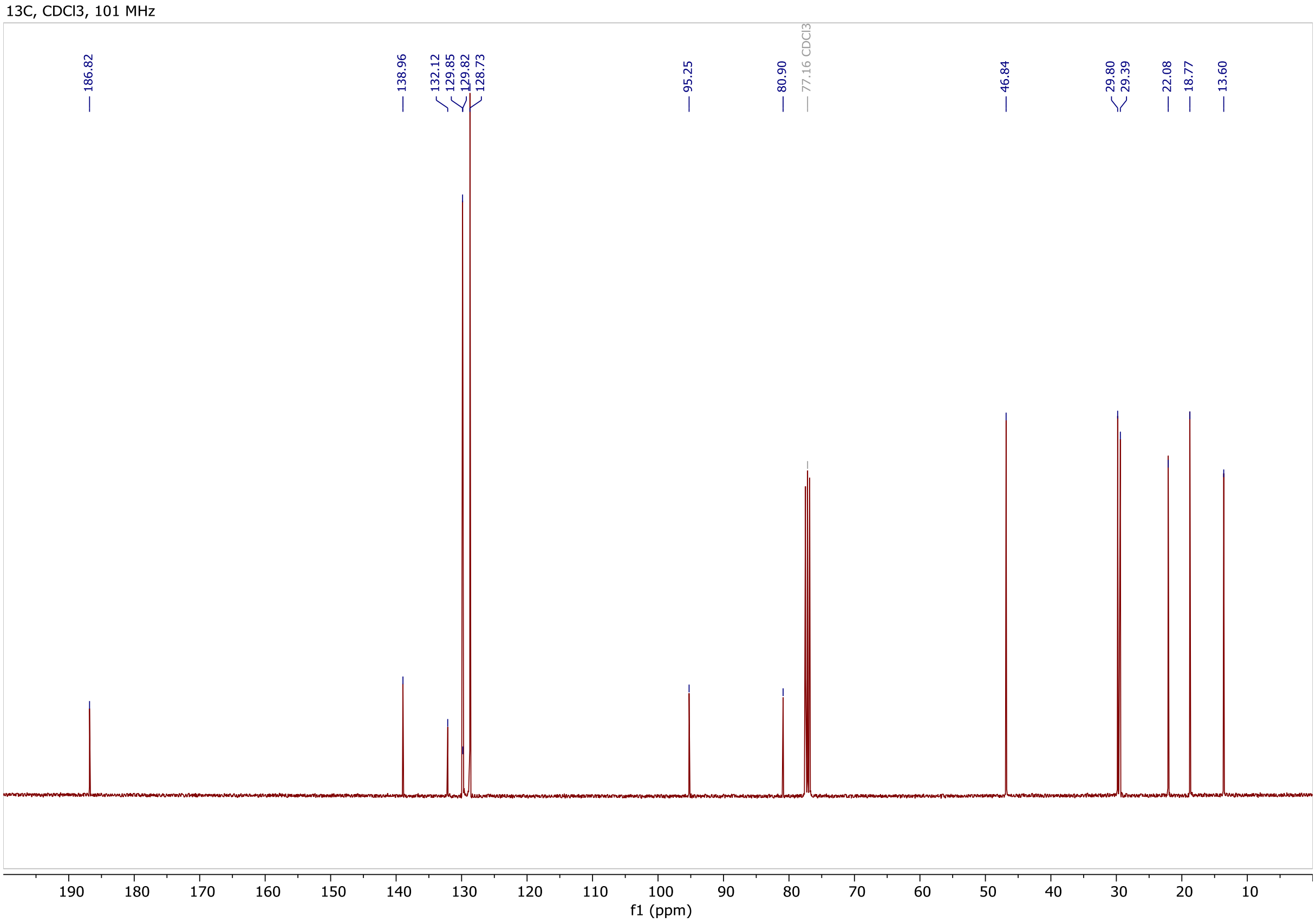

Compound S6

1H, CDCl3, 400 MHz

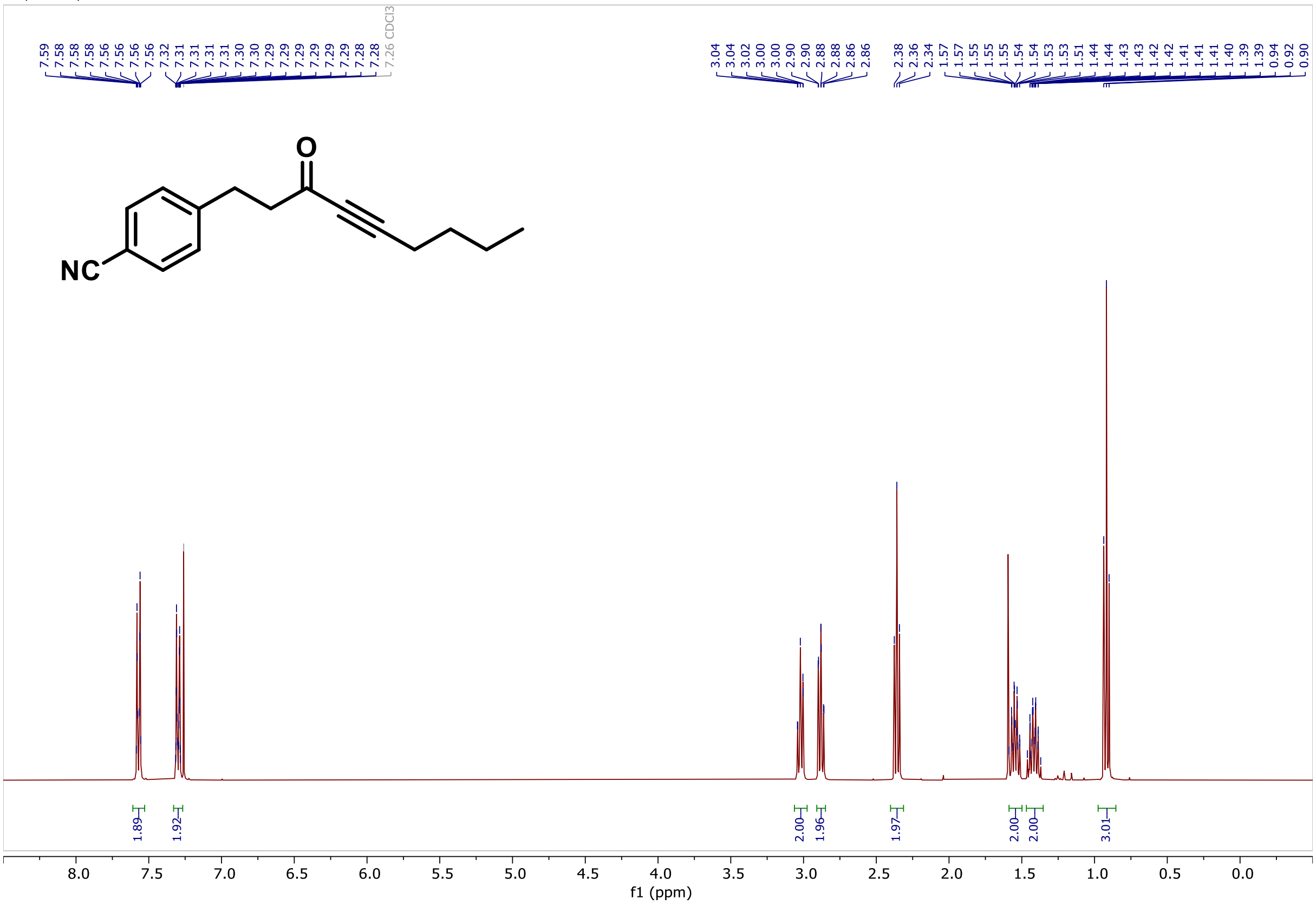

13C, CDCl3, 101 MHz

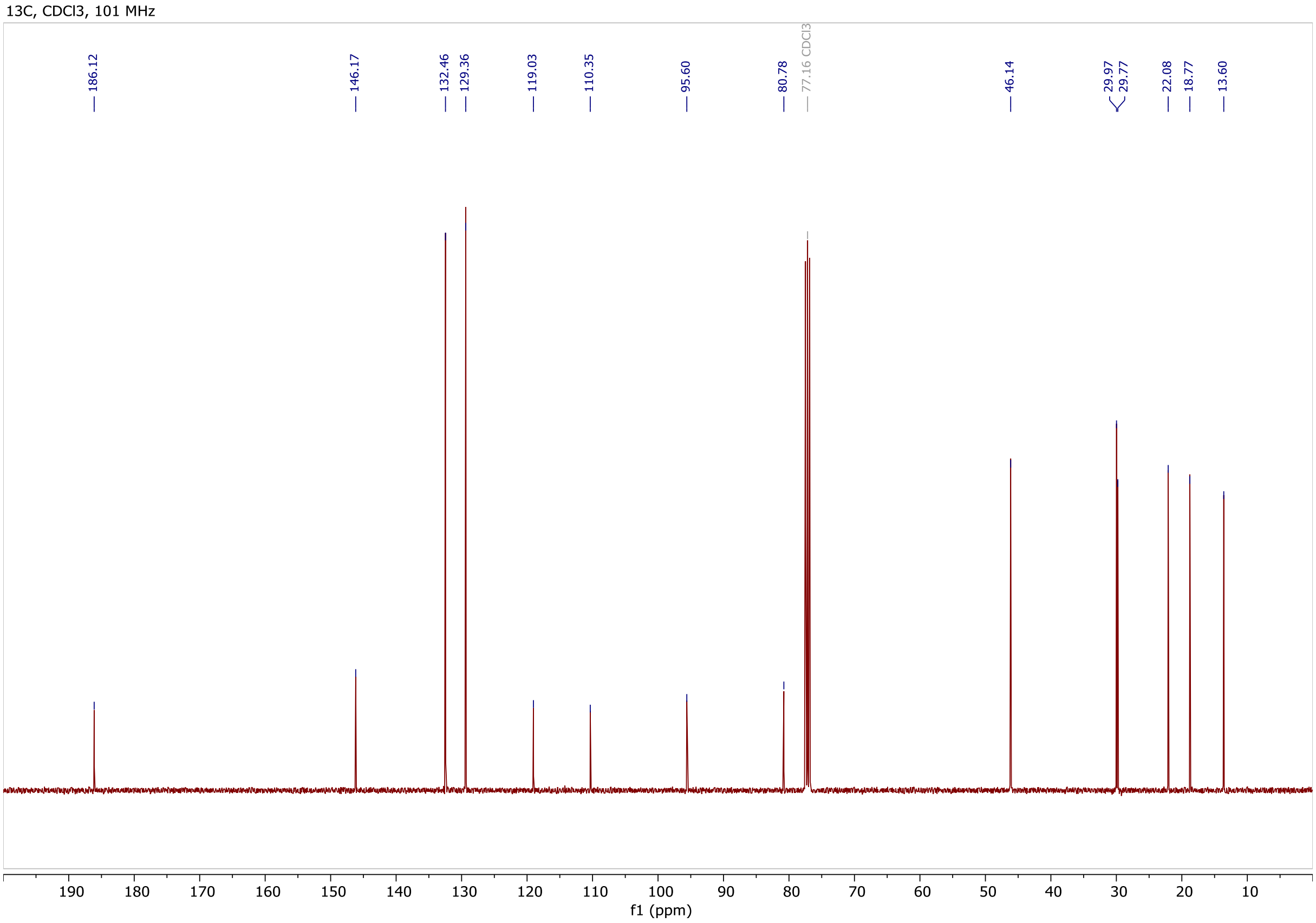

Compound S7

1H, CDCl3, 400 MHz

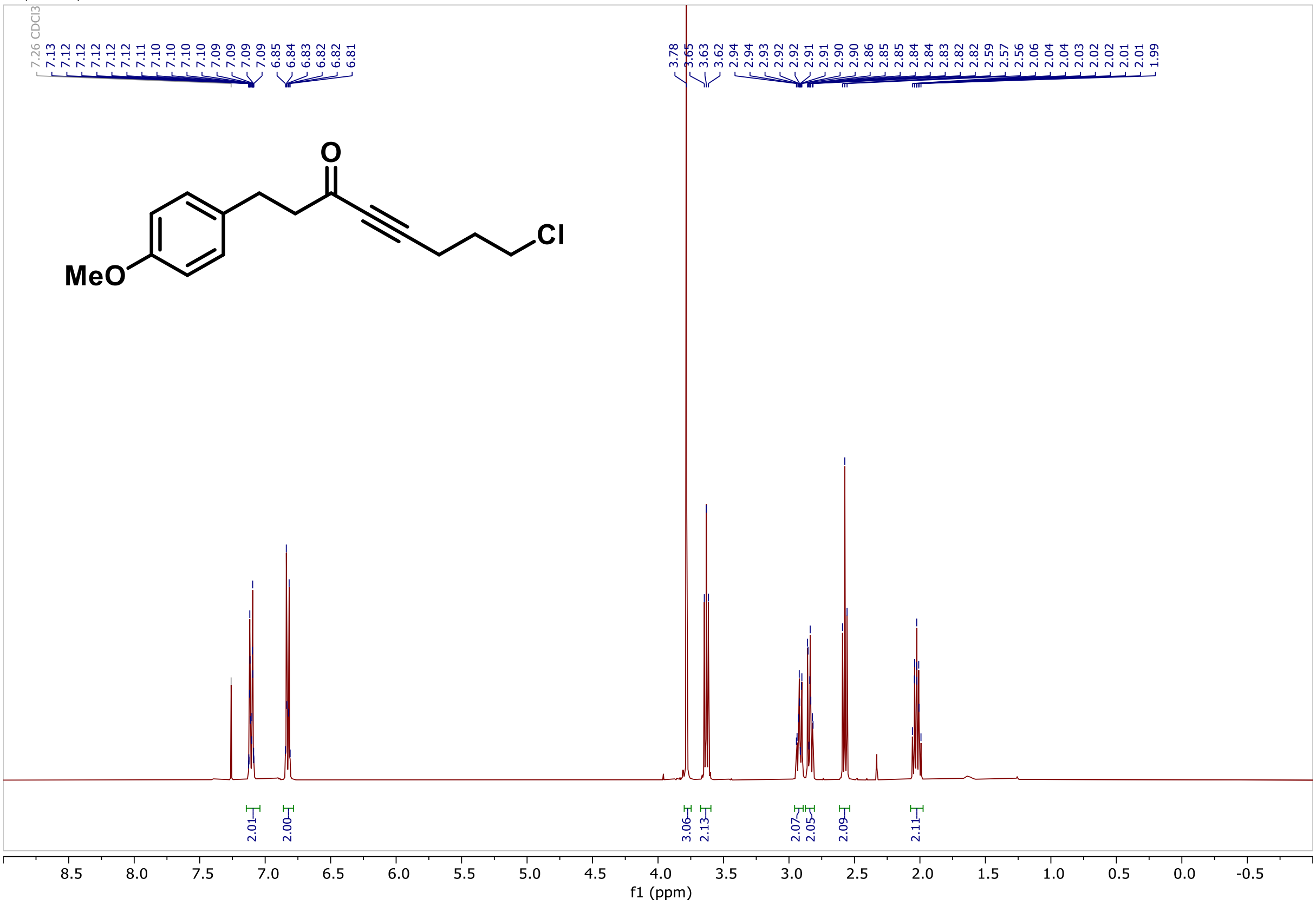

13C, CDCl3, 101 MHz

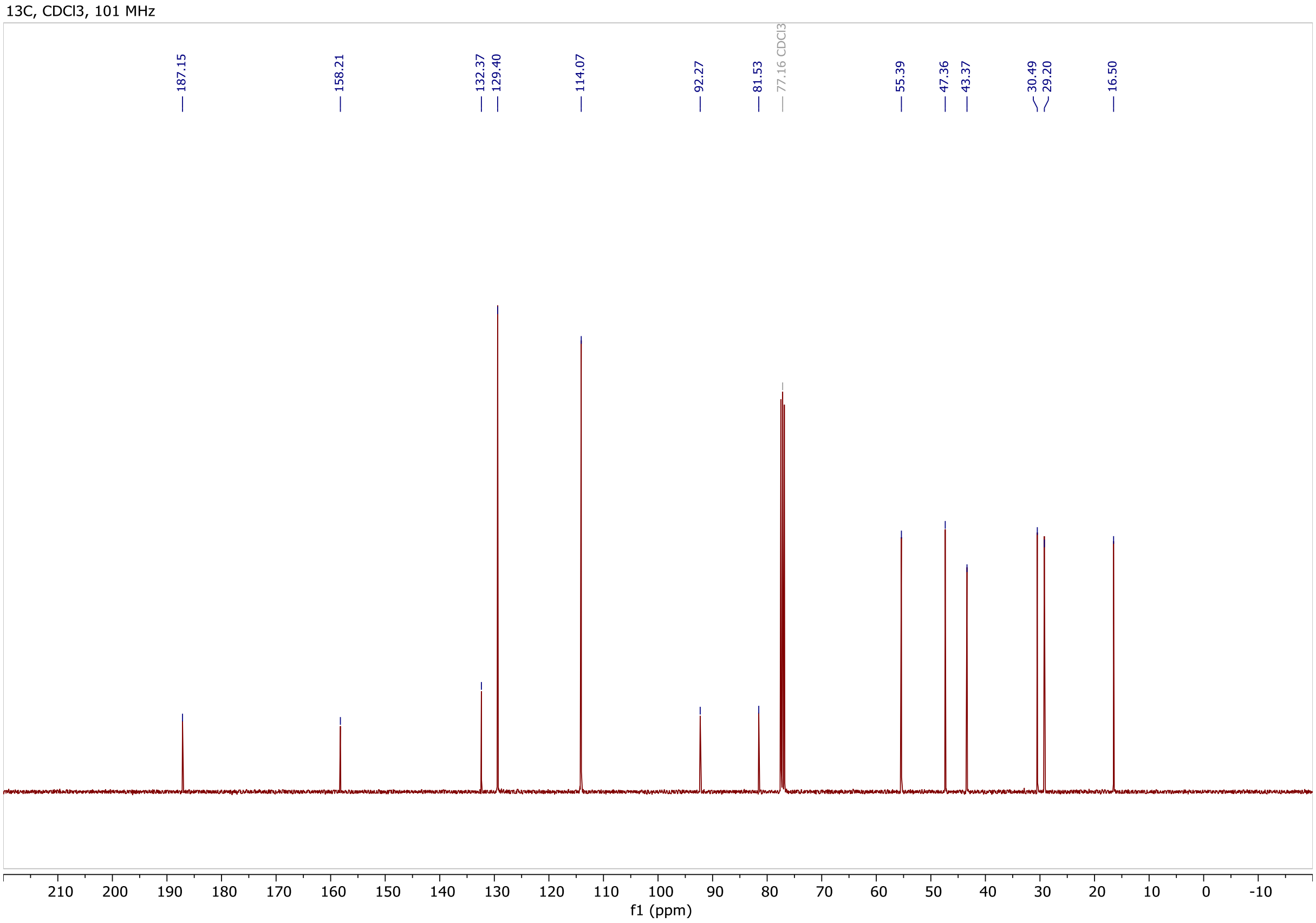

Compound S8

1H, CDCl3, 400 MHz

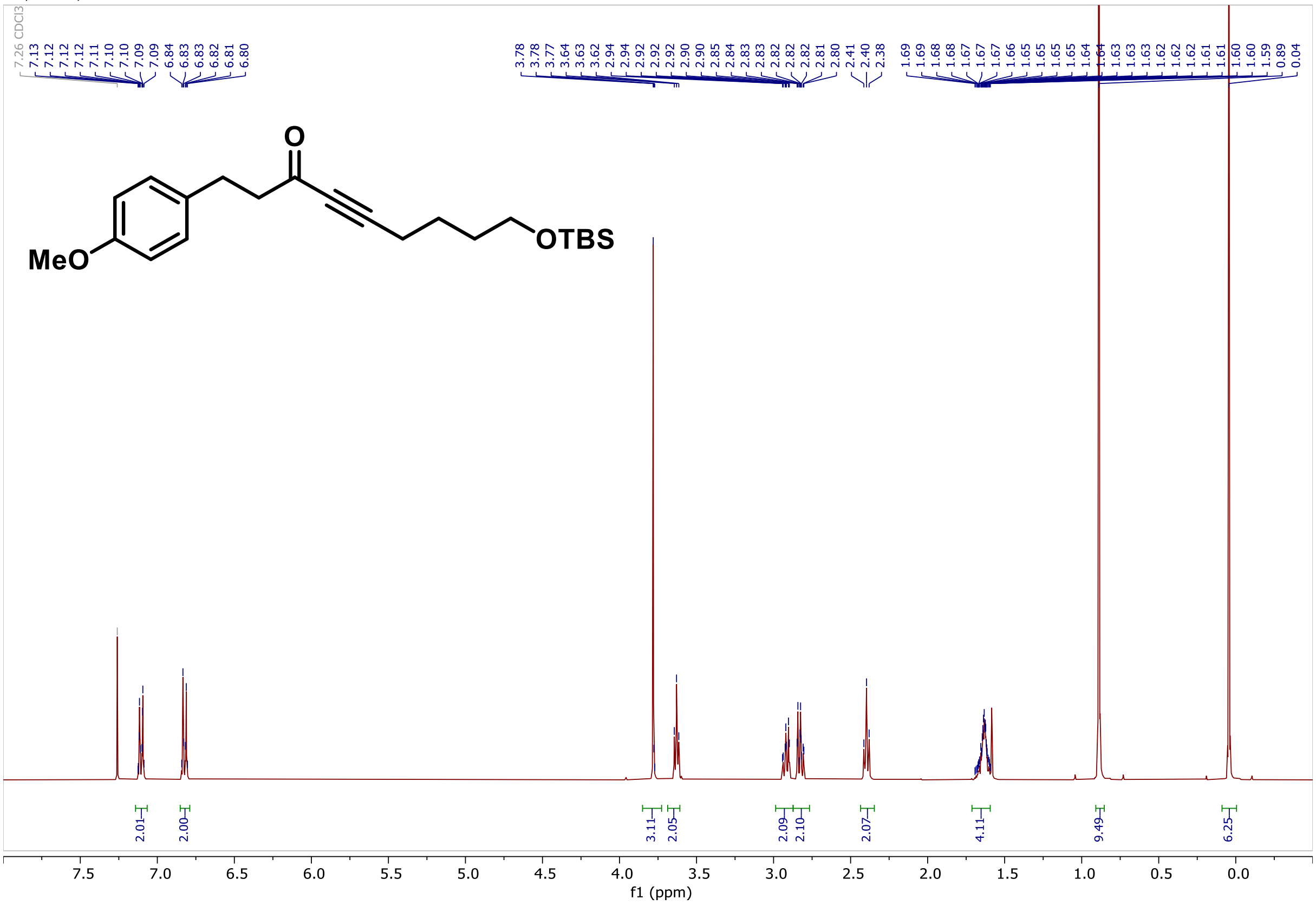

13C, CDCl3, 101 MHz

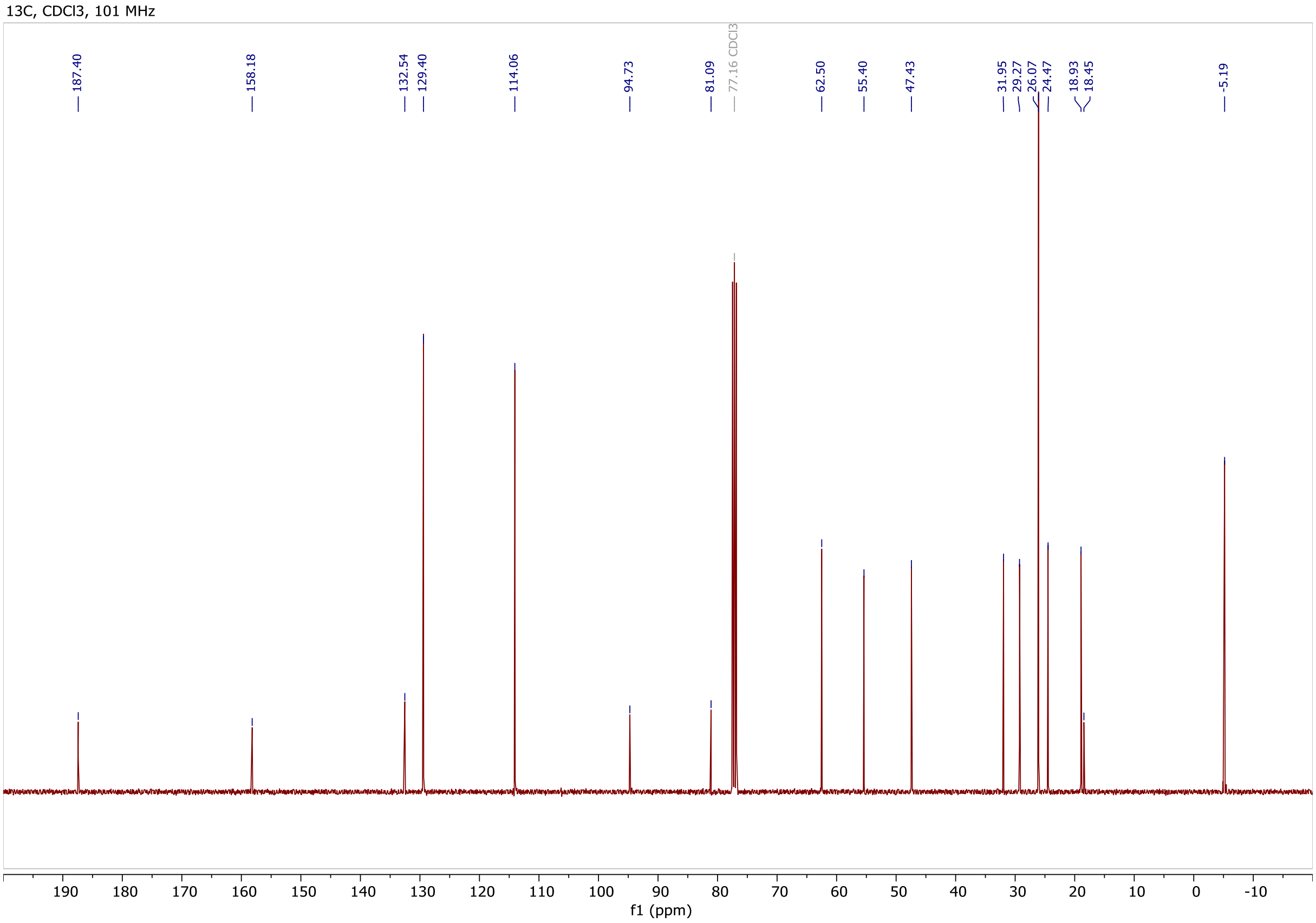

Compound S9

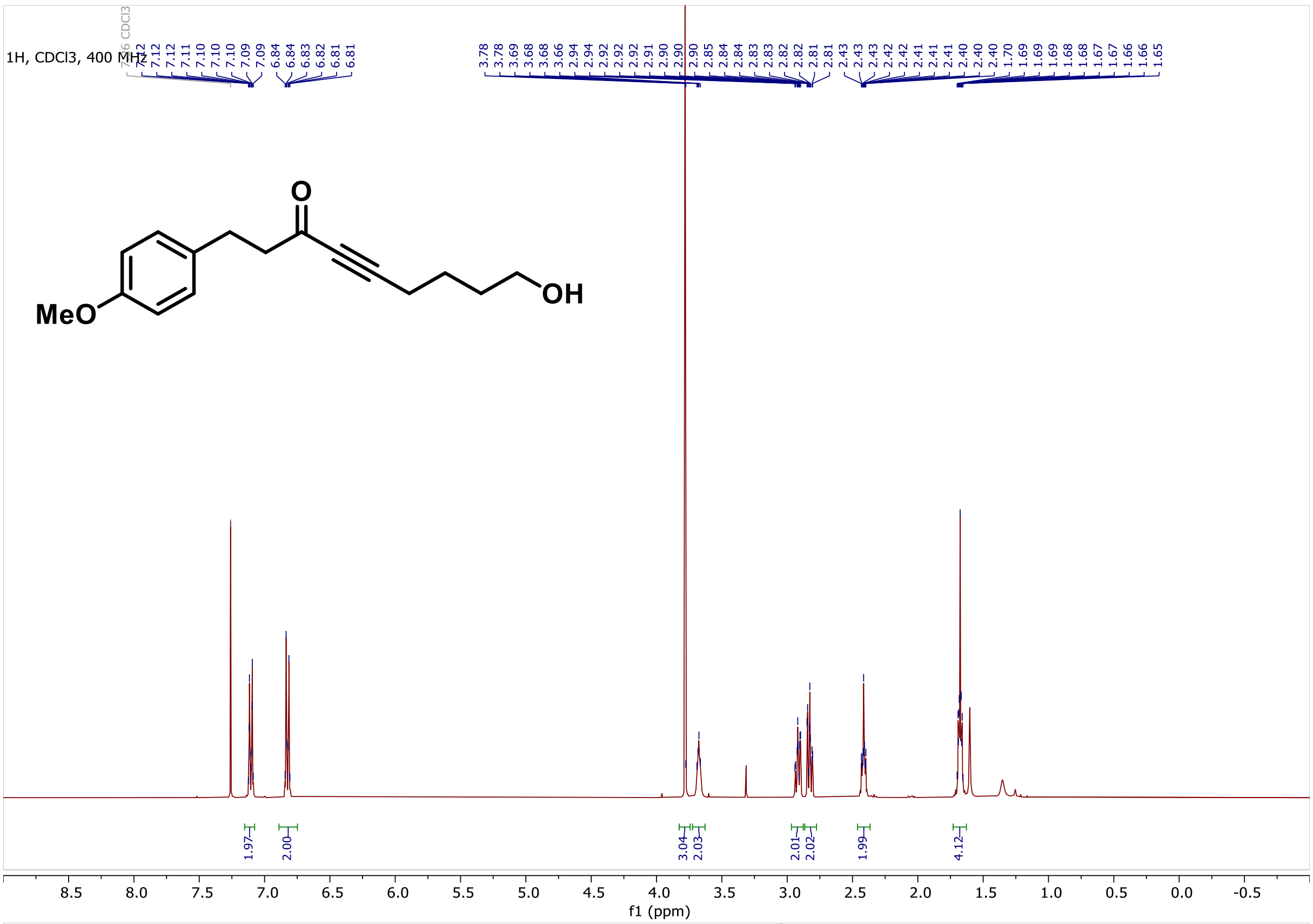

1H, CDCl3, 400 MHz
7.26 CDCl3
7.12 7.12 7.11 7.10 7.10 7.09 7.09 6.84 6.84 6.83 6.82 6.81 6.81
3.78 3.78 3.69 3.68 3.68 3.66 2.94 2.94 2.92 2.92 2.92 2.91 2.90 2.90 2.90 2.85 2.84 2.84 2.83 2.83 2.82 2.82 2.81 2.81 2.43 2.43 2.42 2.42 2.41 2.41 2.41 2.40 2.40 2.40 1.70 1.69 1.69 1.69 1.68 1.68 1.67 1.67 1.66 1.65
MeO
O
OH
1.97 2.00 3.04 2.03 2.01 2.02 1.99 4.12
f1 (ppm)

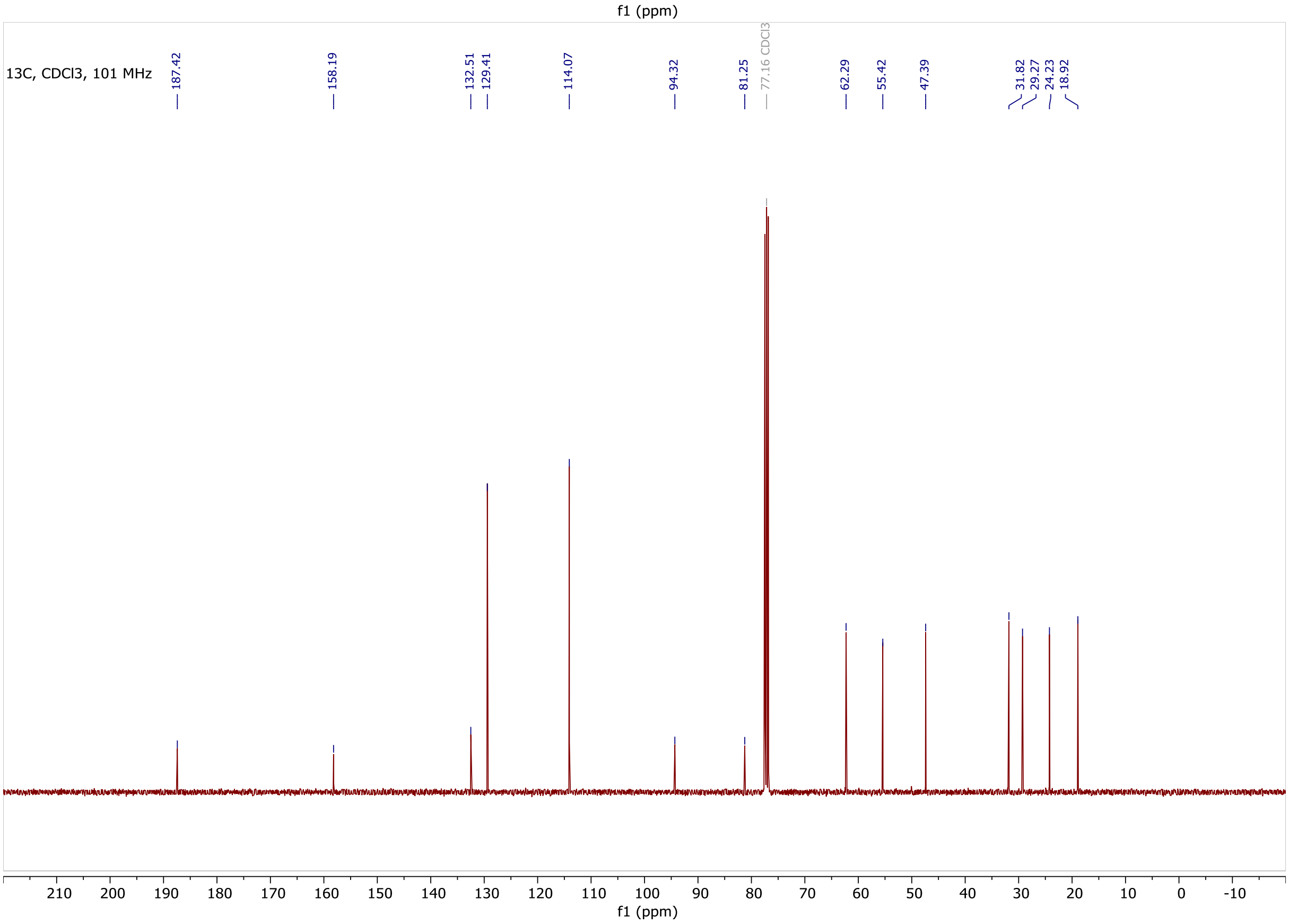

13C, CDCl3, 101 MHz
187.42
158.19
132.51
129.41
114.07
94.32
81.25
77.16 CDCl3
62.29
55.42
47.39
31.82
29.27
24.23
18.92
f1 (ppm)

Compound S10

1H, CDCl3, 400 MHz

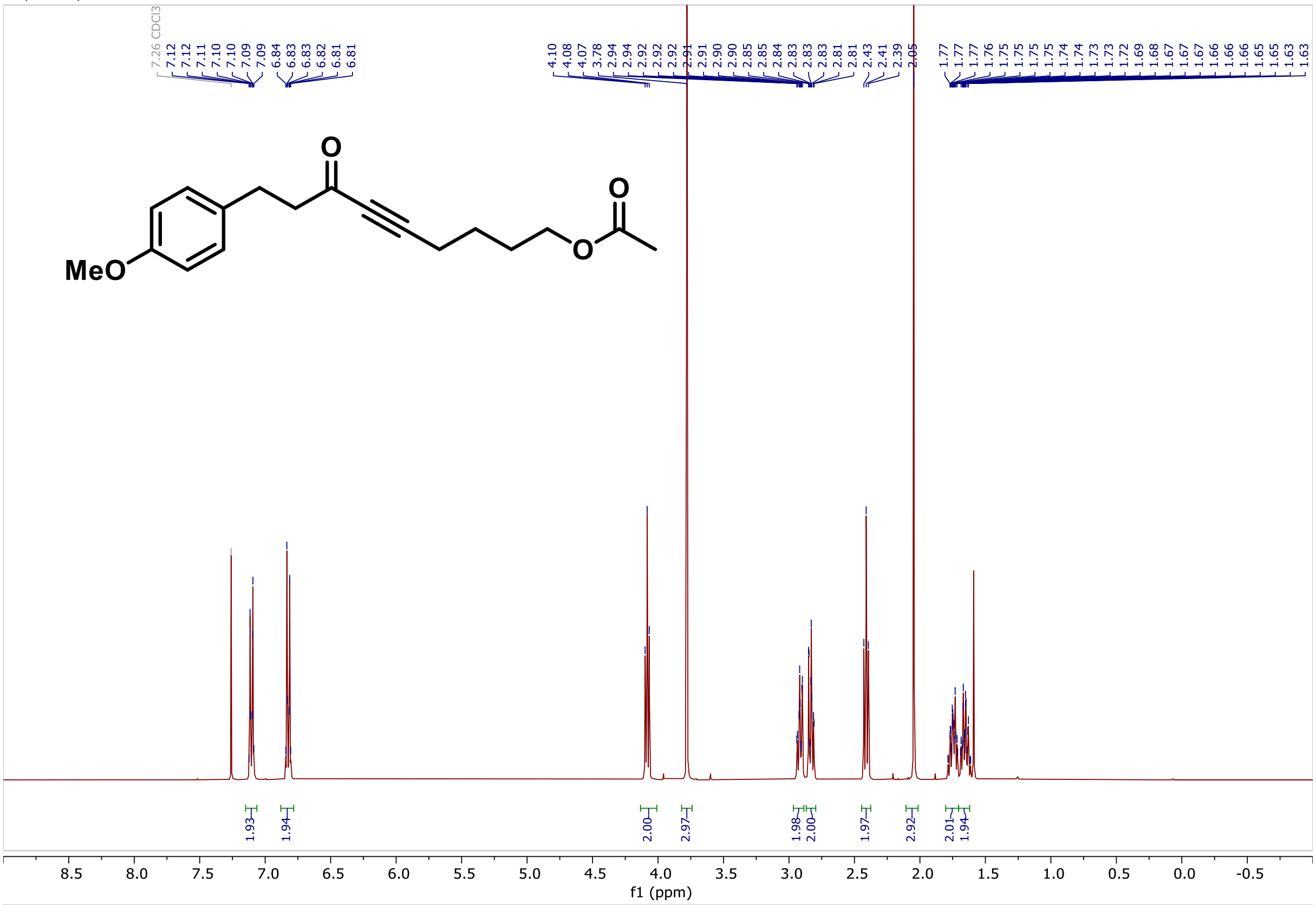

13C, CDCl3, 101 MHz

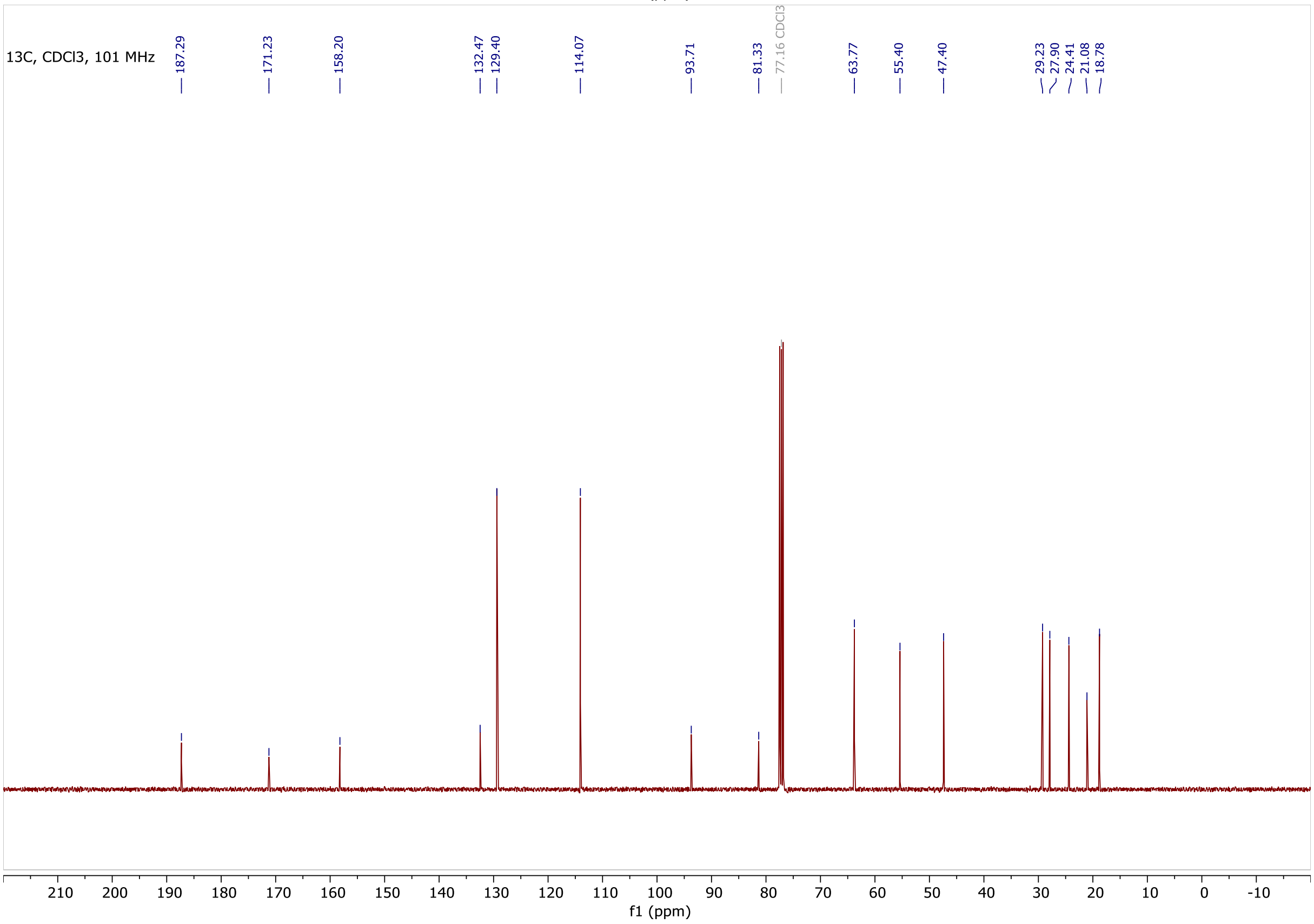

Compound S11

1H, CDCl3, 400 MHz

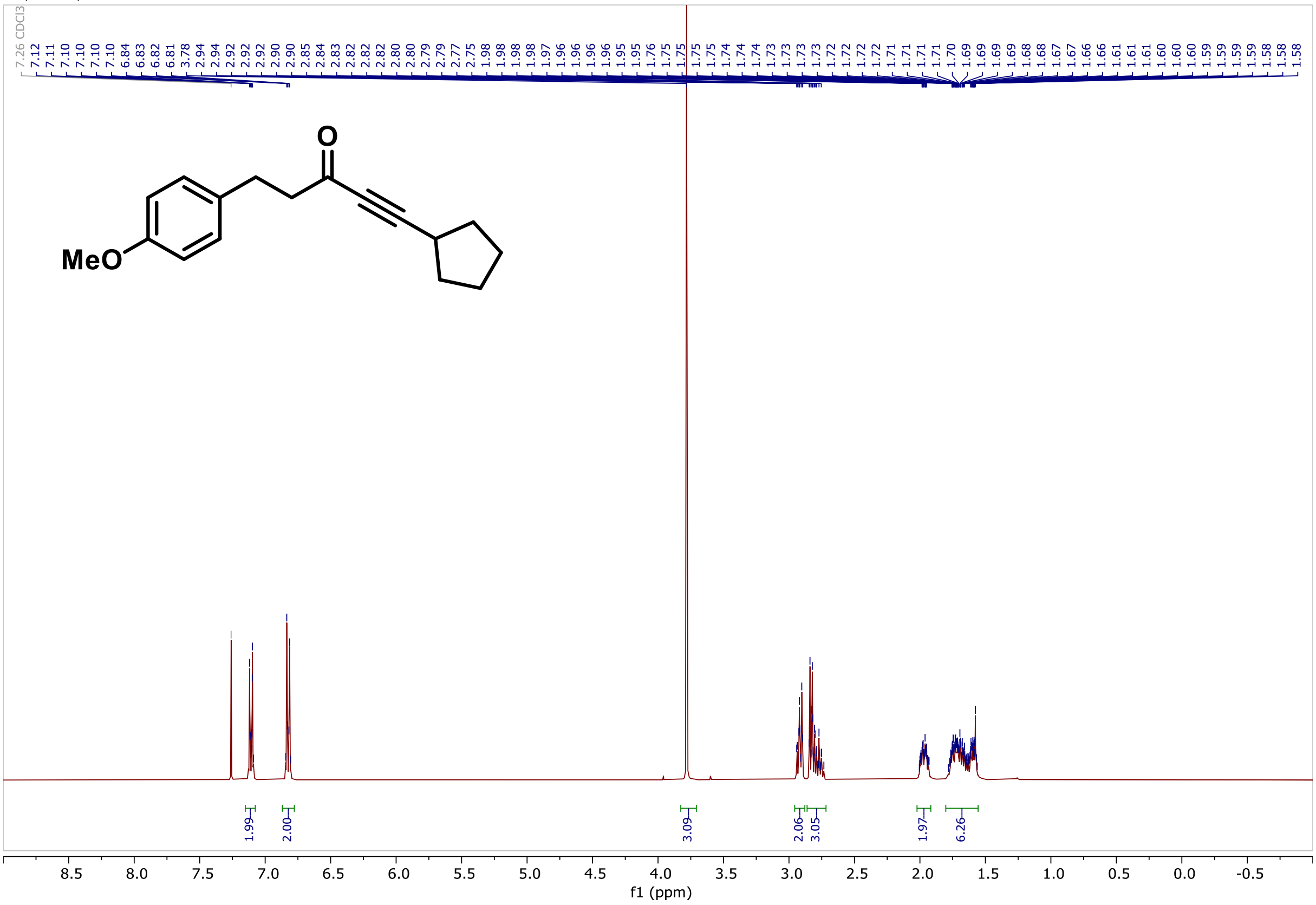

13C, CDCl3, 101 MHz

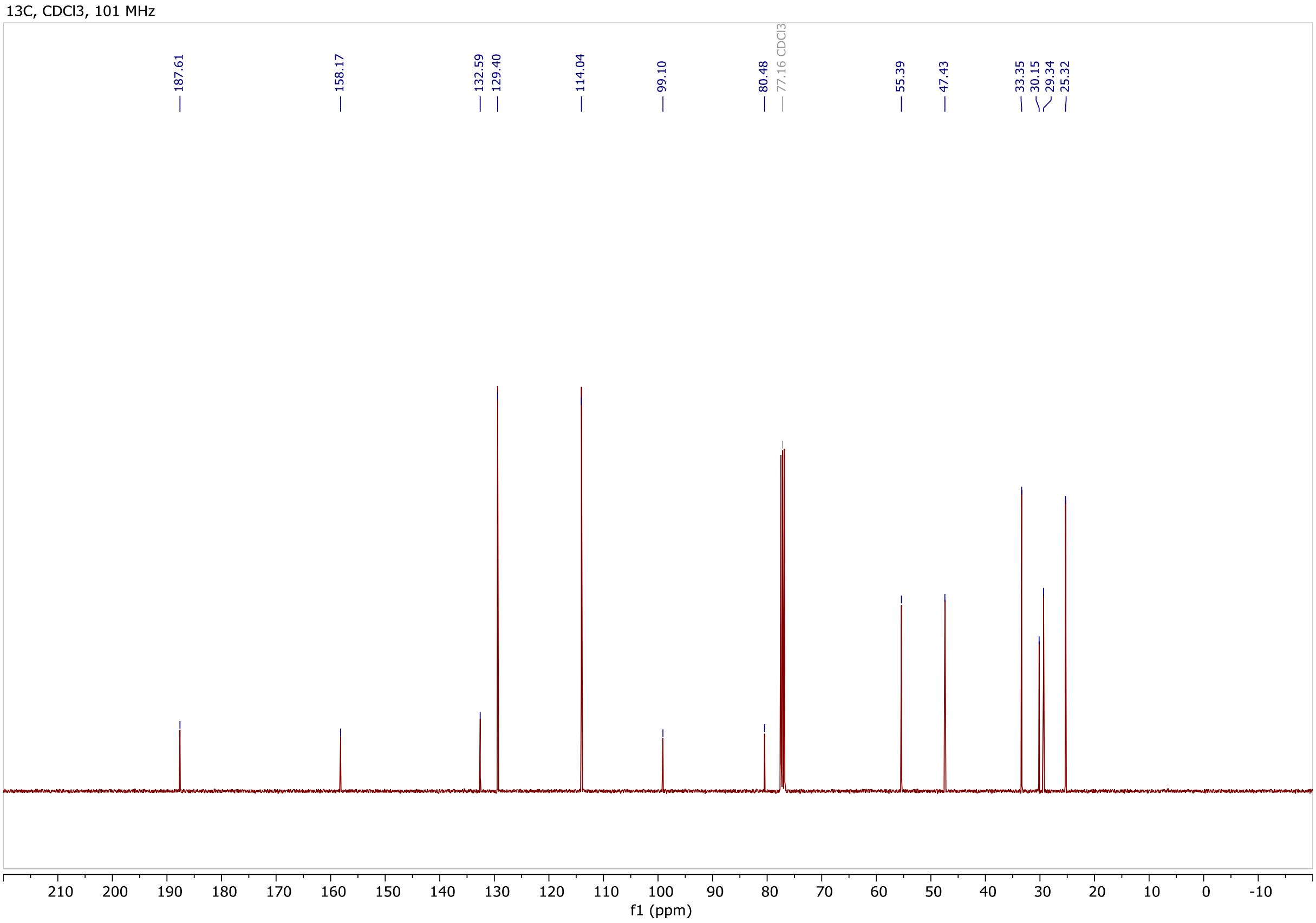

Compound S12

1H, CDCl3, 400 MHz

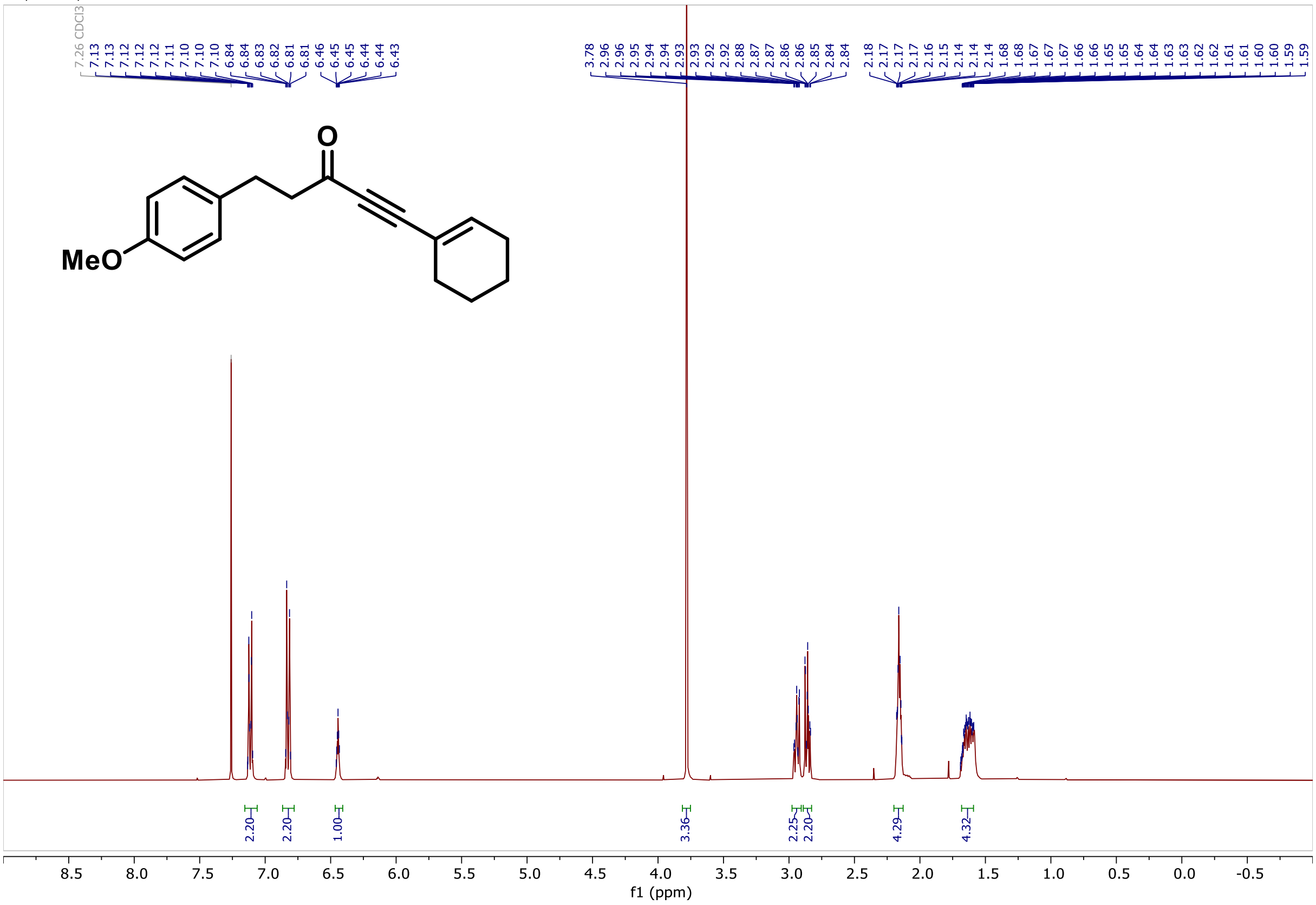

13C, CDCl3, 101 MHz

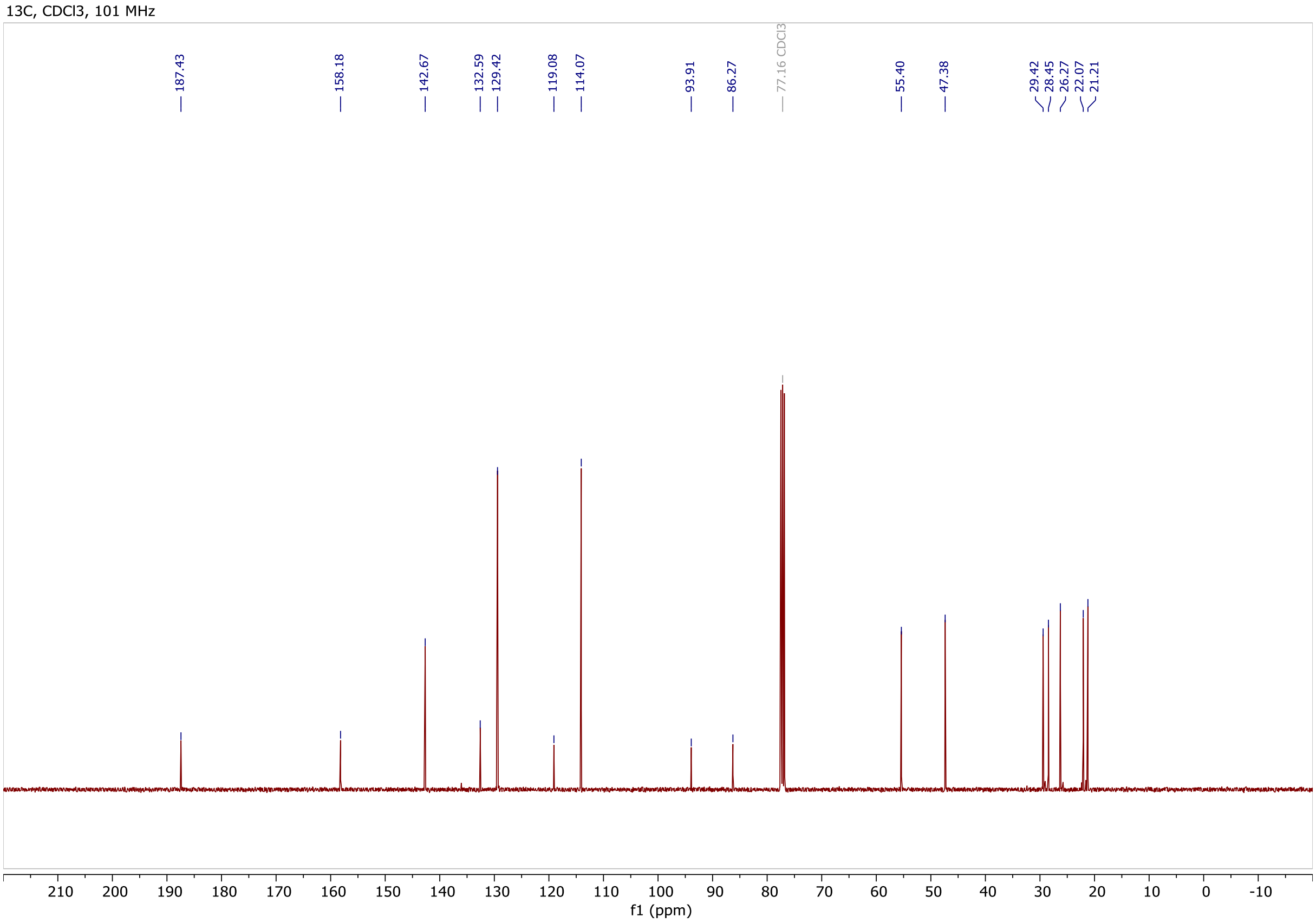

Compound S13

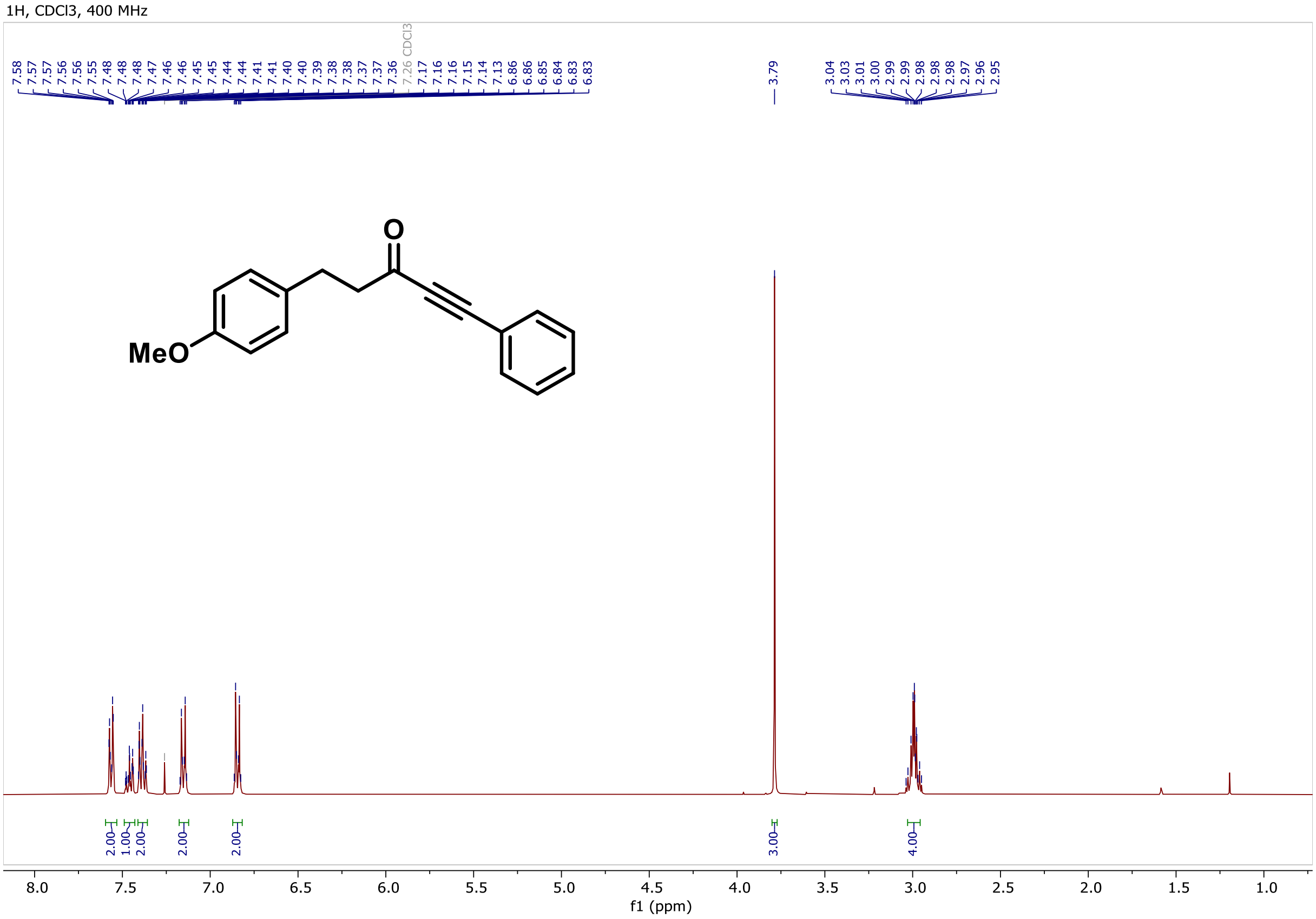

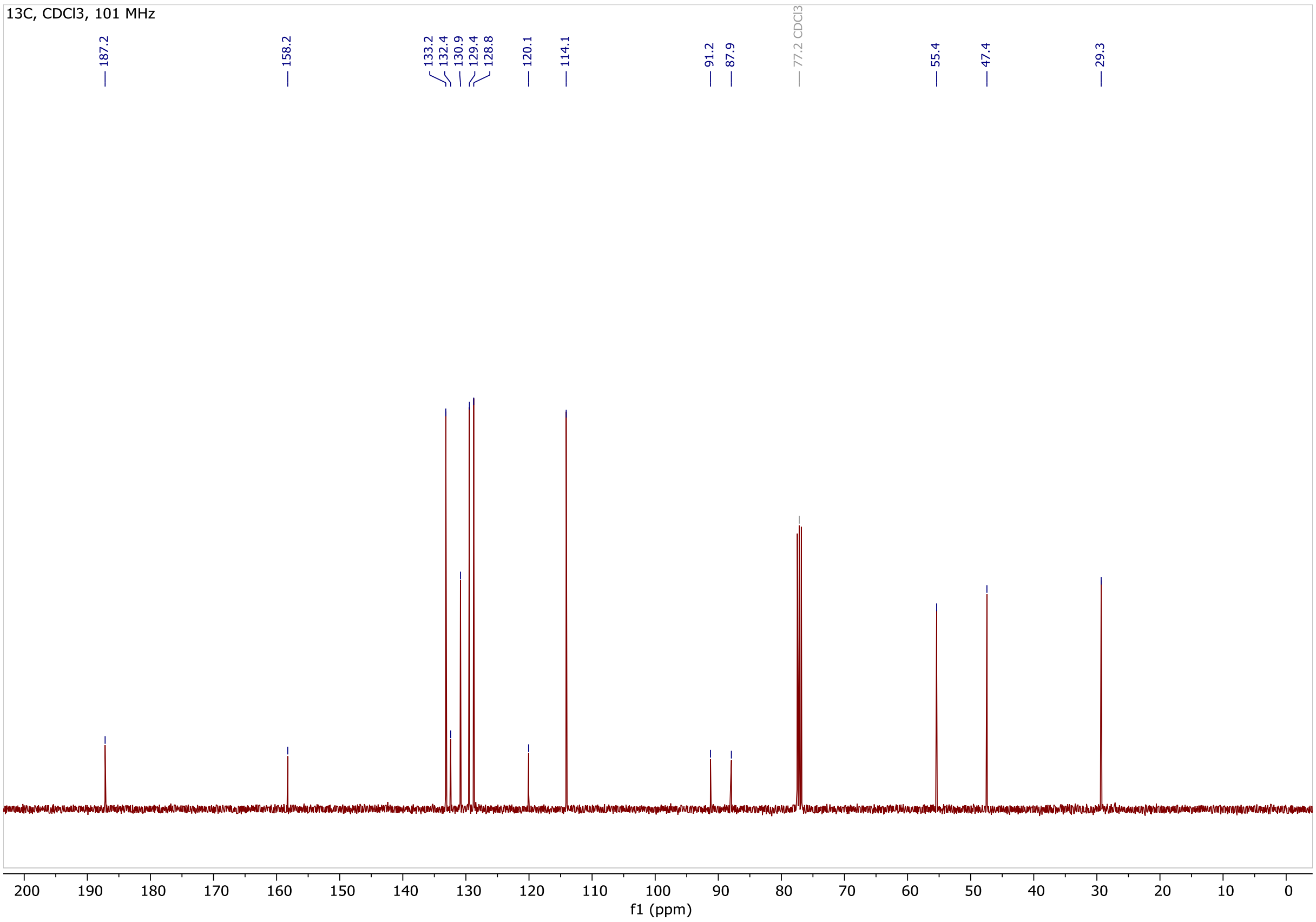

Compound 4

1H, CDCl3, 400 MHz

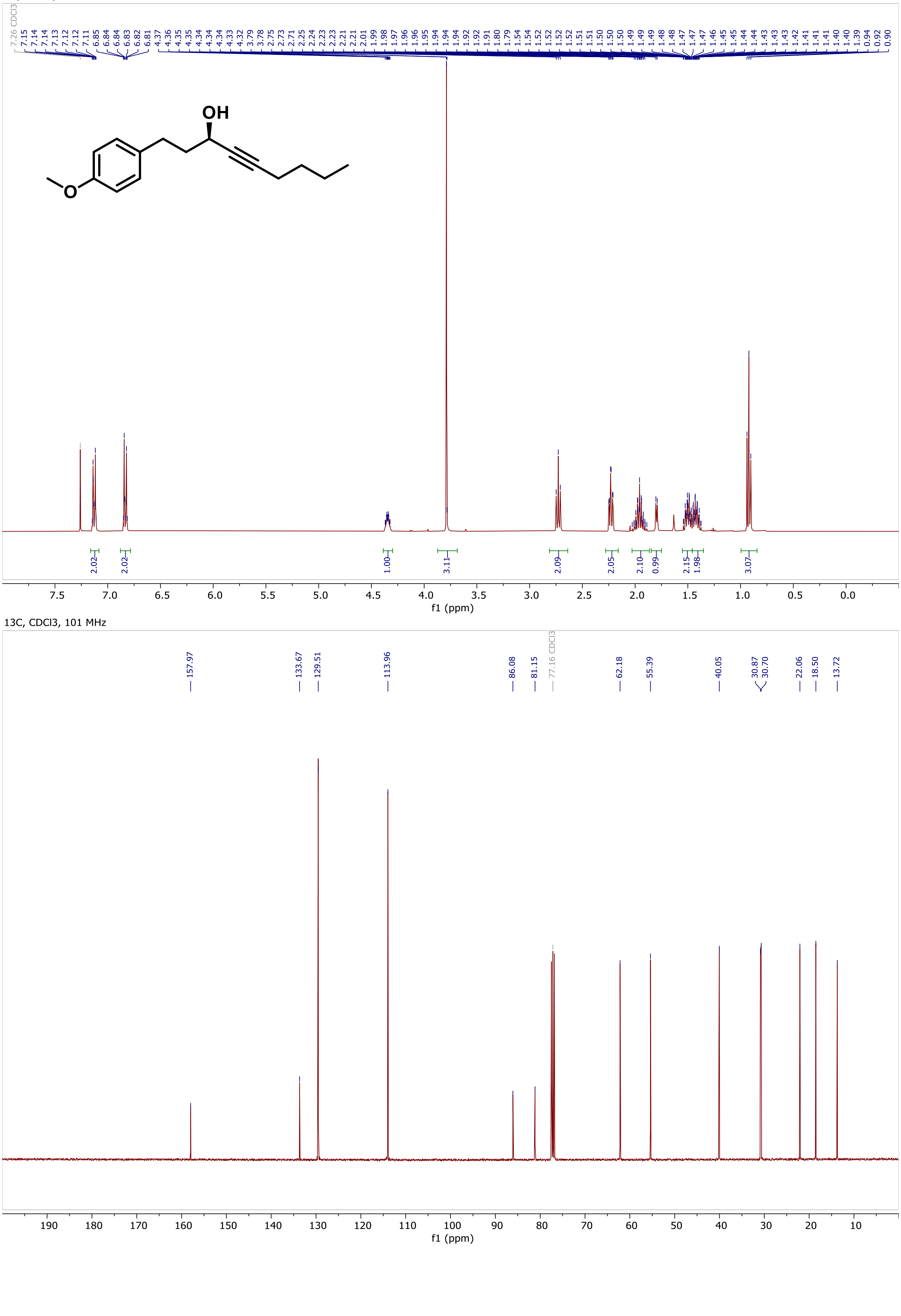

13C, CDCl3, 101 MHz

Compound S14

1H, CDCl3, 400 MHz

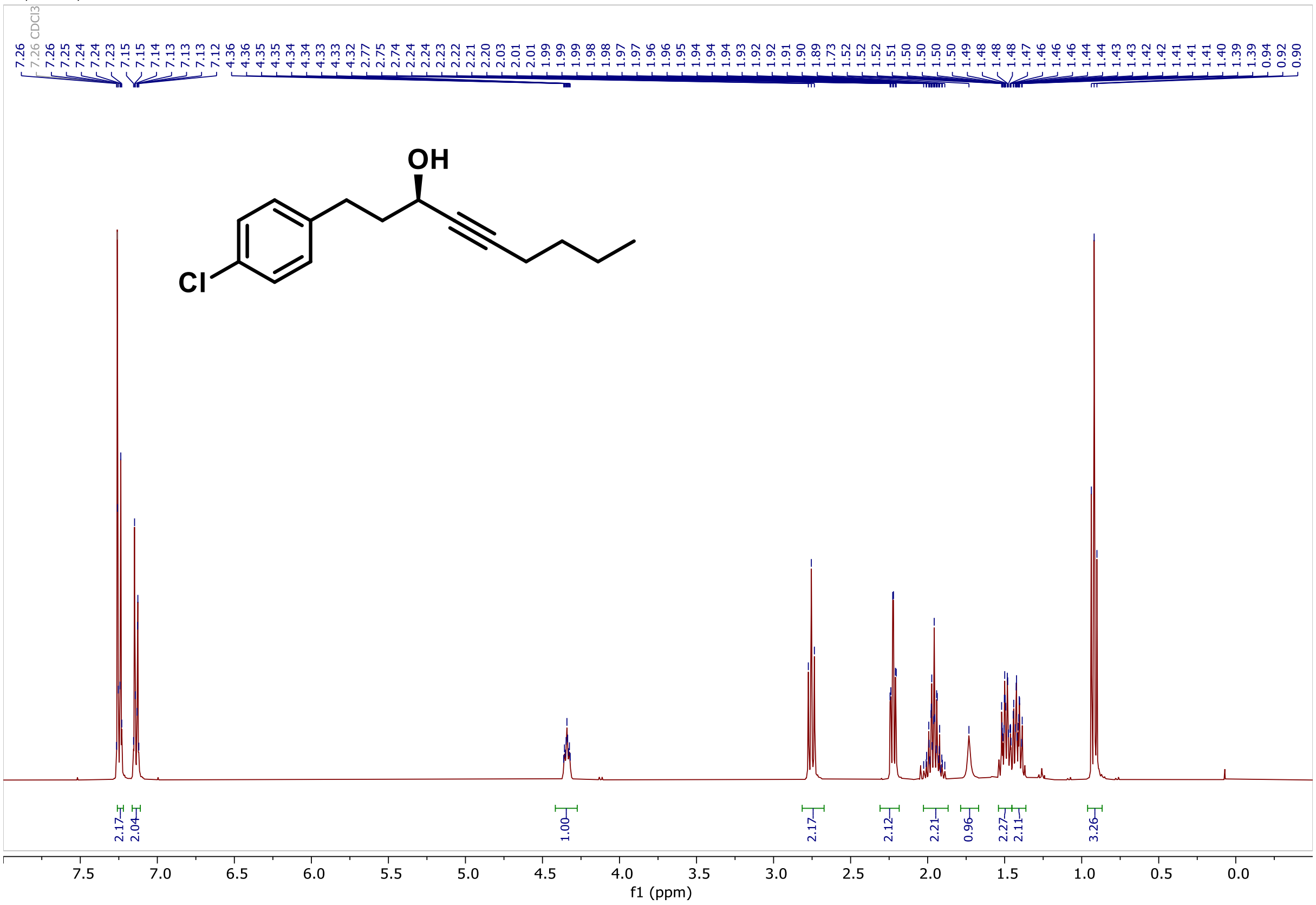

13C, CDCl3, 101 MHz

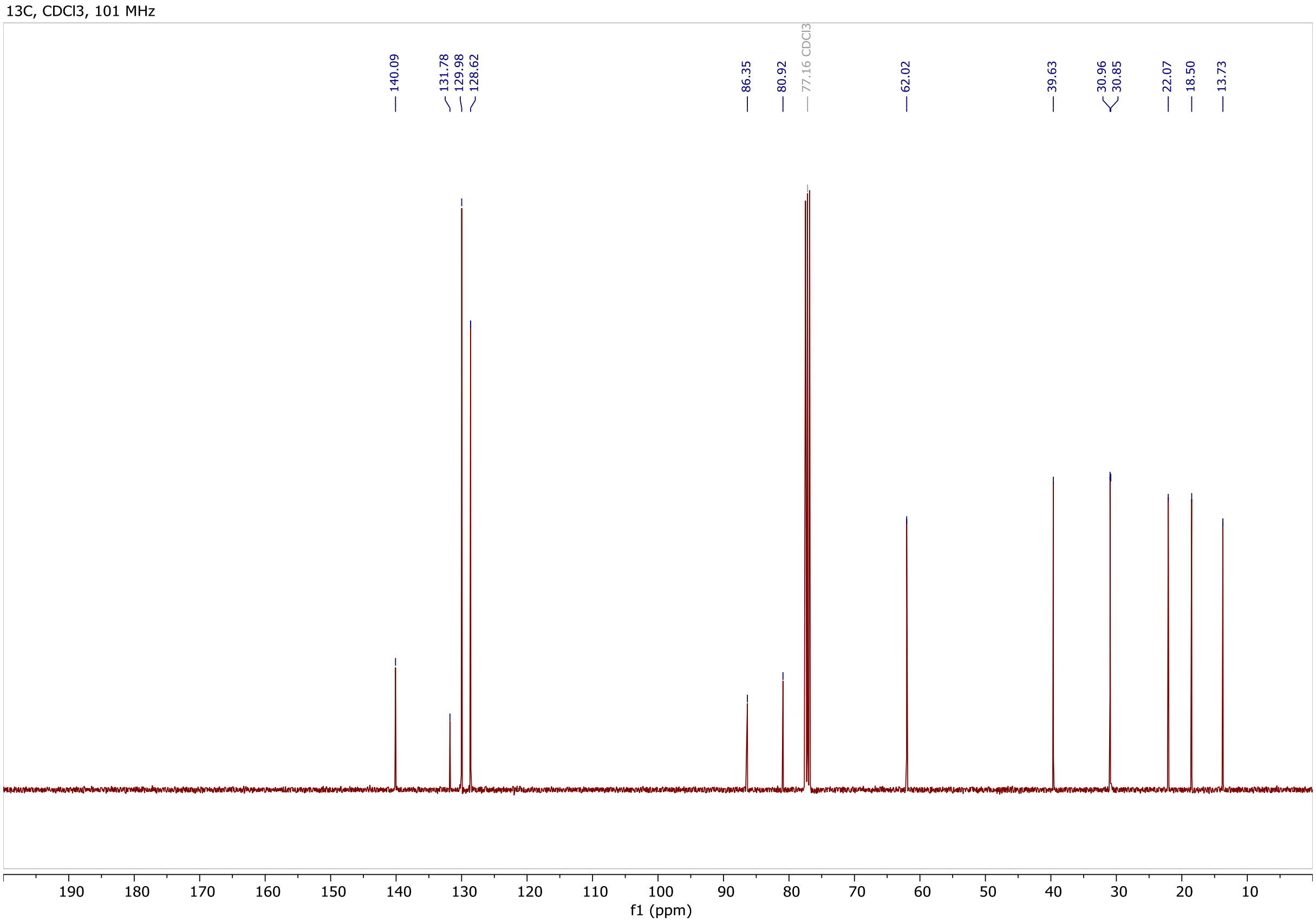

Compound S15

1H, CDCl3, 400 MHz

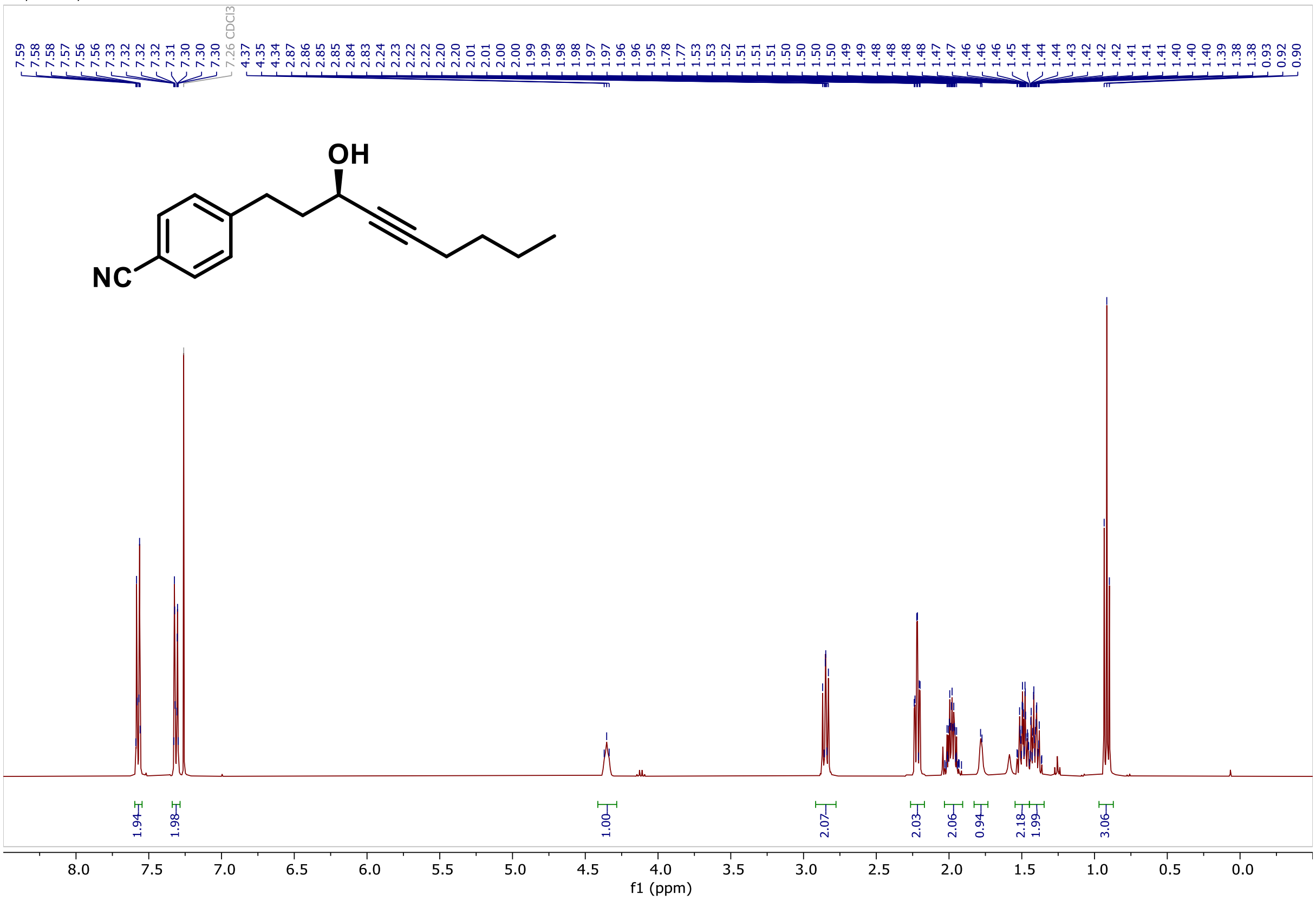

13C, CDCl3, 101 MHz

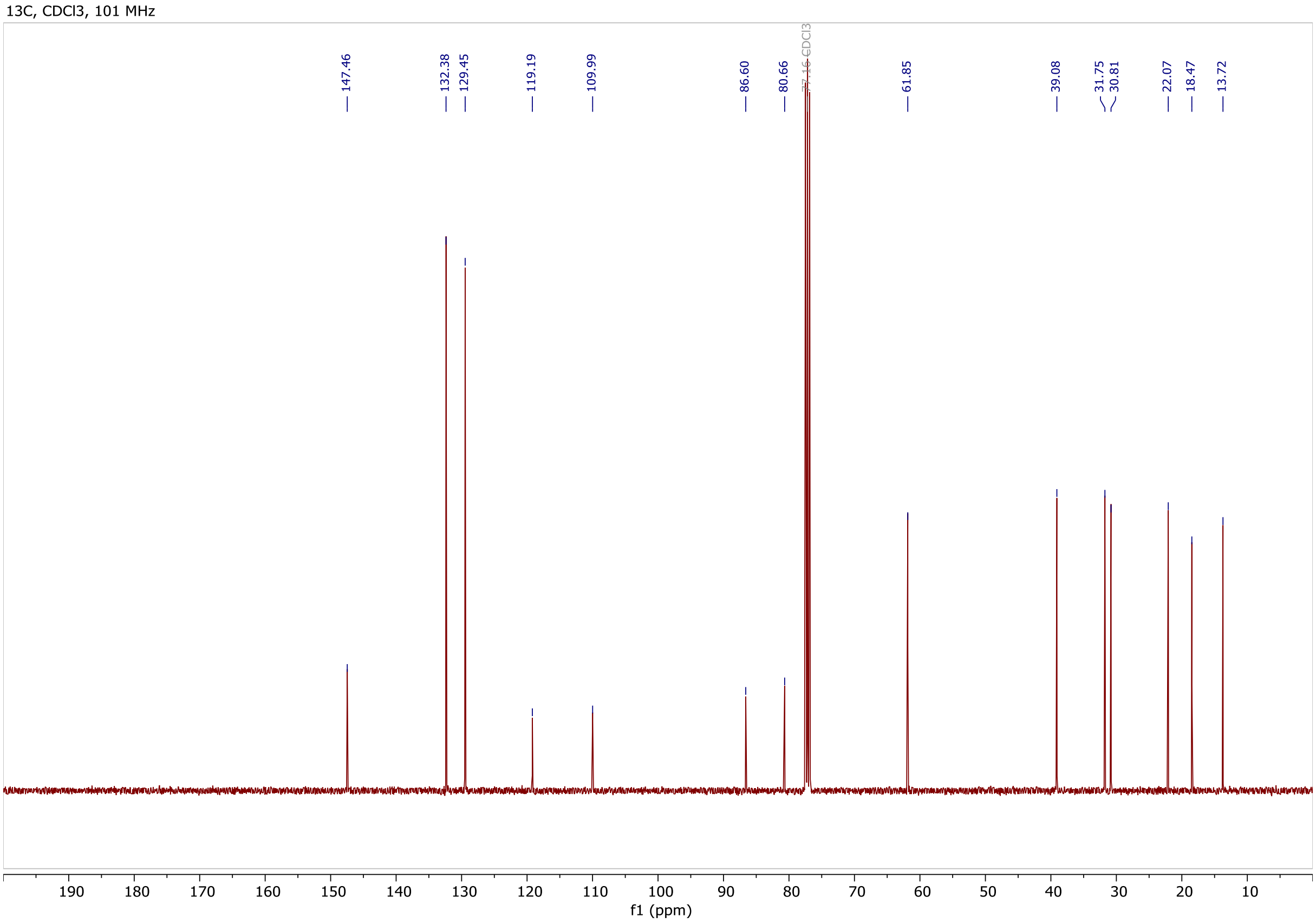

Compound S16

1H, CDCl3, 400 MHz

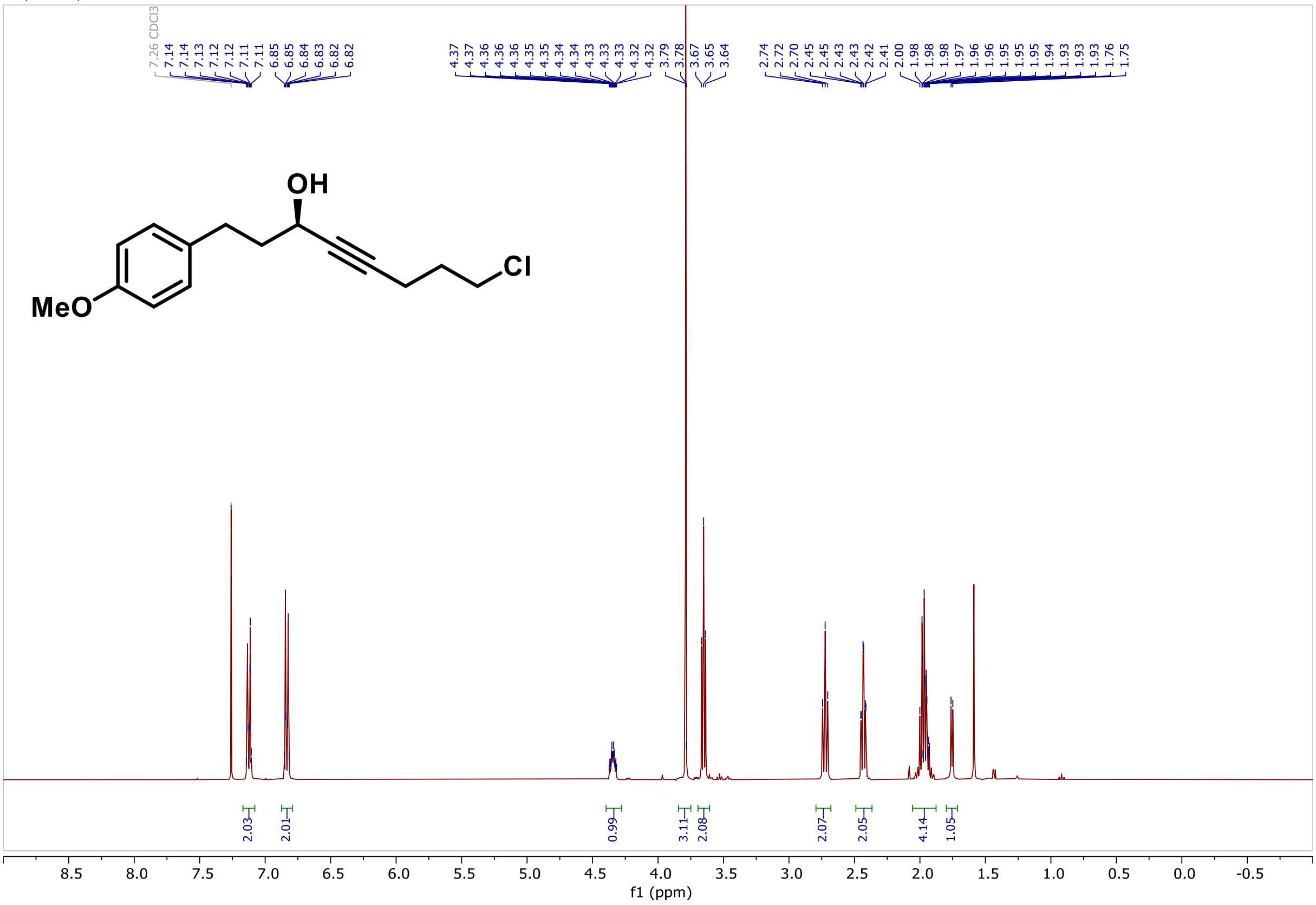

13C, CDCl3, 101 MHz

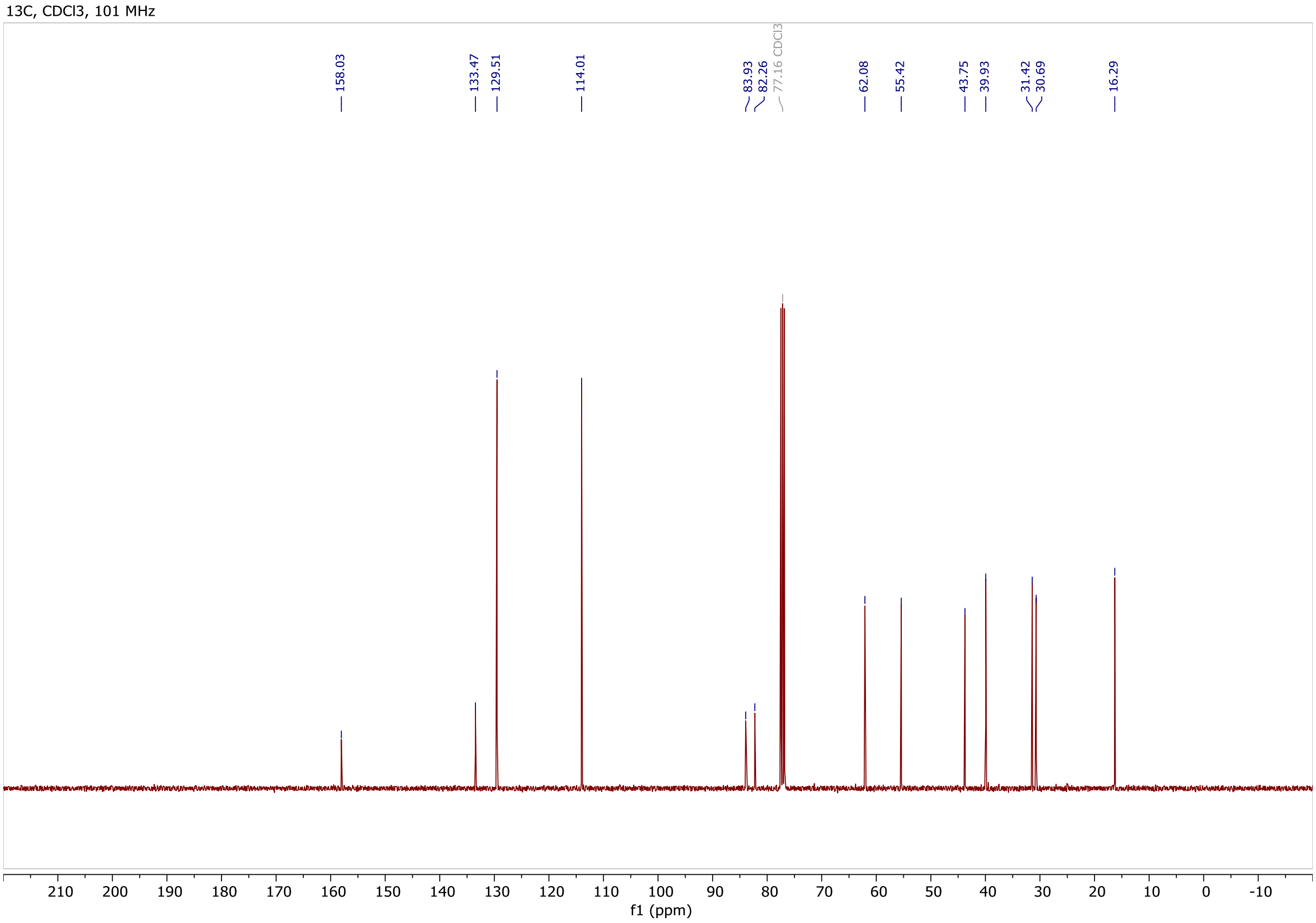

Compound S17

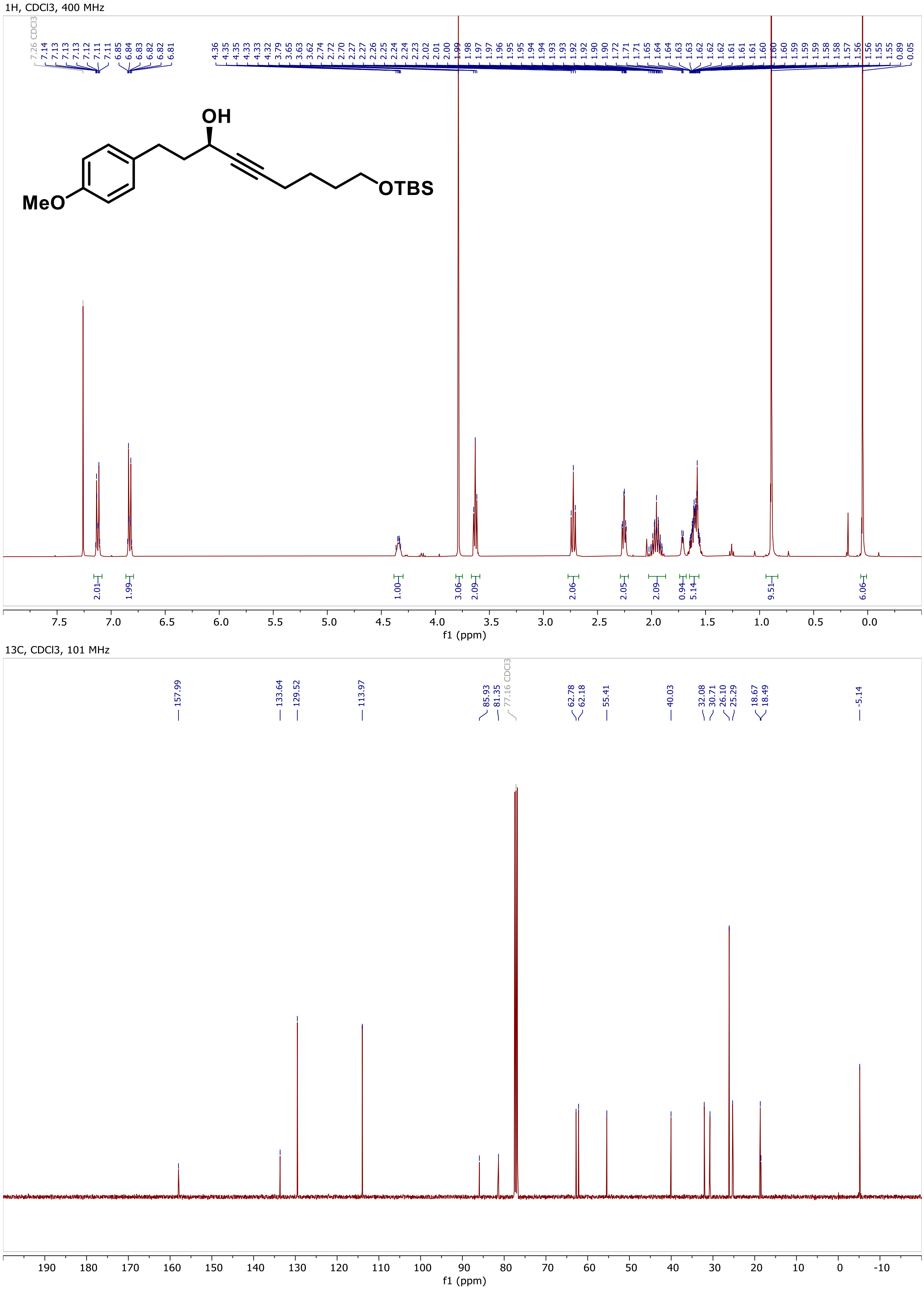

Compound S18

1H, CDCl3, 400 MHz

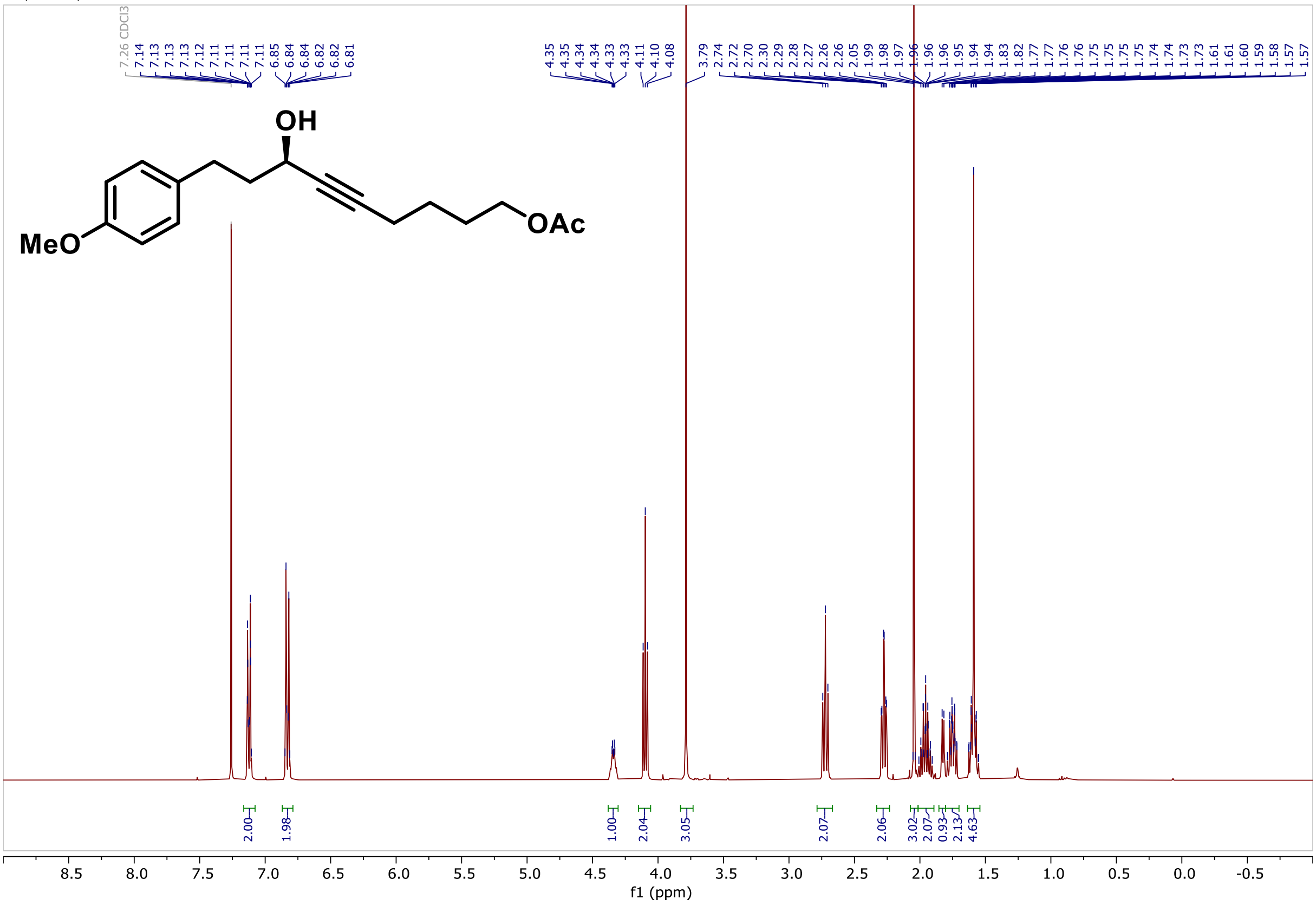

13C, CDCl3, 101 MHz

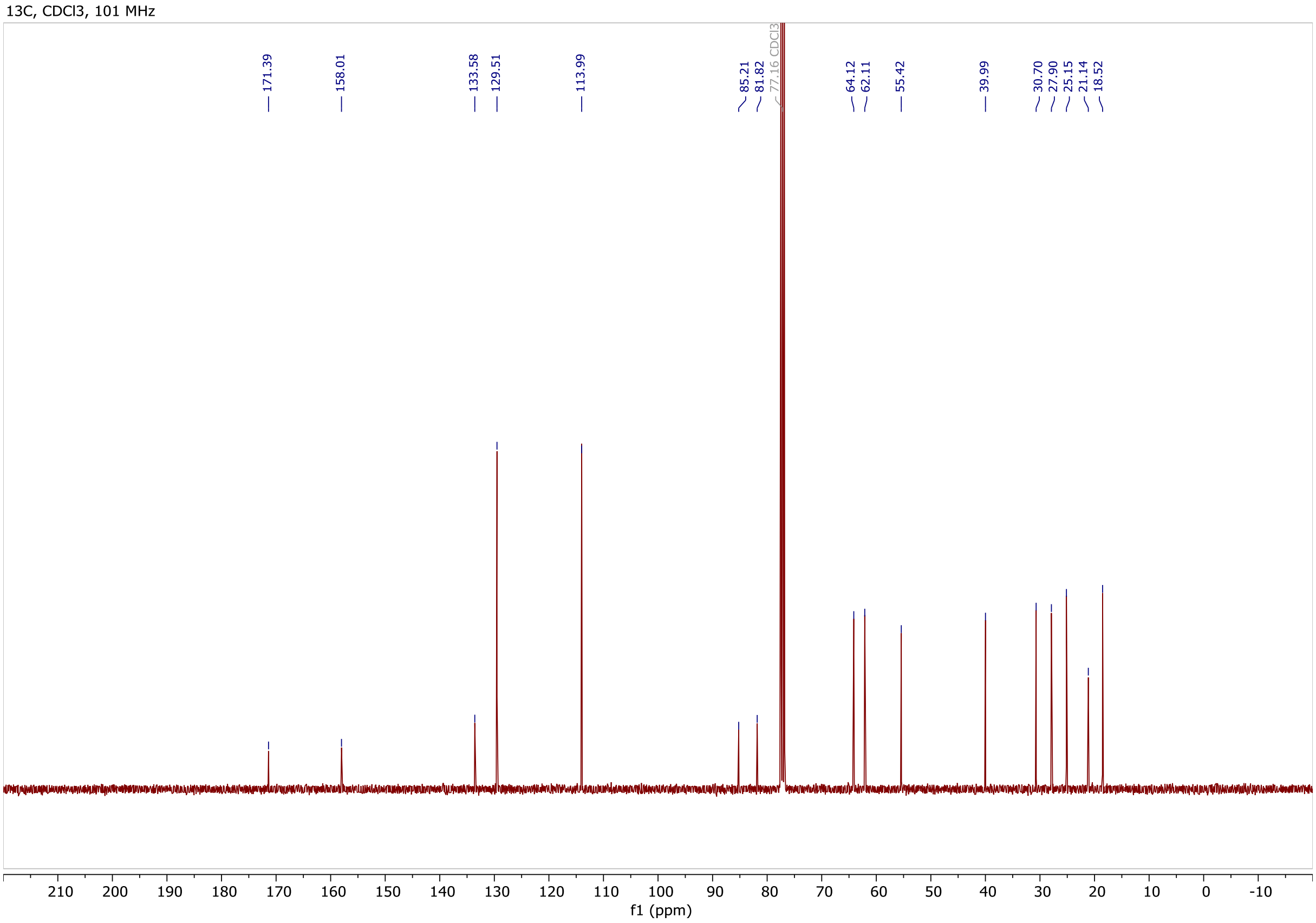

Compound S19

1H, CDCl3, 400 MHz

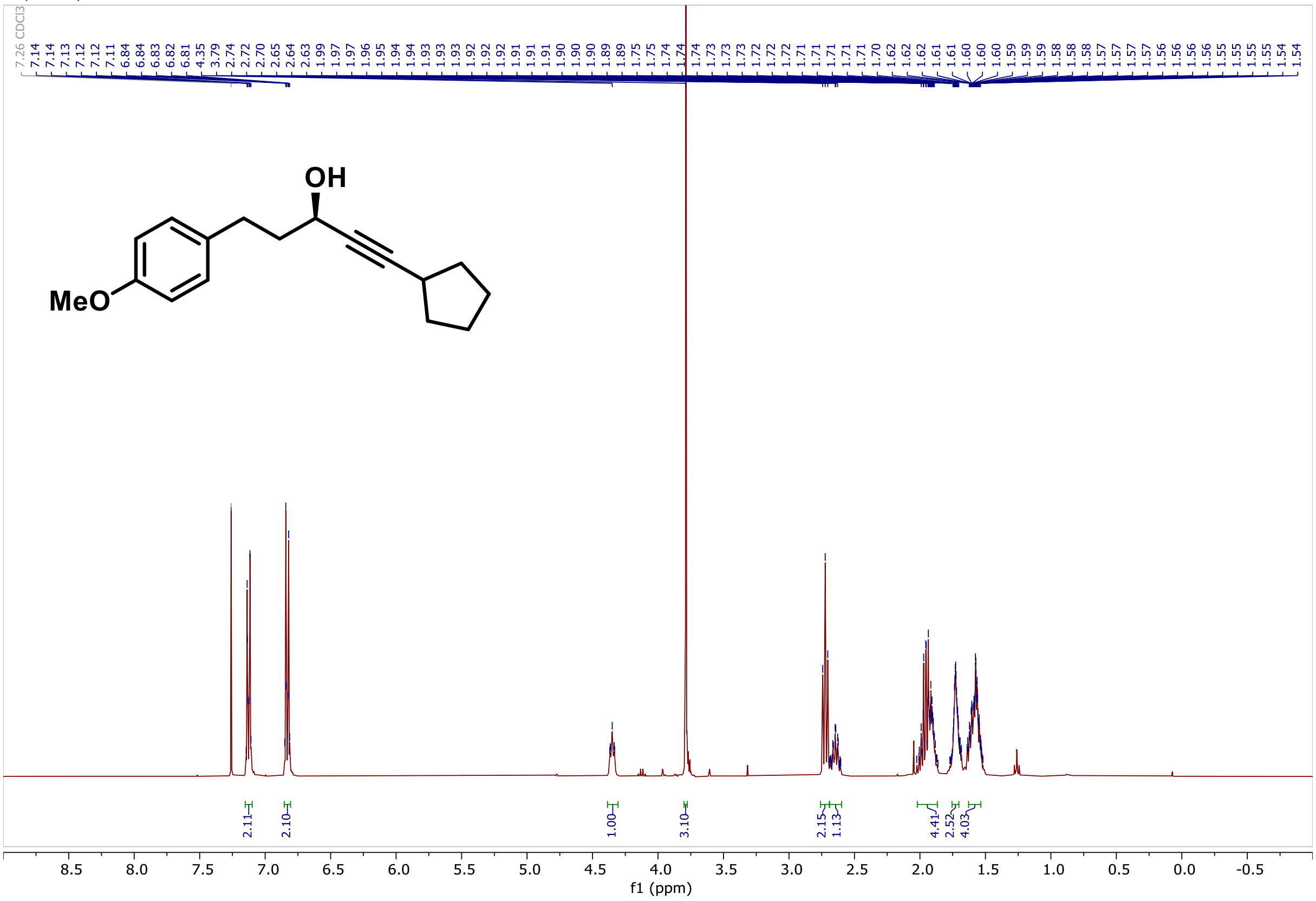

13C, CDCl3, 101 MHz

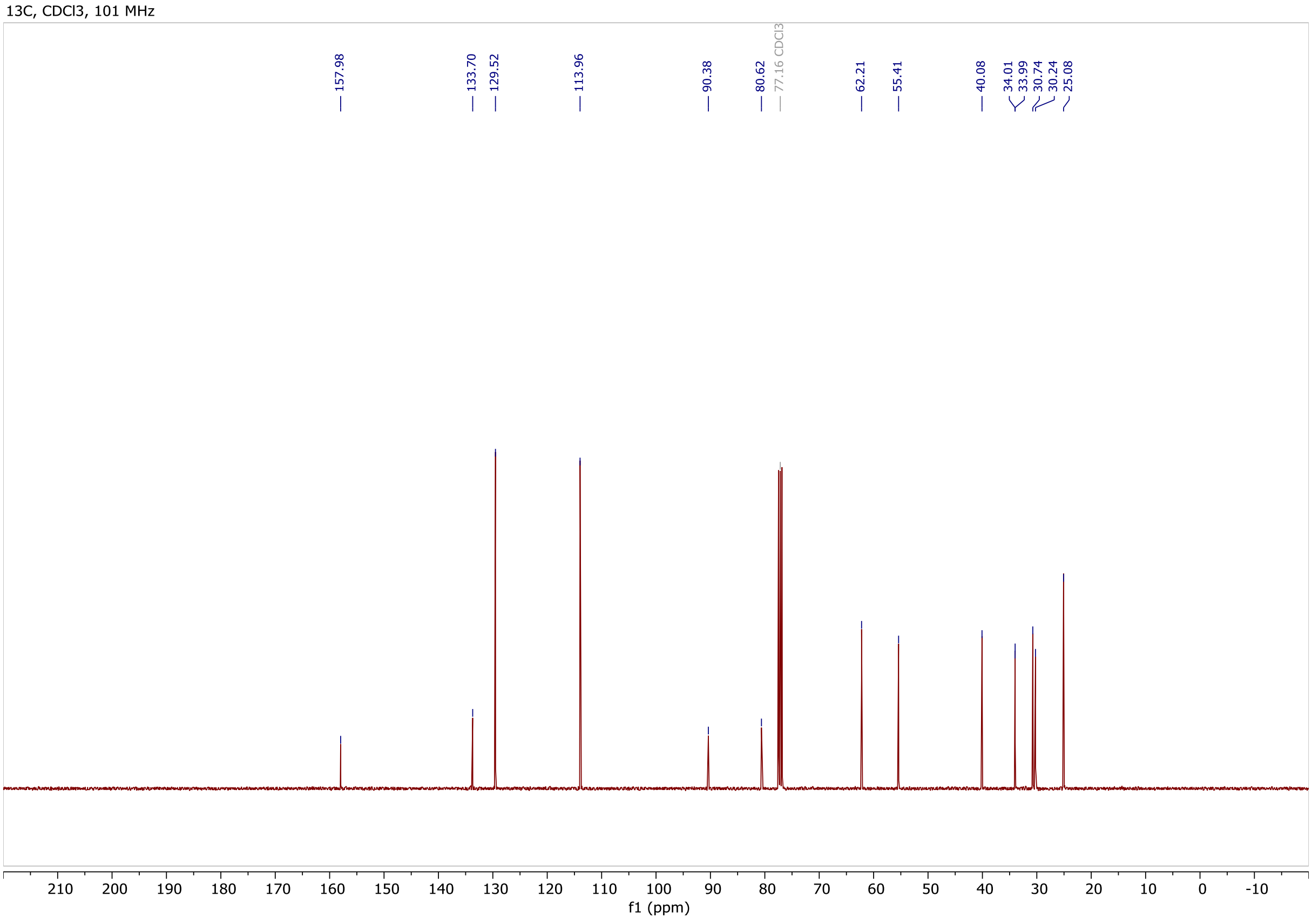

Compound S20

1H, CDCl3, 400 MHz

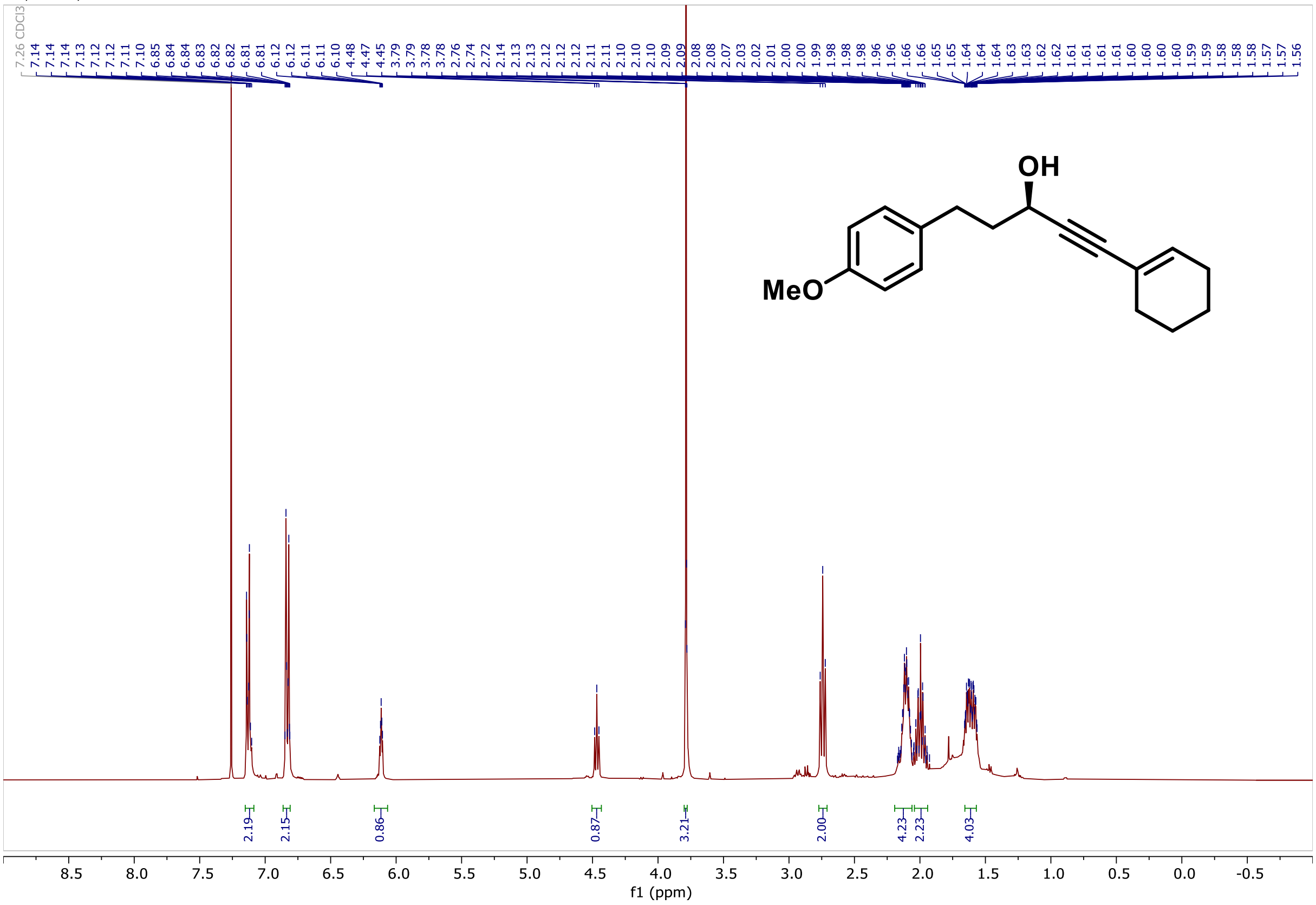

13C, CDCl3, 101 MHz

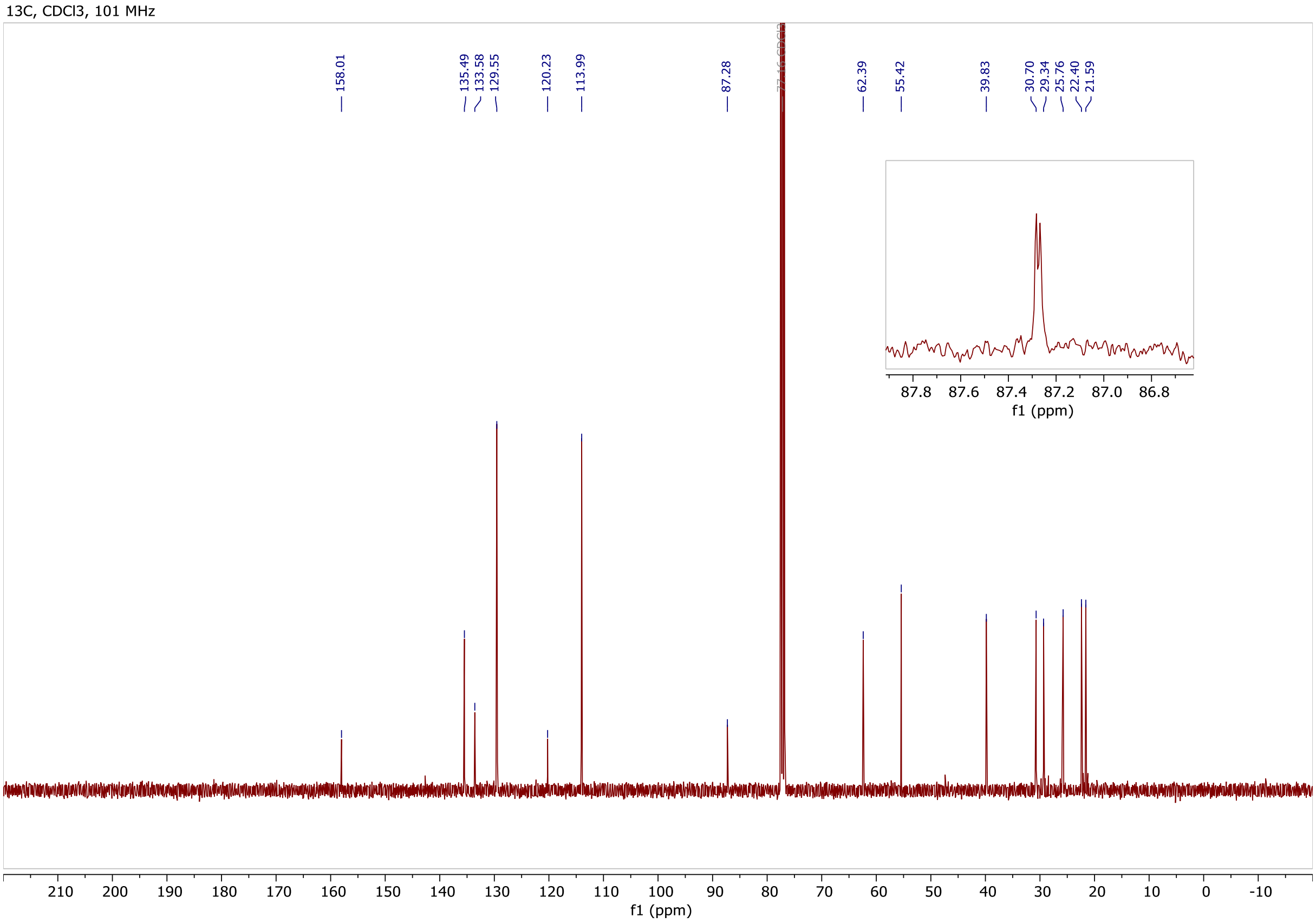

Compound S21

1H, CDCl3, 400 MHz

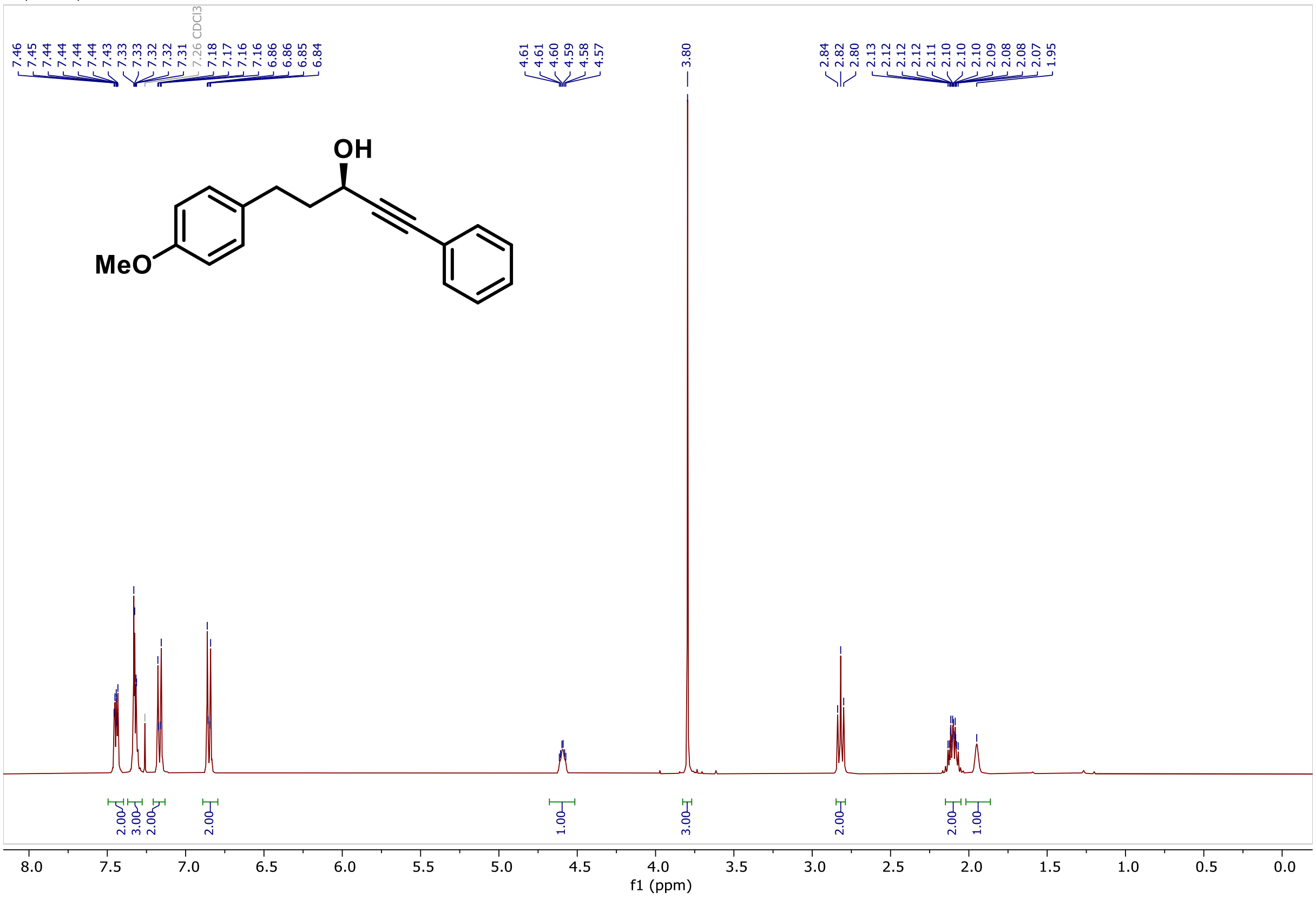

13C, CDCl3, 101 MHz

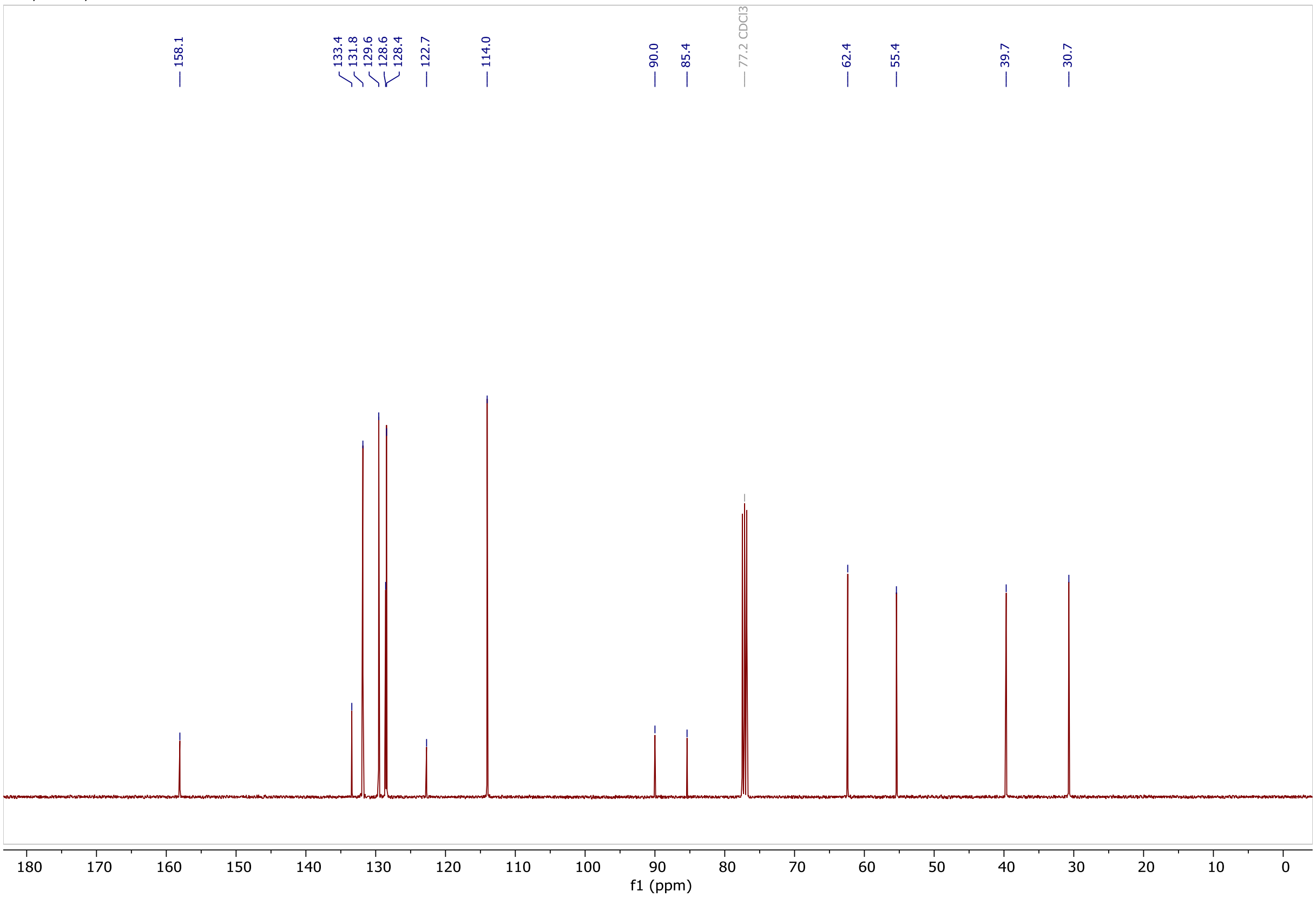

Compound S23

1H, CDCl3, 400 MHz

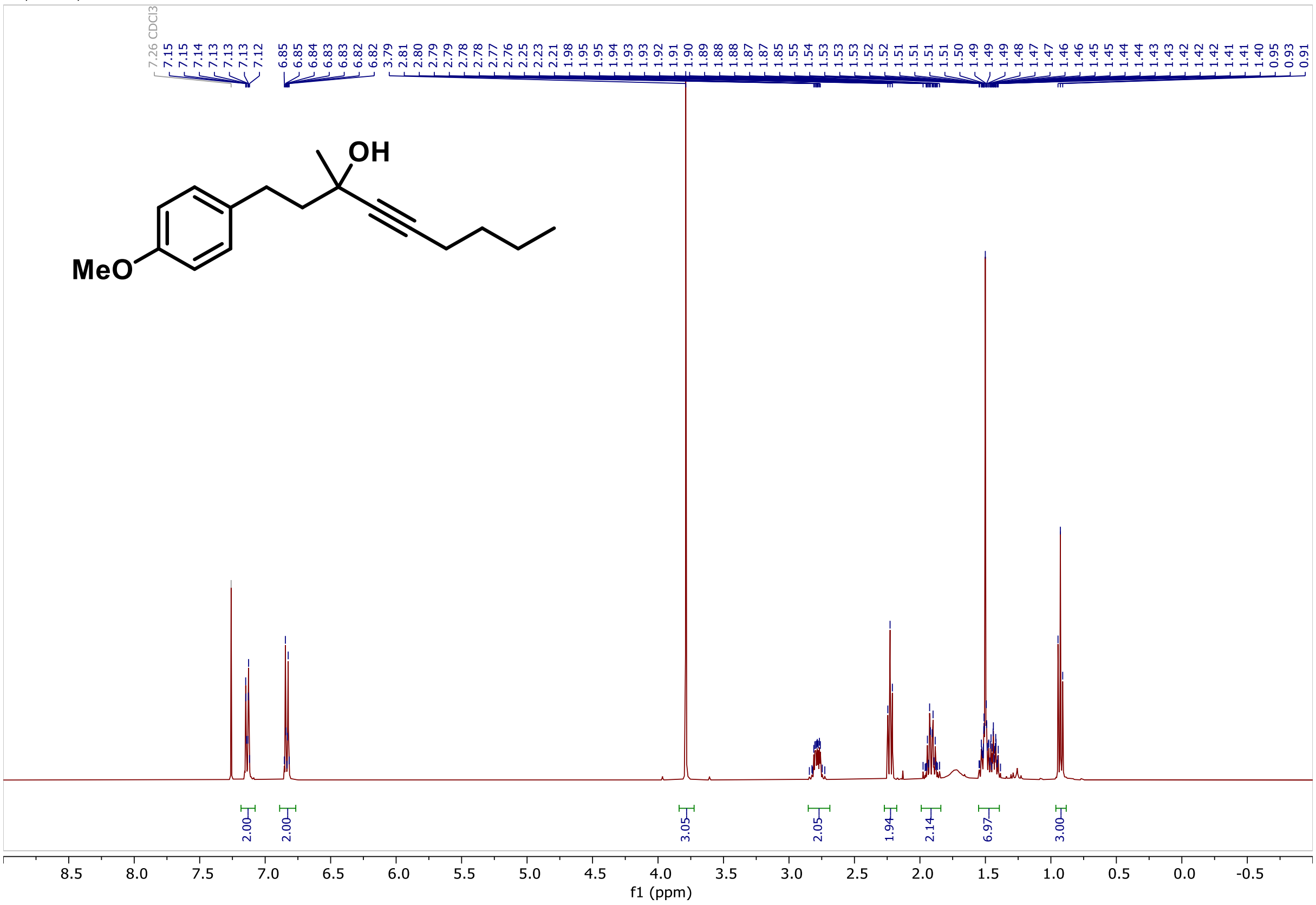

13C, CDCl3, 101 MHz

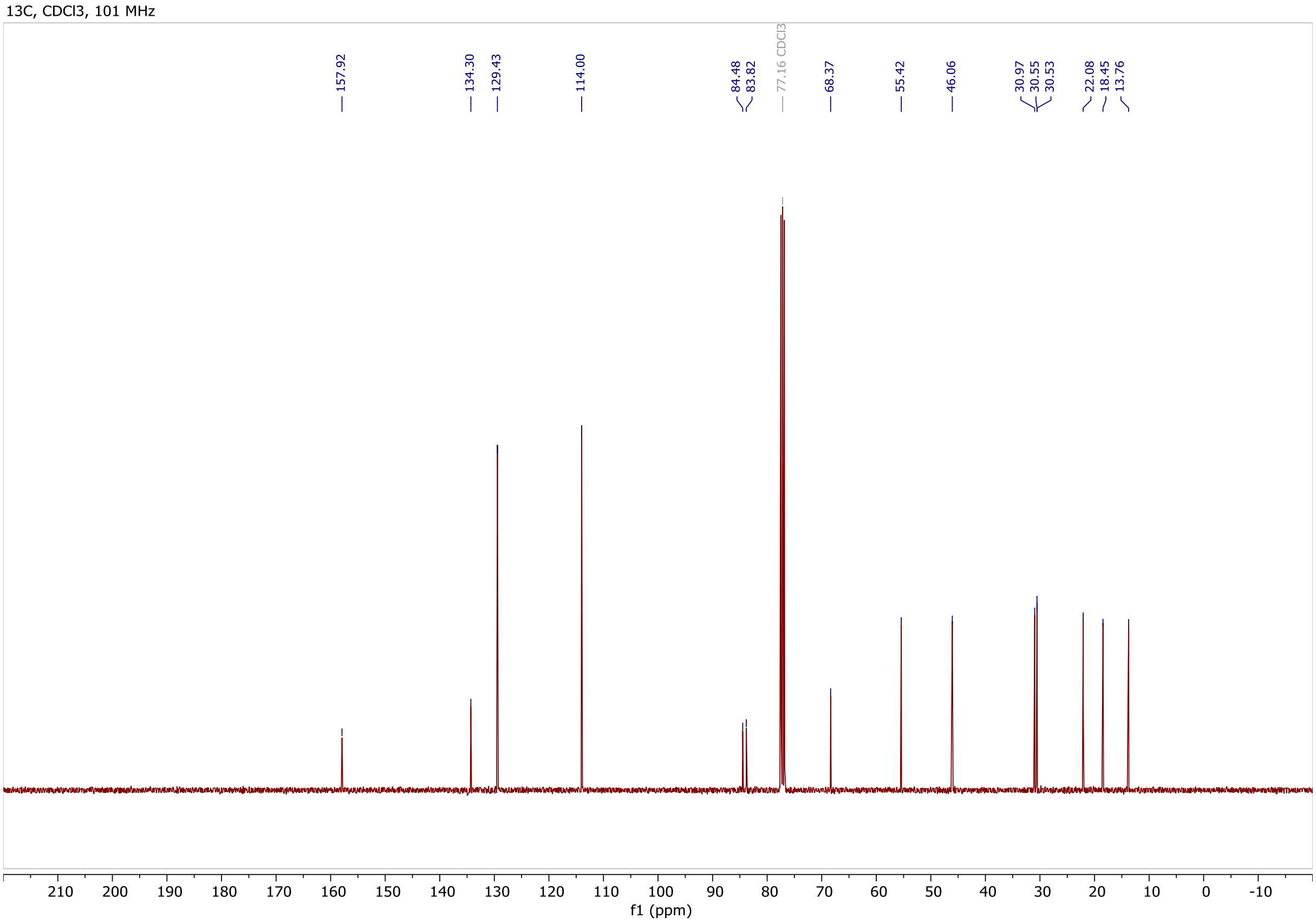

Compound S24

1H, CDCl3, 400 MHz

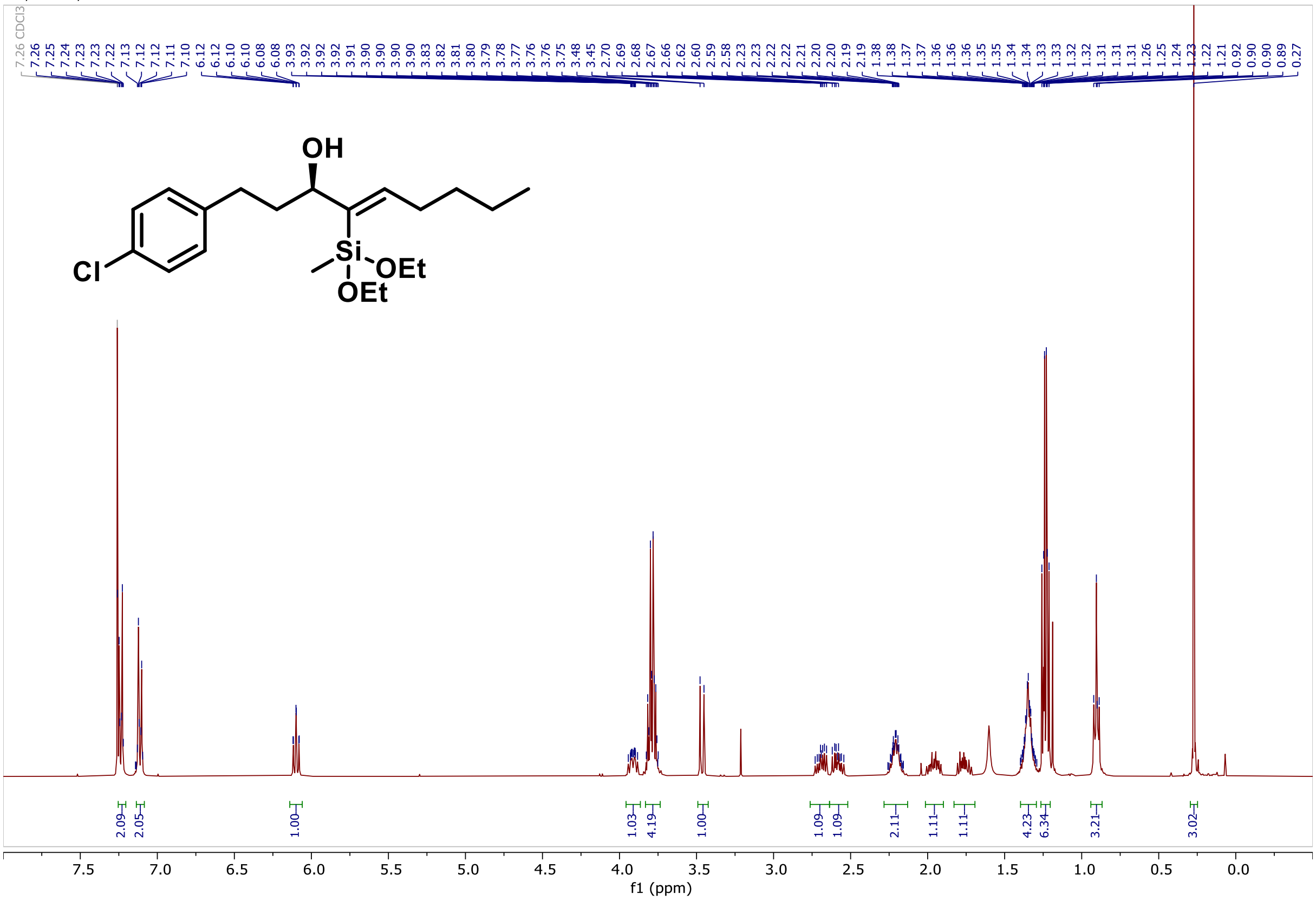

13C, CDCl3, 101 MHz

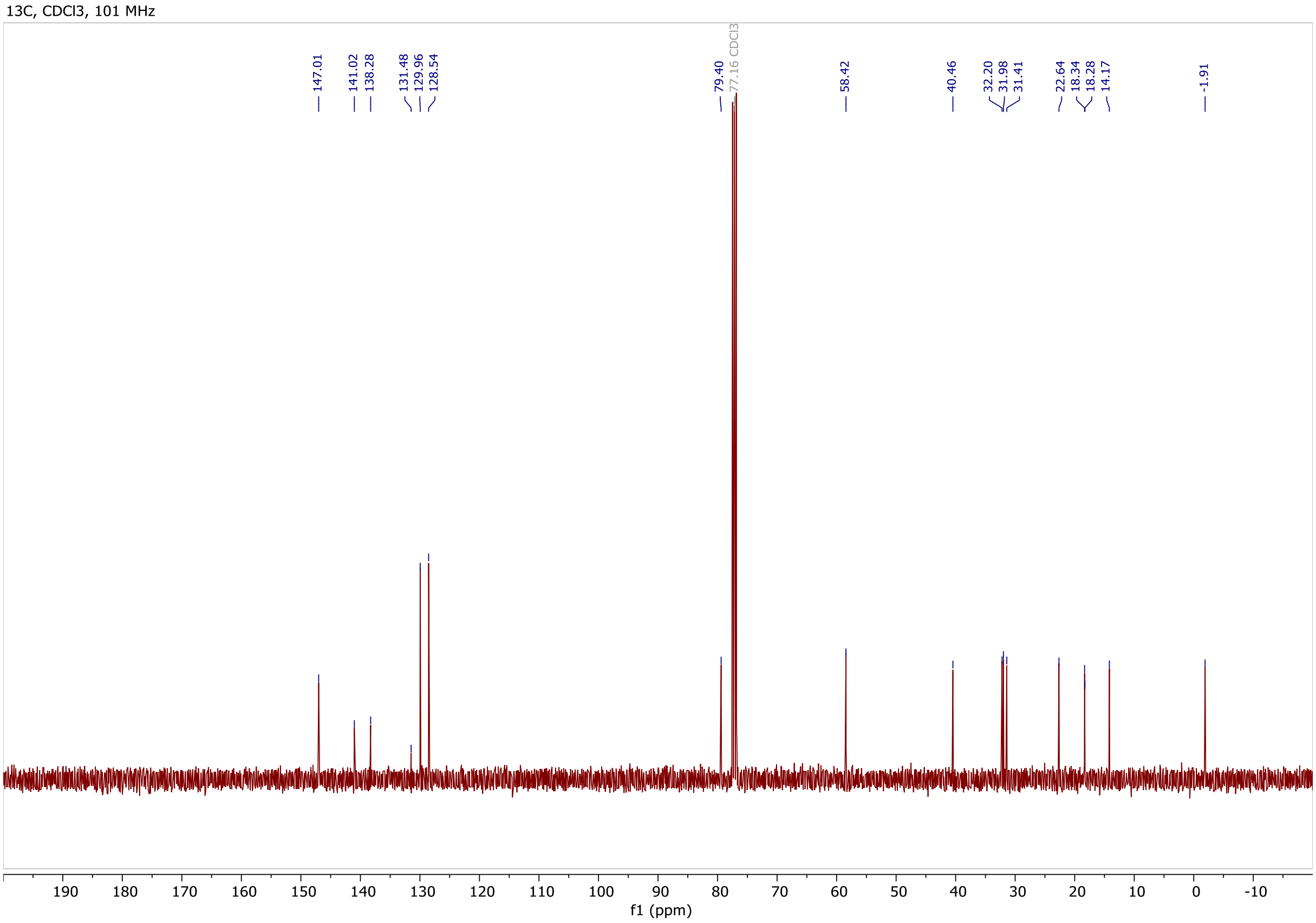

Compound S25

1H, CDCl3, 400 MHz

13C, CDCl3, 101 MHz

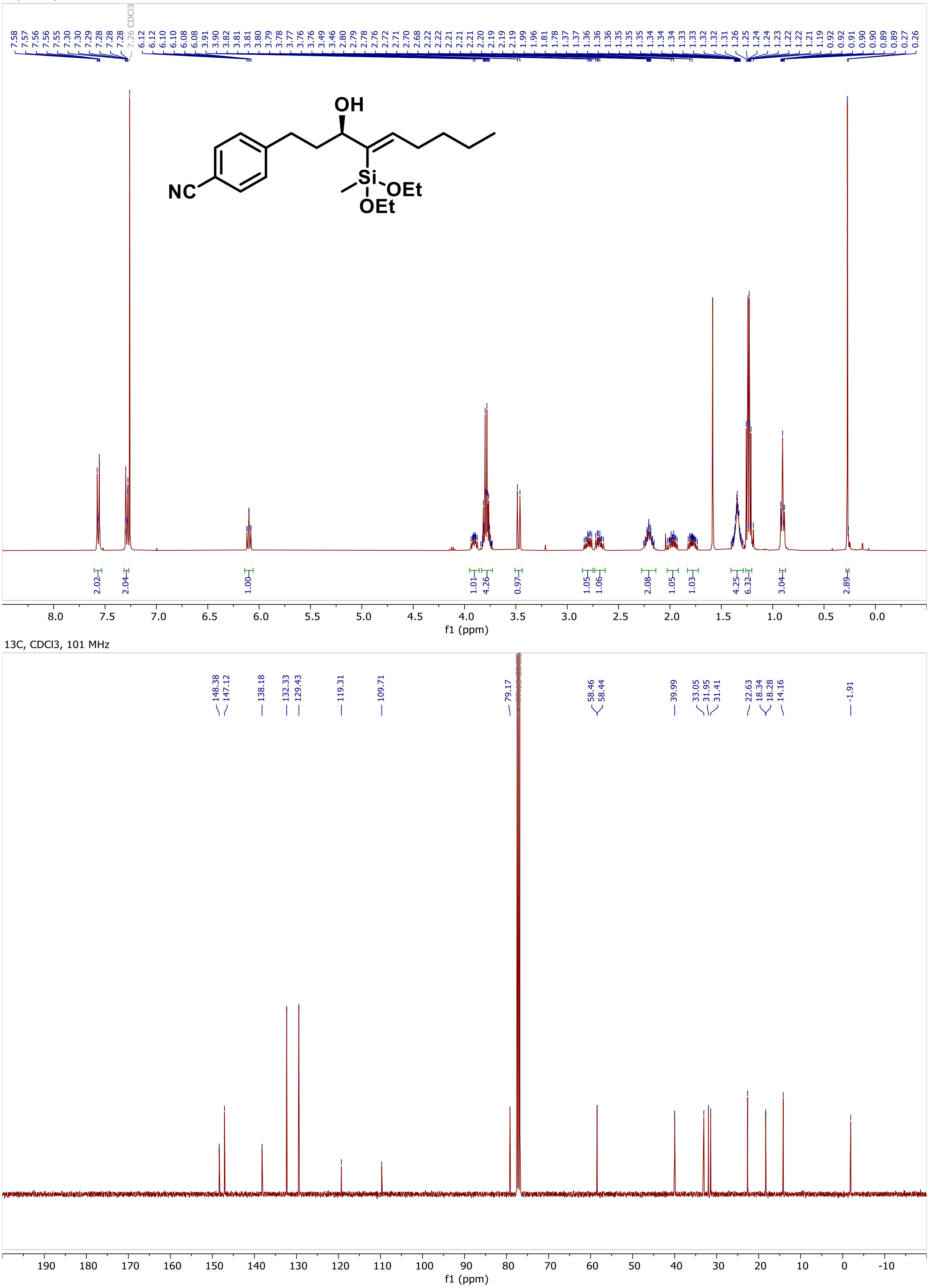

Compound S26

1H, CDCl3, 400 MHz

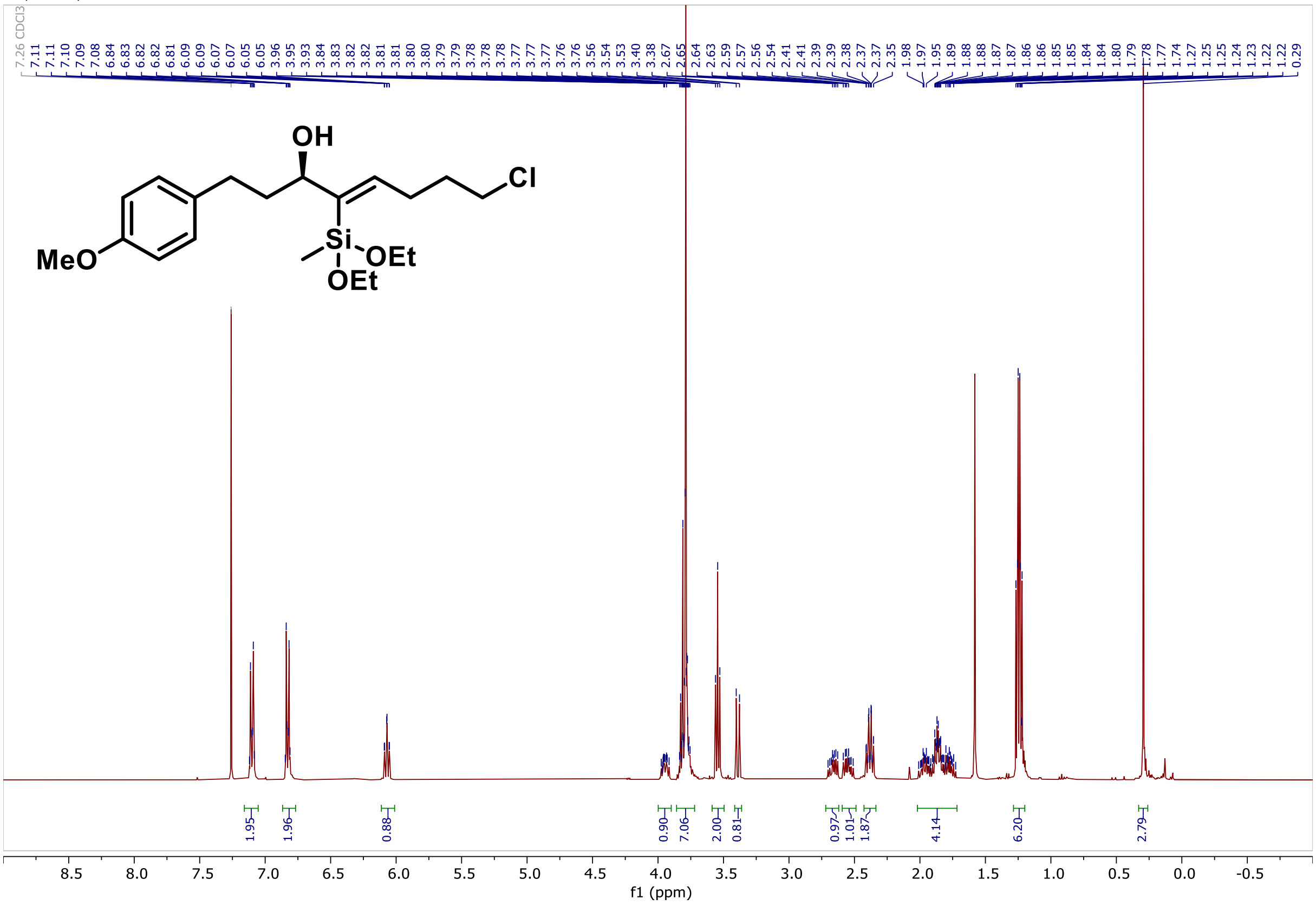

13C, CDCl3, 101 MHz

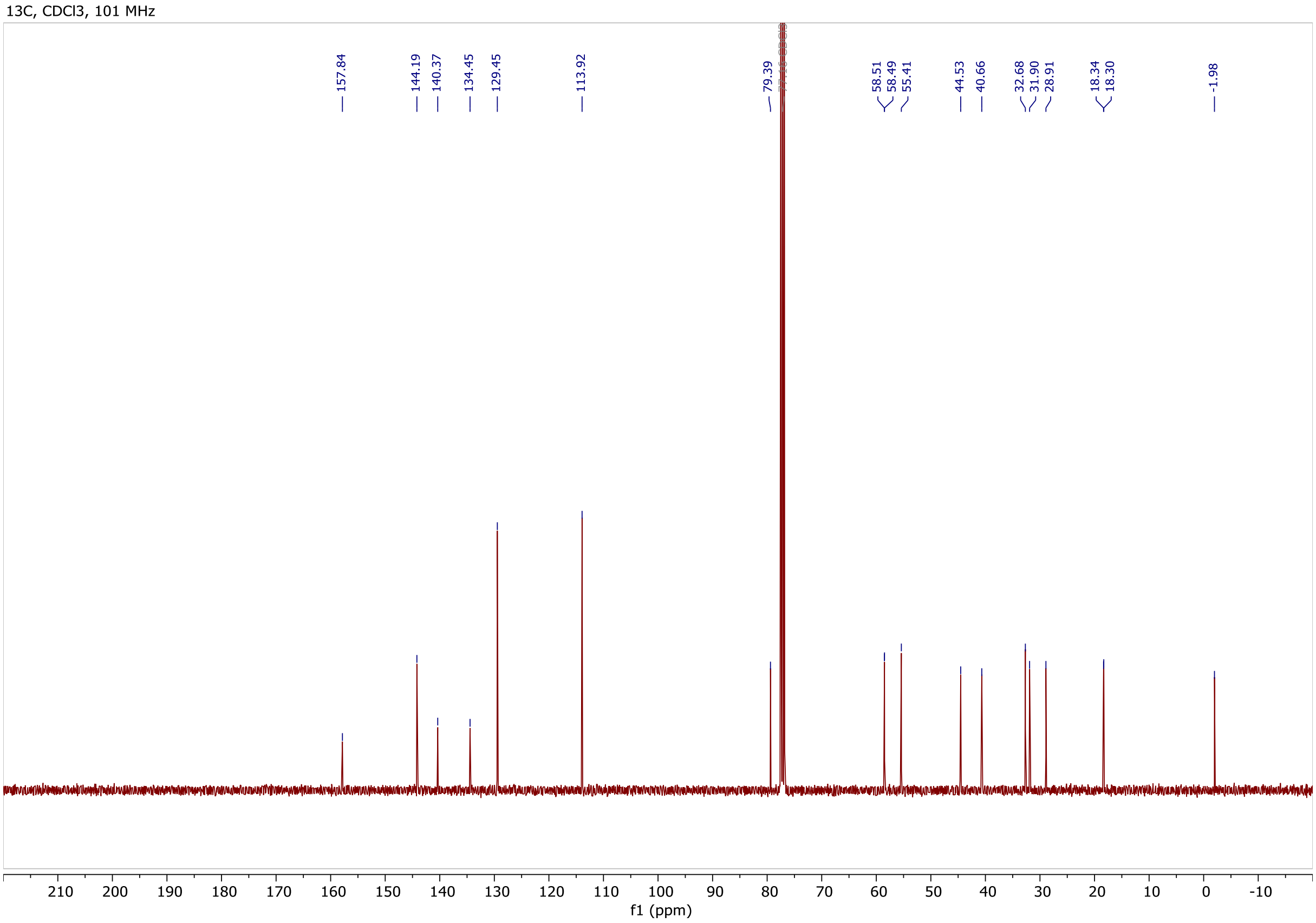

Compound S27

1H, CDCl3, 400 MHz

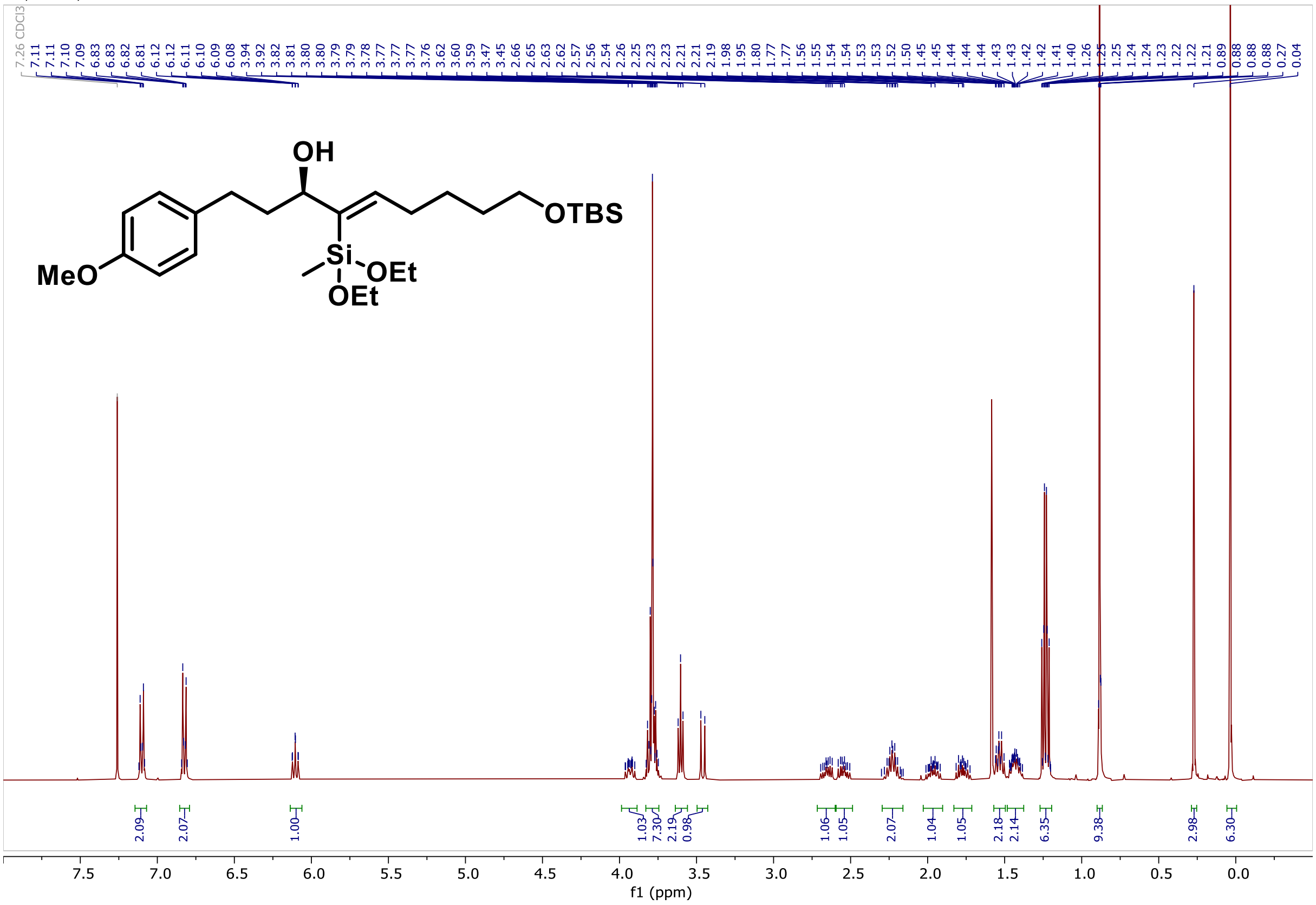

13C, CDCl3, 101 MHz

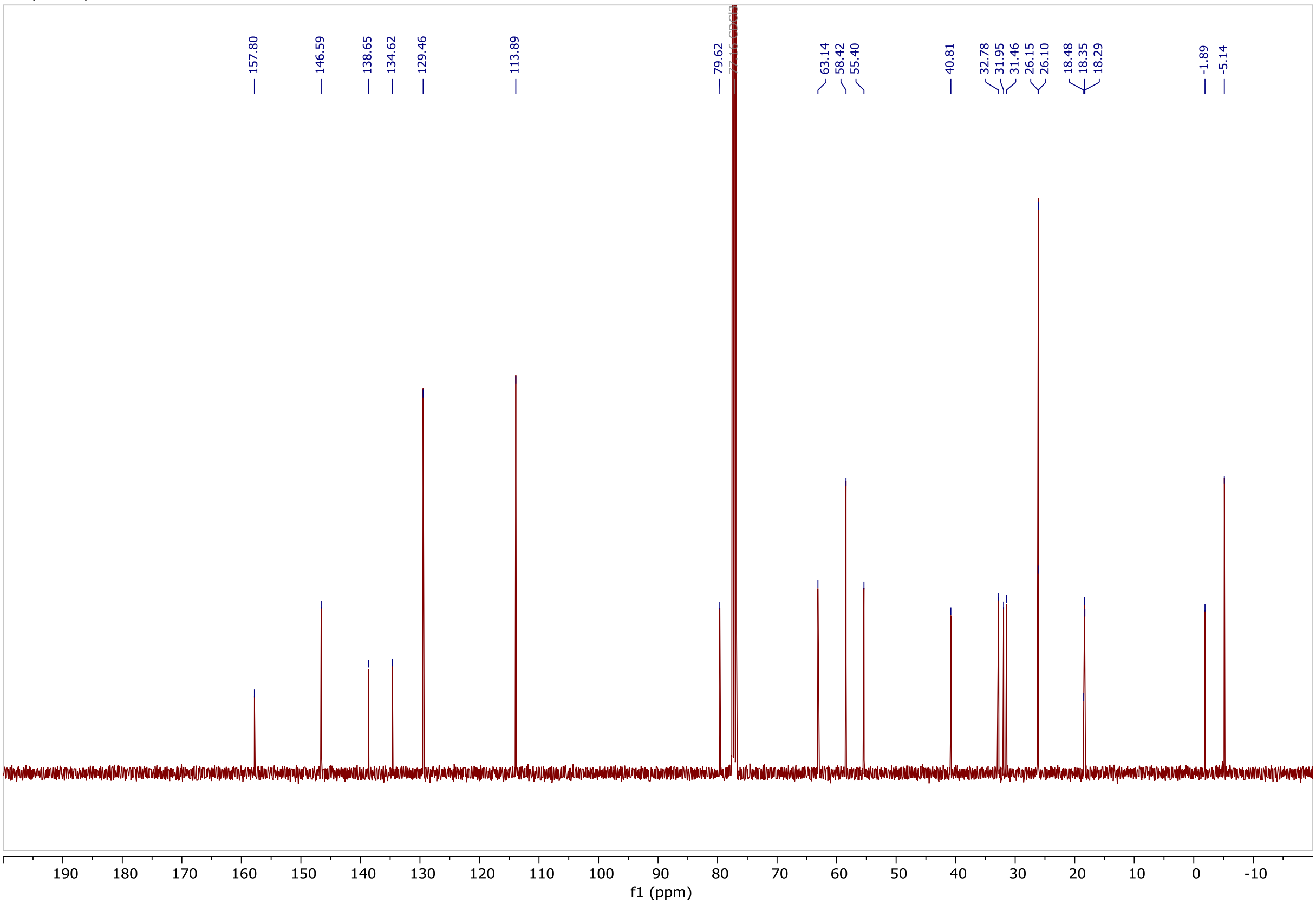

Compound S28

1H, CDCl3, 400 MHz

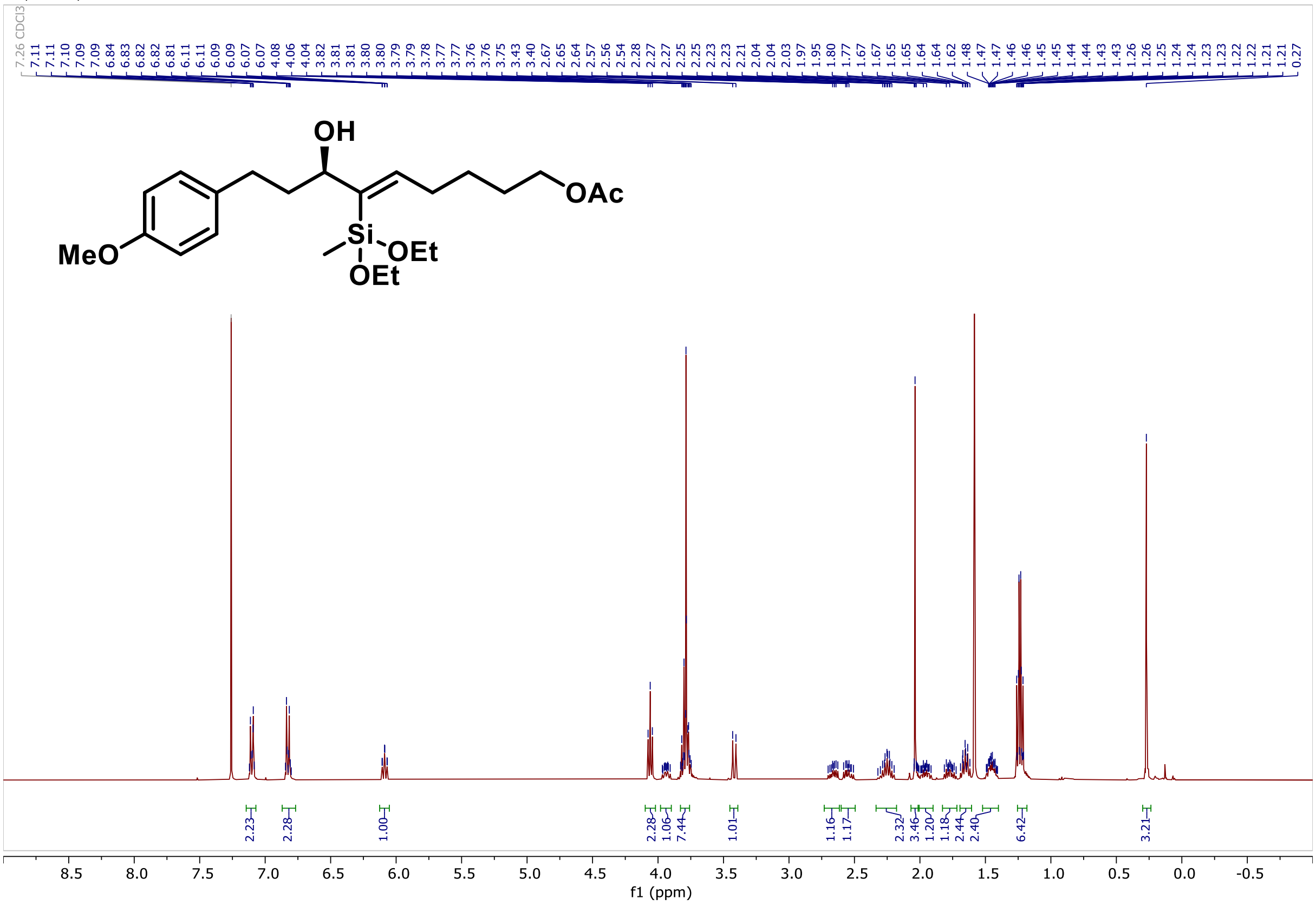

13C, CDCl3, 101 MHz

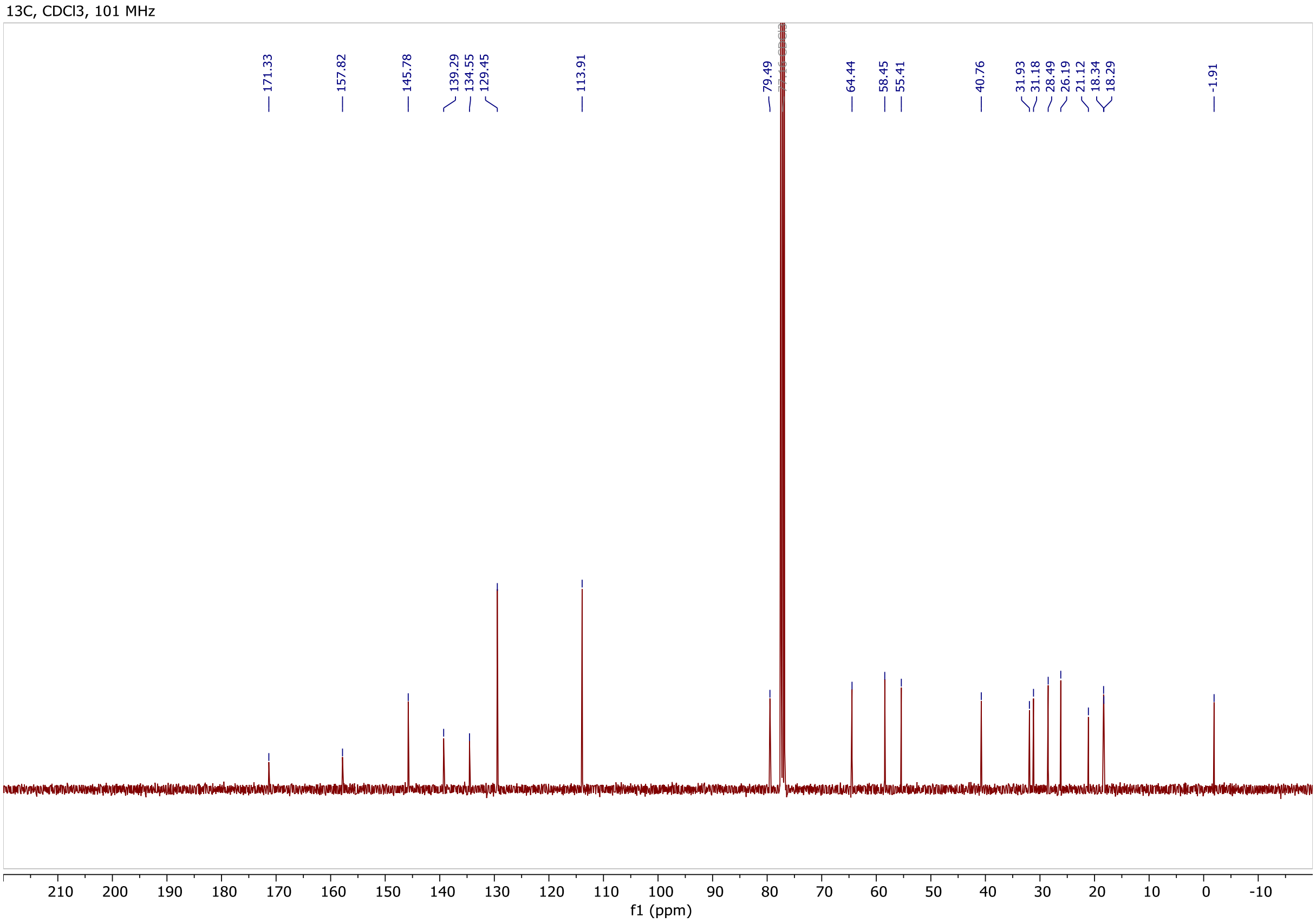

Compound S29

1H, CDCl3, 400 MHz

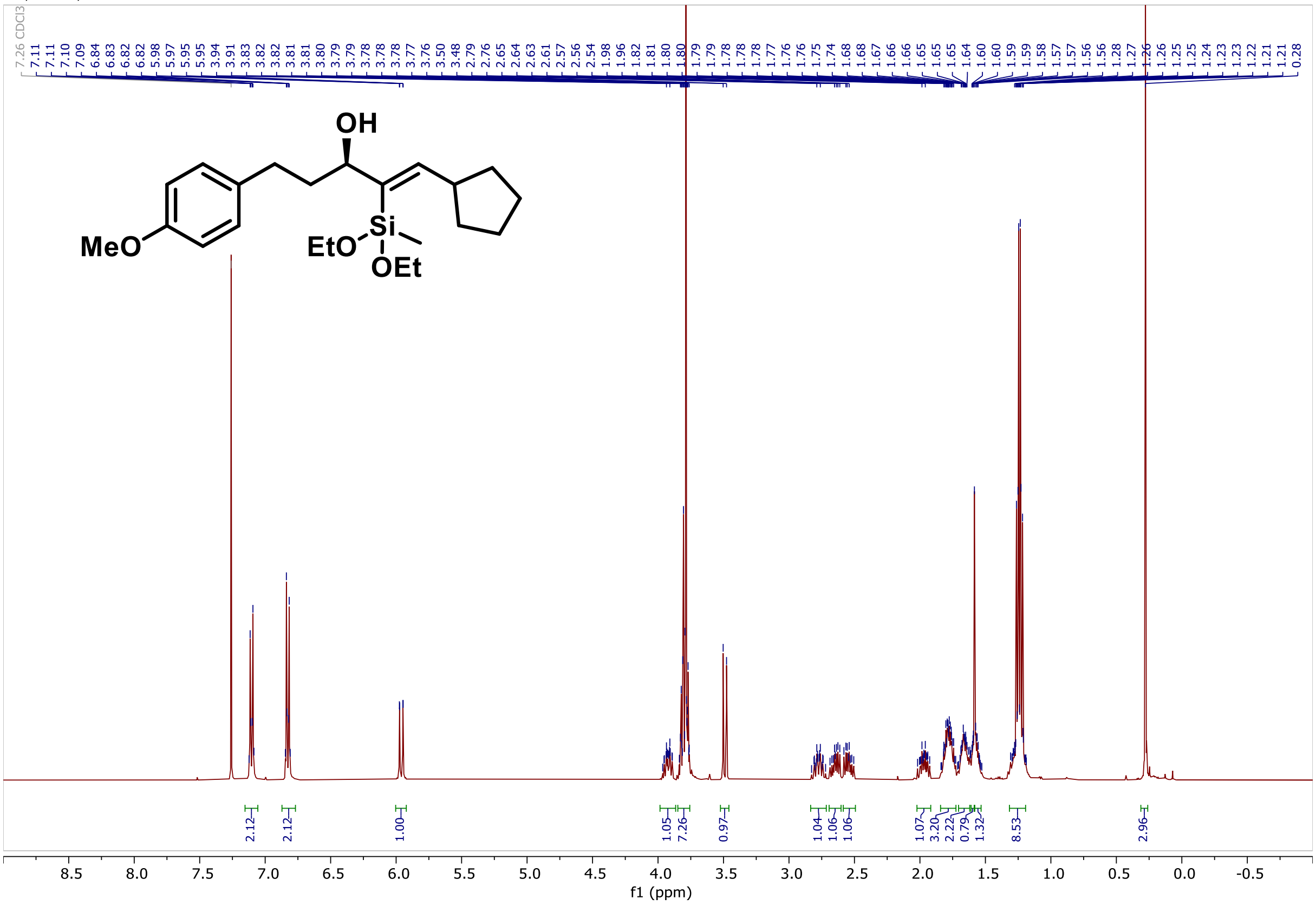

13C, CDCl3, 101 MHz

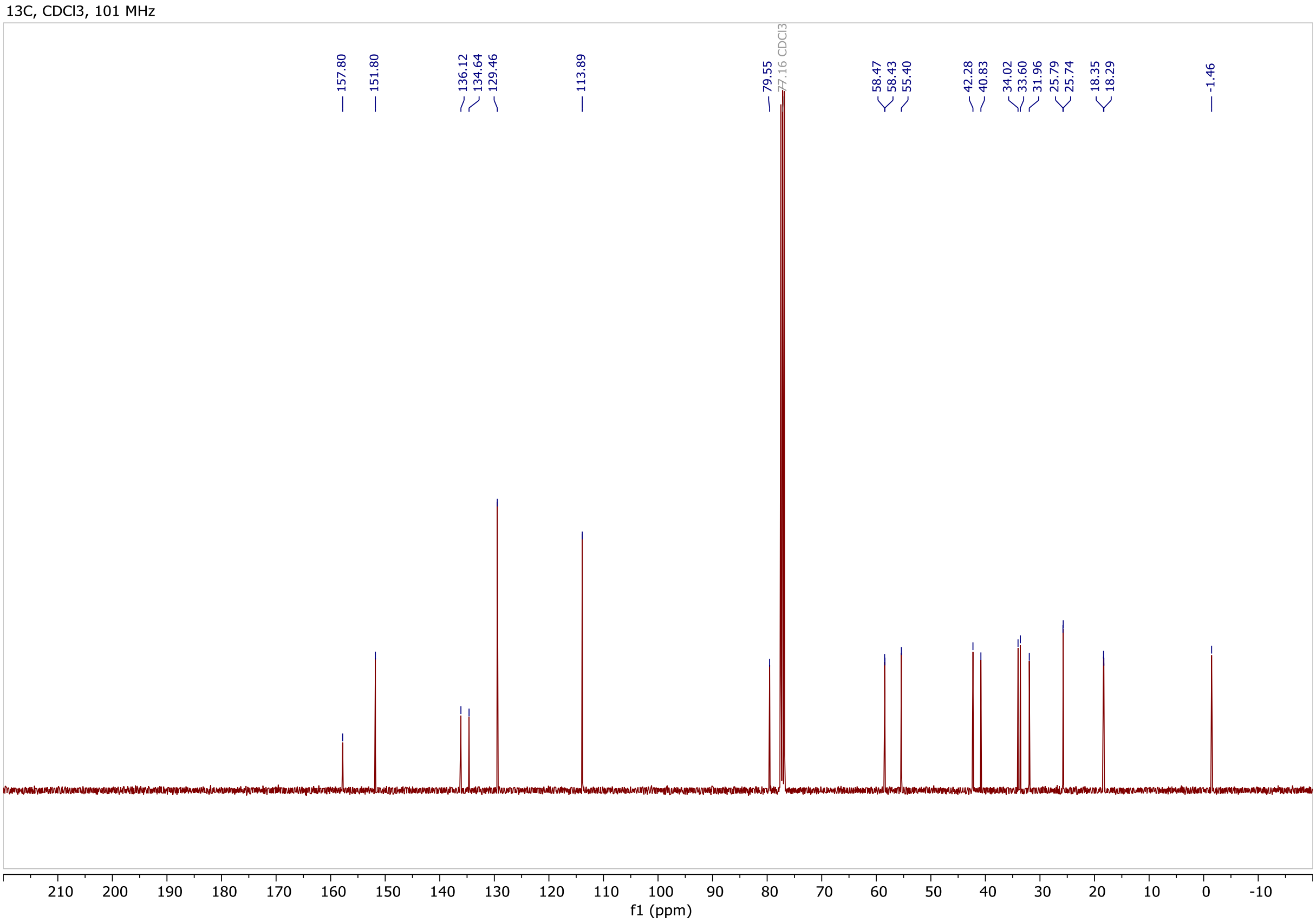

Compound S30

1H, CDCl3, 400 MHz

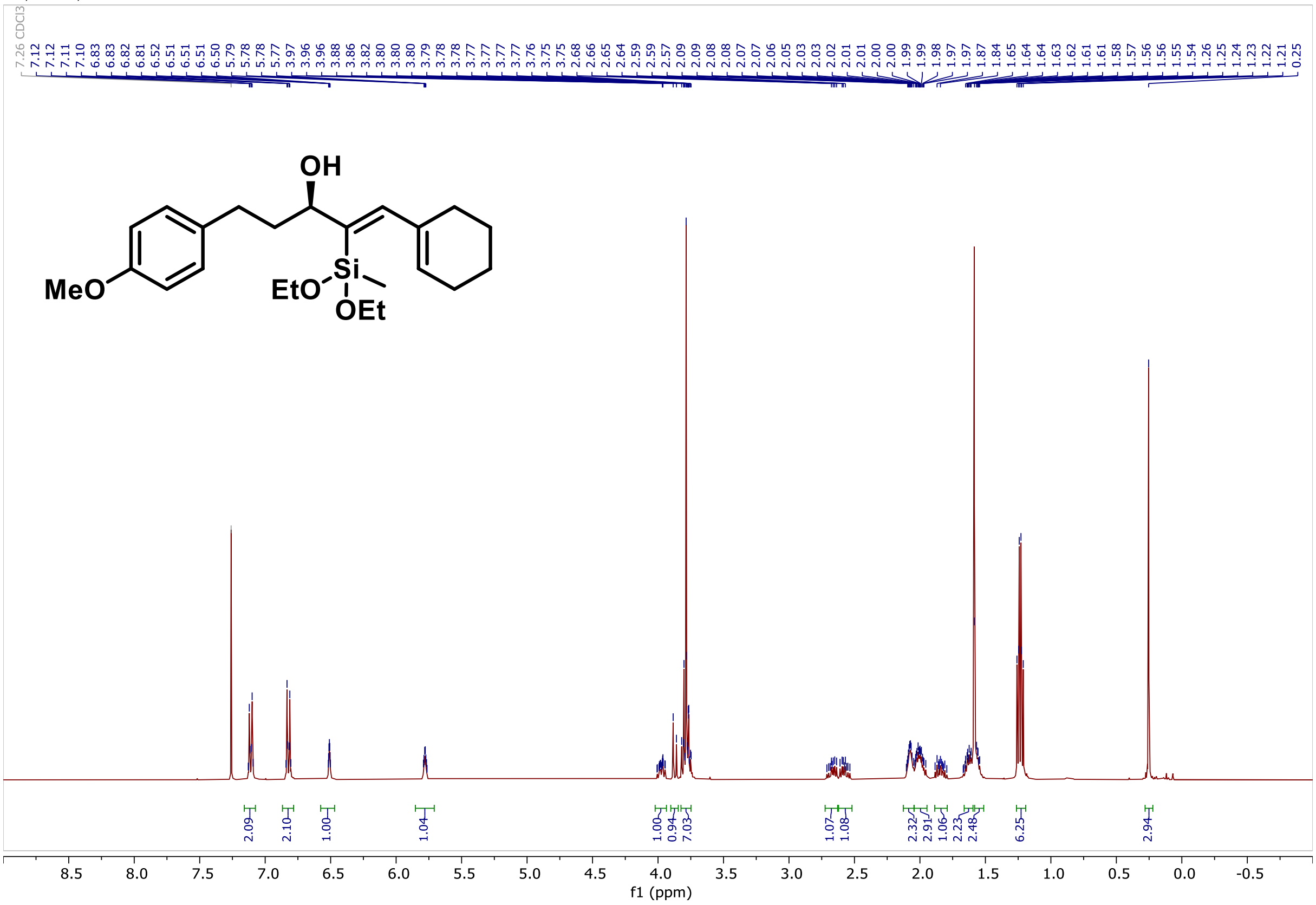

13C, CDCl3, 101 MHz

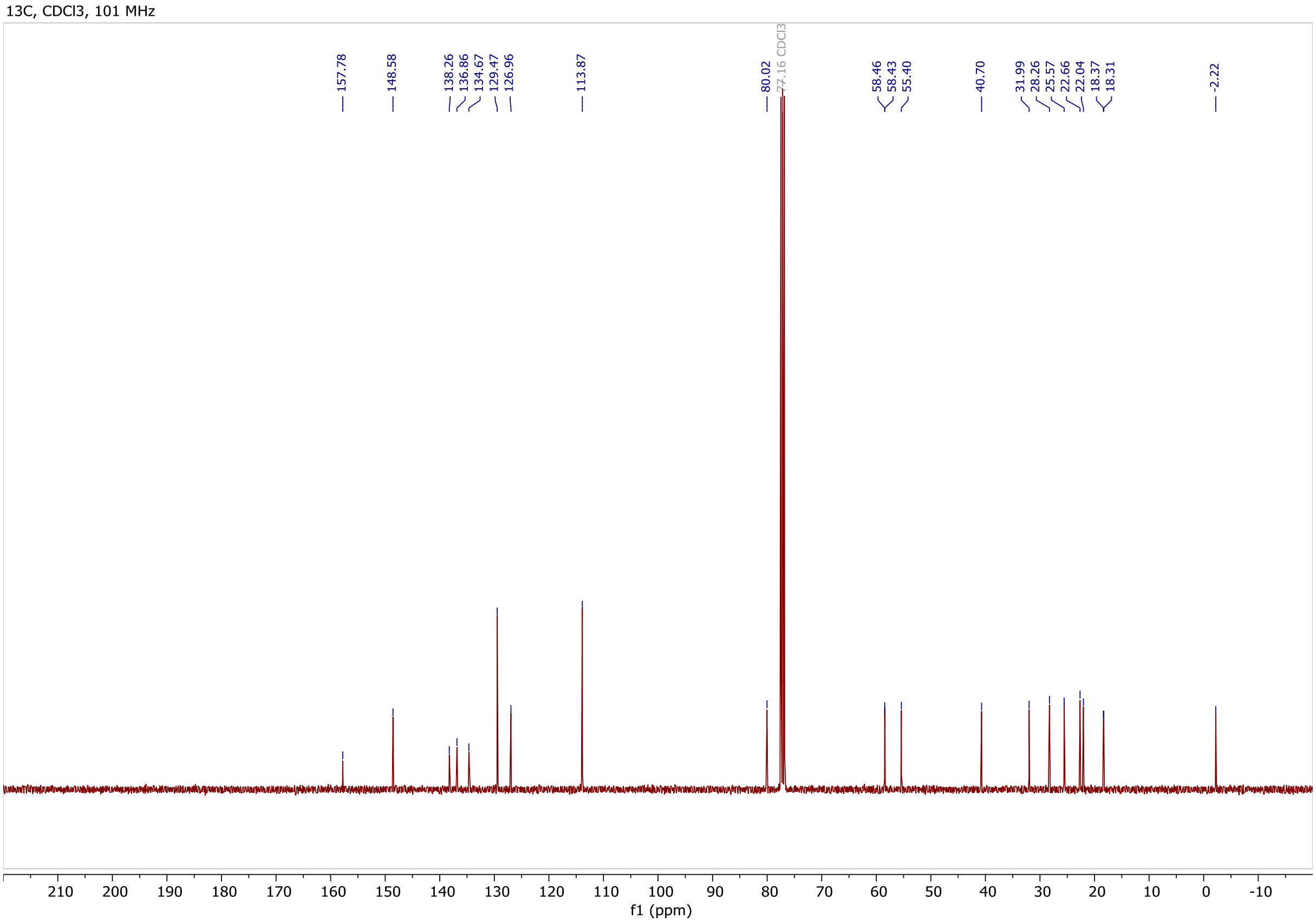

Compound S31

1H, CDCl3, 400 MHz

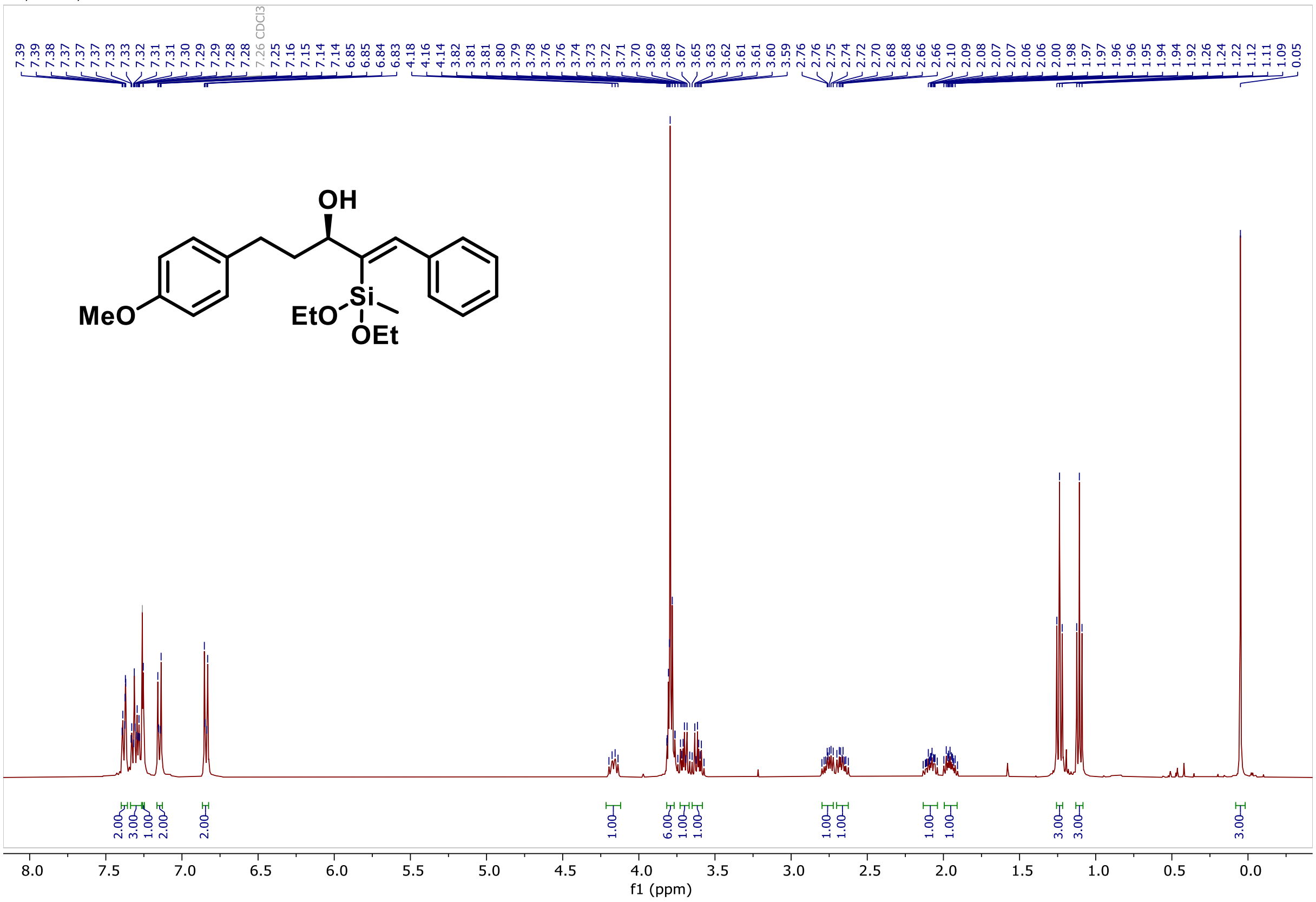

13C, CDCl3, 101 MHz

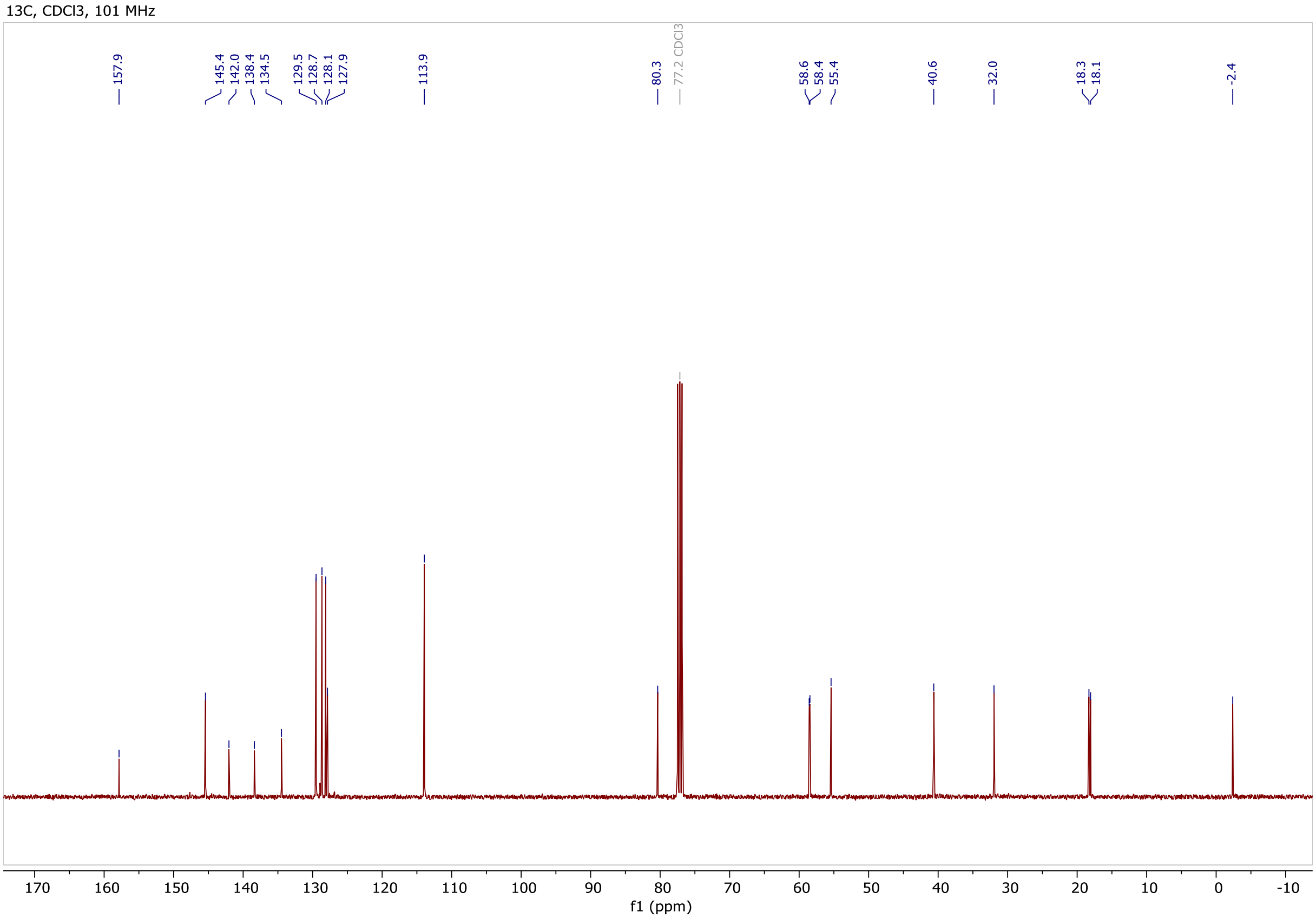

Compound S32

1H, CDCl3, 400 MHz

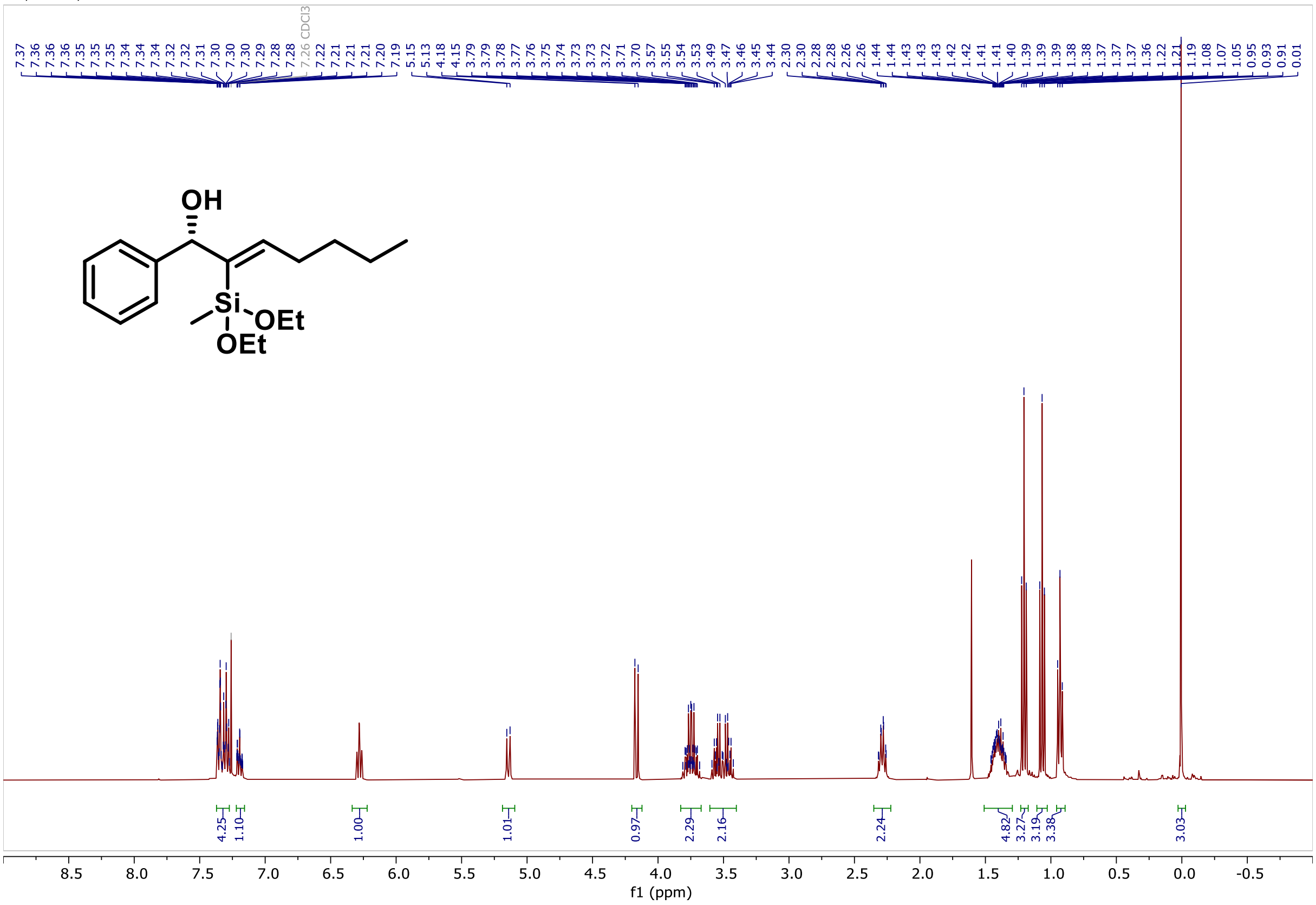

13C, CDCl3, 101 MHz

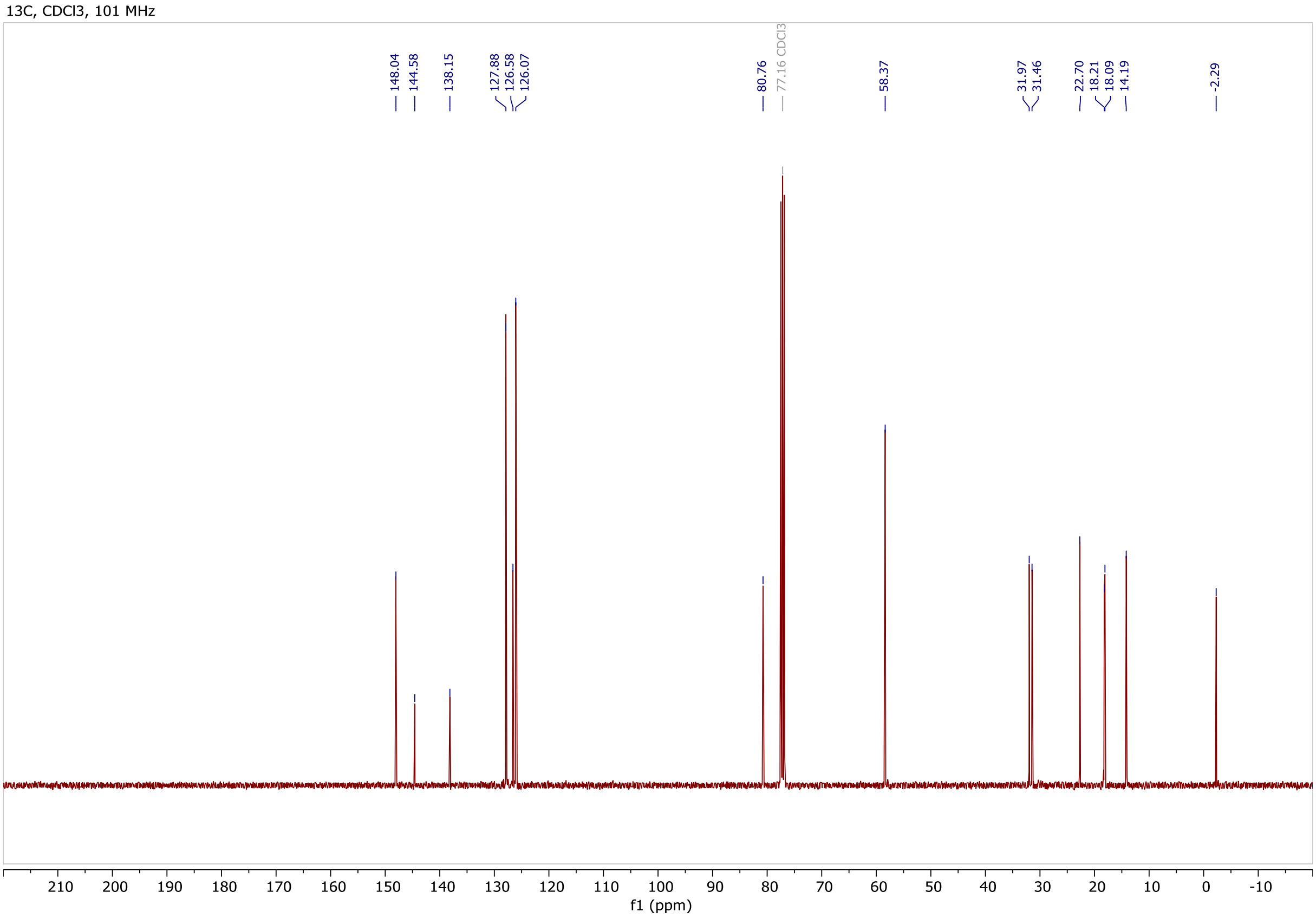

Compound S33

1H, CDCl3, 400 MHz

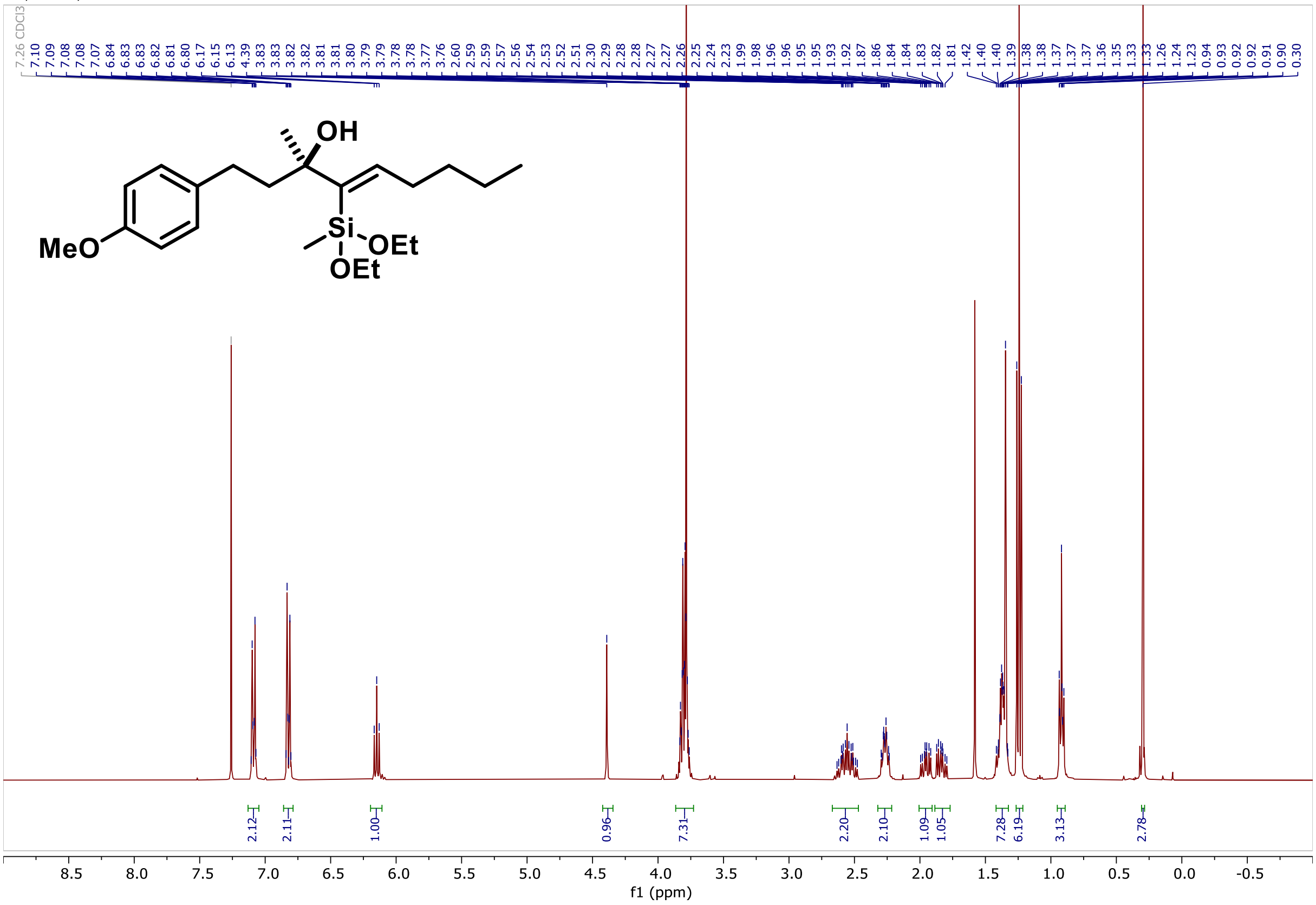

13C, CDCl3, 101 MHz

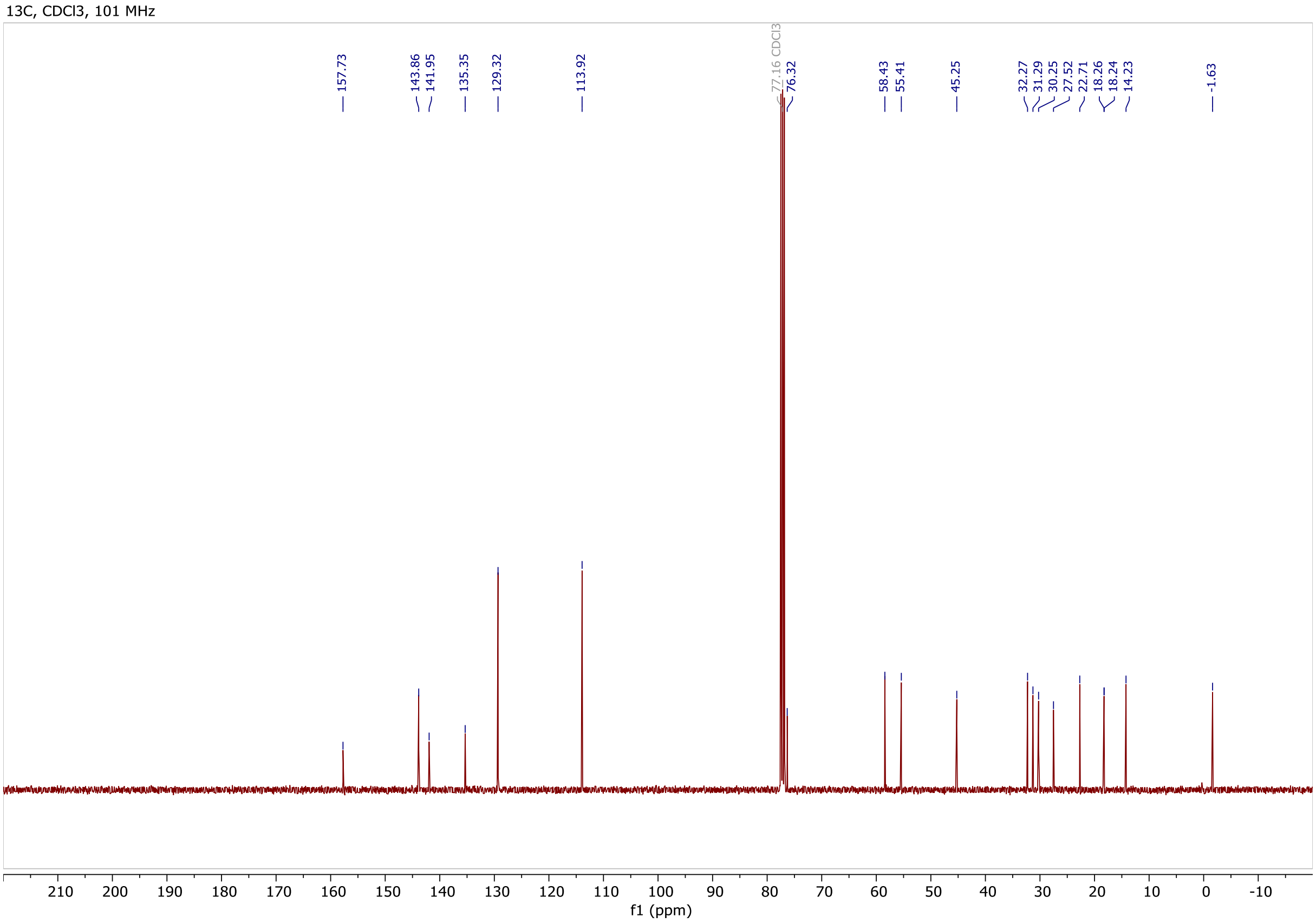

Compound S34

1H, CDCl3, 400 MHz

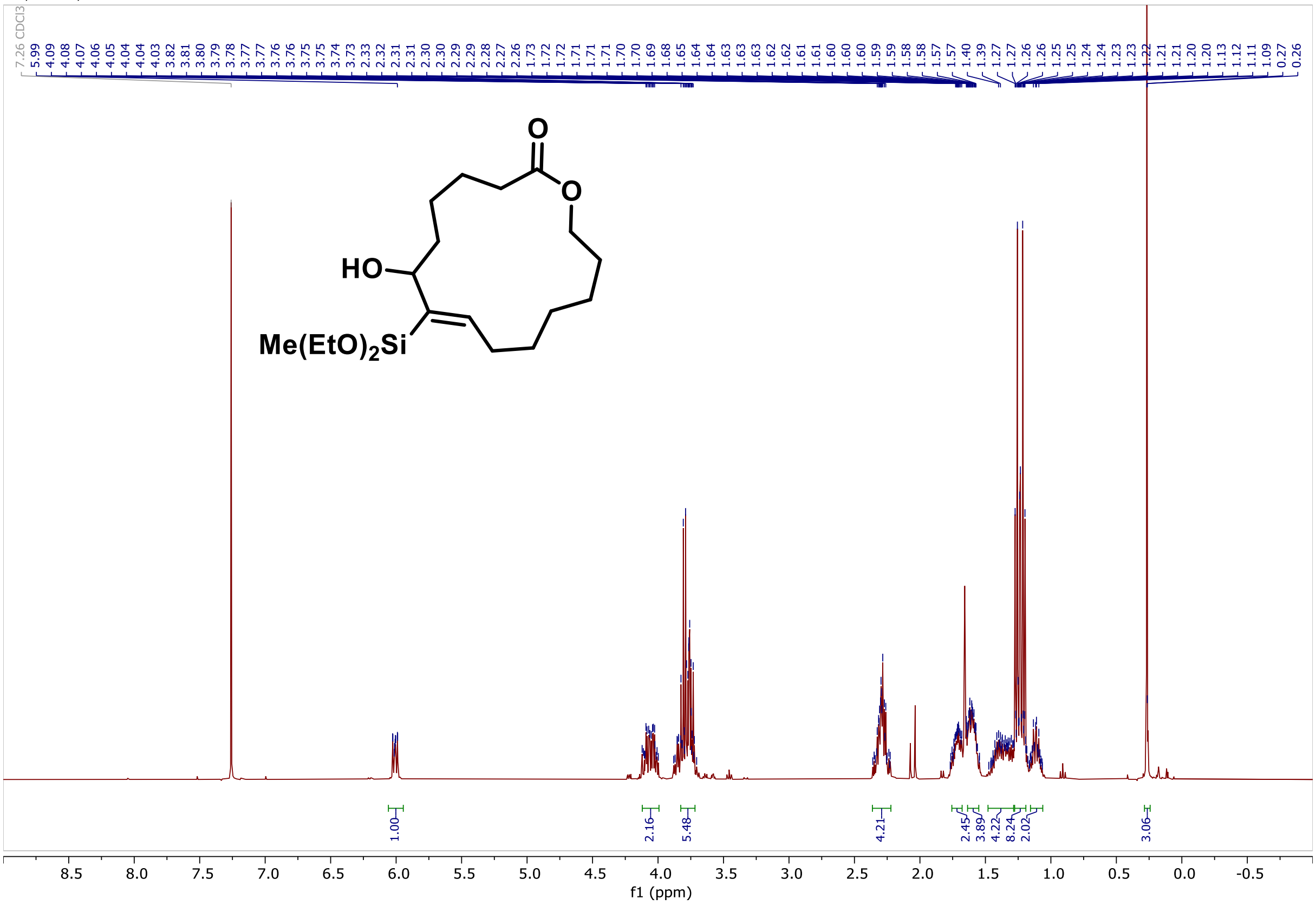

13C, CDCl3, 101 MHz

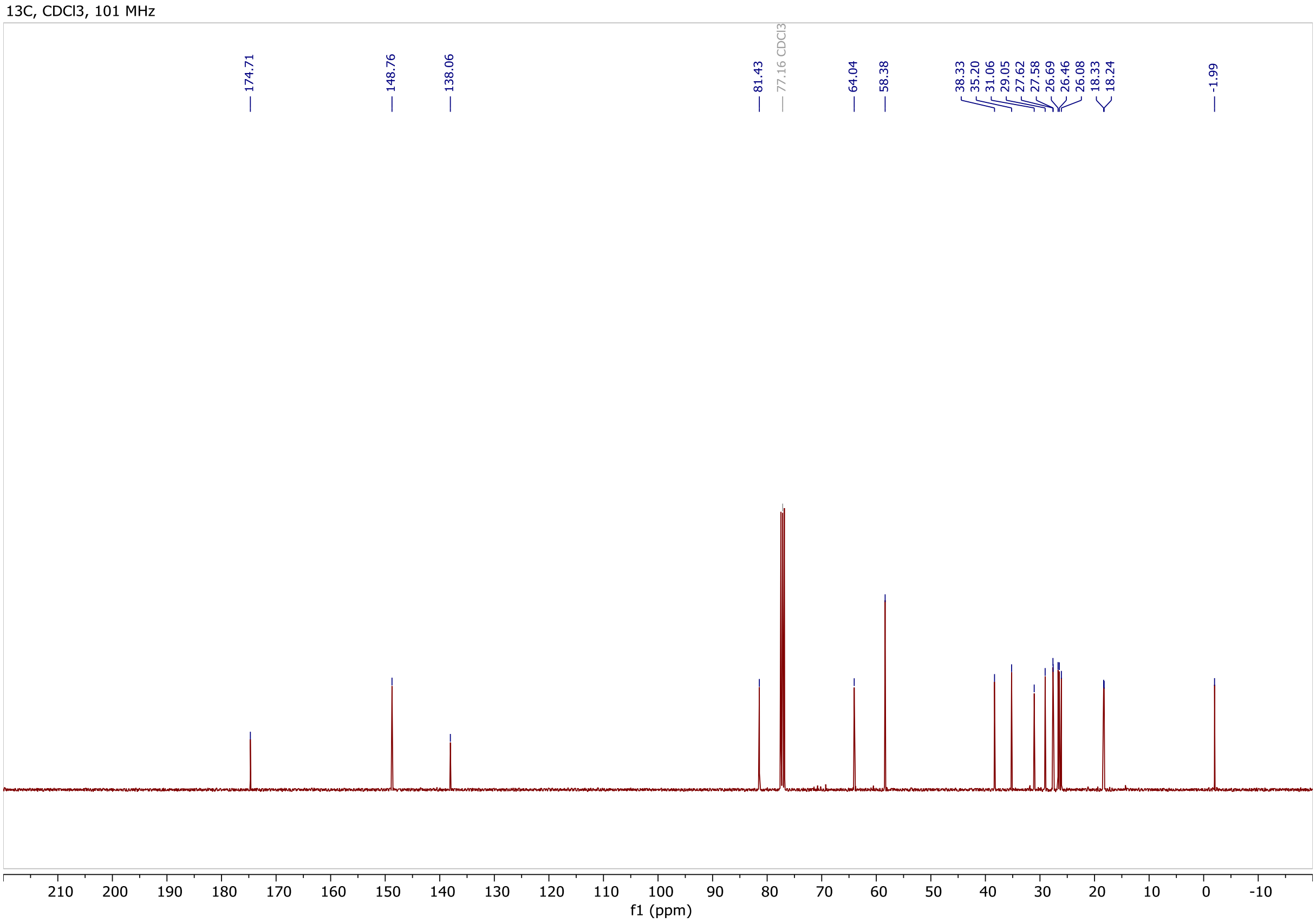

Compound 1

1H, CDCl3, 400 MHz

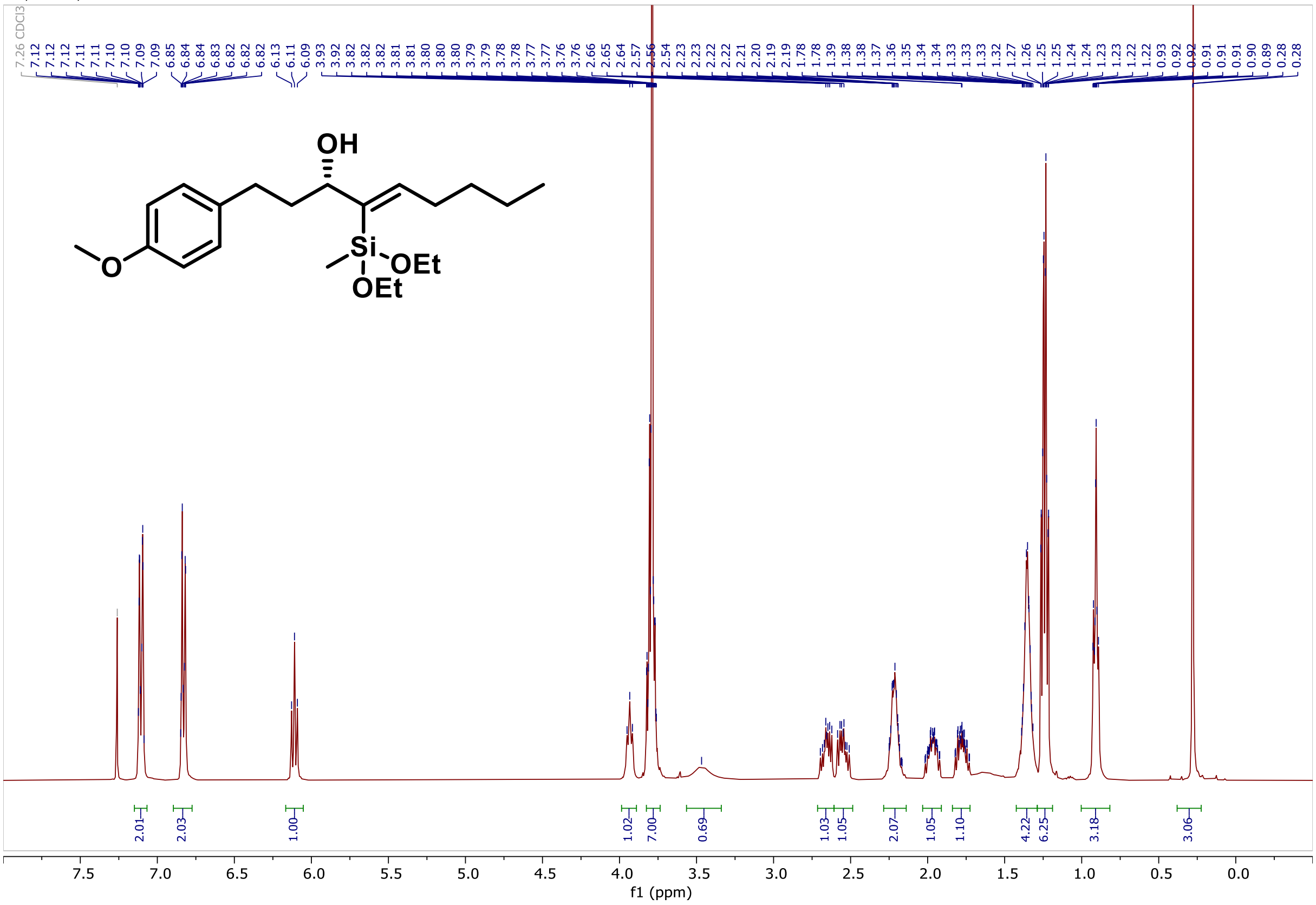

13C, CDCl3, 101 MHz

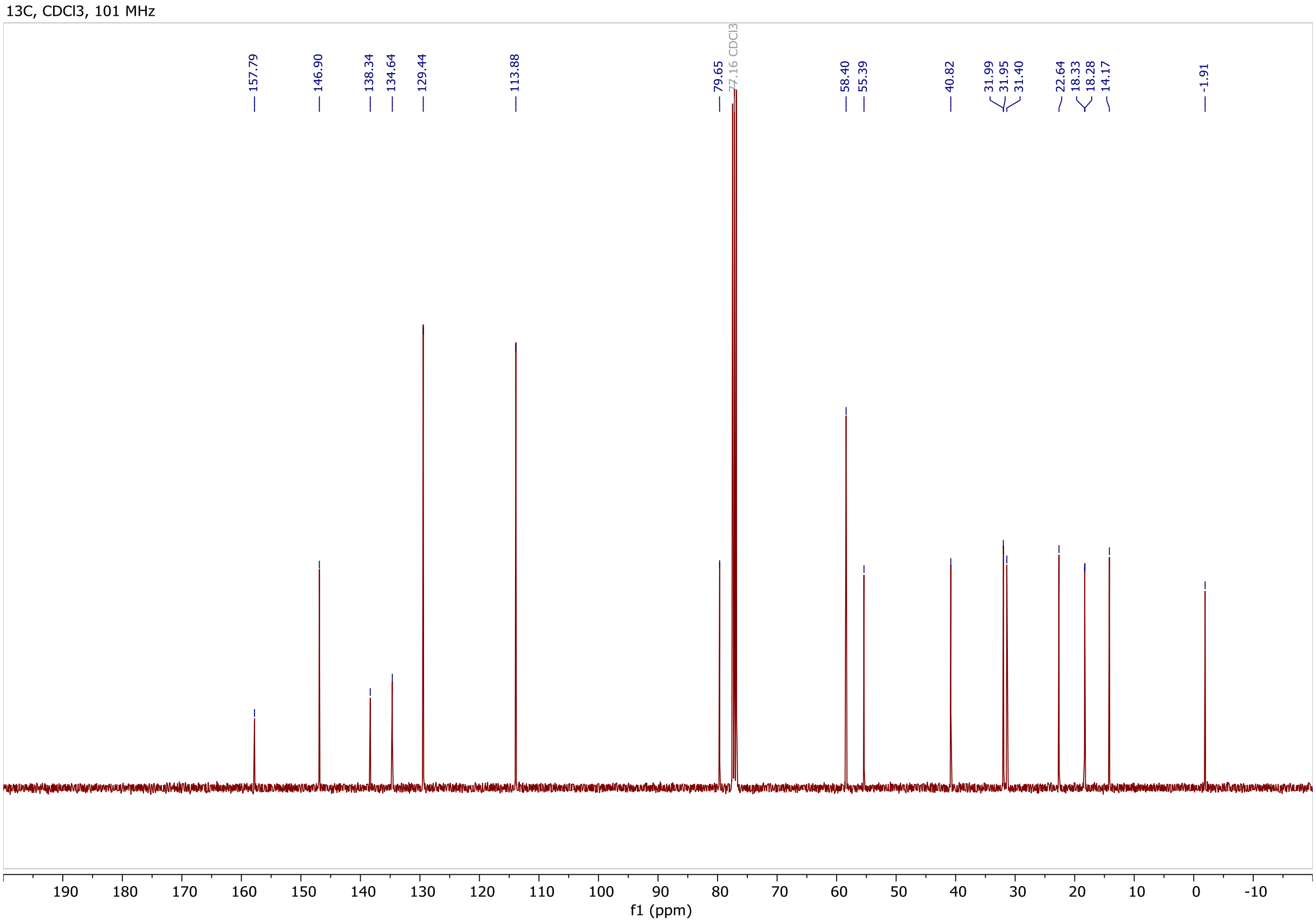

Compound 2

1H, CDCl3, 400 MHz

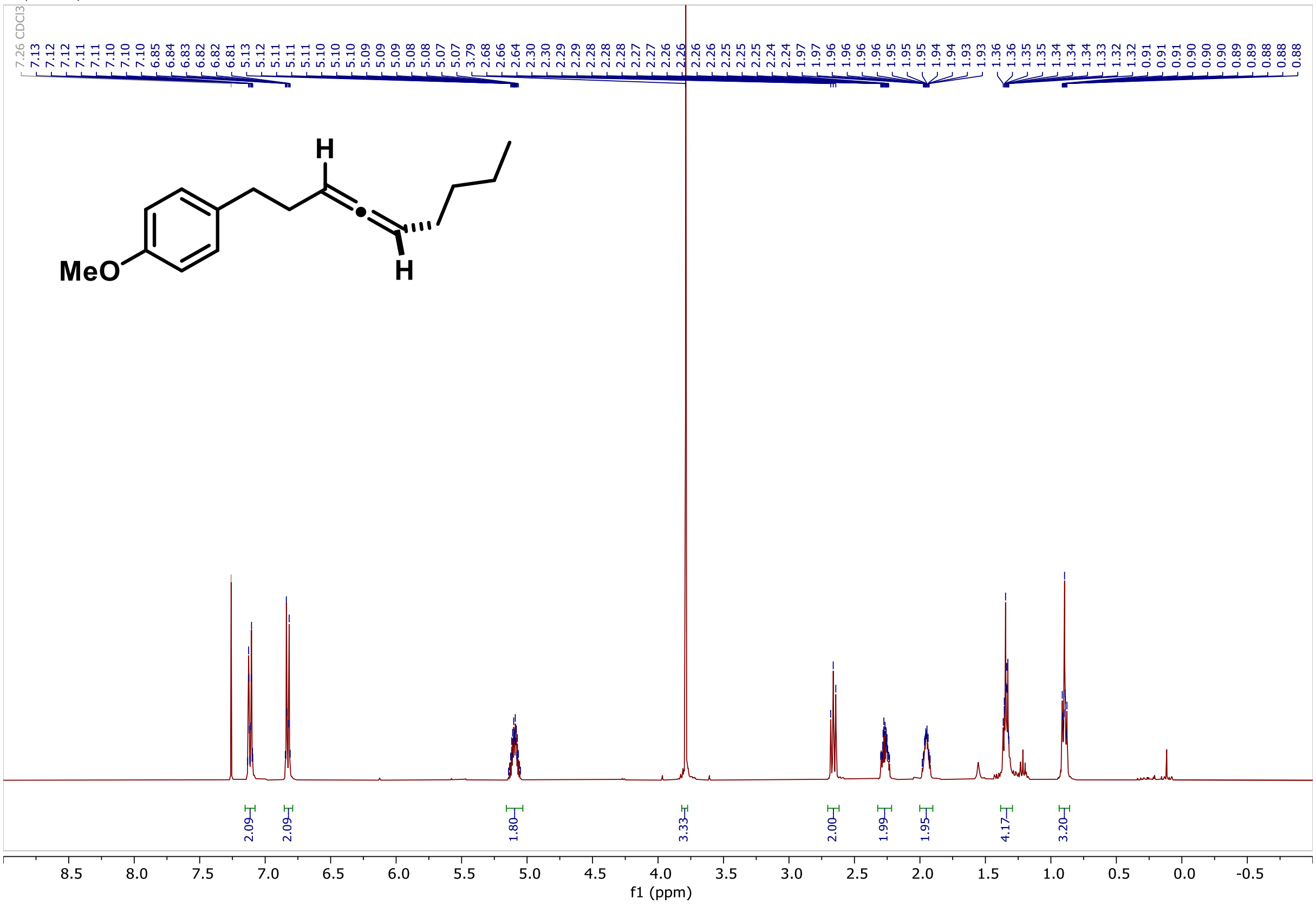

13C, CDCl3, 101 MHz

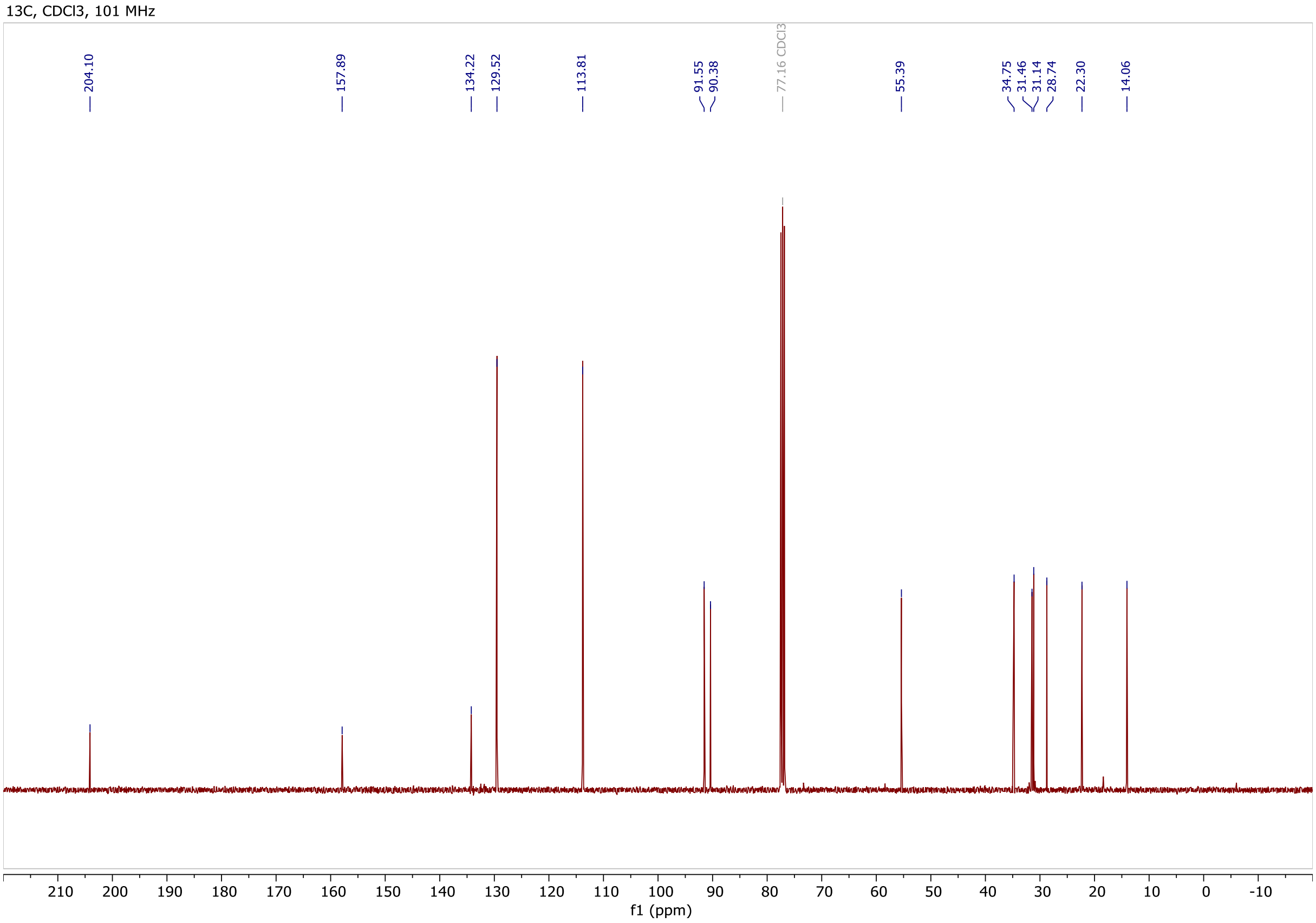

Compound 3

1H, CDCl3, 400 MHz

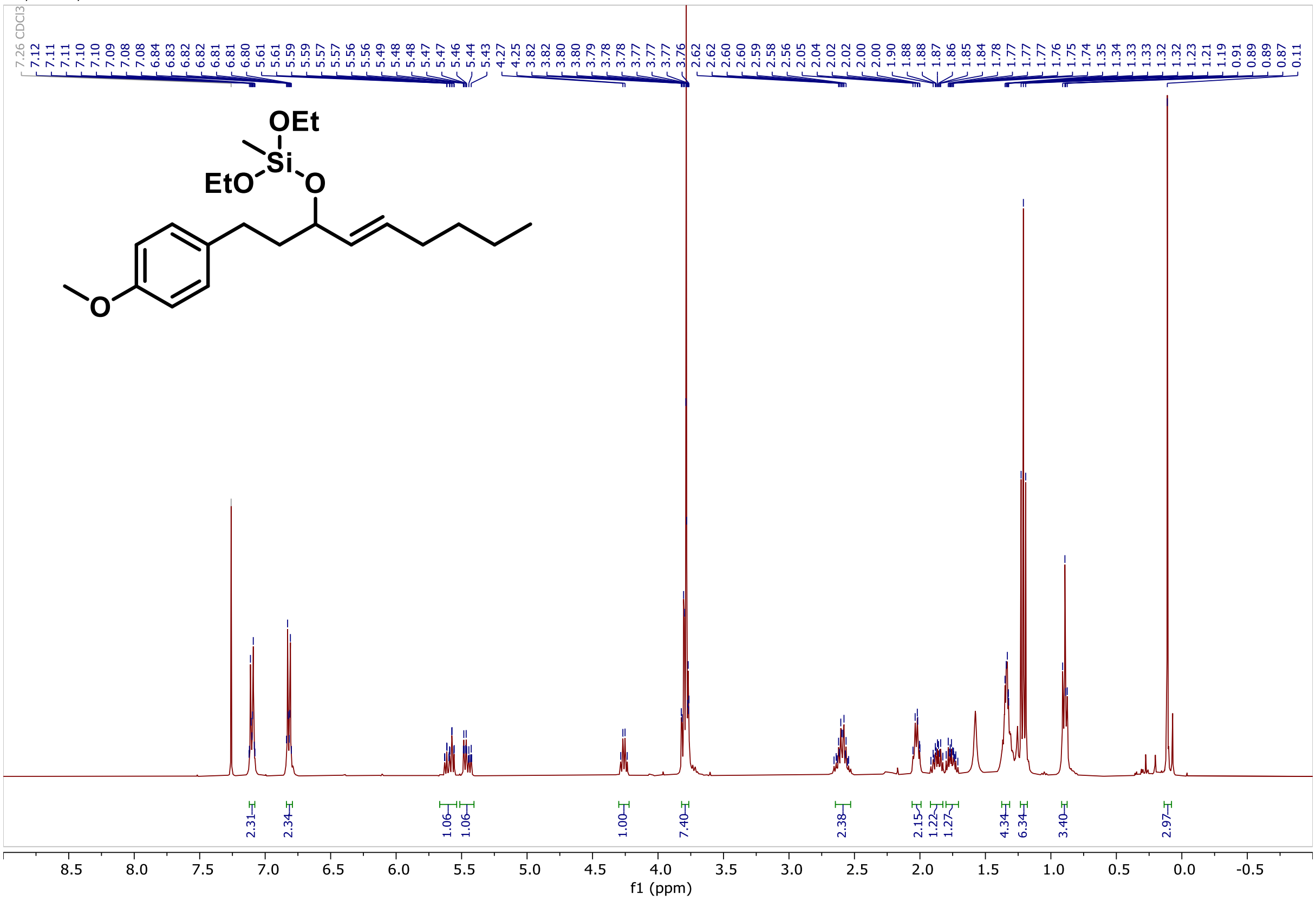

13C, CDCl3, 101 MHz

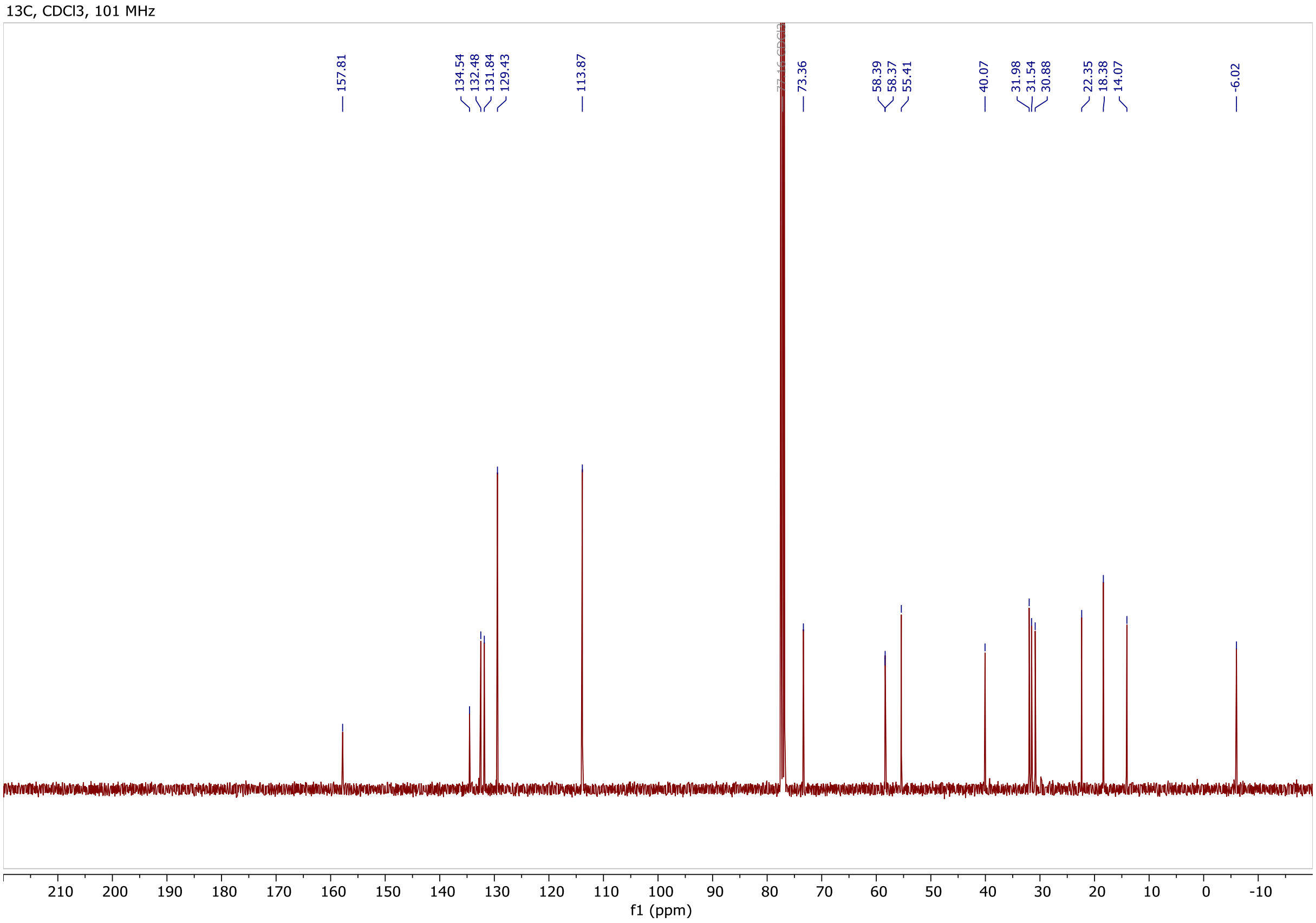

Compound 5

1H, CDCl3, 400 MHz

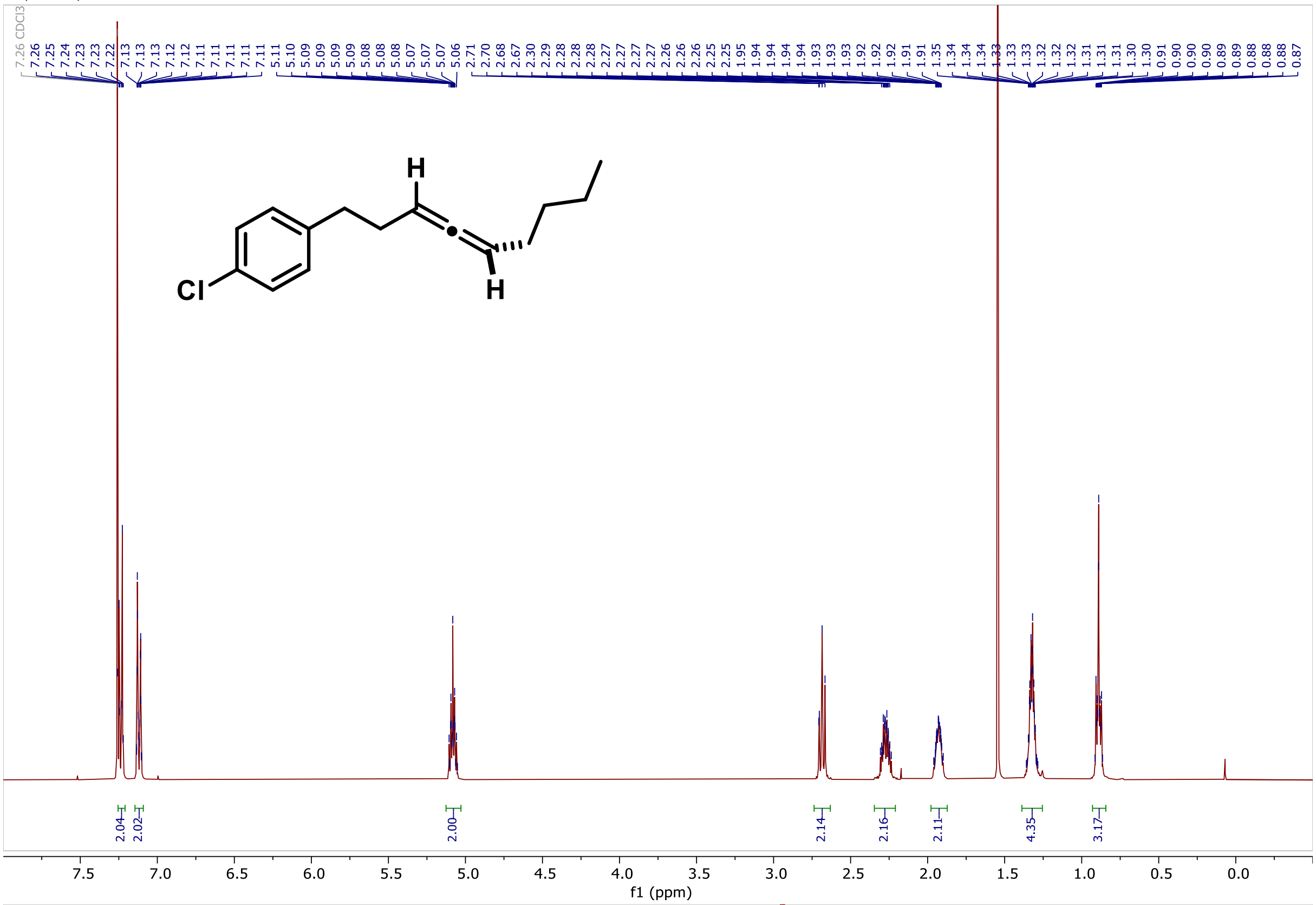

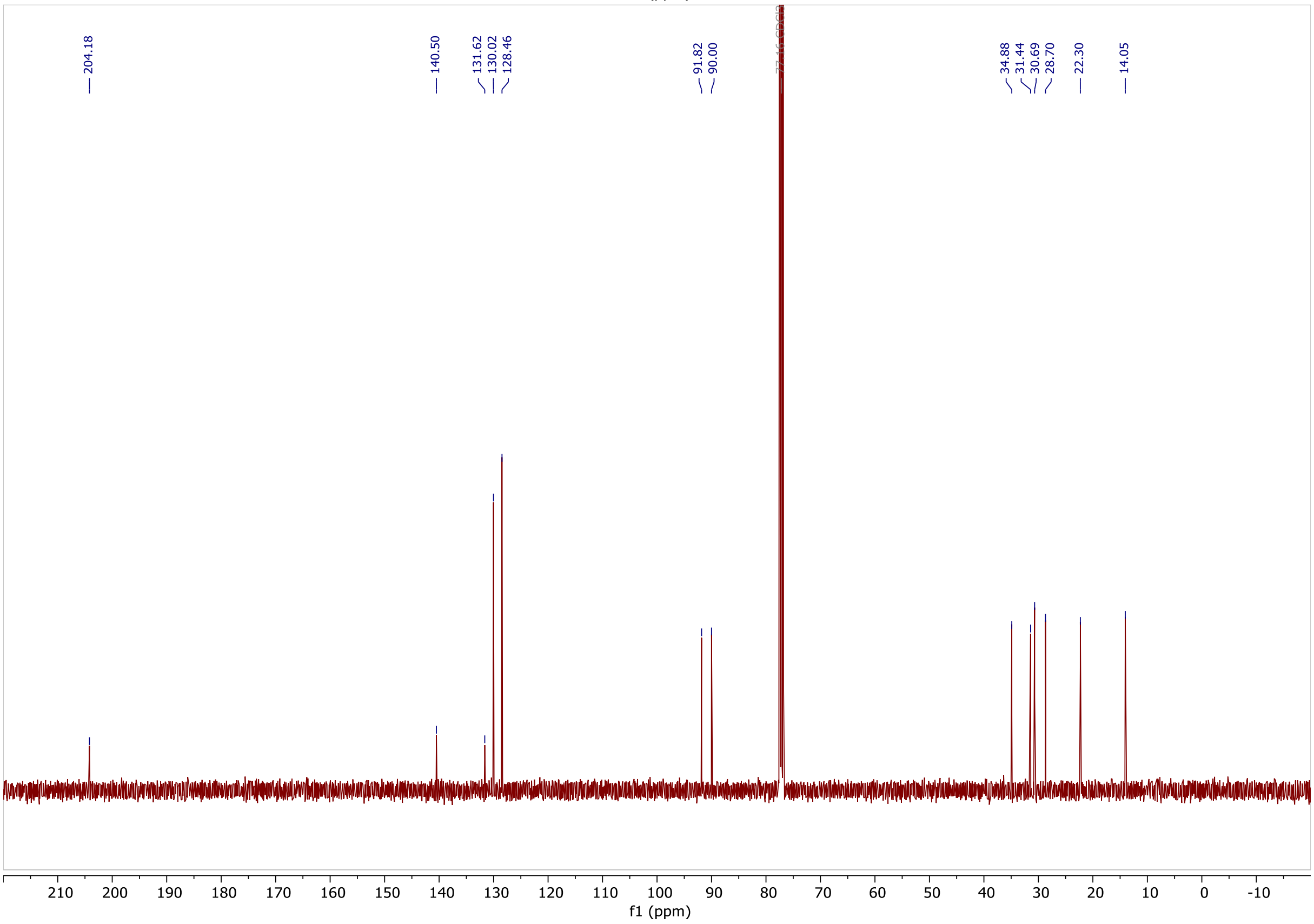

Compound 6

1H, CDCl3, 600 MHz

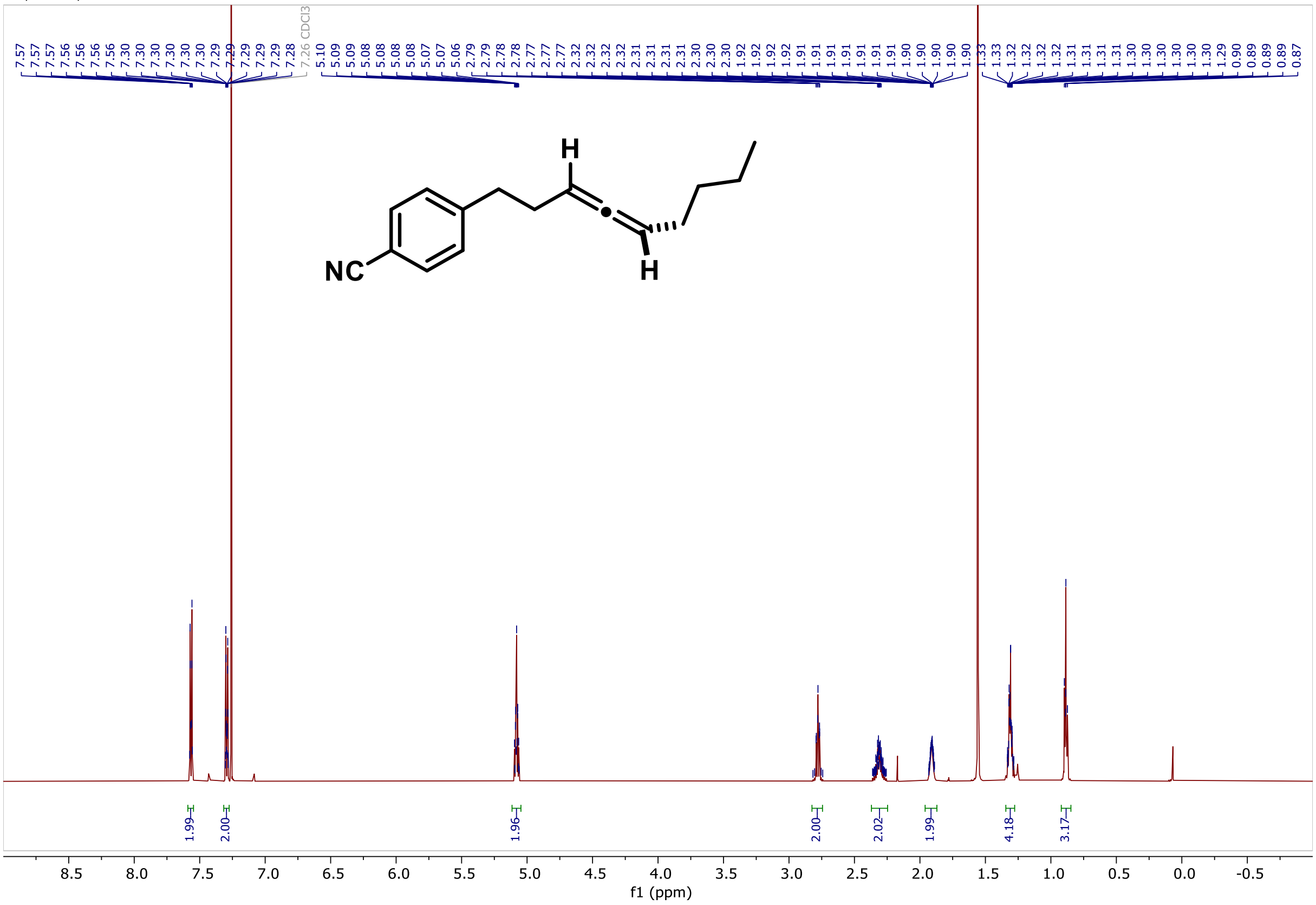

13C, CDCl3, 151 MHz

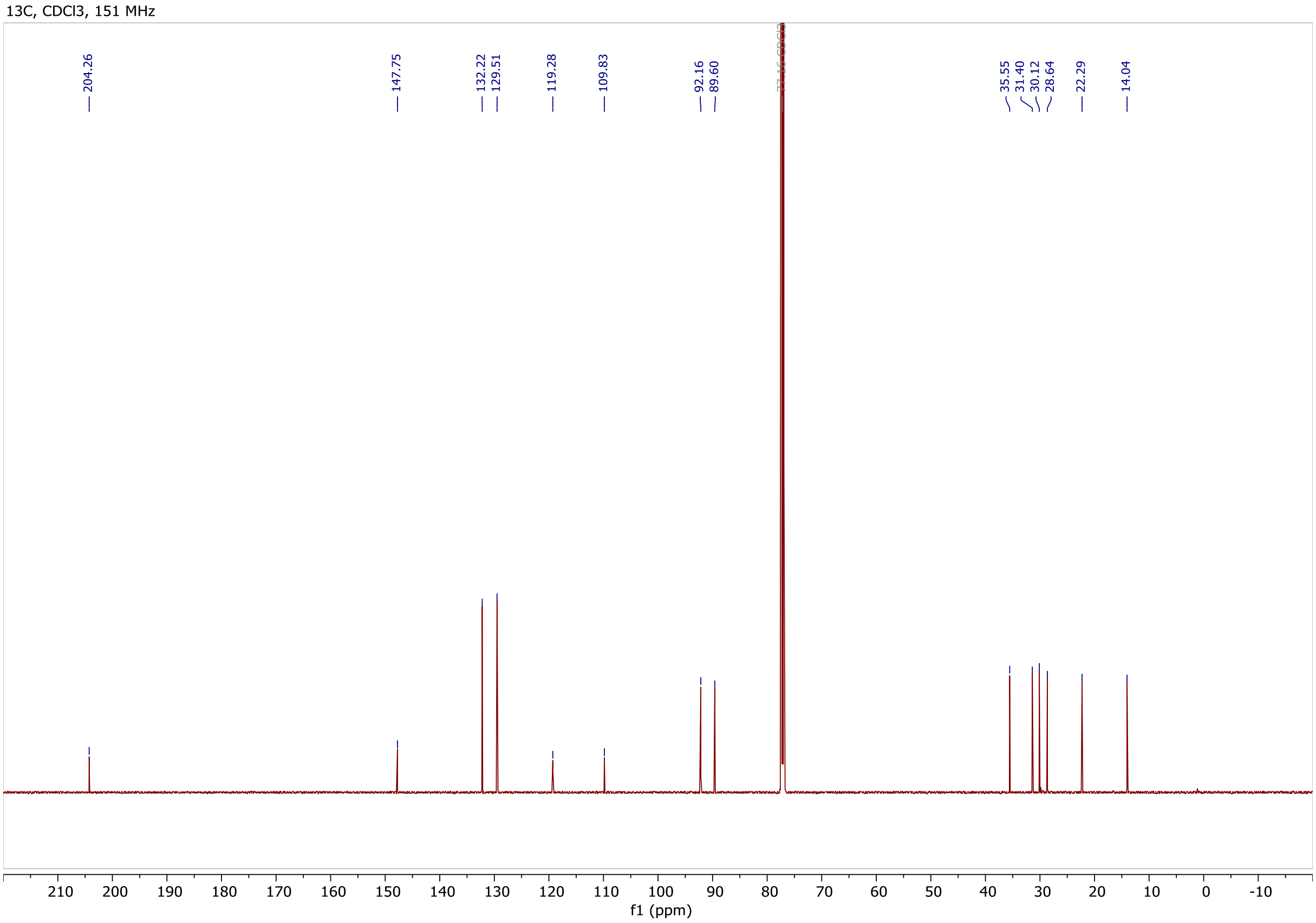

Compound 7 (contaminated by ≈5% protodecupration product)

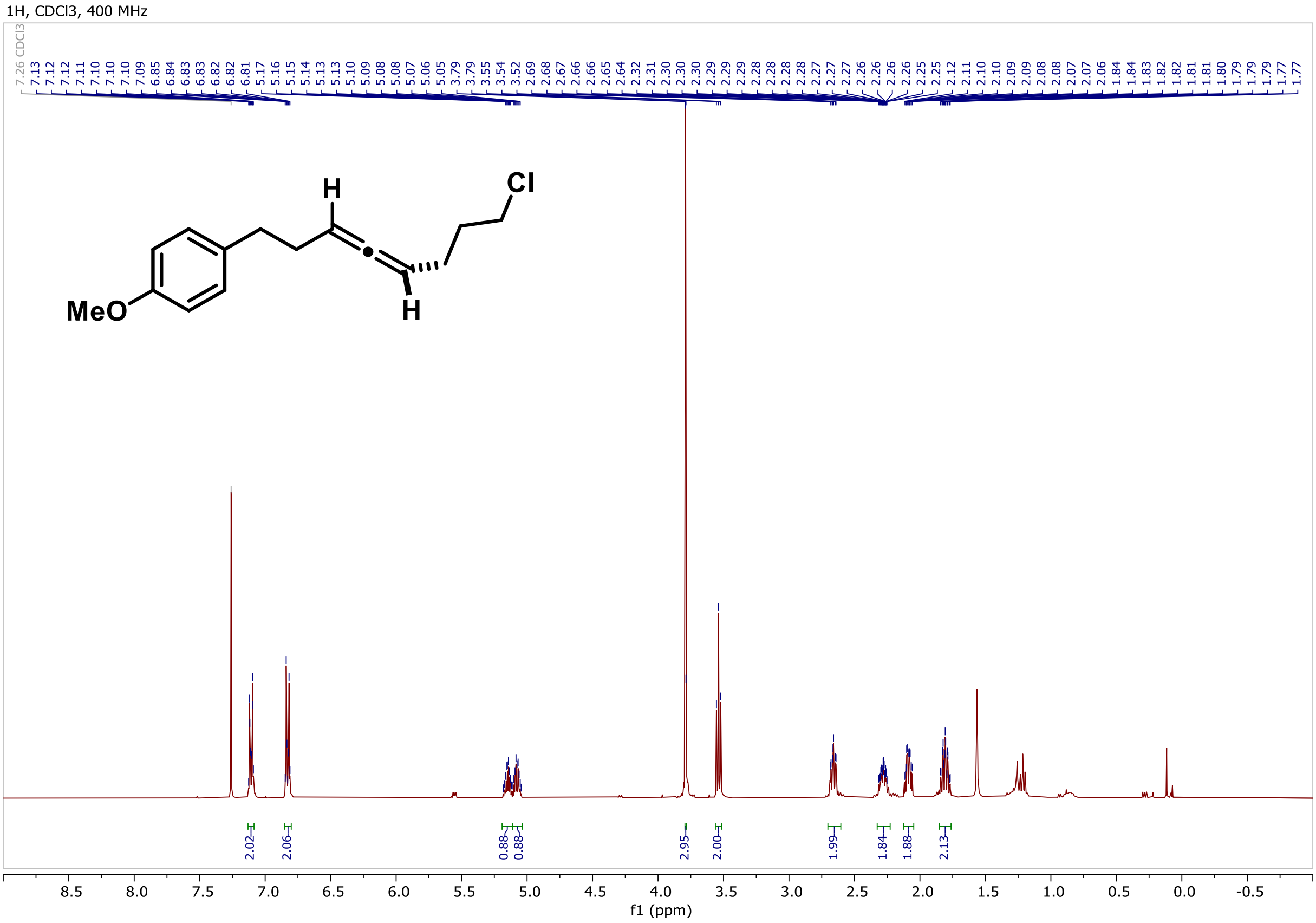

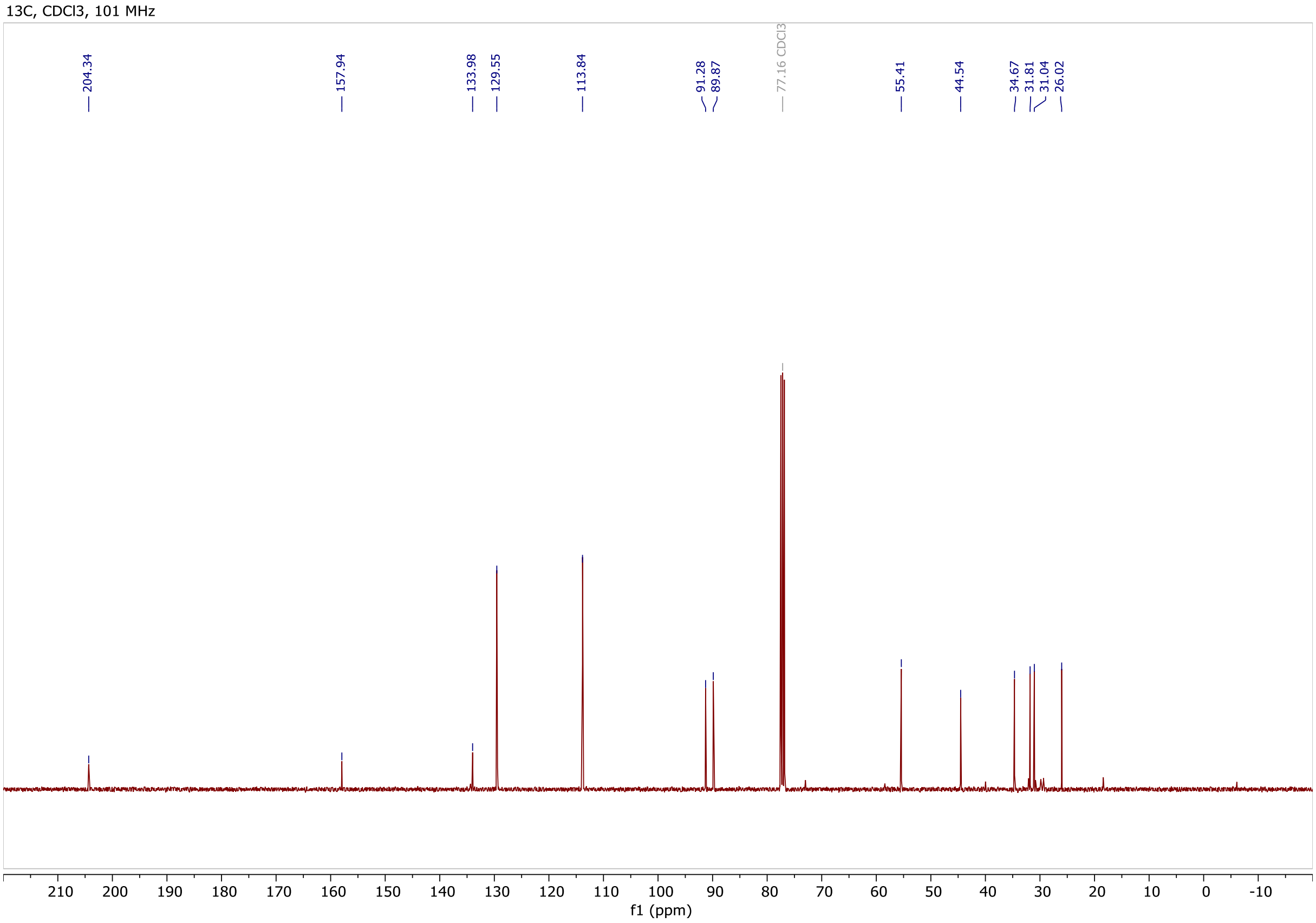

Compound 8

1H, CDCl3, 400 MHz

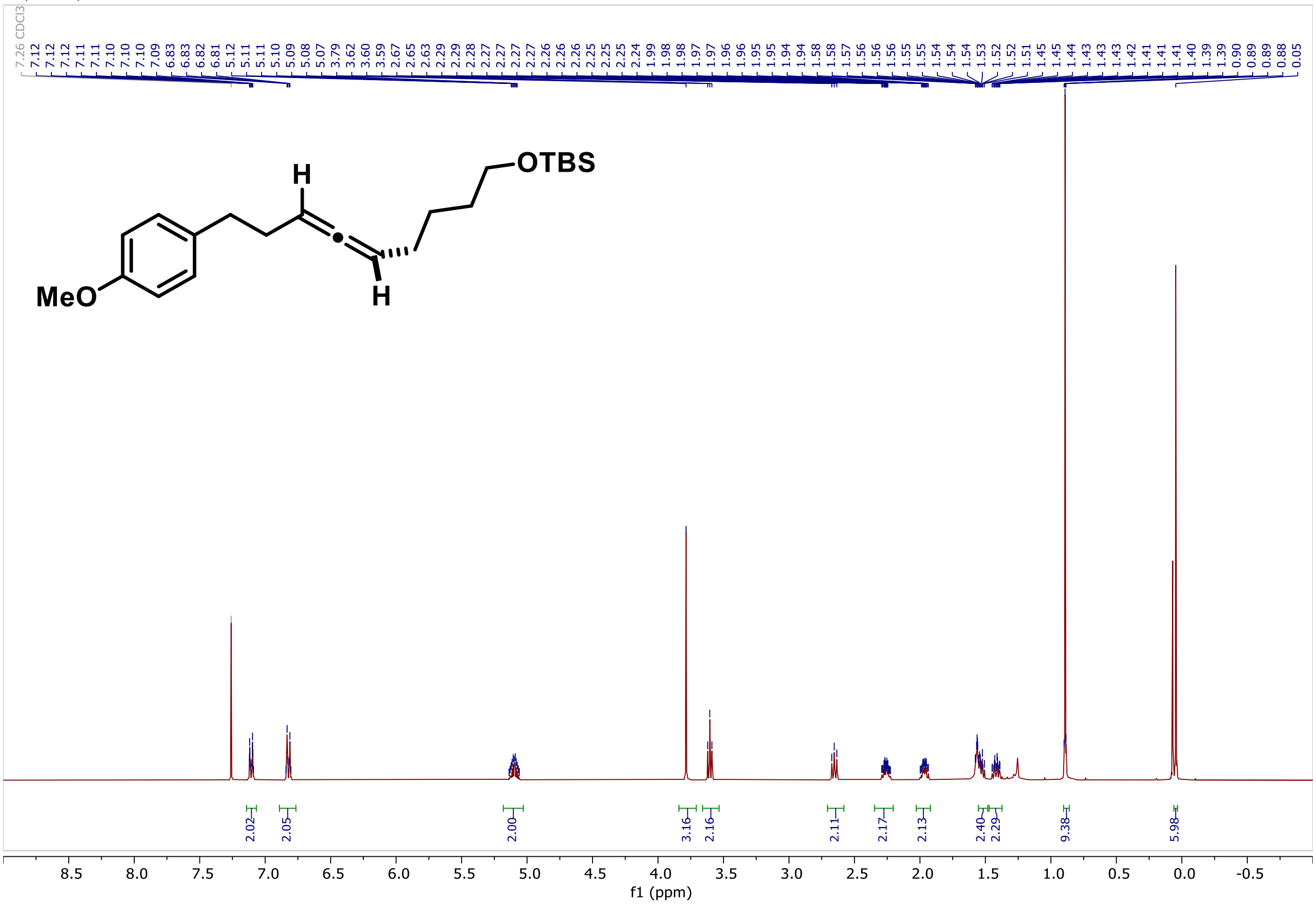

13C, CDCl3, 101 MHz

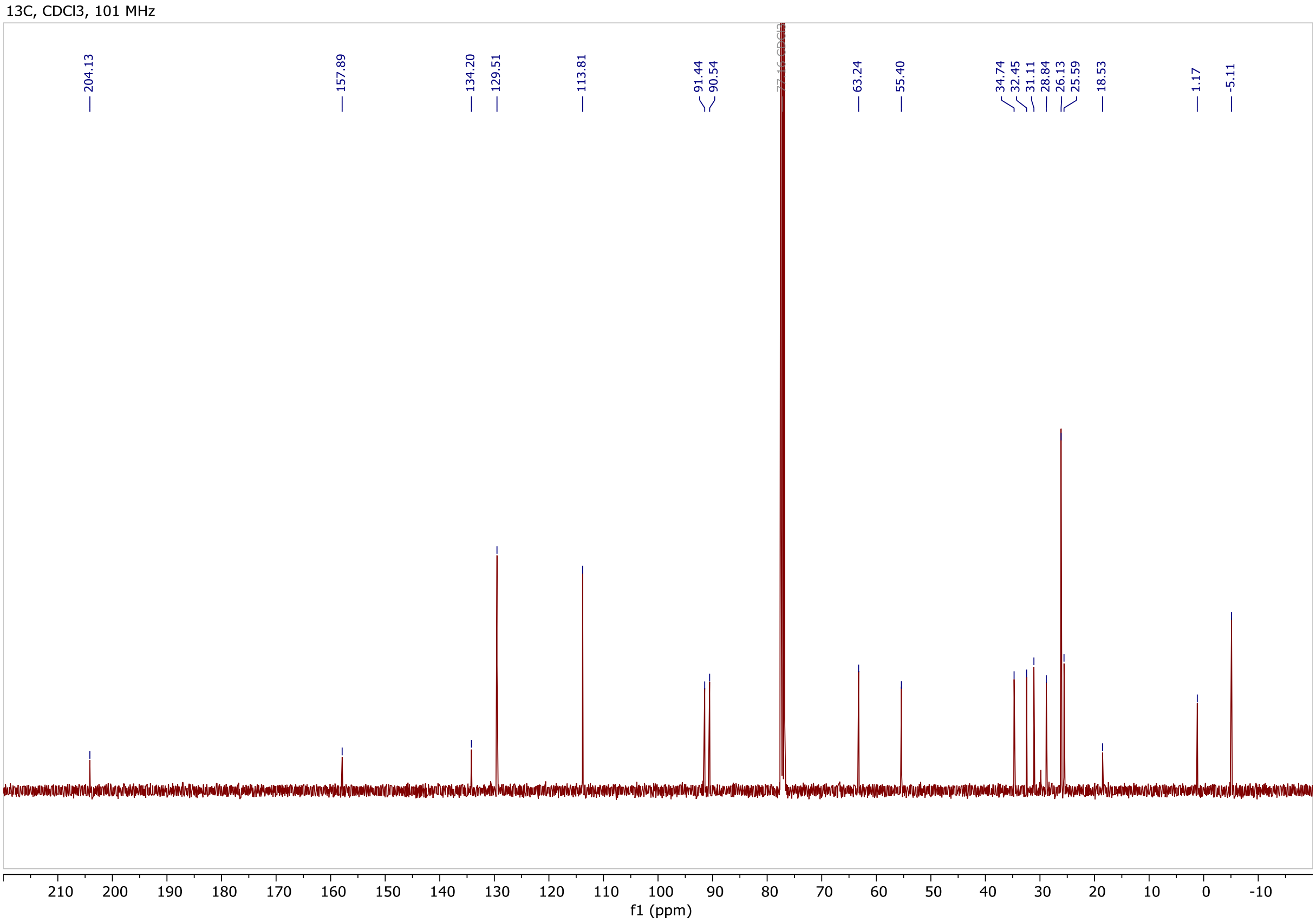

Compound 9

1H, CDCl3, 400 MHz

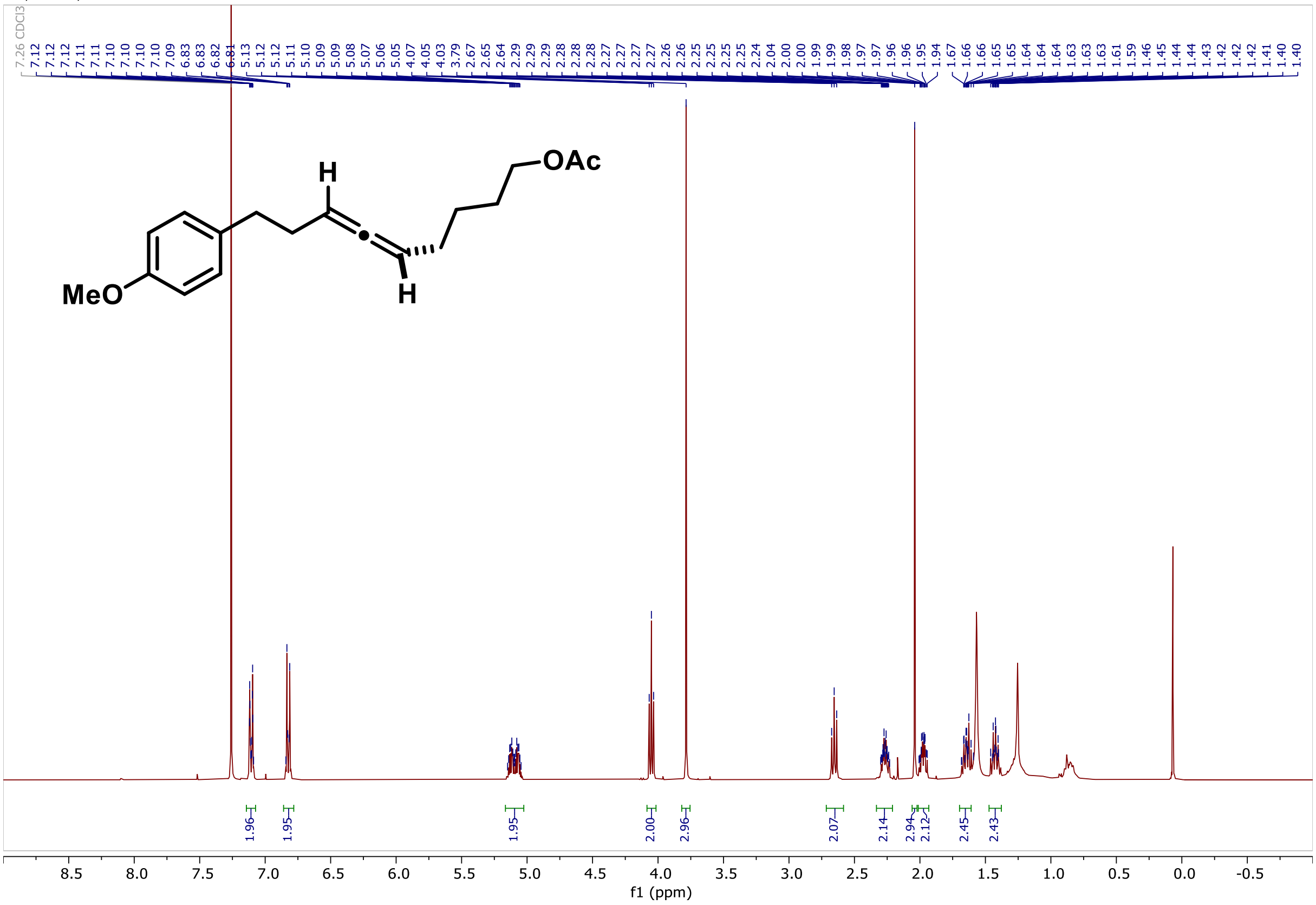

13C, CDCl3, 101 MHz

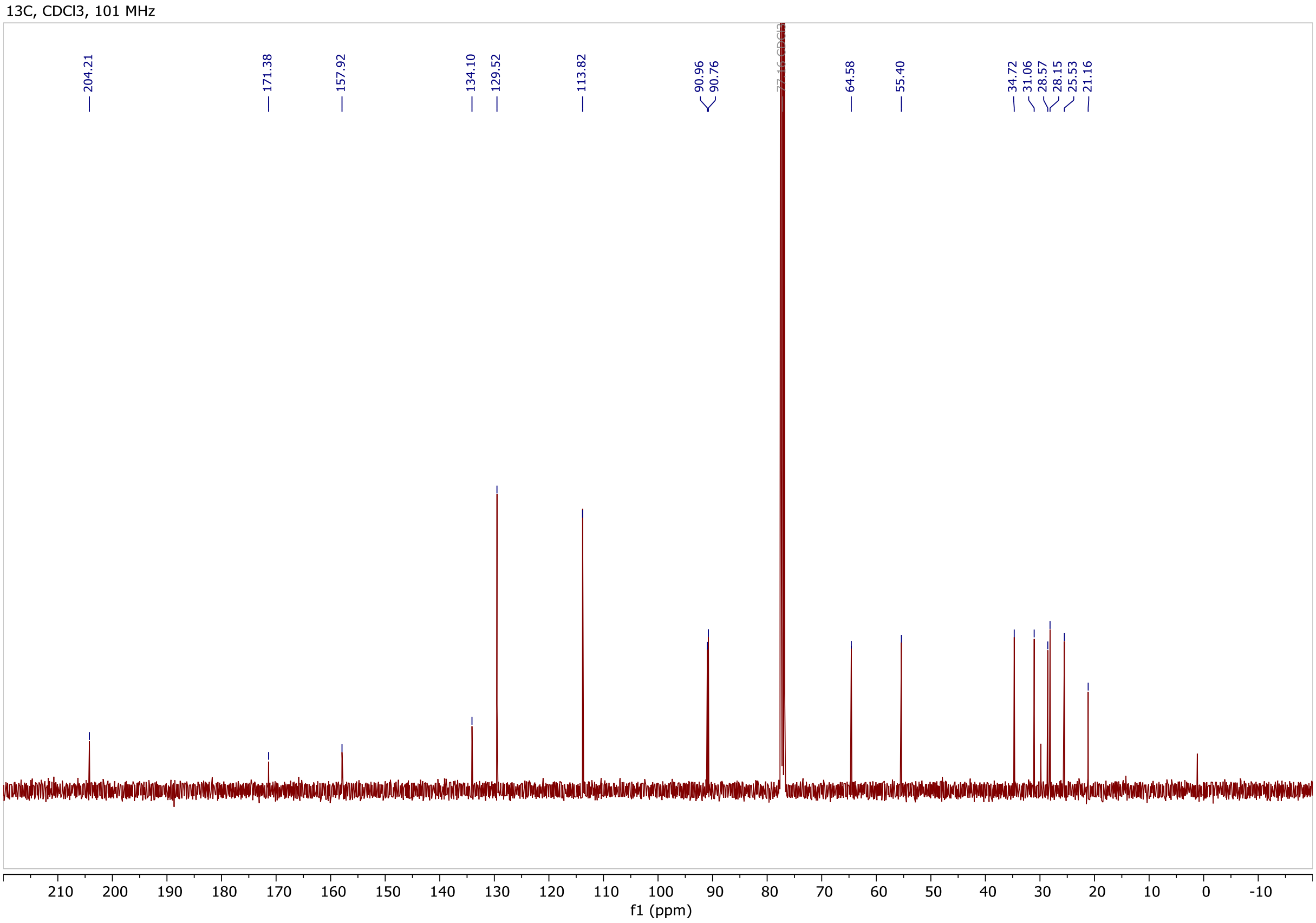

Compound 10

1H, CDCl3, 400 MHz

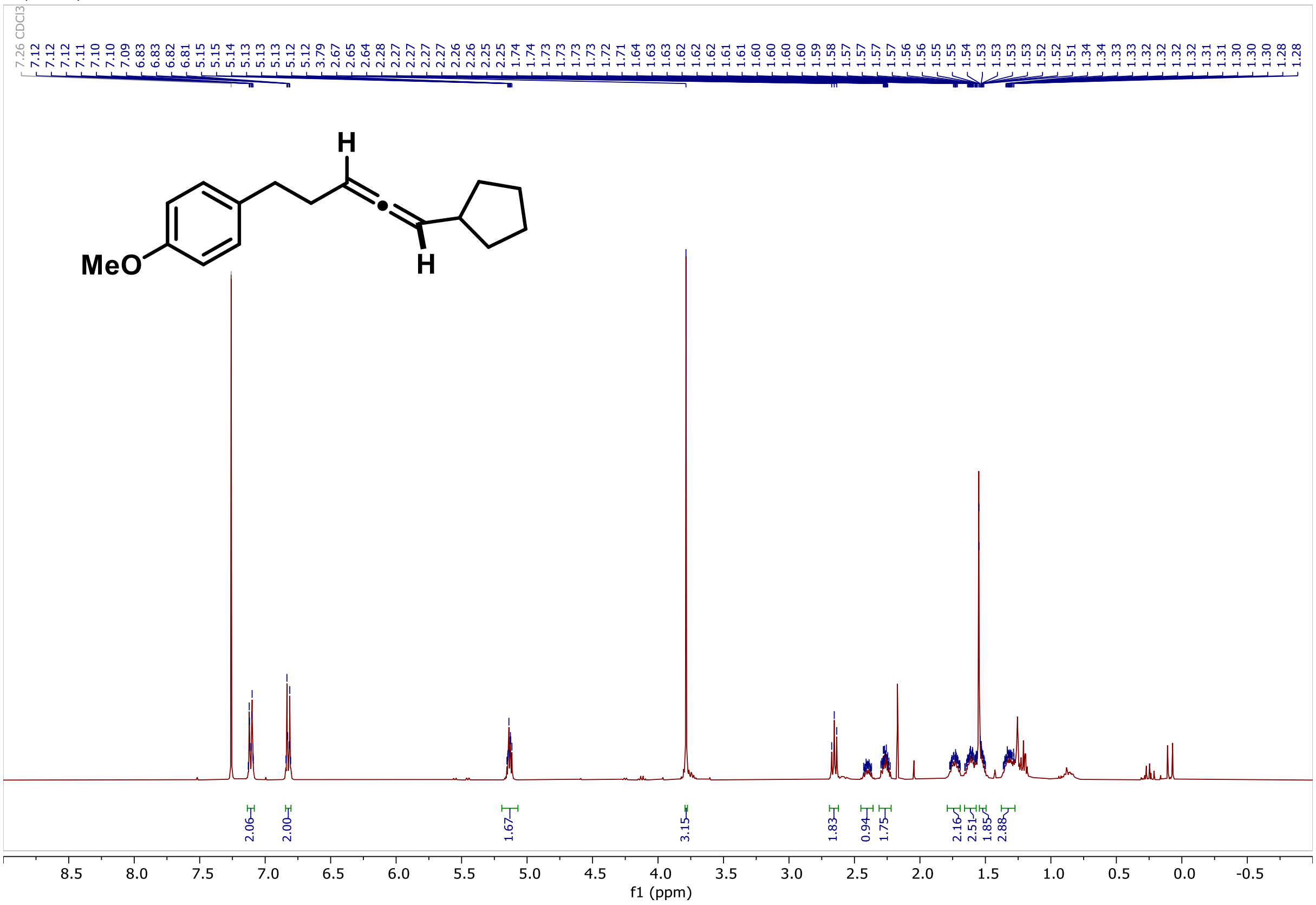

13C, CDCl3, 101 MHz

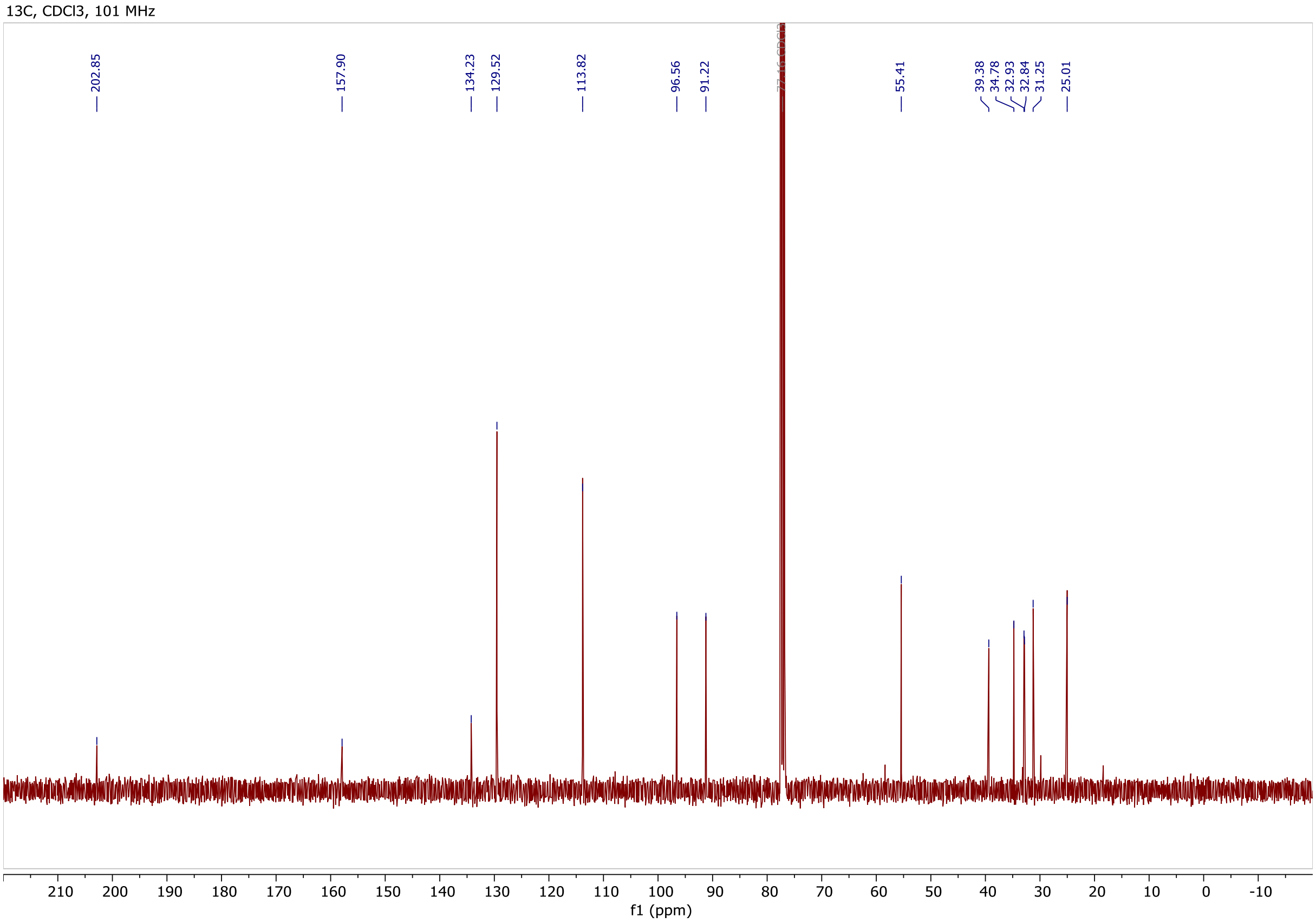

Compound 11a

1H, CDCl3, 400 MHz

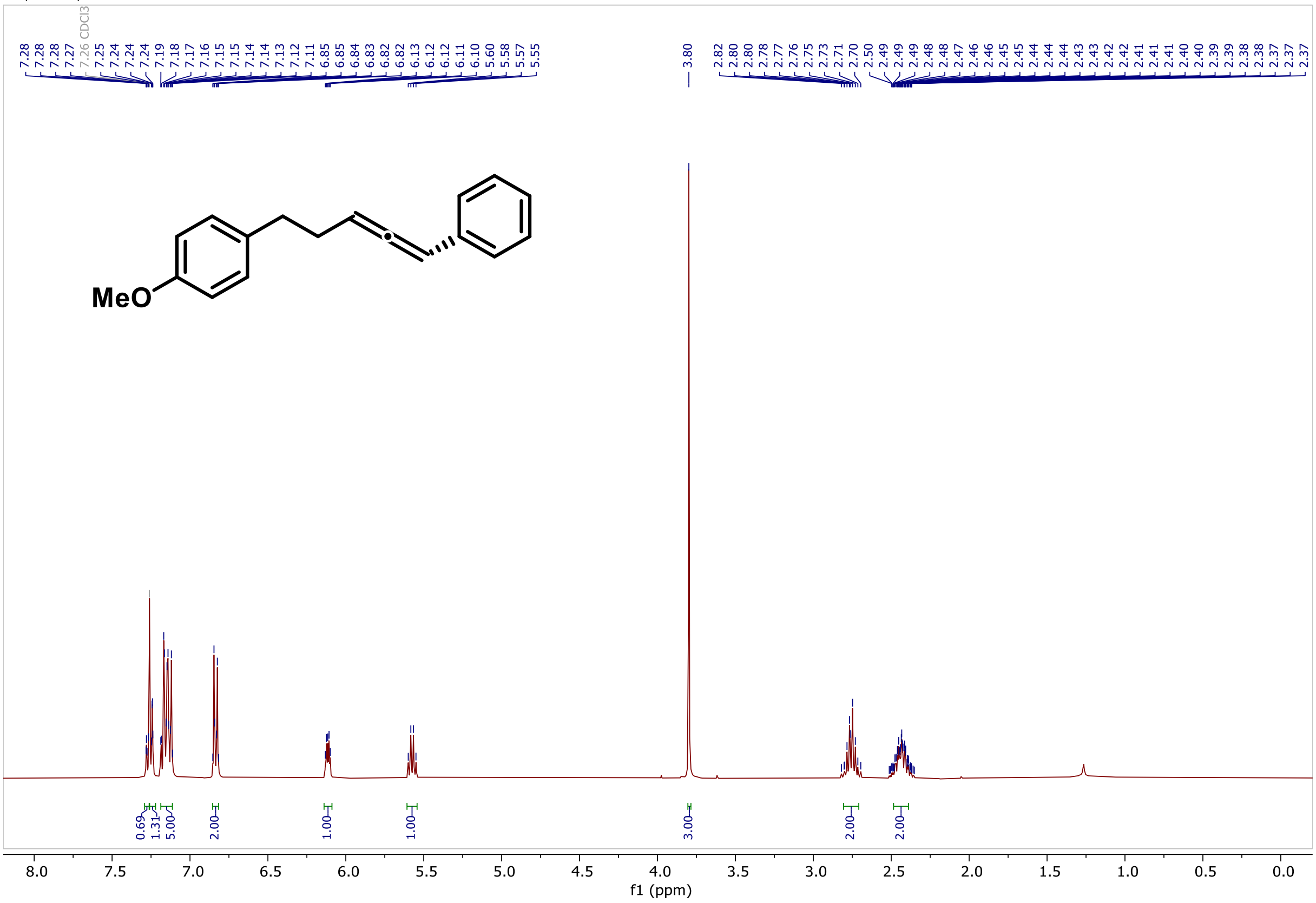

13C, CDCl3, 101 MHz

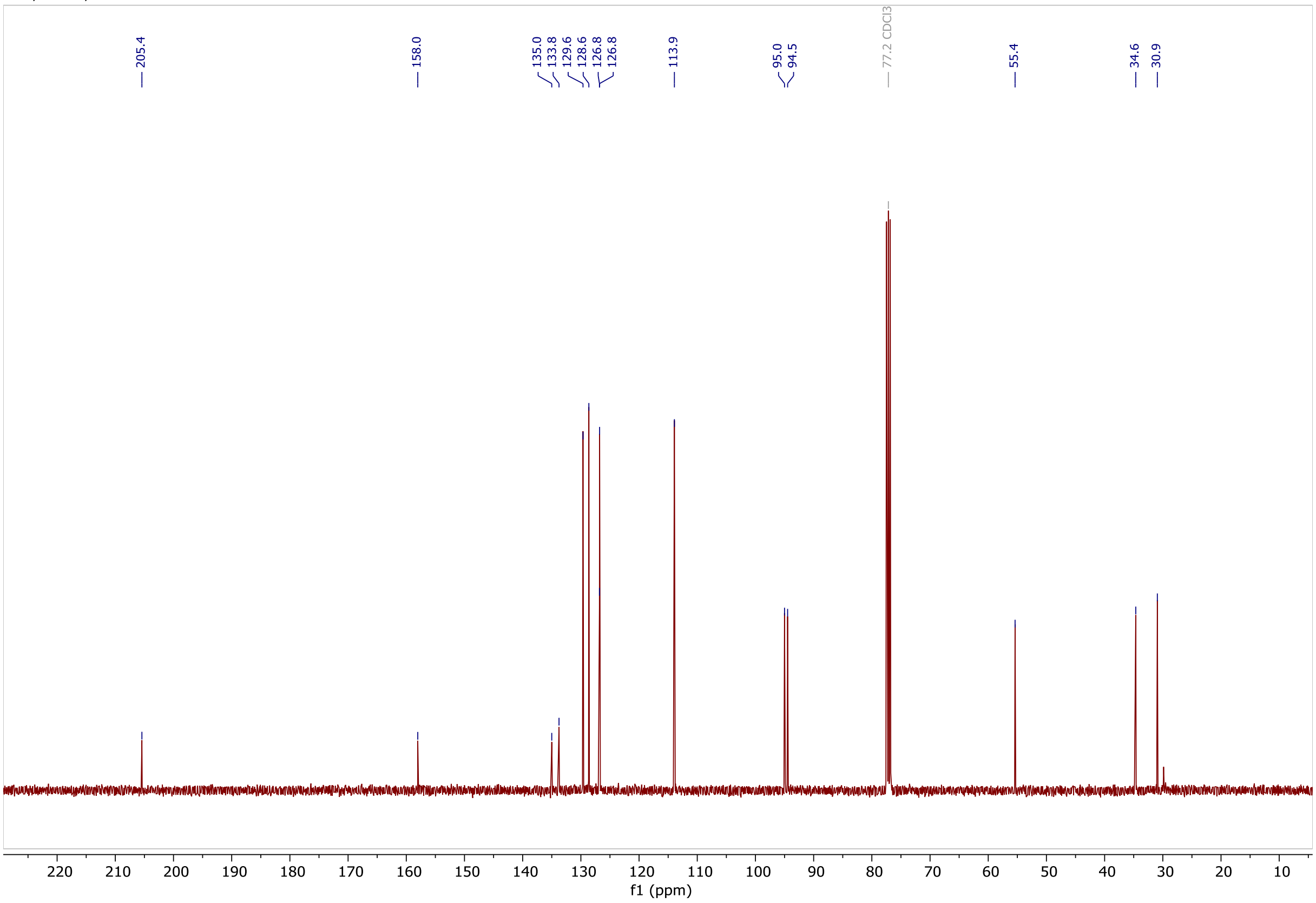

Compound 11b

1H, CDCl3, 400 MHz

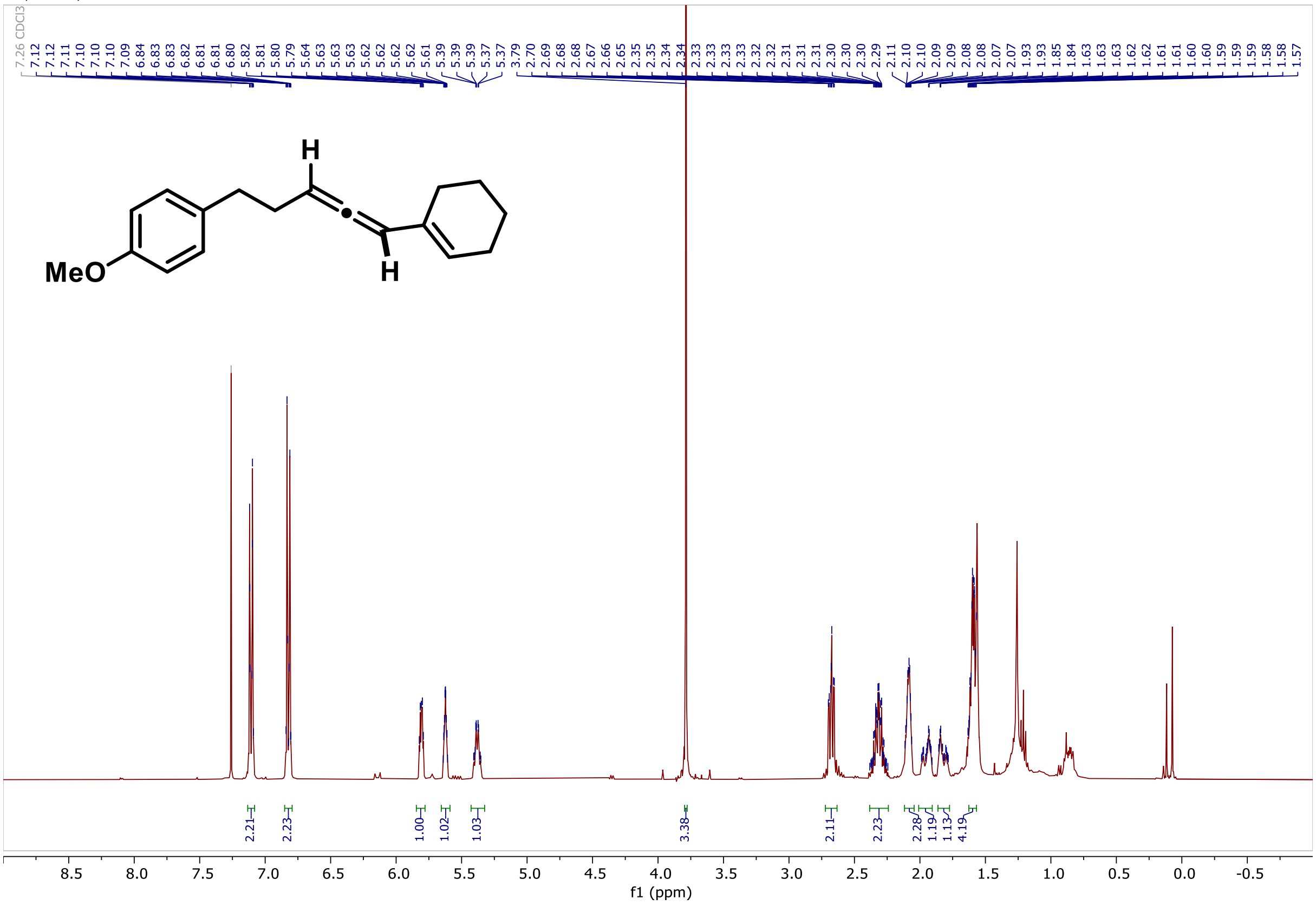

13C, CDCl3, 101 MHz

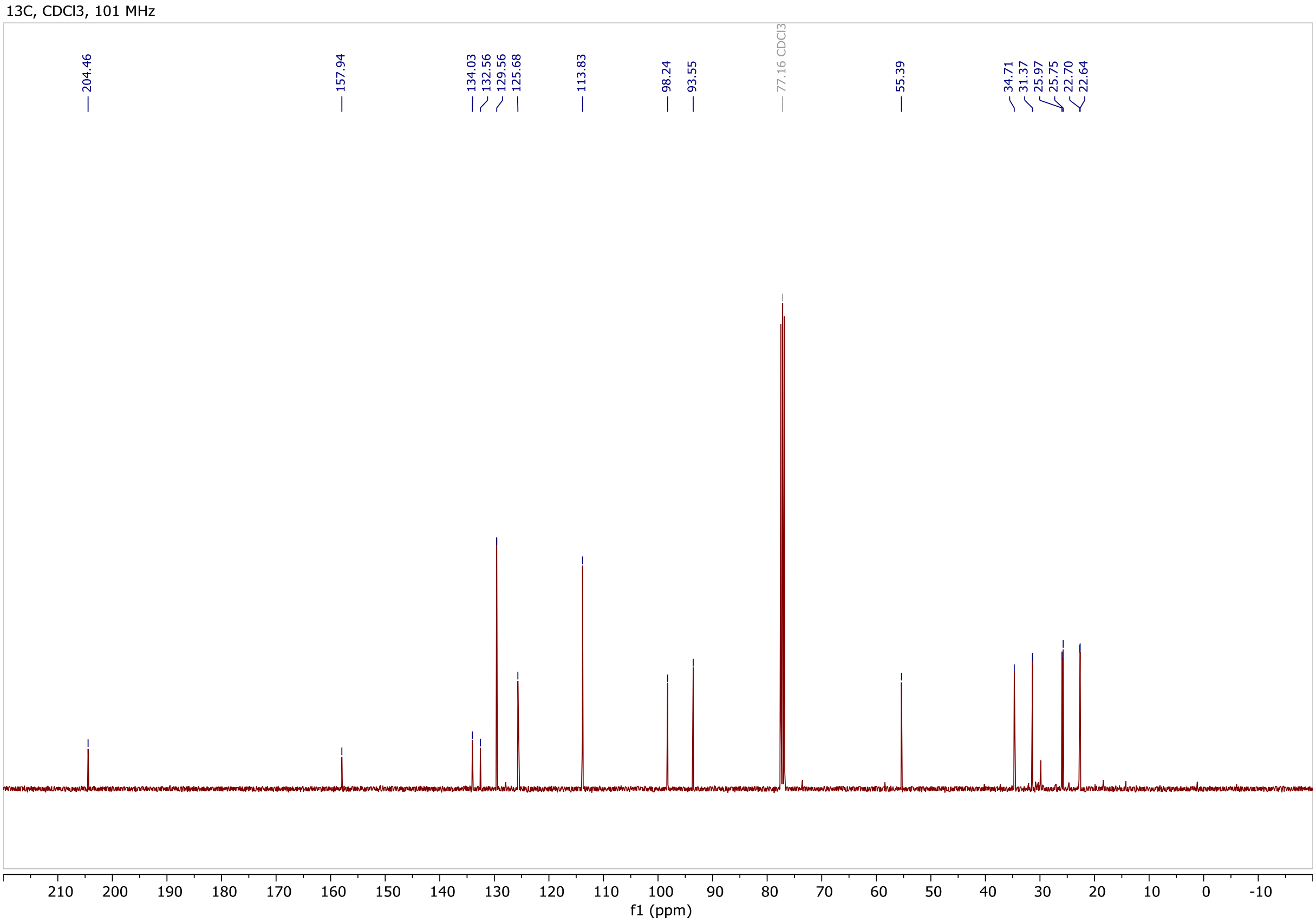

Compound 12

1H, CDCl3, 400 MHz

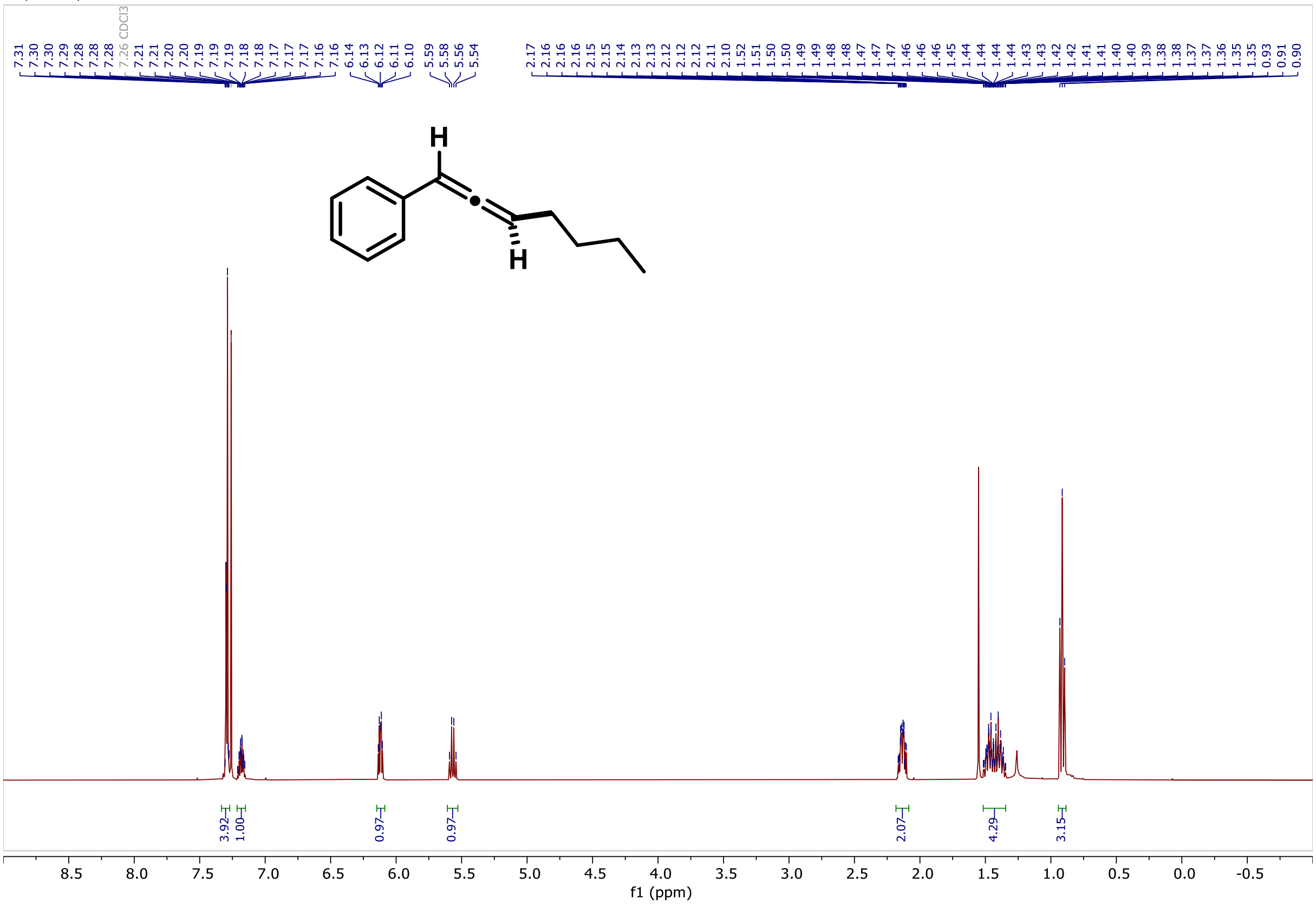

13C, CDCl3, 101 MHz

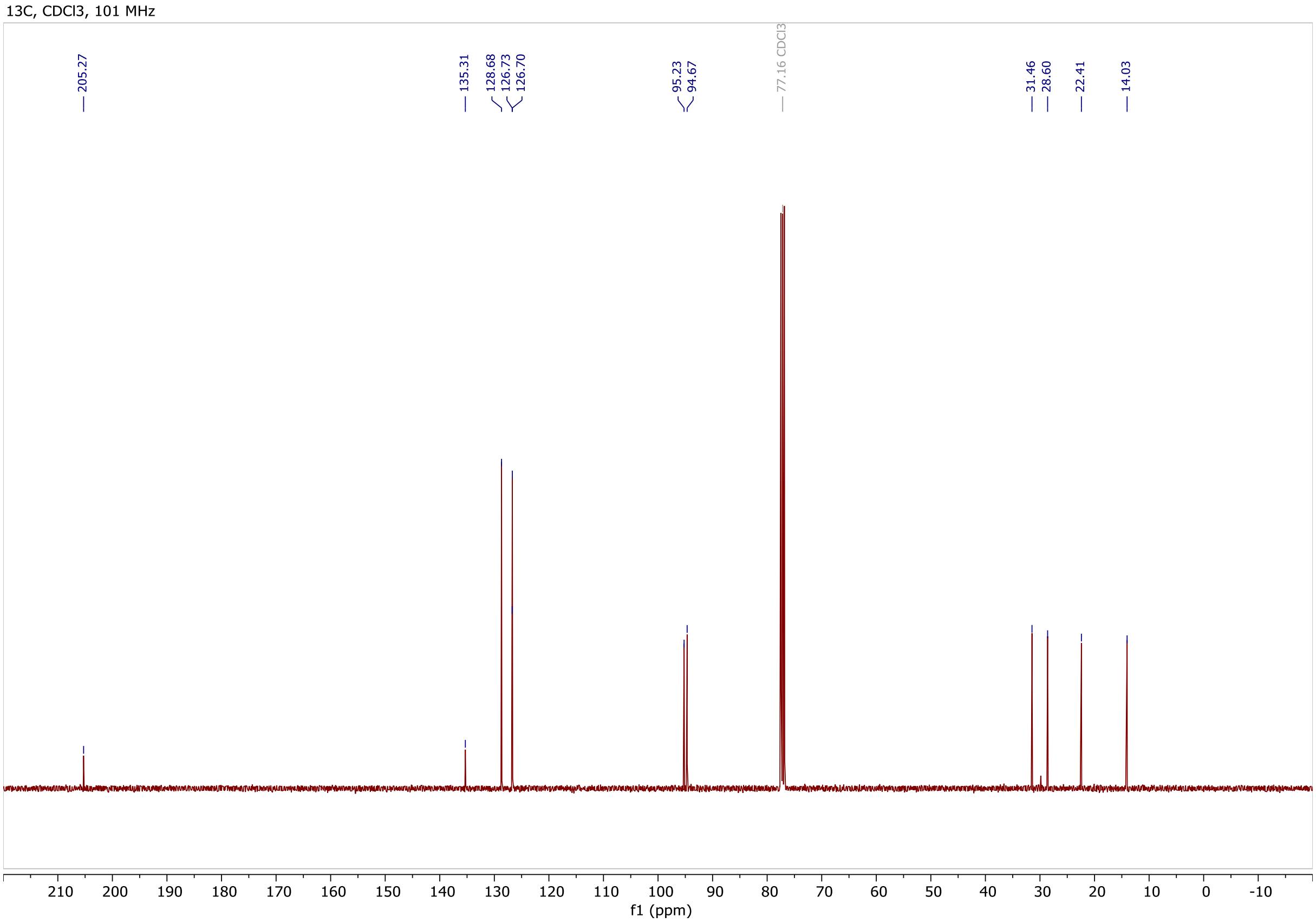

Compound 13

1H, CDCl3, 400 MHz

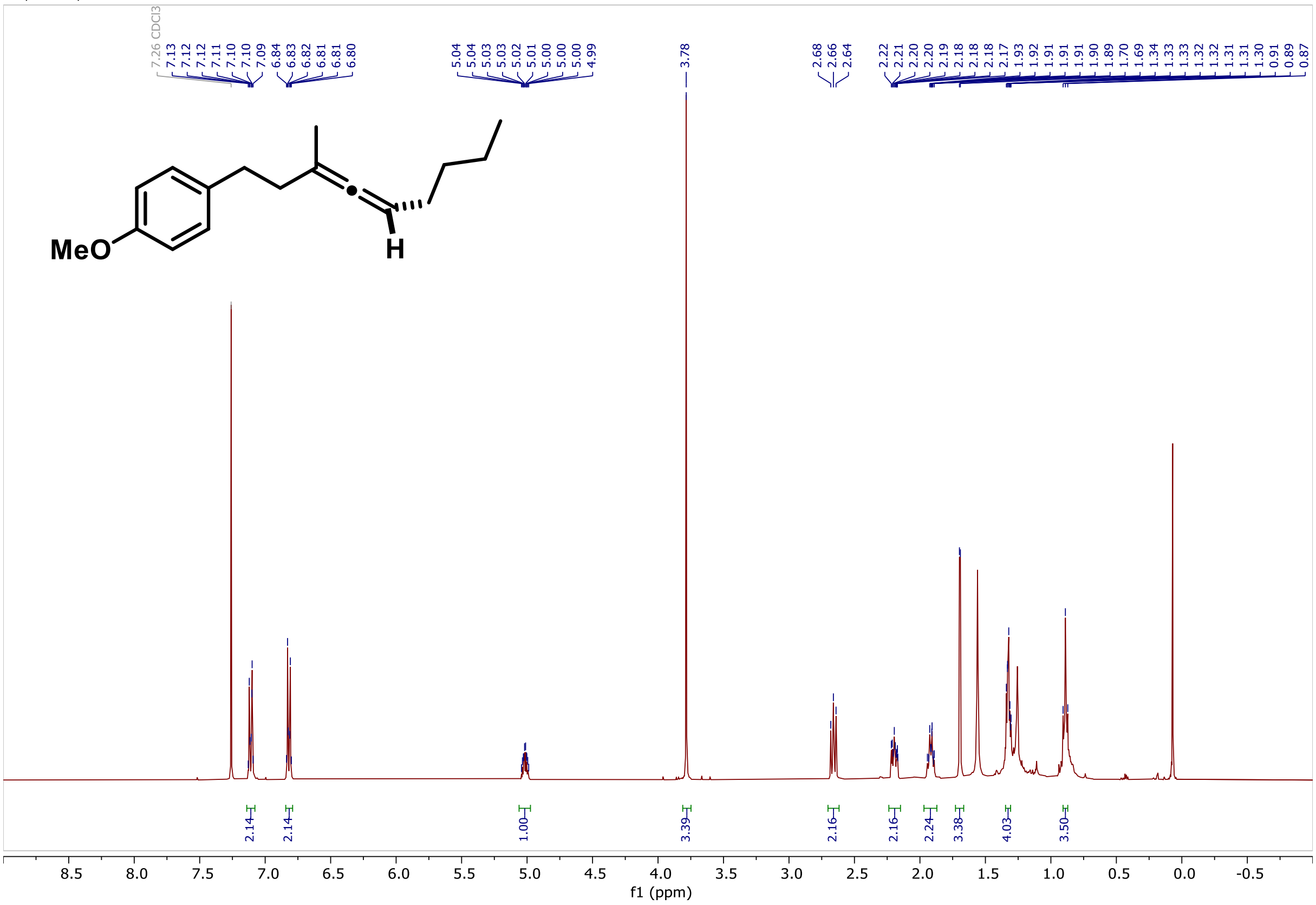

13C, CDCl3, 101 MHz

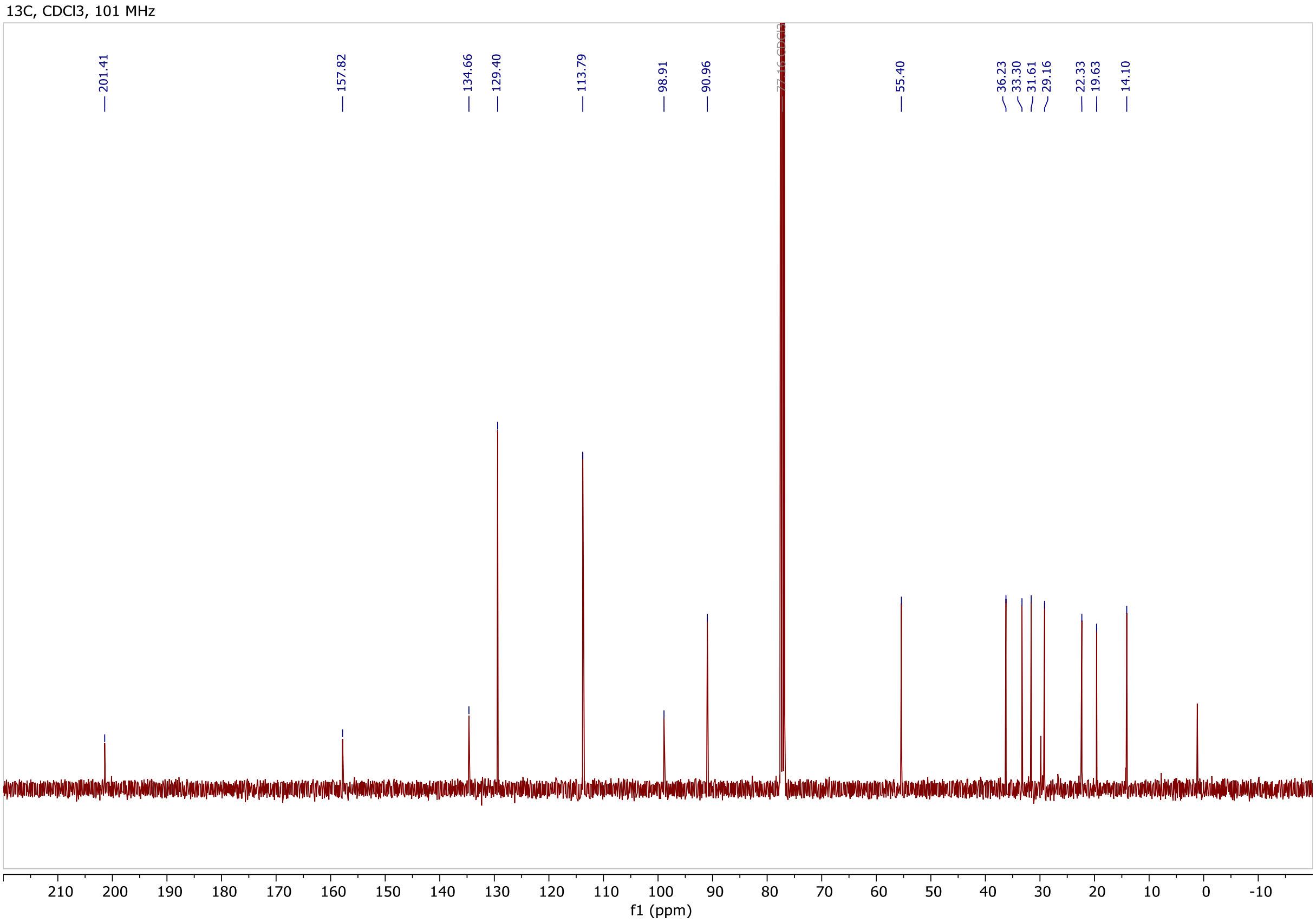

Compound 14

1H, CDCl3, 400 MHz

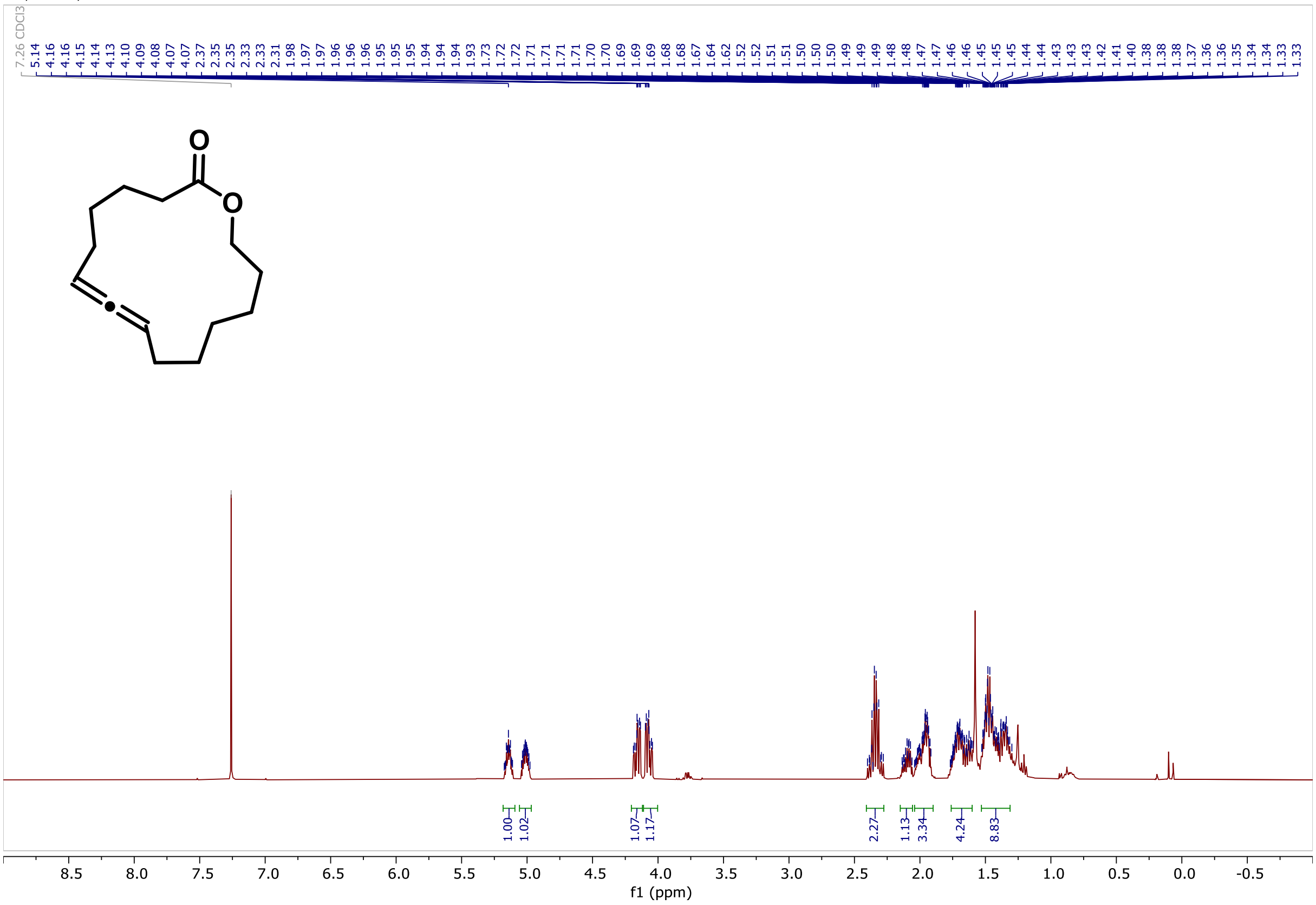

13C, CDCl3, 101 MHz

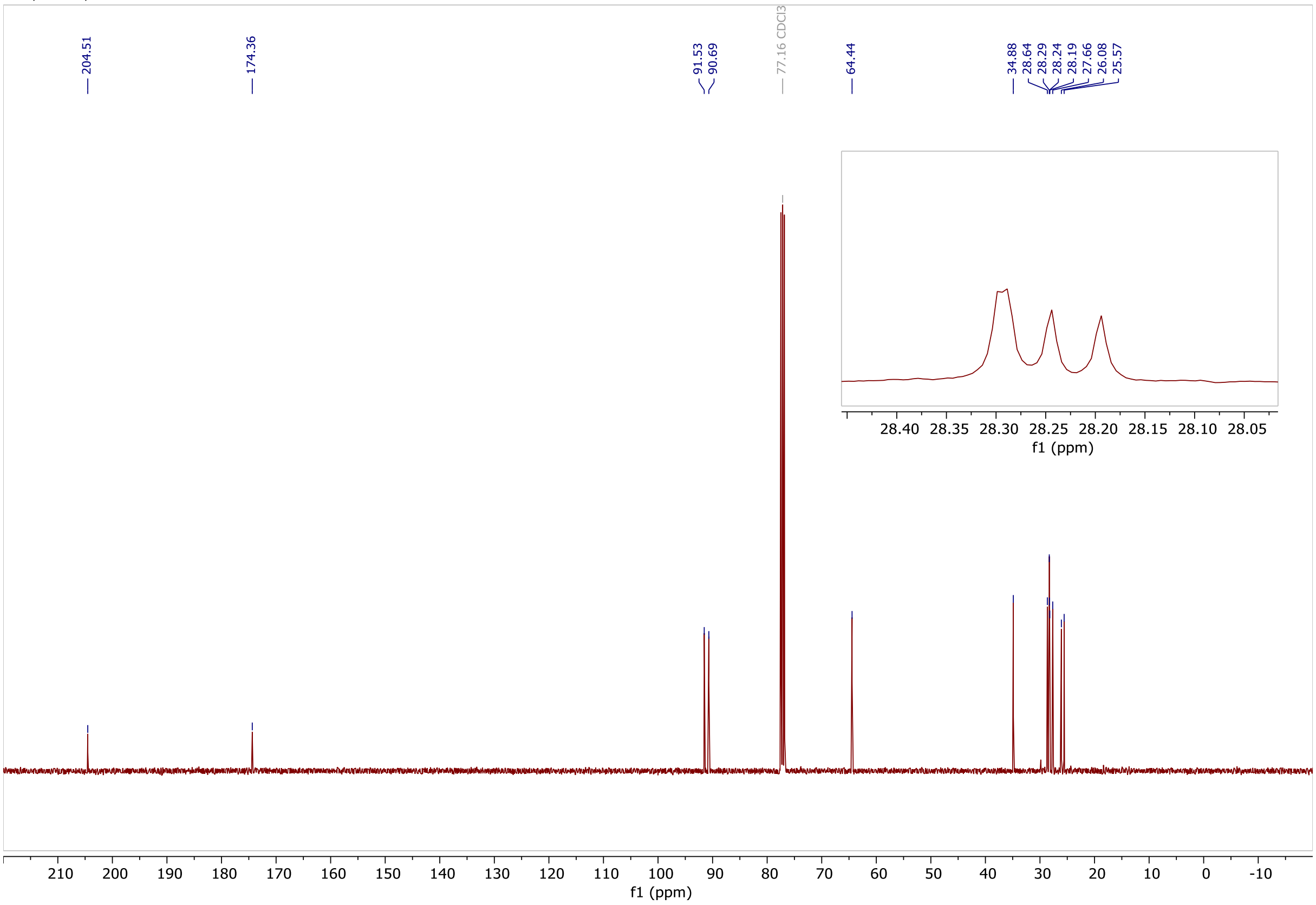

Compound 15

1H, CDCl3, 400 MHz

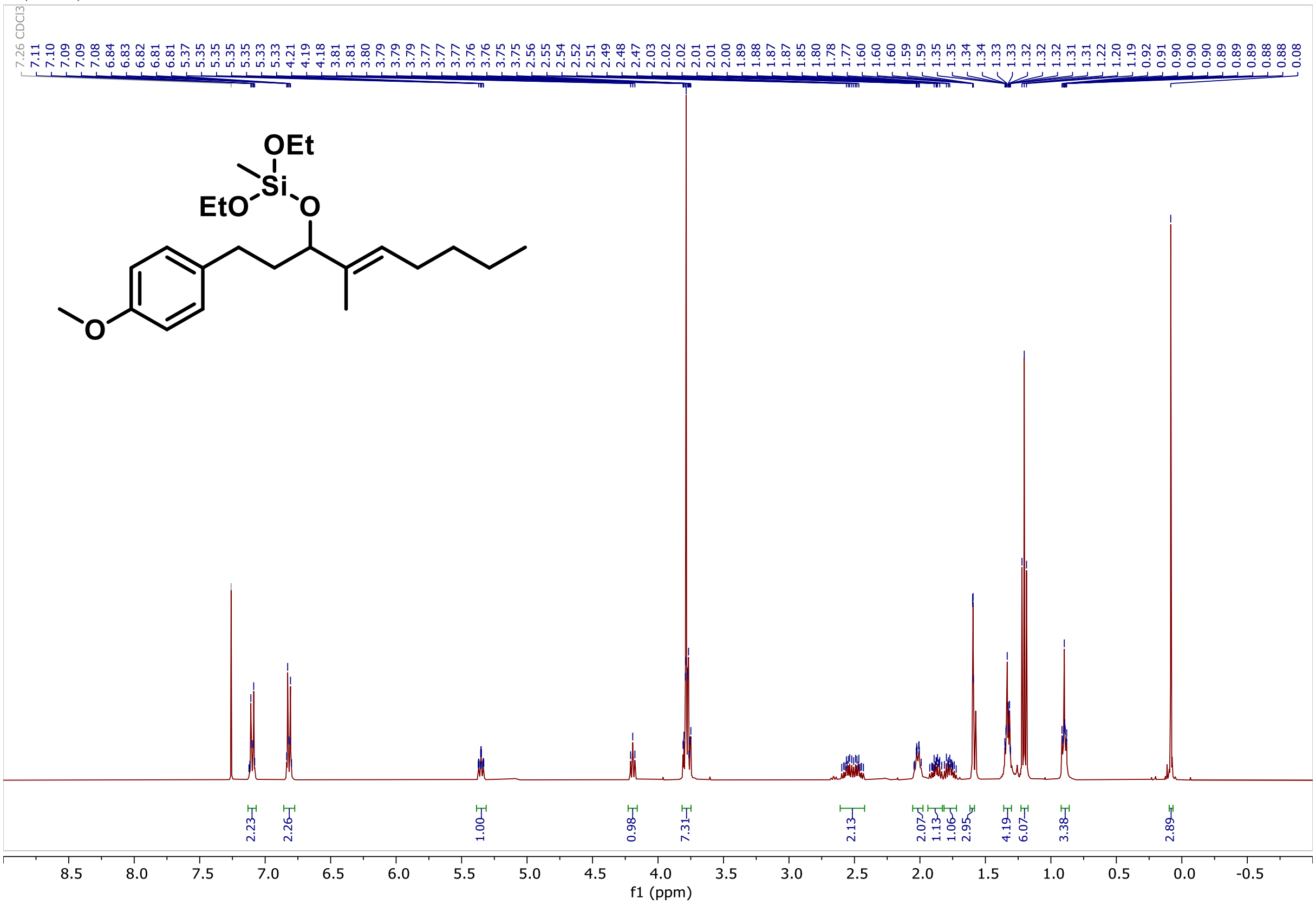

13C, CDCl3, 101 MHz

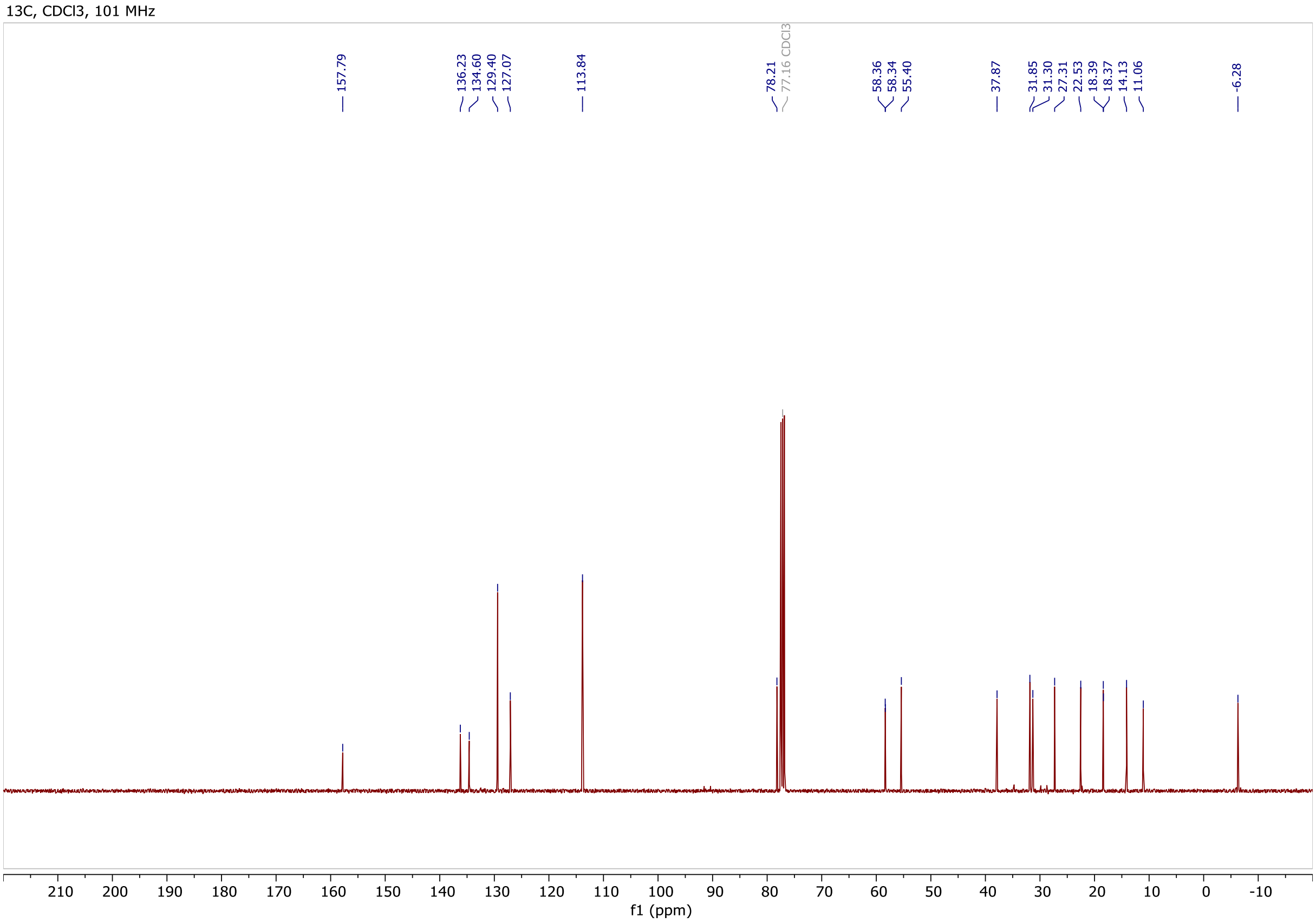

Compound 16

1H, CDCl3, 400 MHz

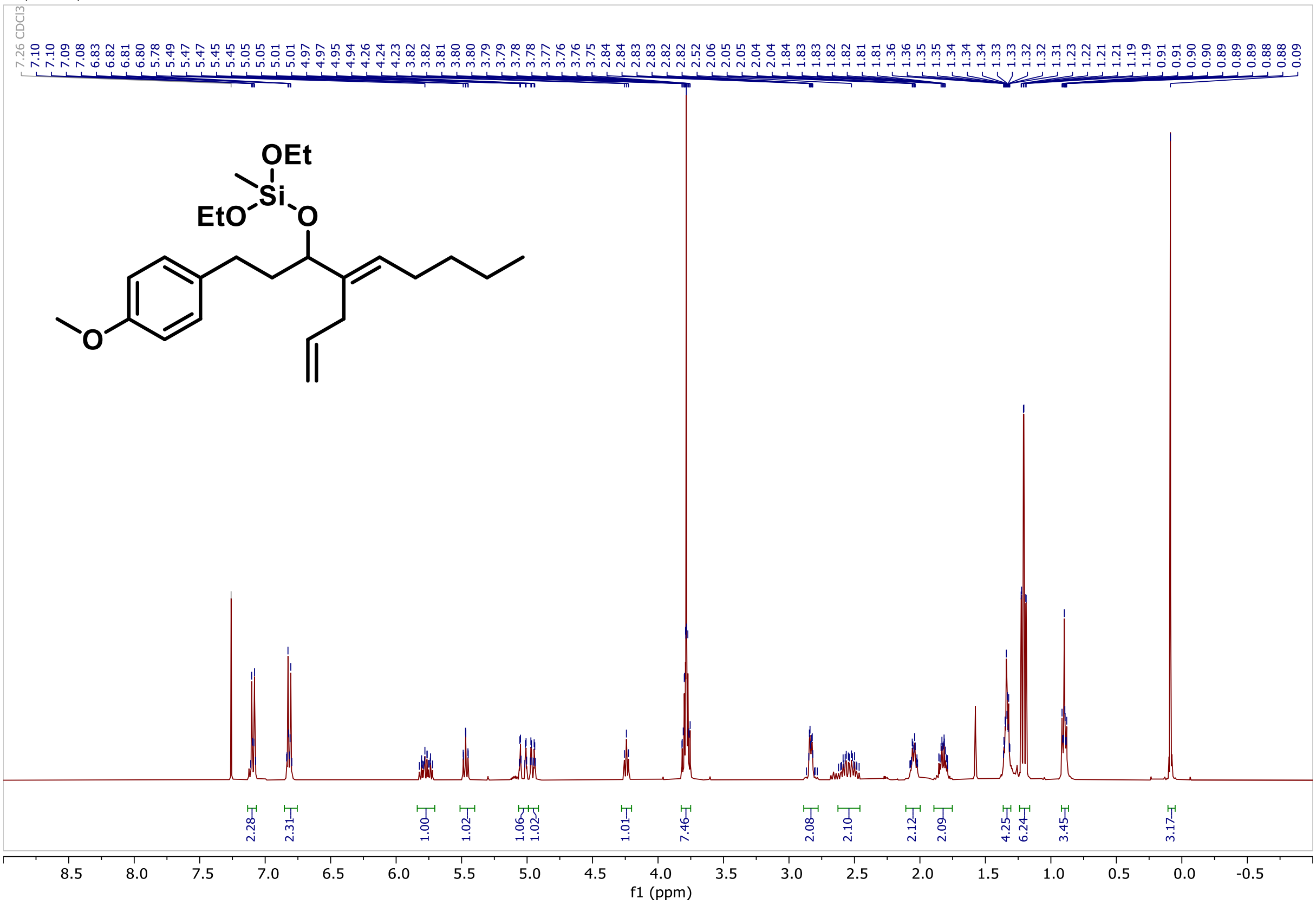

13C, CDCl3, 101 MHz

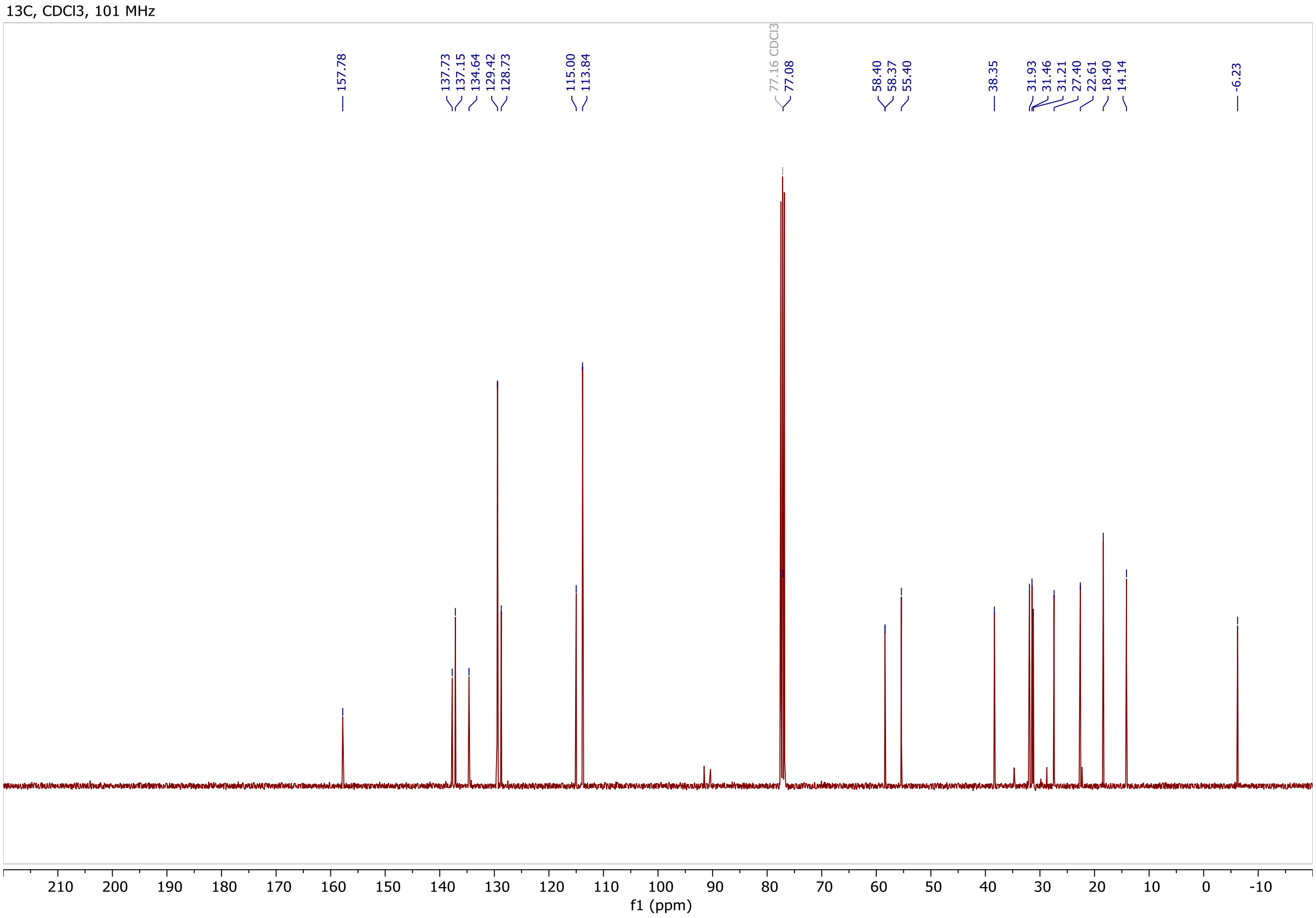

Compound 17

1H, CDCl3, 400 MHz

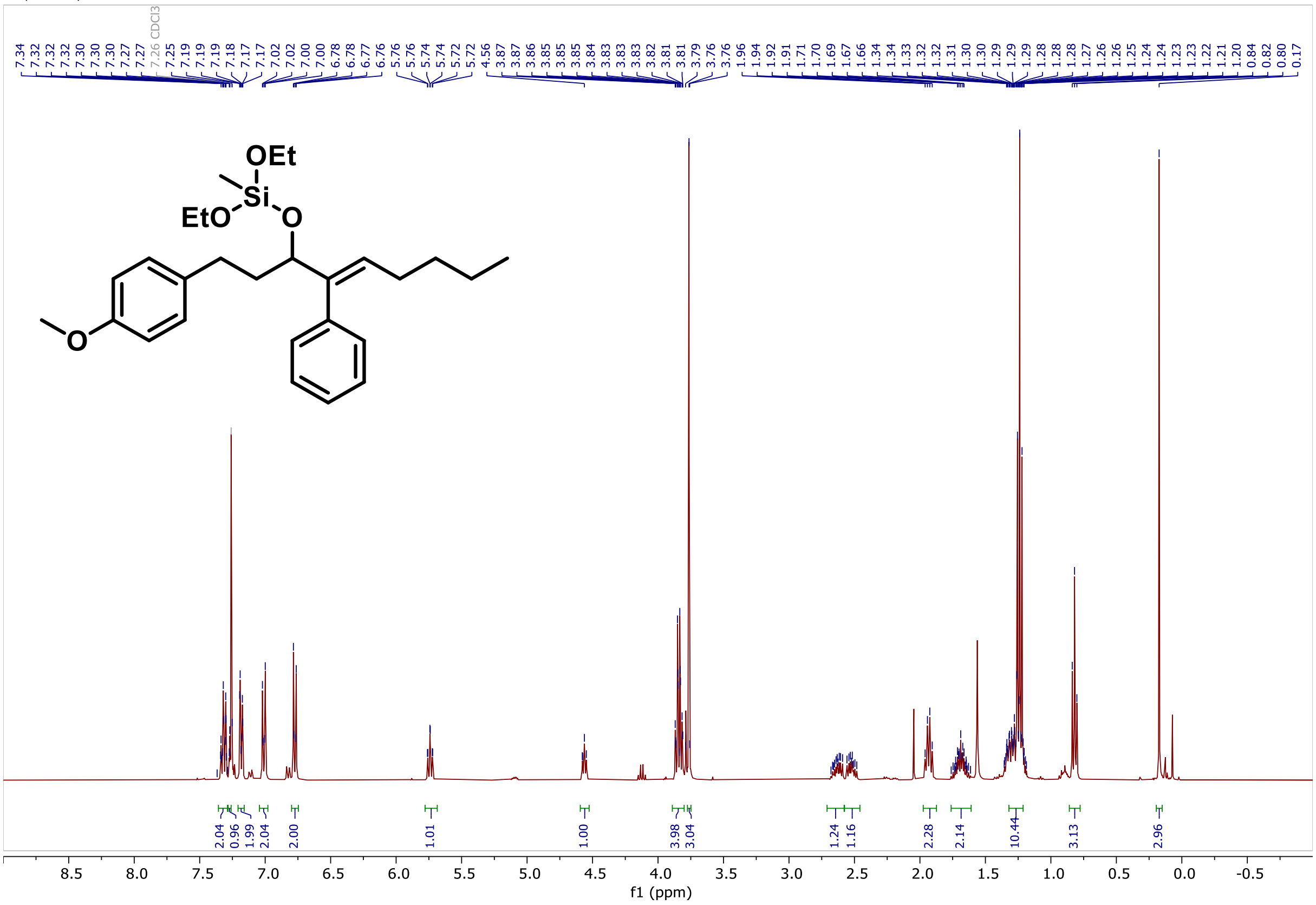

13C, CDCl3, 101 MHz

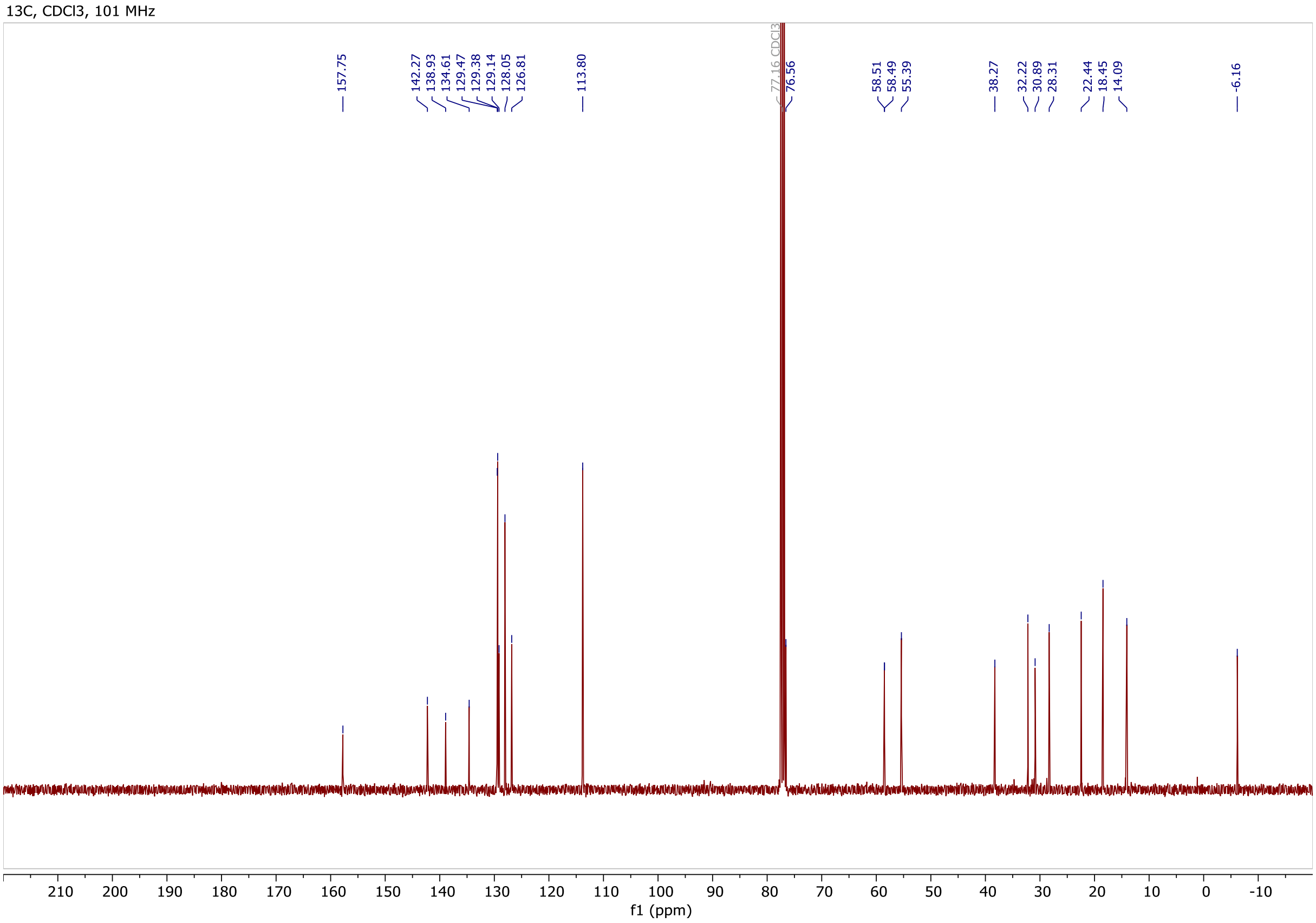

Compound 18

1H, CDCl3, 400 MHz

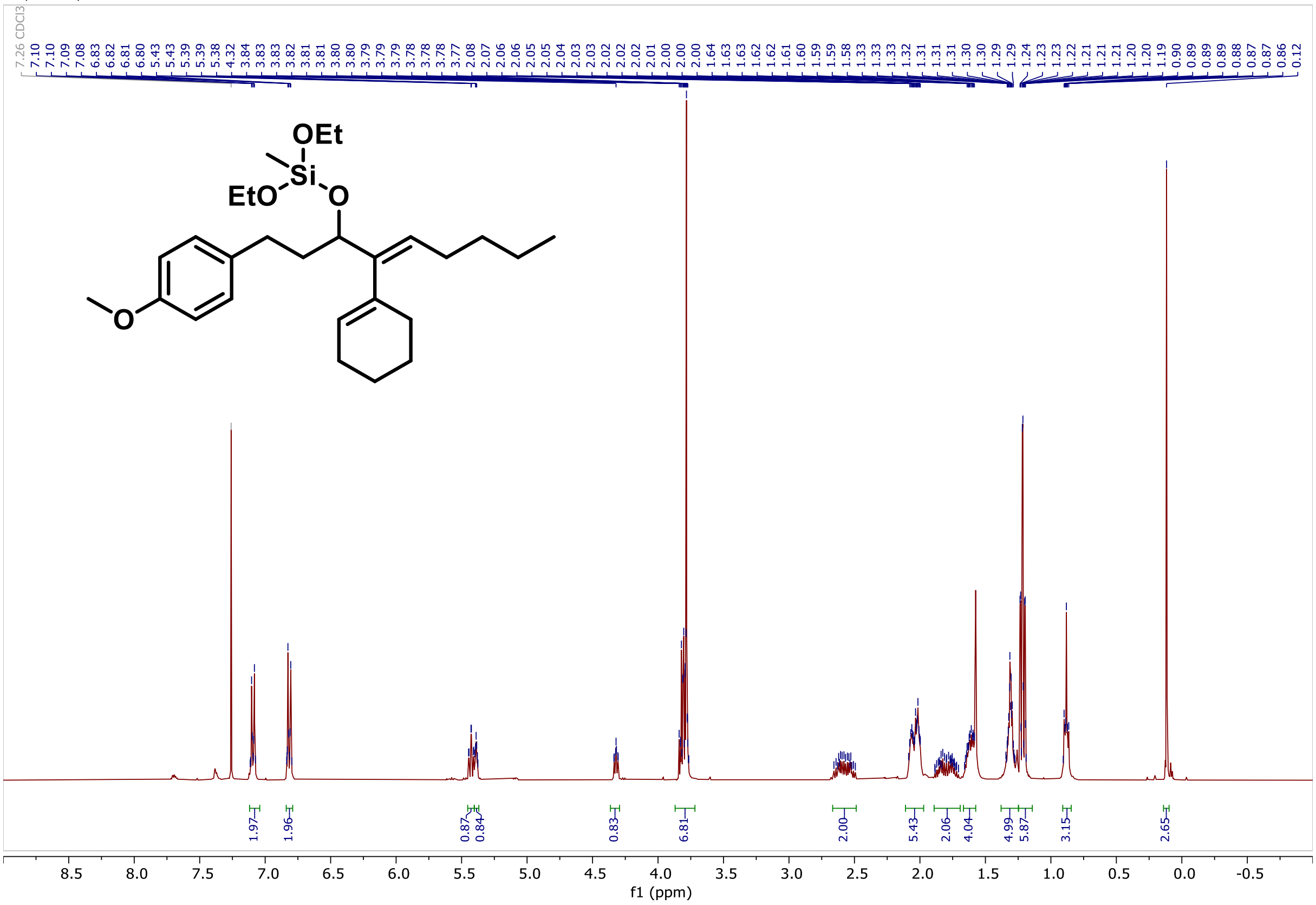

13C, CDCl3, 101 MHz

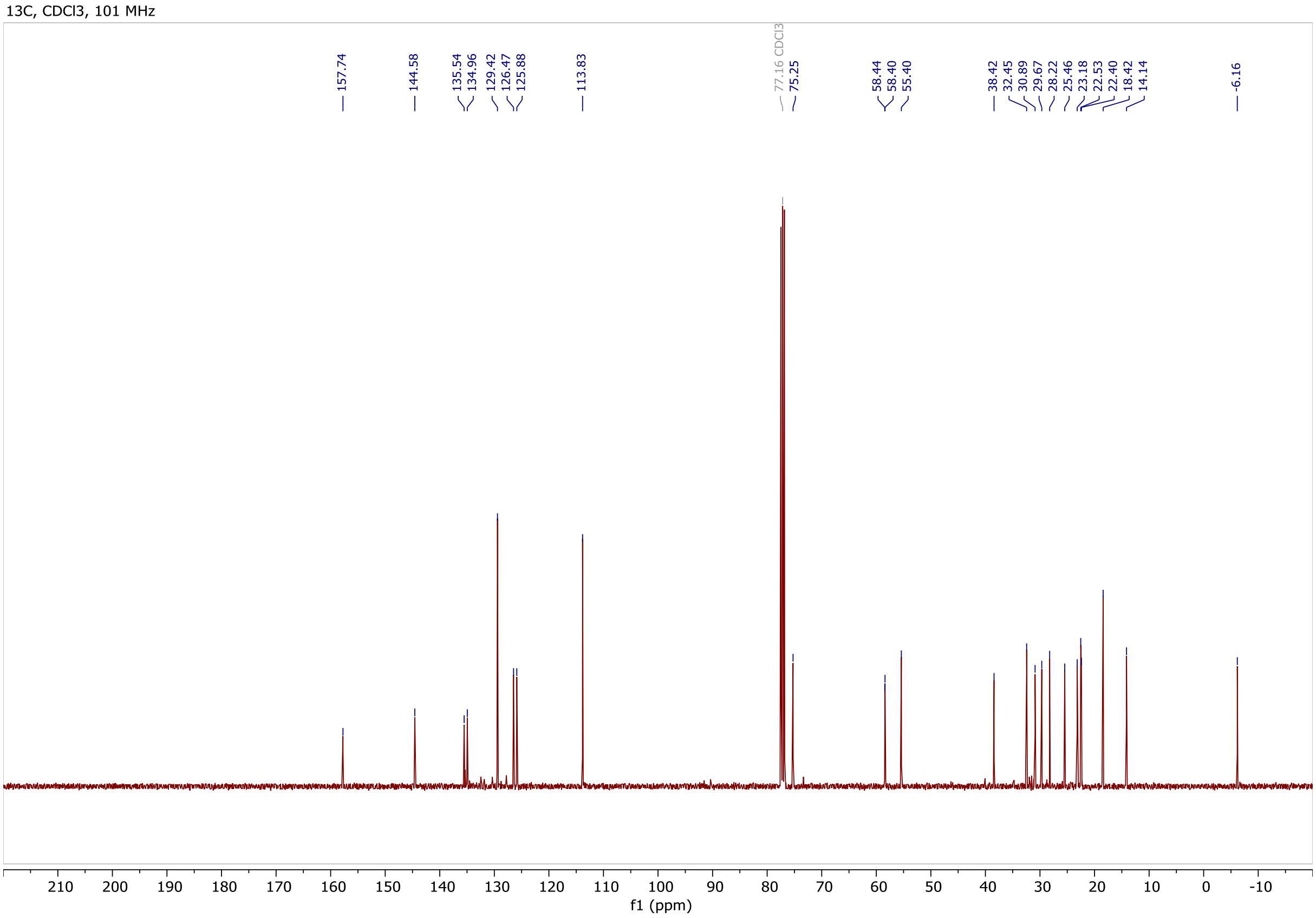

Compound 19

1H, CDCl3, 400 MHz

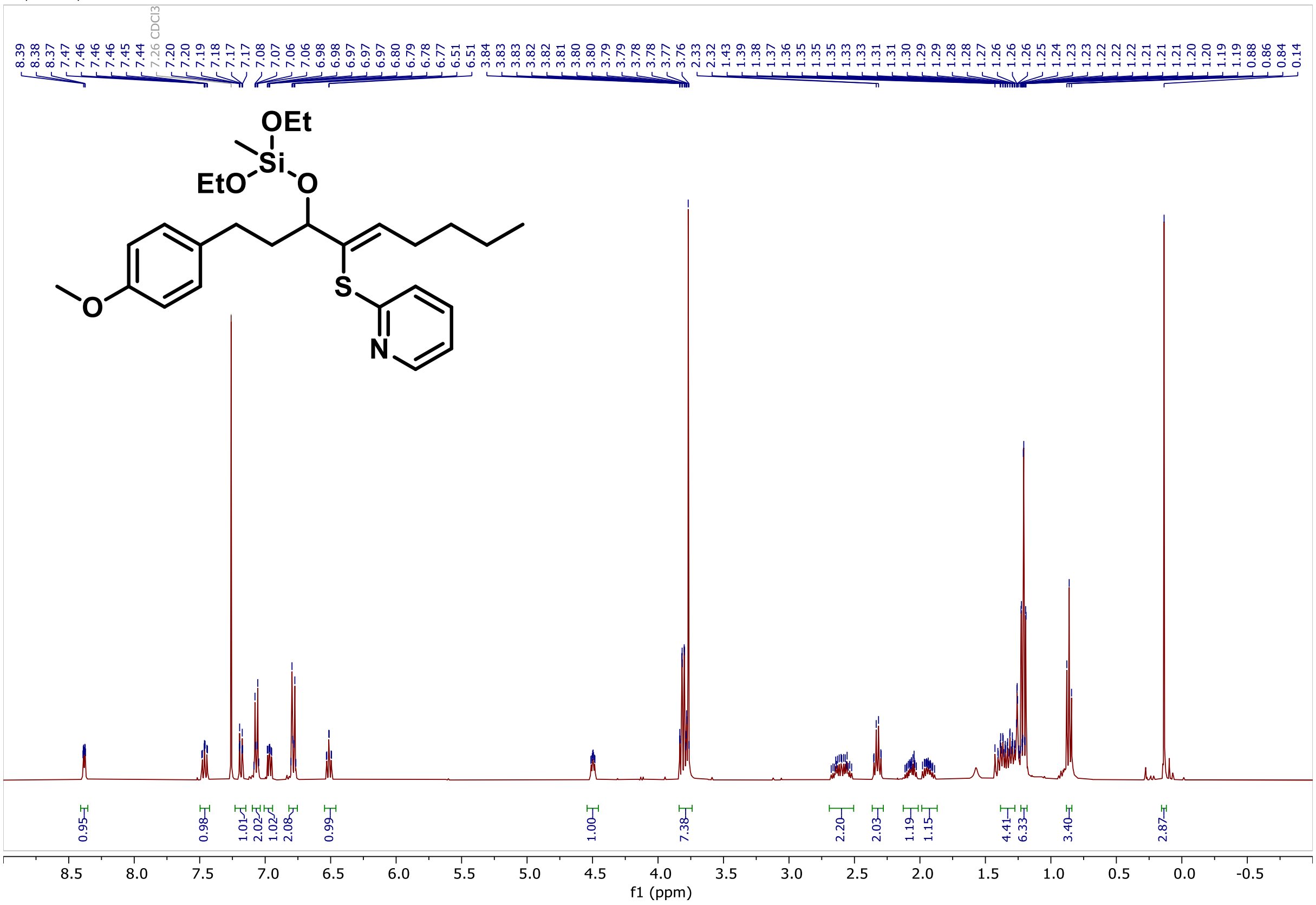

13C, CDCl3, 101 MHz

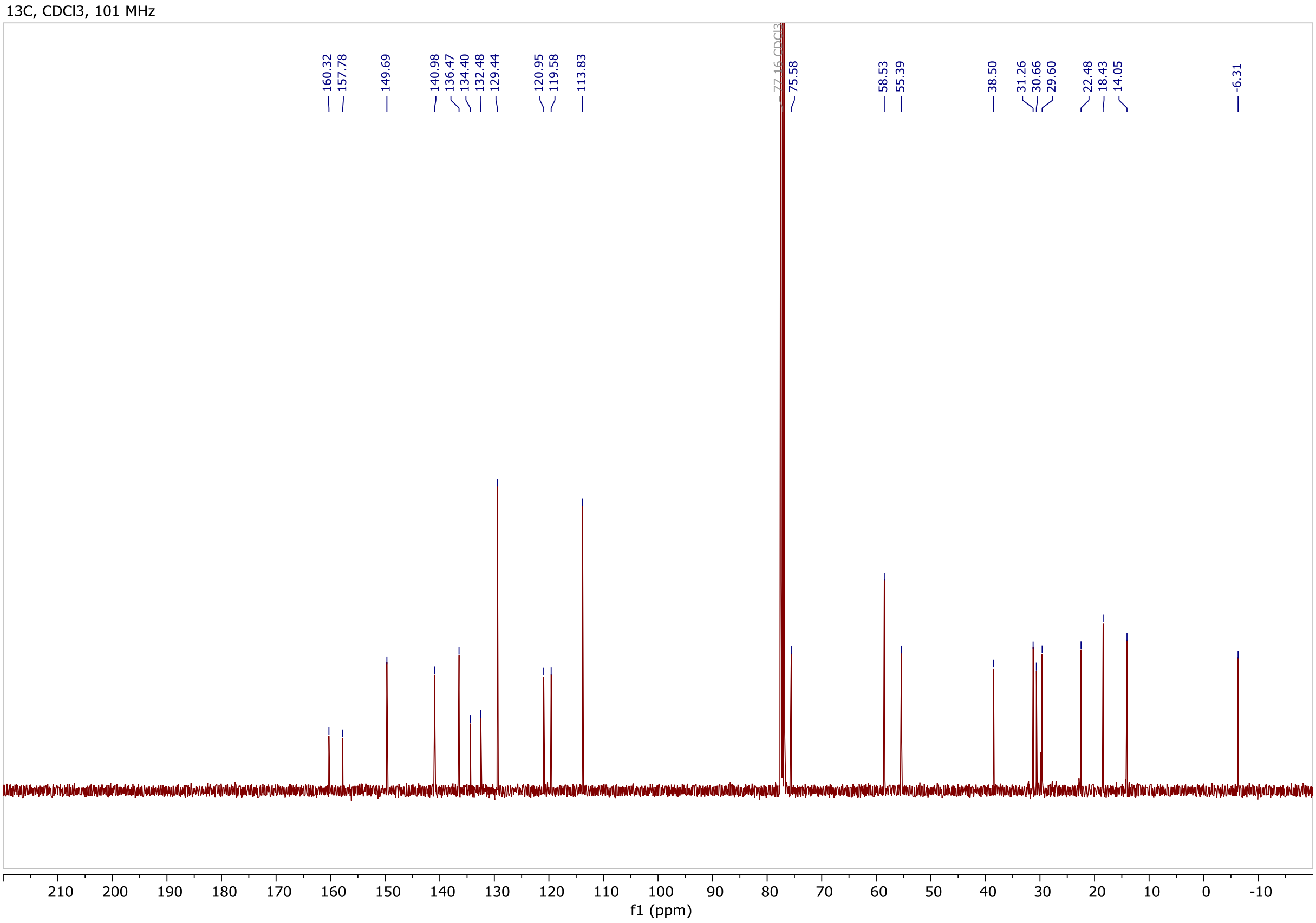

Compound 21

1H, CDCl3, 400 MHz

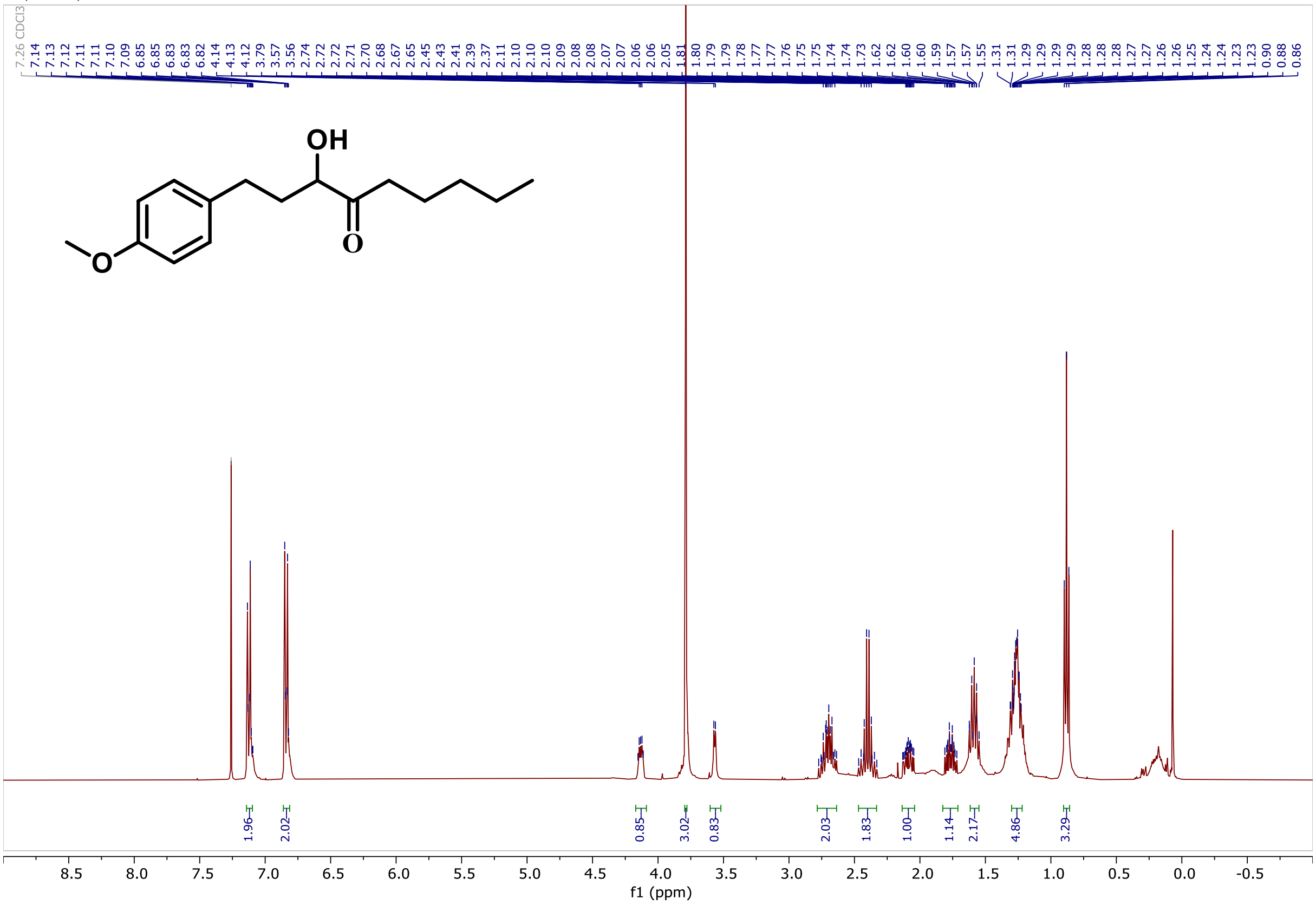

13C, CDCl3, 101 MHz

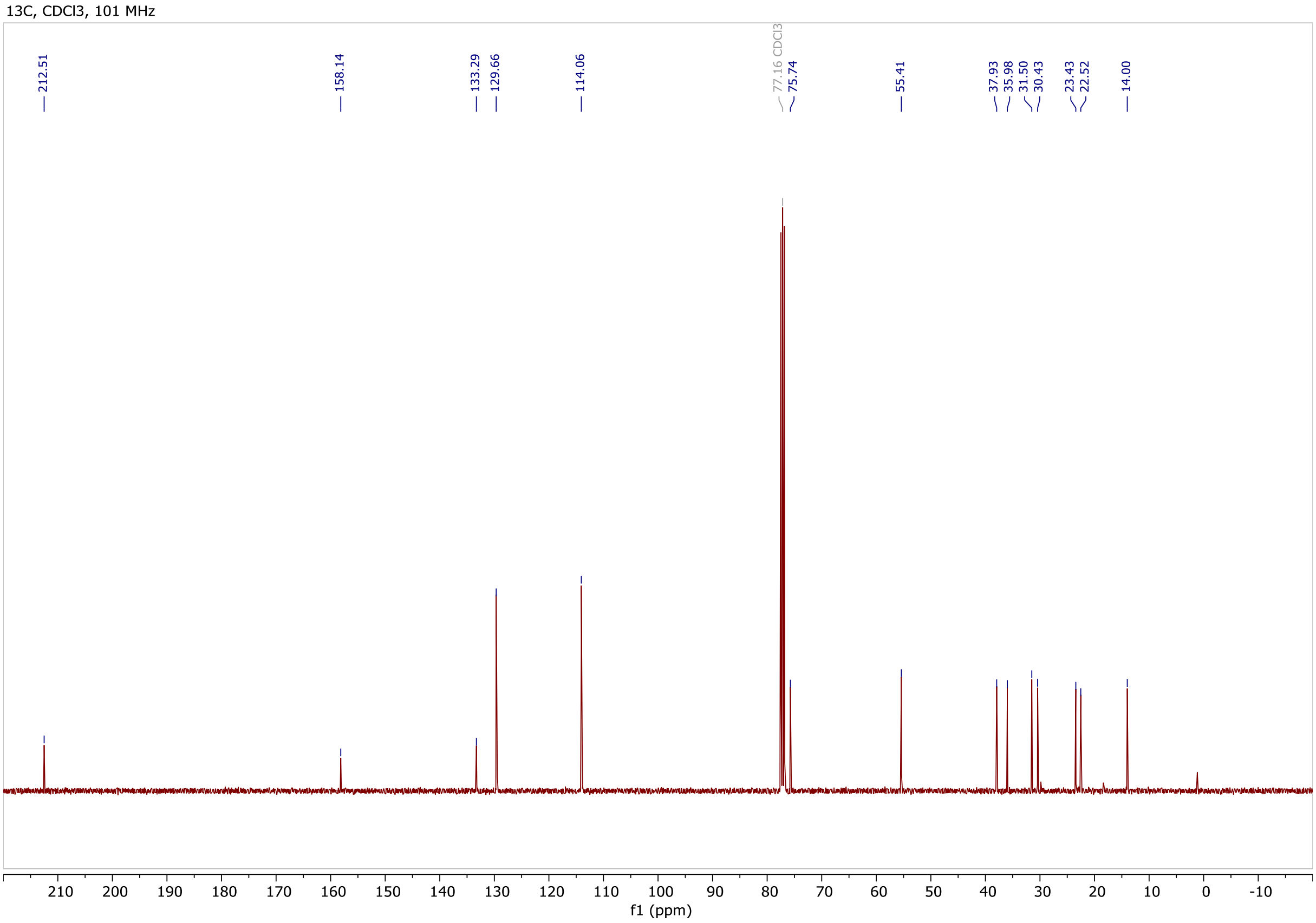

Compound 22

1H, CDCl3, 400 MHz

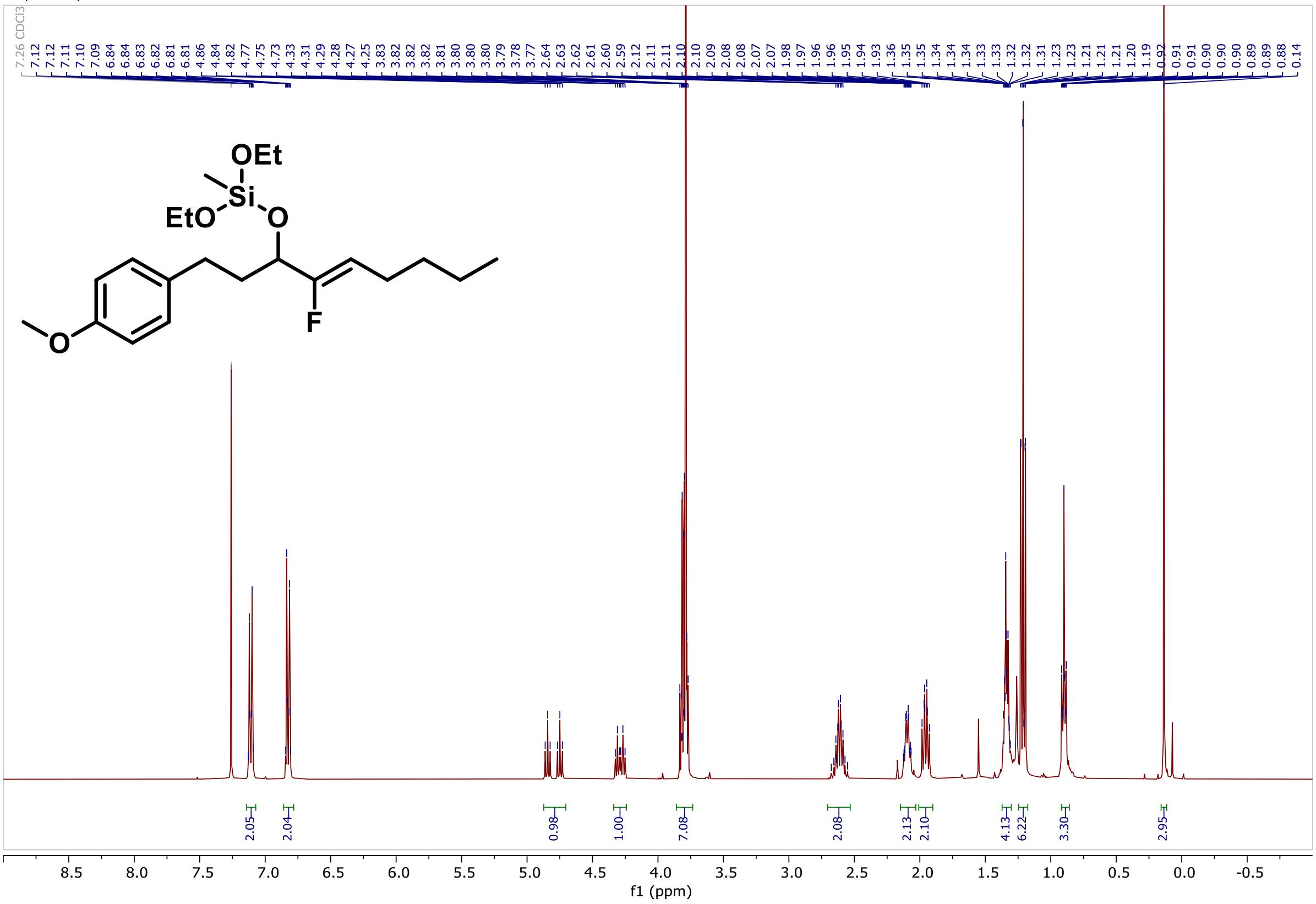

13C, CDCl3, 101 MHz

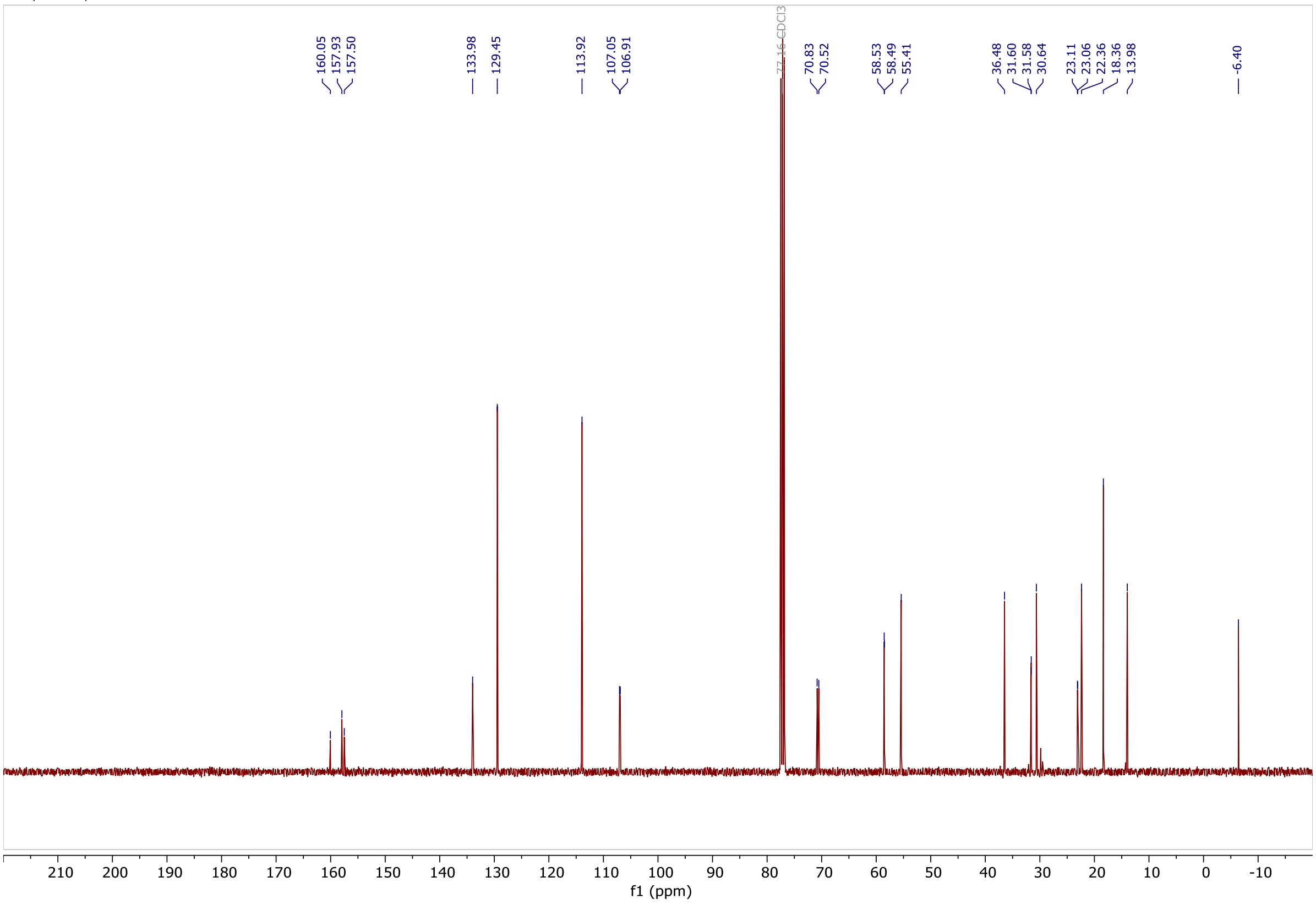

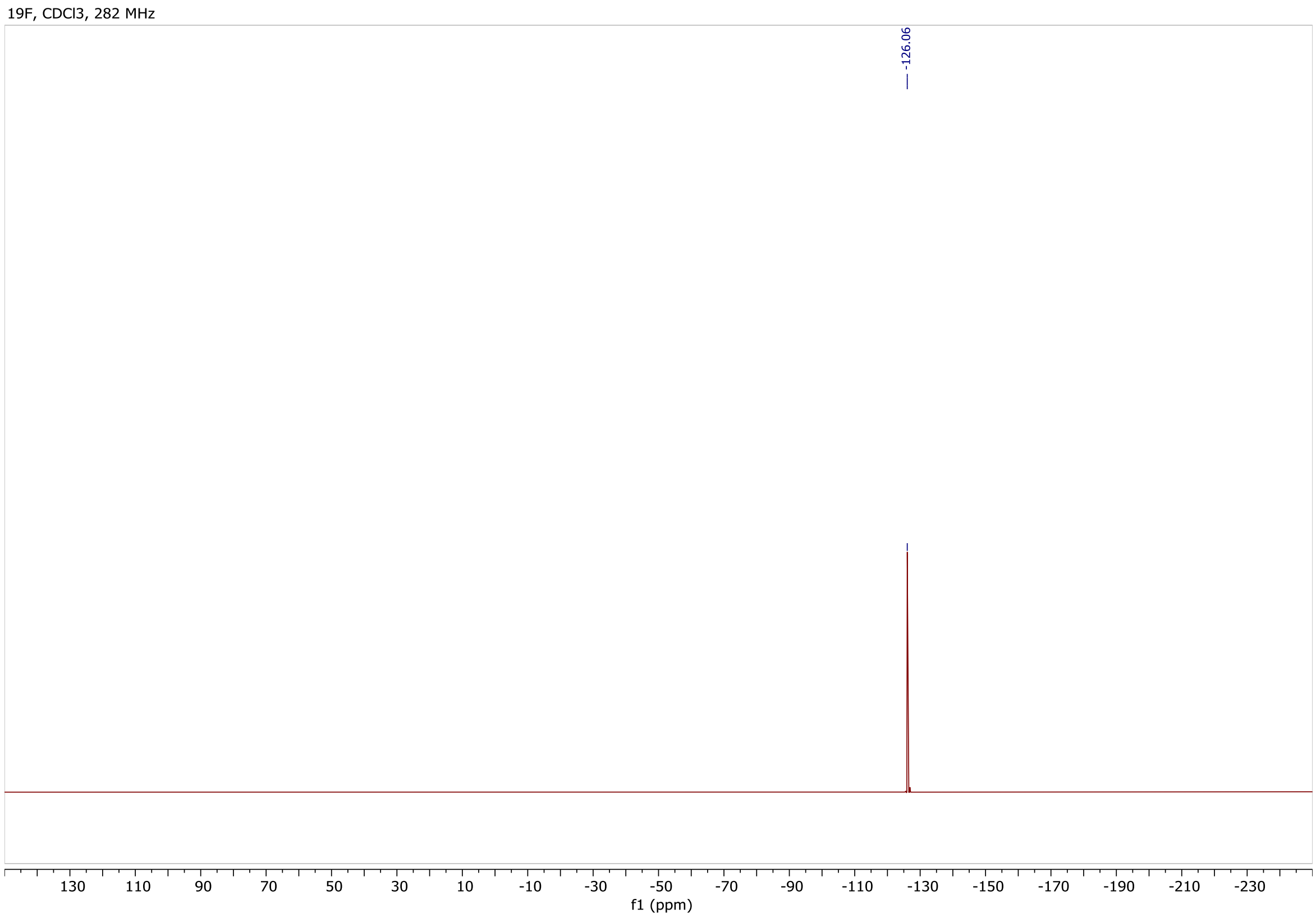
19F, CDCl3, 282 MHz
-126.06
130
110
90
70
50
30
10
-10
-30
-50
-70
-90
-110
-130
-150
-170
-190
-210
-230
f1 (ppm)

Compound 23

1H, CDCl3, 600 MHz

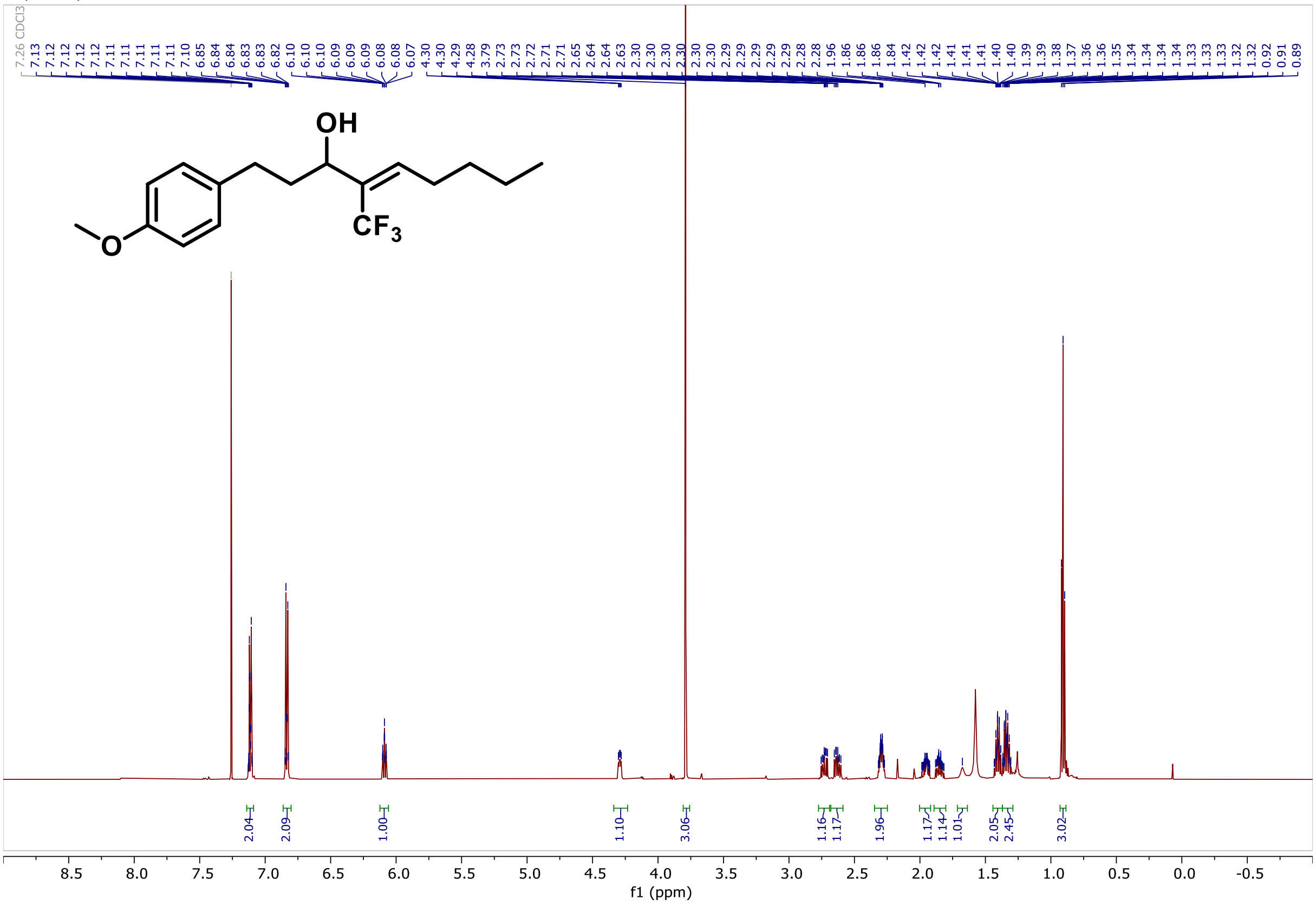

13C, CDCl3, 151 MHz

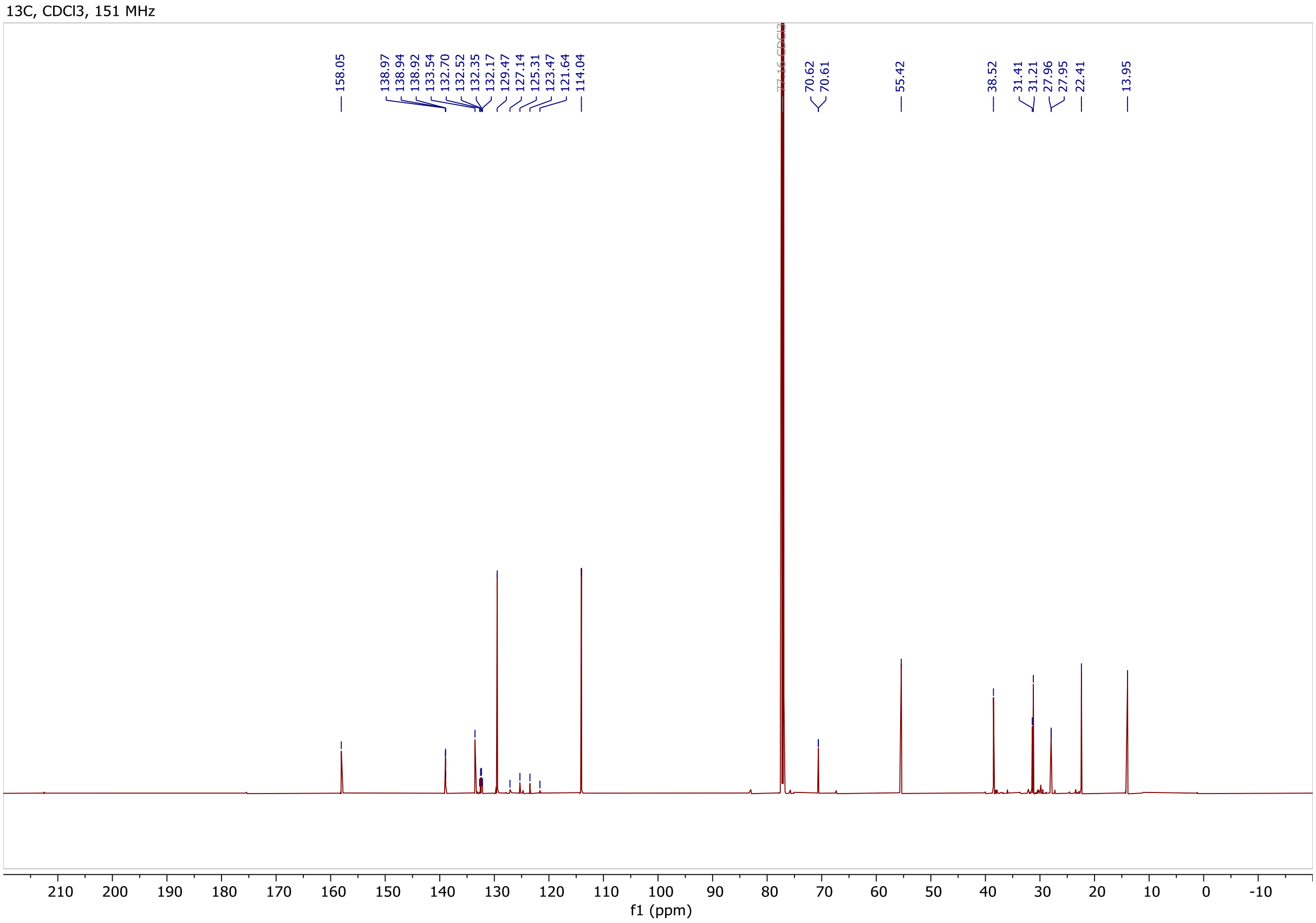

19F, CDCl3, 565 MHz

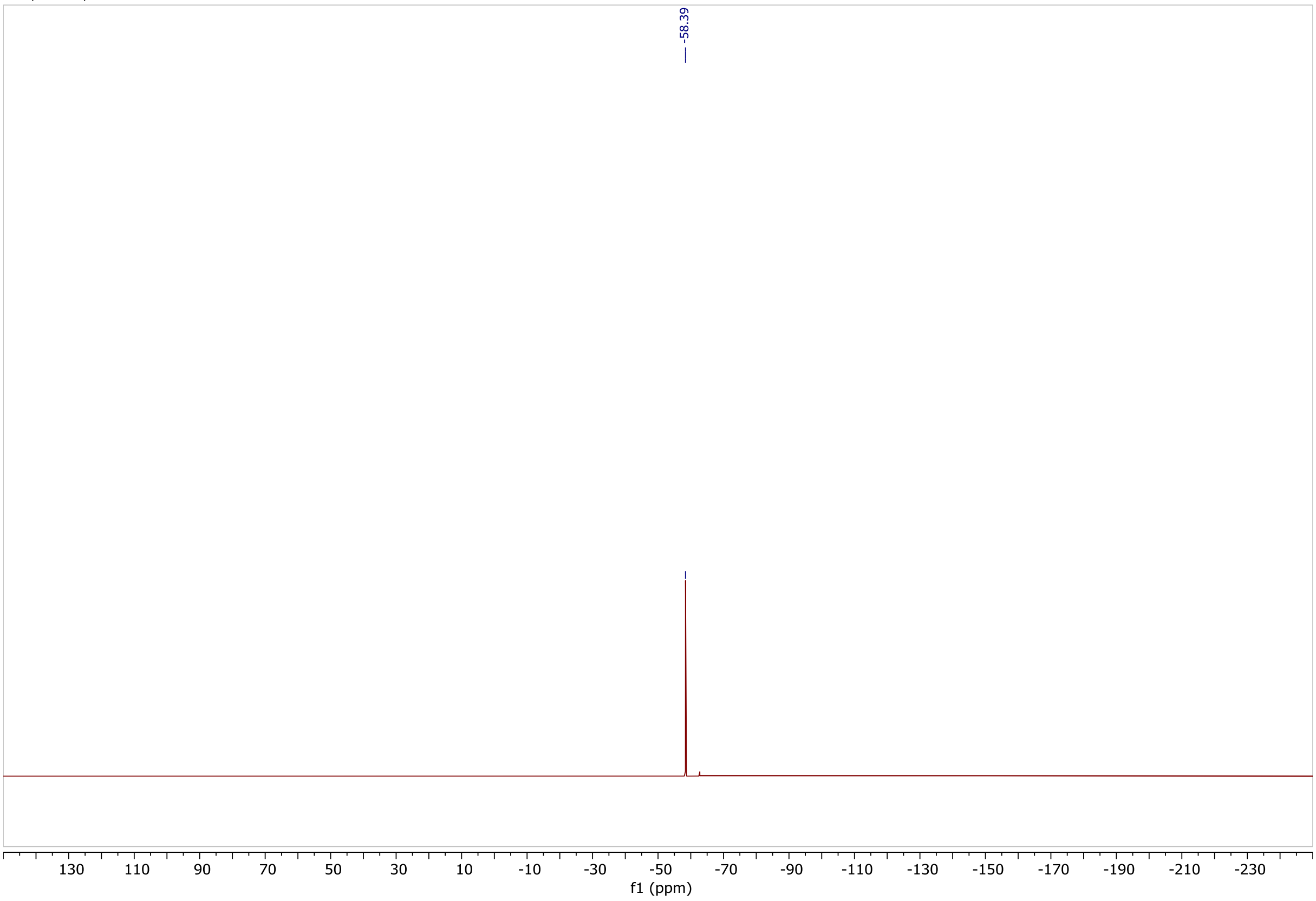

Compound 24

1H, CDCl3, 400 MHz

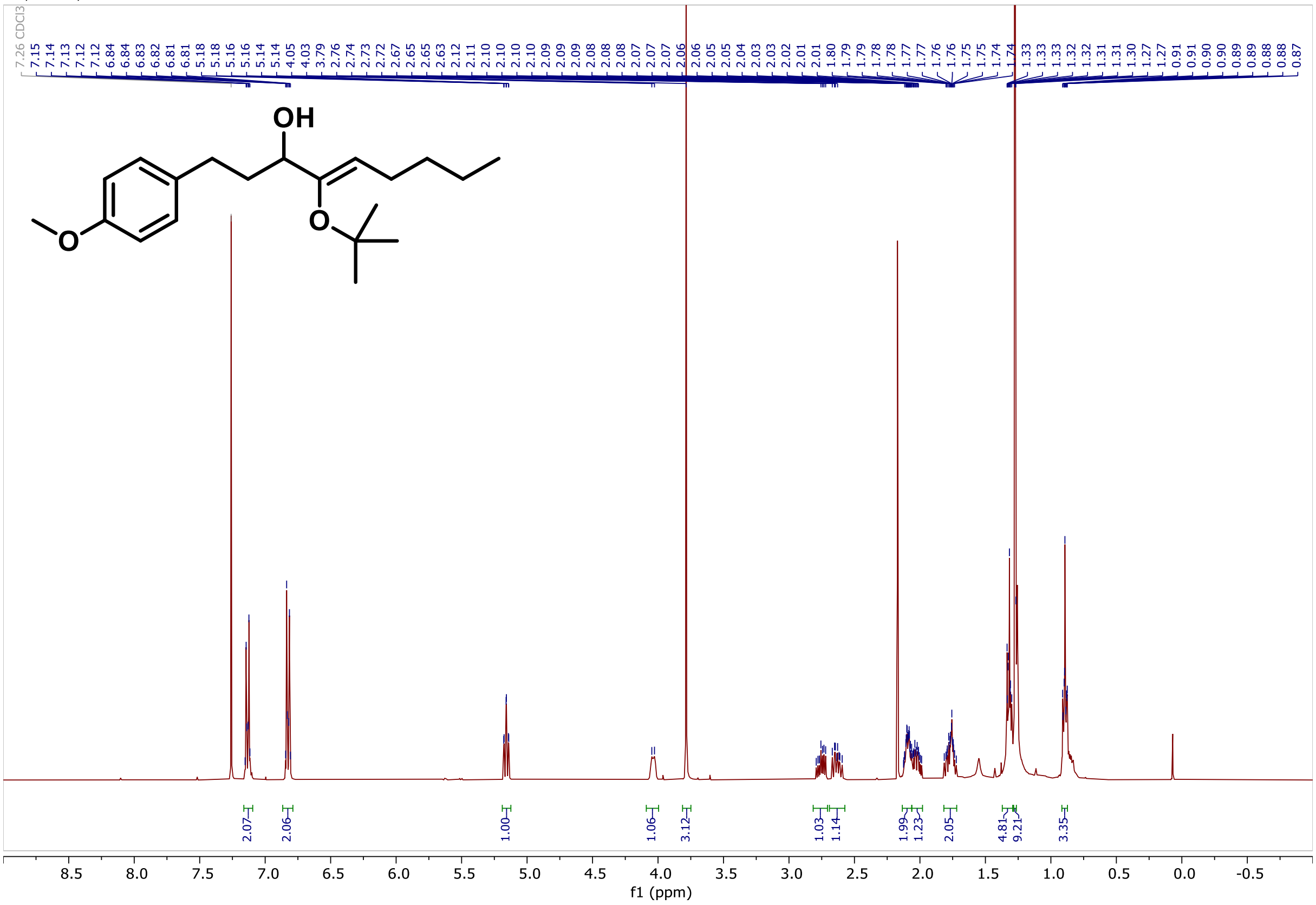

13C, CDCl3, 101 MHz

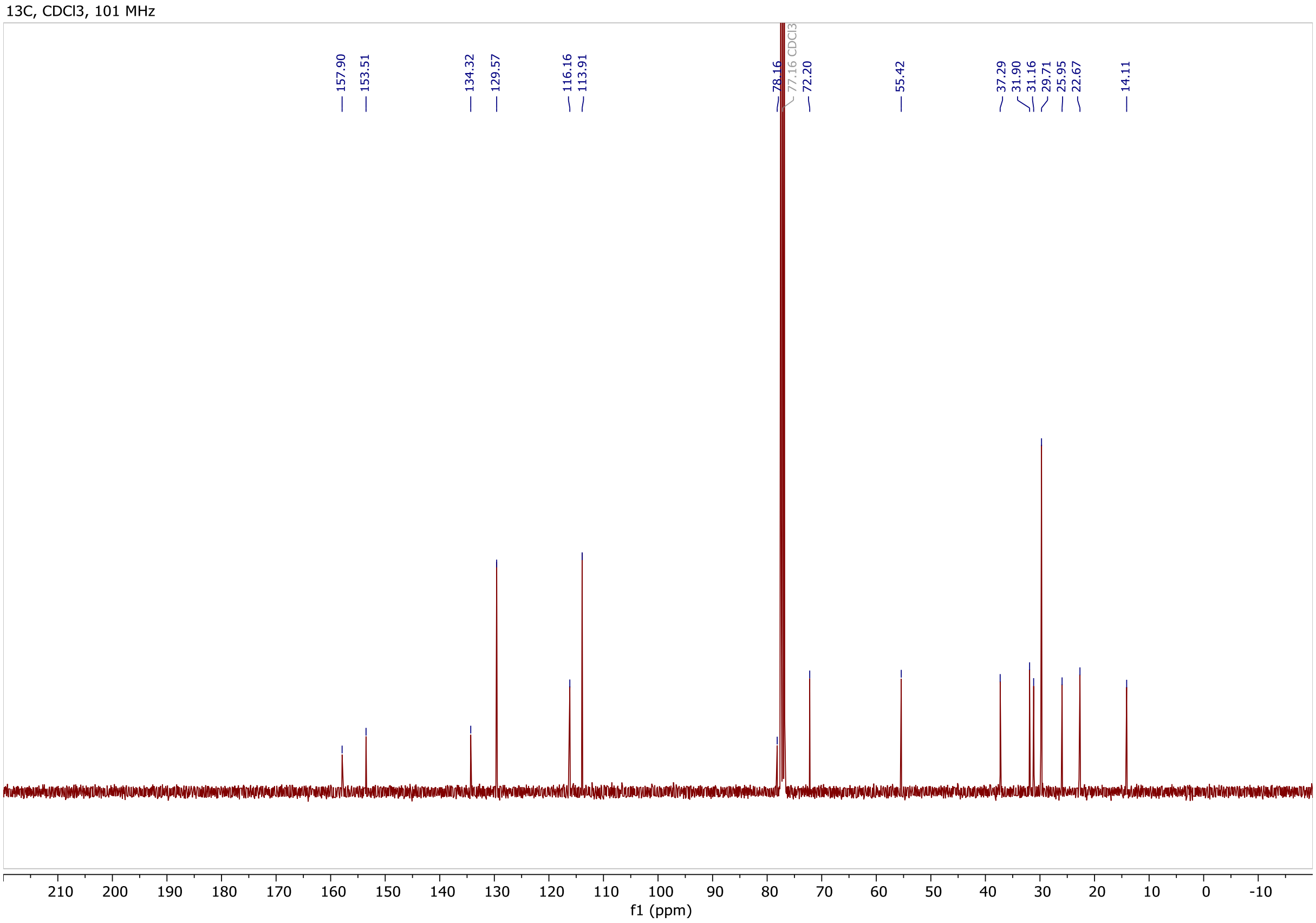

Compound 25

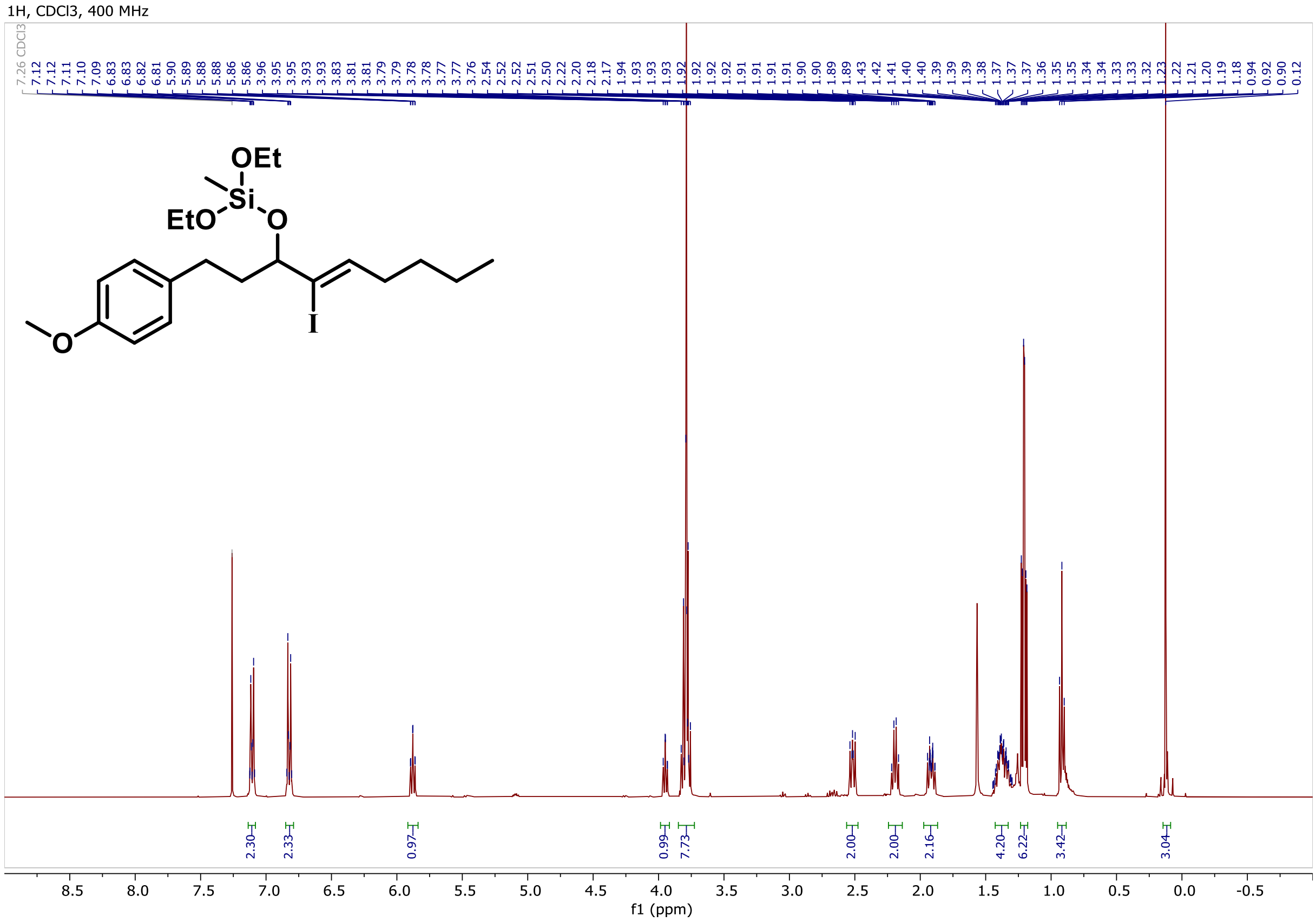

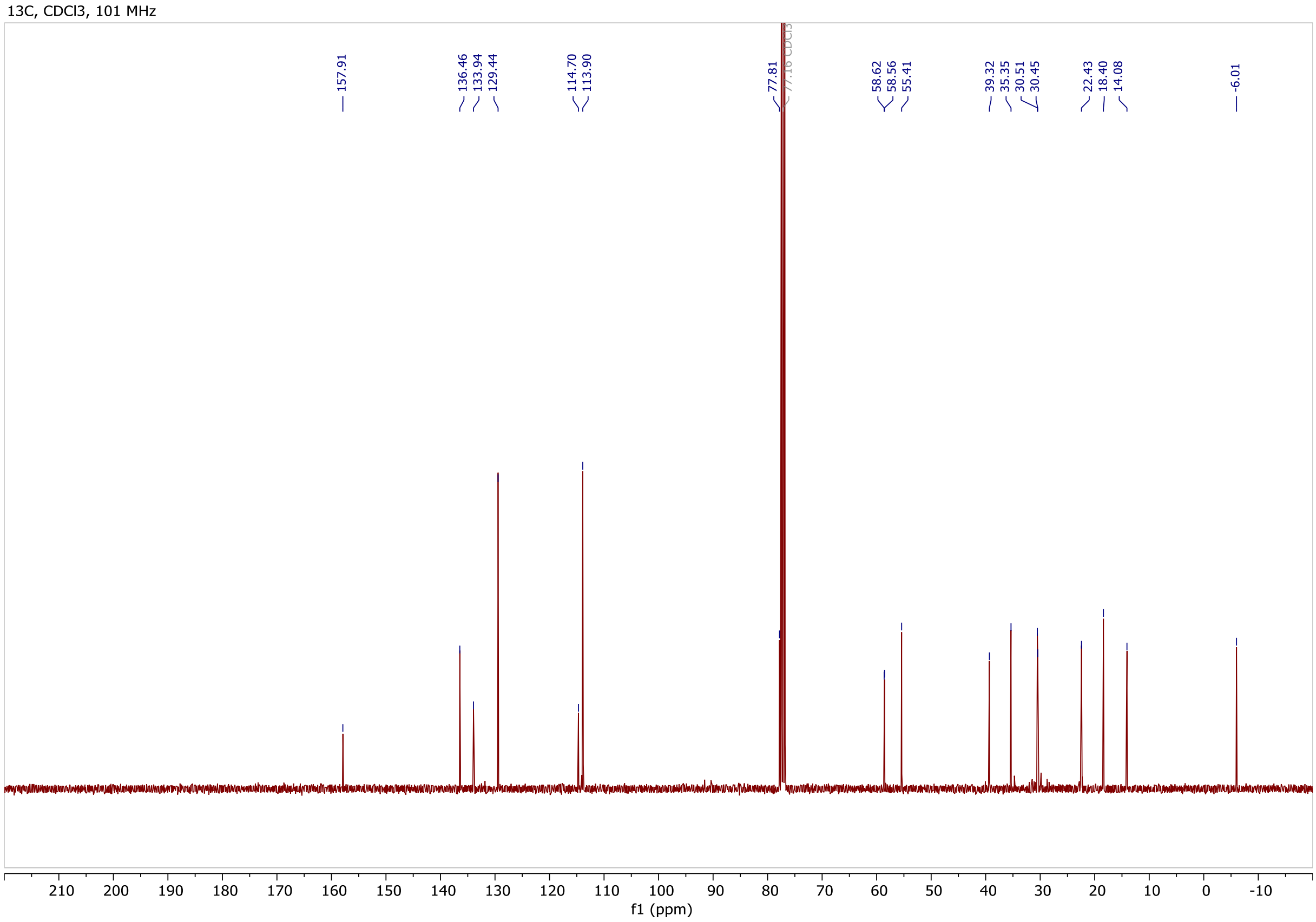

Compound 26

1H, CDCl3, 400 MHz

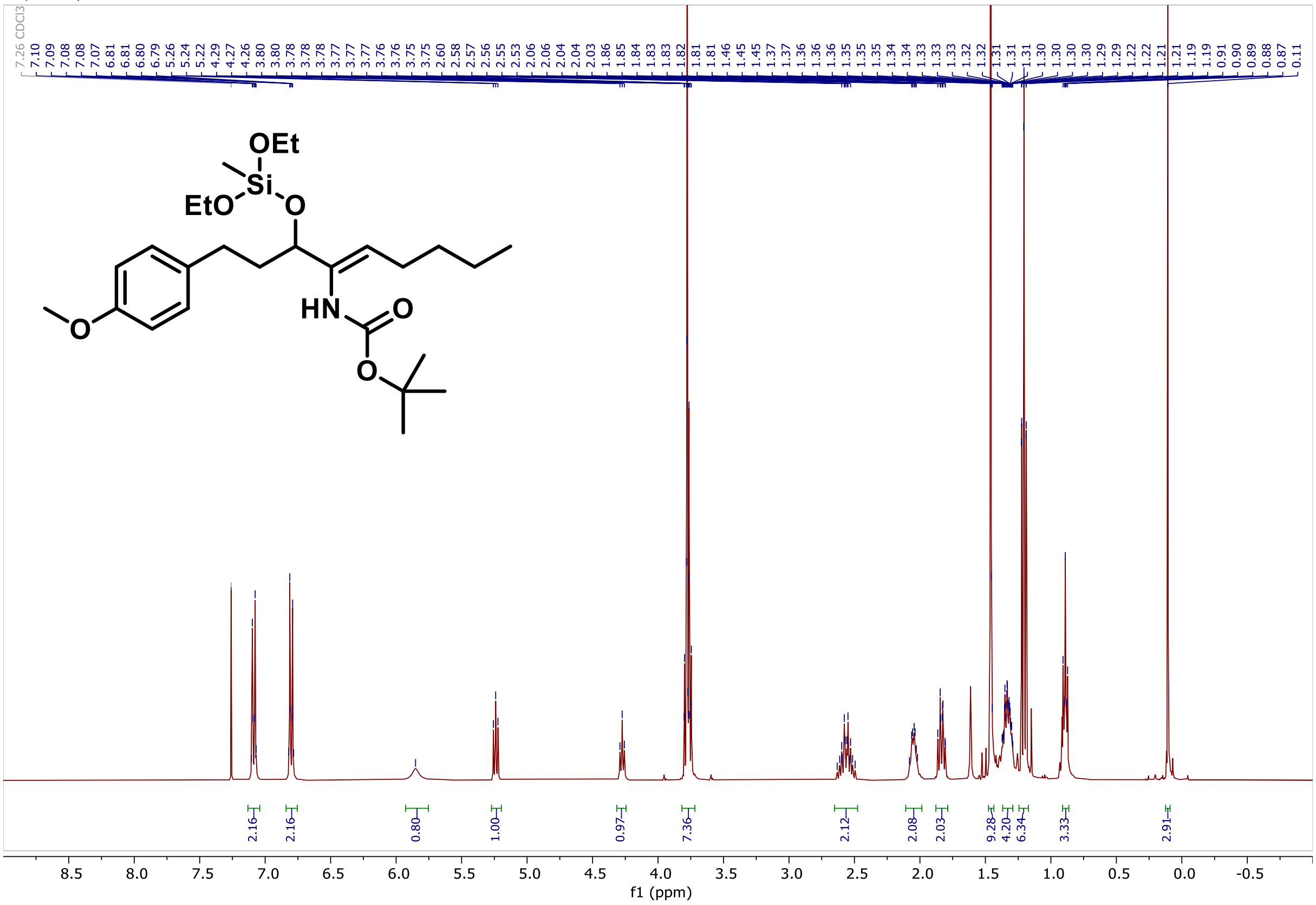

13C, CDCl3, 101 MHz

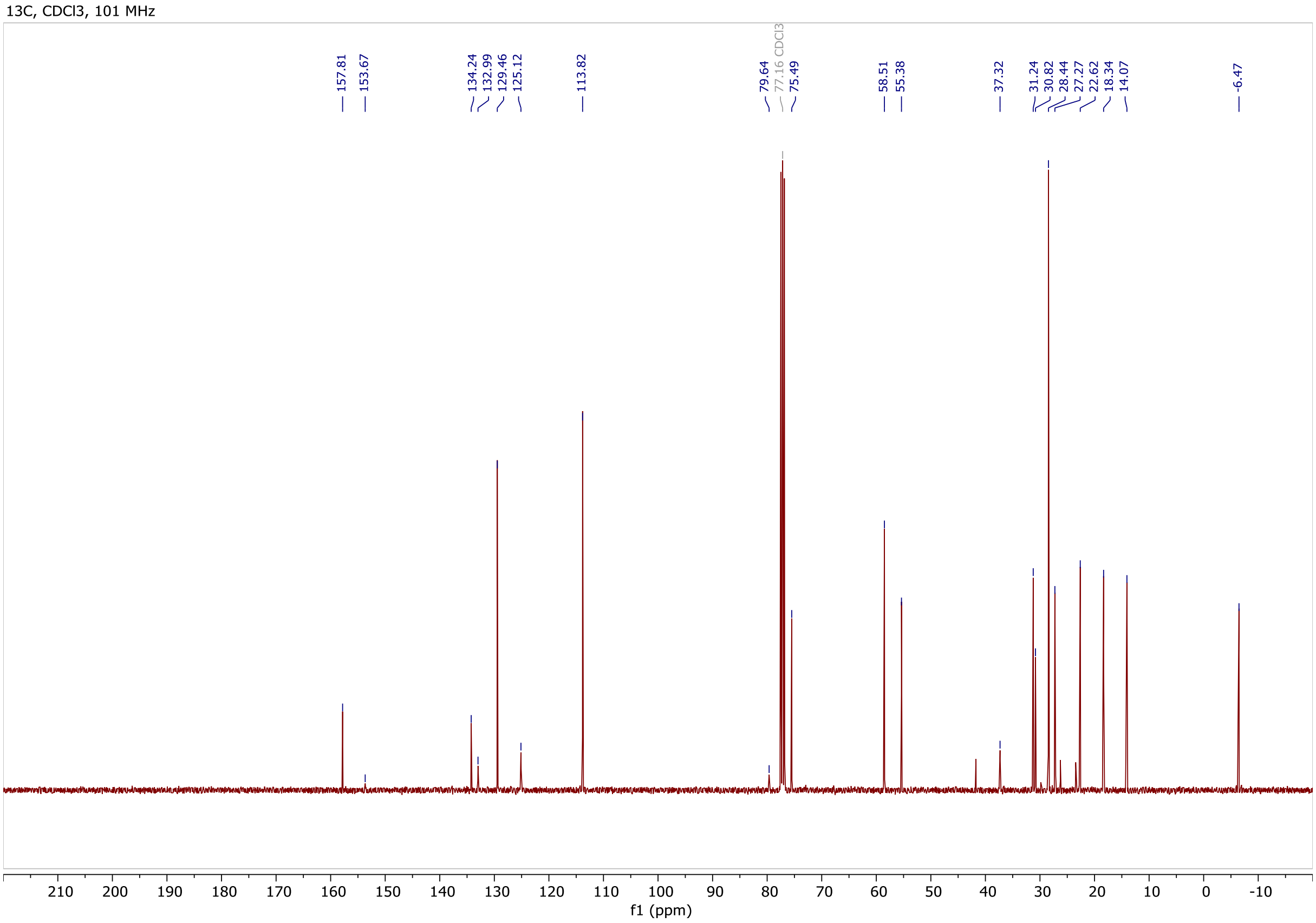

References

[1] A. Fujii, S. Hashiguchi, N. Uematsu, T. Ikariya, R. Noyori, *J. Am. Chem. Soc.* **1996**, *118*, 2521-2522.

[2] K. Matsumura, S. Hashiguchi, T. Ikariya, R. Noyori, *J. Am. Chem. Soc.* **1997**, *119*, 8738-8739.

[3] M. M. Midland, D. C. McDowell, R. L. Hatch, A. Tramontano, *J. Am. Chem. Soc.* **1980**, *102*, 867-869.

[4] M. M. Midland, R. S. Graham, Org. Synth. 1985, 63, 57.

[5] V. Laserna, M. J. Porter, T. D. Sheppard, *J. Org. Chem.* **2019**, *84*, 11391-11406.

[6] A. G. Myers, B. Zheng, *J. Am. Chem. Soc.* **1996**, *118*, 4492-4493.

[7] R. J. Armstrong, M. Nandakumar, R. M. P. Dias, A. Noble, E. L. Myers, V. K. Aggarwal, *Angew. Chem. Int. Ed.* **2018**, *57*, 8203-8208.

[8] T. Umemoto, K. Kawada, K. Tomita, *Tetrahedron Lett.* **1986**, *27*, 4465-4468.
